# The genomes of 5 underutilized Papilionoideae crops provide insights into root nodulation and disease resistance

**DOI:** 10.1093/gigascience/giae063

**Published:** 2024-08-27

**Authors:** Lihua Yuan, Lihong Lei, Fan Jiang, Anqi Wang, Rong Chen, Hengchao Wang, Sihan Meng, Wei Fan

**Affiliations:** Guangdong Laboratory for Lingnan Modern Agriculture (Shenzhen Branch), Genome Analysis Laboratory of the Ministry of Agriculture and Rural Affairs, Agricultural Genomics Institute at Shenzhen, Chinese Academy of Agricultural Sciences, Shenzhen, Guangdong 518120, China; State Key Laboratory of Crop Stress Adaptation and Improvement, School of Life Sciences, Henan University, Kaifeng 475004, China; Shenzhen Research Institute of Henan University, Shenzhen 518000, China; Guangdong Laboratory for Lingnan Modern Agriculture (Shenzhen Branch), Genome Analysis Laboratory of the Ministry of Agriculture and Rural Affairs, Agricultural Genomics Institute at Shenzhen, Chinese Academy of Agricultural Sciences, Shenzhen, Guangdong 518120, China; State Key Laboratory of Crop Stress Adaptation and Improvement, School of Life Sciences, Henan University, Kaifeng 475004, China; Shenzhen Research Institute of Henan University, Shenzhen 518000, China; Guangdong Laboratory for Lingnan Modern Agriculture (Shenzhen Branch), Genome Analysis Laboratory of the Ministry of Agriculture and Rural Affairs, Agricultural Genomics Institute at Shenzhen, Chinese Academy of Agricultural Sciences, Shenzhen, Guangdong 518120, China; Guangdong Laboratory for Lingnan Modern Agriculture (Shenzhen Branch), Genome Analysis Laboratory of the Ministry of Agriculture and Rural Affairs, Agricultural Genomics Institute at Shenzhen, Chinese Academy of Agricultural Sciences, Shenzhen, Guangdong 518120, China; Guangdong Laboratory for Lingnan Modern Agriculture (Shenzhen Branch), Genome Analysis Laboratory of the Ministry of Agriculture and Rural Affairs, Agricultural Genomics Institute at Shenzhen, Chinese Academy of Agricultural Sciences, Shenzhen, Guangdong 518120, China; Guangdong Laboratory for Lingnan Modern Agriculture (Shenzhen Branch), Genome Analysis Laboratory of the Ministry of Agriculture and Rural Affairs, Agricultural Genomics Institute at Shenzhen, Chinese Academy of Agricultural Sciences, Shenzhen, Guangdong 518120, China; Guangdong Laboratory for Lingnan Modern Agriculture (Shenzhen Branch), Genome Analysis Laboratory of the Ministry of Agriculture and Rural Affairs, Agricultural Genomics Institute at Shenzhen, Chinese Academy of Agricultural Sciences, Shenzhen, Guangdong 518120, China; Guangdong Laboratory for Lingnan Modern Agriculture (Shenzhen Branch), Genome Analysis Laboratory of the Ministry of Agriculture and Rural Affairs, Agricultural Genomics Institute at Shenzhen, Chinese Academy of Agricultural Sciences, Shenzhen, Guangdong 518120, China

**Keywords:** Papilionoideae, underutilized legume, whole genome duplication, root-nodule symbiosis, R genes

## Abstract

**Background:**

The Papilionoideae subfamily contains a large amount of underutilized legume crops, which are important for food security and human sustainability. However, the lack of genomic resources has hindered the breeding and utilization of these crops.

**Results:**

Here, we present chromosome-level reference genomes for 5 underutilized diploid Papilionoideae crops: sword bean (*Canavalia gladiata*), scarlet runner bean (*Phaseolus coccineus*), winged bean (*Psophocarpus tetragonolobus*), smooth rattlebox (*Crotalaria pallida*), and butterfly pea (*Clitoria ternatea*), with assembled genome sizes of 0.62 Gb, 0.59 Gb, 0.71 Gb, 1.22 Gb, and 1.72 Gb, respectively. We found that the long period of higher long terminal repeat retrotransposon activity is the major reason that the genome size of smooth rattlebox and butterfly pea is enlarged. Additionally, there have been no recent whole-genome duplication (WGD) events in these 5 species except for the shared papilionoid-specific WGD event (∼55 million years ago). Then, we identified 5,328 and 10,434 species-specific genes between scarlet runner bean and common bean, respectively, which may be responsible for their phenotypic and functional differences and species-specific functions. Furthermore, we identified the key genes involved in root-nodule symbiosis (RNS) in all 5 species and found that the *NIN* gene was duplicated in the early Papilionoideae ancestor, followed by the loss of 1 gene copy in smooth rattlebox and butterfly pea lineages. Last, we identified the resistance (R) genes for plant defenses in these 5 species and characterized their evolutionary history.

**Conclusions:**

In summary, this study provides chromosome-scale reference genomes for 3 grain and vegetable beans (sword bean, scarlet runner bean, winged bean), along with genomes for a green manure crop (smooth rattlebox) and a food dyeing crop (butterfly pea). These genomes are crucial for studying phylogenetic history, unraveling nitrogen-fixing RNS evolution, and advancing plant defense research.

## Introduction

Papilionoideae, the largest subfamily in Fabaceae (Legume) [1] whose name probably originated from its flower’s resemblance to a butterfly (Latin: Papilio), has an extremely important position in agriculture and makes great contributions to the human diet and food security. In addition to the several well-known crops such as soybean [2], peanut [3], faba bean [4], mung bean [5], pea [6], common bean [7], and alfalfa [8], this subfamily also includes many other underutilized crops [9]. For example, sword bean (*Canavalia gladiata*: NCBI:txid3824), scarlet runner bean (*Phaseolus coccineus*: NCBI:txid412098), and winged bean (*Psophocarpus tetragonolobus*: NCBI:txid3891) are both grain and vegetable plants: the mature bean seeds are protein-rich grains, while the young bean pods are used as vegetables. Smooth rattlebox (*Crotalaria pallida*: NCBI:txid1127389) and butterfly pea (*Clitoria ternatea*; NCBI:txid43366) are often used as green manure and forage grass due to their high protein content. The dried flowers of butterfly pea are also used as a natural food colorant (blue), which is popular in Southeast Asian countries [10]. In addition, sword bean has also been used as a traditional medicine to improve poor appetite and alleviate vomiting in China for thousands of years [11], and scientists found that smooth rattlebox also has antitumor properties in recent years [12].

The Papilionoideae plants also play a unique ecological role in nitrogen fixation through symbiotic root nodules [13], which is indispensable for the global nitrogen cycle. The ability to nitrogen fixation from the atmosphere also helps agriculture production use fewer synthetic fertilizers, thereby reducing the energy consumption and mitigating soil pollution [14]. As a model of special host–bacteria interaction, the formation of nitrogen-fixing root nodules has been intensively studied on 2 model species, *Medicago truncatula* and *Lotus japonicus* [15, 16], both belonging to the subfamily Papilionoideae. The host plants excrete flavonoids into the rhizosphere and induce the rhizobia to express the nodulation (nod) genes [13]. Then, the metabolite products of these nod genes (Nod factors) are sensed by the host plants to start nodulation, which requires the coordination of rhizobial infection at the root epidermis with cell division in the cortex [17]. Inside the nodule, rhizobia live in an organelle-like structure known as symbiosome, and the host plants secrete leghemoglobin (Lb) to maintain a low-oxygen environment in order to facilitate the nitrogen-fixing reactions in symbiosomes [18]. Recent phylogenomics and phylotranscriptomics studies have shown a single origin of nitrogen-fixing root-nodule symbiosis (RNS), followed by multiple independent losses, occurred in various lineages [19, 20].

Resistance (R) gene-mediated defense plays an important role in plant defenses against all pathogens. It recognizes the pathogen-derived proteins, referred to as effectors, and induces a state in the host, defined as effector-triggered susceptibility (ETS), which in turn leads to the local hypersensitive response (HR) cell death to restrict pathogen growth and propagation [21]. Most cloned R genes encode intracellular nucleotide-binding, leucine-rich-repeat (NLR) receptors, which are typically composed of 3 domains: a central NB (NB-ARC) domain, bordered by a C-terminal leucine-rich repeat domain (LRR), and an N-terminal coiled-coil (CC) or Toll/interleukin 1 receptor (TIR) or resistance to powdery mildew (RPW8) domain [22]. The RPW8 domain is rare in comparison to the 2 major CC and TIR domains.

For the purpose of nodulation studies and crop breeding, tens of agriculturally important plants in the subfamily Papilionoideae have been sequenced, including all the above well-known species, as well as adzuki bean [23], lablab bean [24], velvet bean [25], kudzu vine [26], pagoda tree [27], and winged bean (Ma3 cultivar) [28]. However, the subfamily contains many other rare but valuable species, which still lack reference genomes, hindering the in-depth biological studies and exploitation of these species. Here, we present the chromosome-scale reference genomes for 3 grain and vegetable beans (sword bean, scarlet runner bean, winged bean), a green manure crop (smooth rattlebox), and a food dyeing crop (butterfly pea) to investigate the phylogenetic history, explore the evolution of RNS, and identify the R genes involved in controlling crop diseases.

## Results

### Chromosome-scale assembly of 5 underutilized legumes

We generated 67 Gb (103×), 70 Gb (119×), 64 Gb (93×), 69 Gb (52×), and 167 Gb (95×) HiFi data for sword bean, scarlet runner bean, winged bean, smooth rattlebox, and butterfly pea, respectively (Supplementary Table S1). Analyzing the distribution of *k*-mer frequencies [29], we found that all sequenced materials are highly homozygous and the estimated genome size is 0.65 Gb, 0.59 Gb, 0.69 Gb, 1.33 Gb, and 1.76 Gb for each species (Supplementary Fig. S1). Then, the HiFi data were assembled into large contigs with total size 0.62 Gb, 0.59 Gb, 0.71 Gb, 1.22 Gb, and 1.72 Gb, which were further linked into 11, 11, 9, 8, and 8 chromosome-scale scaffolds by Hi-C data for sword bean, scarlet runner bean, winged bean, smooth rattlebox, and butterfly pea, respectively (Supplementary Tables S2–S4, Supplementary Fig. S2). Overall, most chromosomes include fewer than 5 contigs, suggesting a very high continuity of our assembly (Supplementary Fig. S3). Besides, the BUSCO complete ratio is over 99%, and the quality value (QV) calculated by Merqury version 1.3 [30] is over 70 for all 5 species, indicating the high accuracy of our assembly (Table 1, Supplementary Tables S5–S6).

**Table 1:** Statistics of genome assembly and annotation.

Genomic features	*Canavalia gladiata*	*Phaseolus coccineus*	*Psophocarpus tetragonolobus*	*Crotalaria pallida*	*Clitoria ternatea*
Genome assembly					
Estimated genome size by *k*-mer (Mb)	650	593	689	1,331	1,761
Total assembly size (bp)	619,186,046	592,734,161	712,813,888	1,217,645,575	1,724,627,994
Contig N50 size (bp)	39,462,069	39,559,522	13,237,817	100,840,643	126,428,166
Scaffold N50 size (bp)	55,284,388	52,871,251	79,694,302	142,152,887	168,933,288
% of sequences anchored to chromosomes	97.5%	95.7%	93.7%	98.2%	97.5%
% of telomeres assembled	72.7%	77.3%	55.6%	93.8%	75.0%
BUSCO complete rate of the genome	99.4%	99.3%	99.2%	99.0%	99.0%
QV	70.0	74.3	69.5	69.6	72.2
Genome annotation					
Length and % of tandem repeats (bp)	105,656,450 (17.1%)	54,428,626 (9.2%)	134,417,242 (18.9%)	119,729,388 (9.8%)	130,389,725 (7.6%)
Length and % of TE sequences (bp)	341,308,218 (55%)	376,131,126 (63%)	456,822,987 (64%)	994,314,842 (82%)	148,366,6381 (86%)
Number of tRNA genes	970	1,141	1,283	1,382	2,307
Number of rRNA (5S + 18S + 28S) genes	1,535	5,030	3,020	6,268	3,158
Number of protein-coding gene models	51,158	35,523	40,081	48,759	40,267
Total CDS size and % in genome (bp)	50,888,808 (8.2%)	42,292,638 (7.1%)	43,752,003 (7.4%)	51,214,428 (4.2%)	41,669,007 (2.4%)
BUSCO complete rate of the genes	99.4%	99.6%	99.2%	98.1%	98.9%

In addition, we compared our DUOXI-ginseng cultivar assembly of winged bean (712 Mb) to the recently published Ma3 cultivar assembly of winged bean (586 Mb) and found that our assembly has a large advantage in resolving the repetitive centromere regions by using HiFi reads, while the Ma3 cultivar assembly has an advantage in the chromosome-level scaffolding of contigs by using a genetic map. The position of a chromosome segment in Chr06 of our assembly for winged bean was corrected according to the Ma3 cultivar assembly (Supplementary Fig. S4).

**Figure 1:**
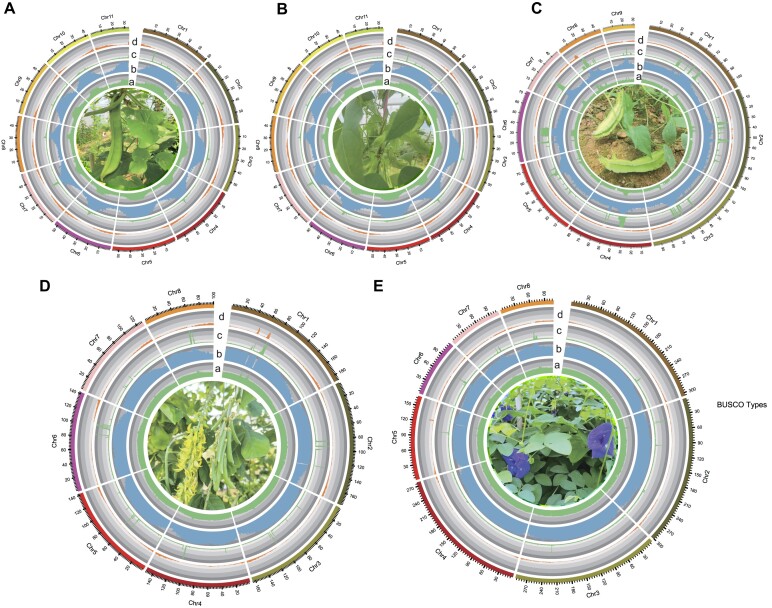
Circos plot of genomic annotations. (A) *Canavalia gladiata*, (B) *Phaseolus coccineus*, (C) *Psophocarpus tetragonolobus*, (D) *Crotalaria pallida*, and (E) *Clitoria ternatea*. The 5 circular tracks from inner to outer refer to (a) GC percentage, (b) transposable element (TE) density, (c) tandem repeats (TR) density, and (d) gene density. These features were calculated by sliding 1-Mb windows. Pictures of species are placed inside the center of the circos plot.

In total, 51,158, 35,523, 40,081, 48,759, and 40,267 protein-coding gene models were predicted in the genome of sword bean, scarlet runner bean, winged bean, smooth rattlebox, and butterfly pea, respectively (Fig. 1, Supplementary Tables S7–S9), with the coding regions covering 2.4% to 8.2% of the genome for each species. The BUSCO complete rates for the gene sets of these 5 species are comparable to those of the genomes, suggesting a high completeness of our gene annotation (Table 1). For function annotation, 72.1% to 89.0% of genes in the 5 species were annotated by at least one of the NCBI-NR, KEGG, InterPro, or Gene Ontology (GO) databases (Supplementary Table S10). In addition, we identified 970, 1,141, 1,283, 1,382 and 2,307 transfer RNA (tRNA) genes and 1,535, 5,030, 3,020, 6,268 and 3,158 ribosomal RNA (rRNA) genes for the five species (Supplementary Table S11).

### LTR-RT activity influences the genome size

**Figure 2:**
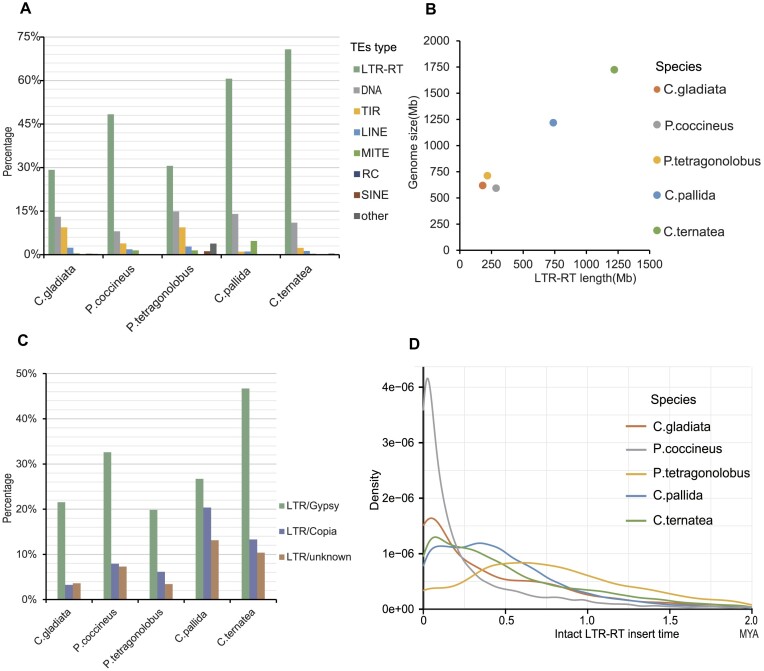
TEs in the 5 sequenced species in this study. (A) Distribution of various types of TEs in each species. (B) A scatterplot illustrating the correlation between the length of LTR-RT and the genome size. (C) Distribution of various types of LTR-RT in each species. (D) The insert time distribution of intact LTR-RT for each species. The sequence of LTR-RTs from intact LTR-RT identified by EDTA was obtained, and the long terminal repeats of each LTR-RT were aligned using MUSCLE. The APE package in R and the K80 model were used to estimate the pairwise distances from the LTR.

The highly continuous reference genomes enabled a comprehensive analysis of the transposable elements (TEs). In total, 55%, 63%, 64%, 82%, and 86% of the genomes are composed of TEs for sword bean, scarlet runner bean, winged bean, smooth rattlebox, and butterfly pea, respectively (Fig. 2A, Supplementary Table S12). Among all the TE types, long terminal repeat retrotransposon (LTR-RT), especially Gypsy-LRT, is the most dominant TE type for all the 5 species. Notably, LTR-RT activity is also the most contributing factor to the genome size (Fig. 2B, C, Supplementary Tables S13–S14), which is consistent with previous reports for most plants [31]. The LTR-RT expansion period in smooth rattlebox and butterfly pea is much wider than the other 3 species, which may partially explain their relatively larger genome sizes. Interestingly, there is a very recent sharp explosion of LTR-RT in scarlet runner bean, although its LTR-RT activity is much lower in the long history period. On the contrary, there is a high LTR-RT expansion in the old history period, but the LTR-RT activity gets lower and lower in the recent history period in winged bean (Fig. 2D). Taken together, these results suggest that the genome size of legume species has been changing in the evolution history along with the LTR-RT expansions and extractions.

### No recent whole-genome duplication was found in the 5 legumes

**Figure 3:**
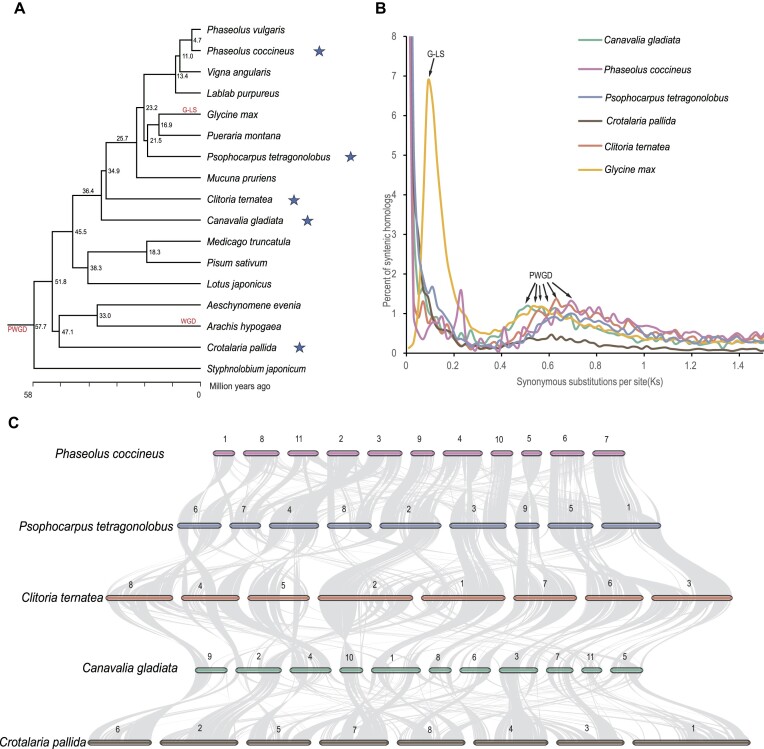
Evolution of Papilionoideae. (A) Phylogentic tree with divergence time estimated by the RelTime branch method in MEGA. Two calibration constraints were used: one was 8.0–19.5 million years ago between *Phaseolus vulgaris* and *Vigna angularis*, and the other was 47.7–56.0 million years ago between *Glycine max* and *Arachis hypogaea*. The 5 sequenced species in this study are marked with blue stars. The numbers on the side of the nodes represent divergence time values, and the whole-genome polyploidization events are indicated on the branches in red. (B) Homologous Ks distribution within species and paralogous gene pairs situated on collinear fragments containing over 5 syntenic gene pairs are employed for Ks calculation using the GMYN model in the KAKS_CALCULATOR. (C) Macro-synteny plots among the 5 studied species.

To study the evolution of Papilionoideae, the reference genes of sword bean, scarlet runner bean, winged bean, smooth rattlebox, butterfly pea, and 12 published Papilionoideae species, including *Phaseolus vulgaris* [32], *Vigna angularis* [23], *Lablab purpureus* [24], *Glycine max* [33], *Pueraria montana* [26], *Mucuna pruriens* [25], *Pisum sativum* [6], *Medicago truncatula* [34], *Lotus japonicus* [35], *Aeschynomene evenia* [36], *Arachis hypogaea* [22], and *Styphnolobium japonicum* [37] (Supplementary Tables S15–S16), were clustered into 35,057 orthologous groups (orthogroups), with each orthogroup containing at least 2 genes. *Vitis vinifera* [38] was used as an outgroup. Then, the 405 single-copy orthogroups were used for phylogeny construction and divergence time estimation (Supplementary Fig. S5). The winged bean, scarlet runner bean, butterfly pea, sword bean, and smooth rattlebox diverged from soybean (*G. max*) at 21.5 million years ago (MYA), 23.2 MYA, 34.9 MYA, 36.4 MYA, and 51.8 MYA, respectively (Fig. 3A).

To investigate the whole-genome duplication events, we calculated the synonymous mutation rate (Ks) values of the paralogue pairs for each species. The distribution of Ks values showed a shared peak at around 0.6 for all the 5 species in this study as well as soybean (Fig. 3B), consistent with previous reports that an ancient whole-genome duplication event (papilionoid-specific whole-genome duplication [PWGD]) occurred at the origin of the papilionoid clade 55 MYA [39]. The large amounts of whole genome–wide syntenic fragments inside each species also confirm this inference (Supplementary Fig. S6, Supplementary Table S17). Unlike soybean, which has a lineage-specific whole-genome duplication event (G-LS) 13 MYA corresponding to Ks peak around 0.1 [2], all the 5 species in this study do not have any recent Ks peaks, indicating that they do not have recent whole-genome duplication events. The gene expansion and contraction analyses along the phylogeny tree also showed no recent genome-wide gene burst on the branches for all 5 species in this study (Supplementary Fig. S7). Although the chromosome numbers have changed among the 5 species, many large syntenic blocks are still present, with multiple large-scale chromosome inversion and translocation events (Fig. 3C).

### Unique genes identified between *P. coccineus* and *P. vulgaris*

**Figure 4:**
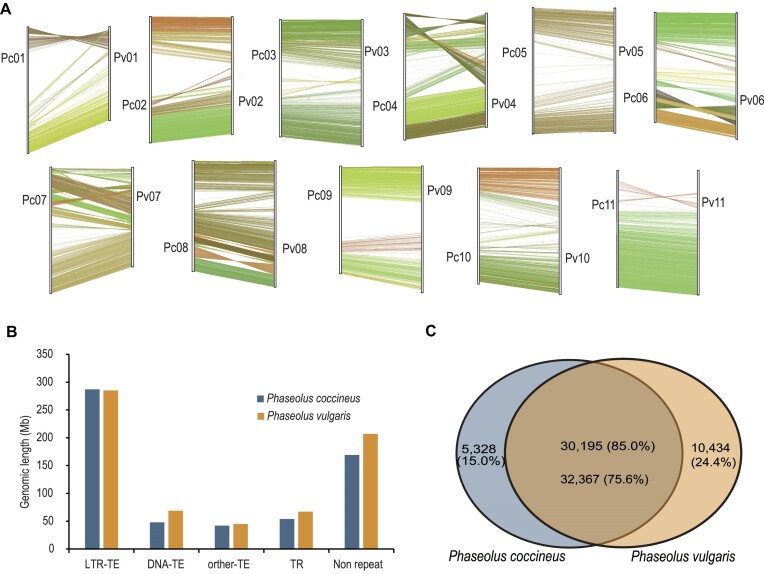
Genomic comparison between *Phaseolus coccineus* (scarlet runner bean) and *Phaseolus vulgaris* (common bean). (A) Macro-synteny blocks between *P. coccineus* and *P. vulgaris*. Collinear fragments containing over 20 syntenic gene pairs are utilized. Pc and Pv represent *P. coccineus* and *P. vulgaris*, respectively. (B) Distribution of LTR-TE, DNA-TE, other TE, TR, and nonrepeat regions in *P. coccineus* and *P. vulgaris*. (C) Overlap of the reference gene sets between *P. coccineus* and *P. vulgaris*. The protein sequences are aligned using DIAMOND with the parameters “–sensitive –evalue 1e-5.” Genes that remain unaligned are considered species-specific genes.

We performed comparative genomic studies between the 2 *Phaseolus* plants, scarlet runner bean (*P. coccineus*) and common bean (*P. vulgaris*) [7], which diverged from each other ∼4.7 MYA (Fig. 3A). Overall, all chromosomes from the 2 species have 1-versus-1 corresponding relationships, with only some intrachromosome inversions (Fig. 4A). The estimated genome sizes of the 2 species were both ∼590 Mb. Our assembly size of scarlet runner bean is 593 Mb, almost equal to the estimated genome size. Meanwhile, the latest assembly size of common bean (NCBI: GCA_029448765.1) is 615 Mb, which is a little larger than the estimated genome size. Looking into the TEs, we found that 63.5% of the scarlet runner bean genome is composed of TEs, a little lower than that of the common bean genome (65%) (Fig. 4B, Supplementary Table S18).

Furthermore, the 35,523 protein-coding genes of scarlet runner bean were compared to the 42,801 protein-coding genes of common bean, which was annotated by our pipeline in this study. Requiring an alignment E-value of 1e-5, 30,195 (85.0%) scarlet runner bean genes and 32,367 (75.6%) common bean genes were matched, leaving 5,328 (15.0%) unique genes in scarlet runner and 10,434 (24.4%) unique genes in common bean (Fig. 4C, Supplementary Table S18), which may be responsible for the phenotypic differences and species-specific functions between the 2 species.

### Expansion of *NIN* and *CHS* genes

Legume is special in plants for its nitrogen-fixing RNS [13], which requires a set of key genes (Supplementary Fig. S8A). The chalcone synthase (CHS) and isoflavone synthase (IFS) are responsible for the biosynthesis of isoflavone, which attracts Rhizobia. Nod factor receptor 1 (NFR1) and Nod factor receptor 5 (NFR5) perceive the Nod factors secreted from Rhizobia and interact with downstream symbiosis receptor-like kinase (SYMRK), which further activates 3-hydroxy-3-methylglutaryl-CoA reductase 1 (HMGR1), inducing nuclear calcium oscillations. Does not make infections 3 (DMI3) detects the calcium signal and activates IPD3/CYCLOPS and DELLA, which further induce downstream transcription factors nodulation signaling pathway 1 (NSP1), nodulation signaling pathway 2 (NSP2), nodule inception (NIN), NIN-like protein 2 (NLP2), and rhizobium-directed polar growth (RPG). Then, the *Lb* gene was activated to produce leghemoglobin [40]. We identified all these key genes in sword bean, scarlet runner bean, winged bean, smooth rattlebox, and butterfly pea (Supplementary Fig. S8B, Supplementary Tables S19–S20), providing a valuable gene resource for RNS studies.

In contrast to other symbiosis-relevant genes involved in infection, NIN and RPG are only known to have nitrogen-fixing nodulation (NFN) symbiosis–specific functions, whereas the mutation of other genes may have more pleiotropic effects. The phylogenomics studies also found that both the *NIN* and *RPG* genes exist in root nodulating legumes but are absent or have become pseudogenes in non-nodulating legumes, indicating that *NIN* and *RPG* are the essential genes for root nodulation [19]. In this study, we found that the *RPG* gene was single copy in each plant (Supplementary Fig. S9), but the *NIN* gene was duplicated in the early Papilionoideae ancestor, possibly as a result of the PWGD that occurred 55 MYA. One gene copy was retained in all 5 plants, but the other gene copy was missing in smooth rattlebox and butterfly pea (Supplementary Fig. S8C, Supplementary Table S21). Therefore, sword bean, scarlet runner bean, and winged bean all have 2 copies of the *NIN* genes, while smooth rattlebox and butterfly pea have only 1 copy of the *NIN* gene for each plant.

Isoflavones, a type of polyphenolic secondary metabolite from the phenylalanine pathway in plants with a C6–C3–C6 structure, are predominantly distributed in plants of the Papilionoideae subfamily and are mostly used as the signaling molecules between leguminous plants and rhizobia [41, 42]. In this study, we identified all the gene copies of *CHS* and *IFS* (Supplementary Fig. S8D, Supplementary Fig. S10, and Supplementary Table S22), which are the key genes responsible for isoflavone biosynthesis. Interestingly, scarlet runner bean and winged bean each have 11 copies of *CHS* genes, almost 2 times of that in the other 3 plants. From the phylogenetic analysis, we found that the expansion of *CHS* genes in scarlet runner bean and winged bean was species specific. Most of the duplicated *CHS* genes are located in a small cluster region on the same chromosome, indicating that the gene expansion occurred through local tandem gene duplications (Supplementary Fig. S8D). The difference in *CHS* gene number may influence the amount of isoflavone production in different species, which needs further investigations in future.

### Evolutionary history of resistance genes

Based on the type of N-terminal domain, the R genes were traditionally divided into 3 classes: TNL (TIR-NB-LRR), CNL (CC-NB-LRR), and RNL (RPW8-NB-LRR) (Supplementary Fig. S11A). In this study, we identified 7, 9, 5, 11, and 12 TNL genes and 13, 9, 7, 6, and 5 CNL genes for sword bean, scarlet runner bean, winged bean, smooth rattlebox, and butterfly pea, respectively (Supplementary Fig. S11B). In addition, only 1 R gene with the N-terminal RPW8 domain was identified in smooth rattlebox (Supplementary Fig. S12). Overall, scarlet runner bean has an equal number of TNL and CNL genes, sword bean and winged bean have more CNL genes than TNL genes, and smooth rattlebox and butterfly pea have more TNL genes than CNL genes. Through phylogenetic analysis, all the TNL and CNL genes were separated into 2 major branches (Supplementary Fig. S11C), consistent with previous studies that showed that the 3 classes of NLR R genes evolved at the early origin of ancestral Angiosperm [43]. Using *Albizia julibrissin* as an outgroup, which belongs to the second largest subfamily Caesalpinioideae in Fabaceae (Legume), we inferred 6 TNL orthologous groups (OGs) and 9 CNL OGs within the family Fabaceae, with each OG derived from a single gene in the common Fabaceae ancestor (Supplementary Fig. S11C). The 6 TNL and 9 CNL ancestral genes in Fabaceae derived from multiple whole-genome polyploidization events or gene duplications since the birth of Angiosperm. Considering that the 5 species in this study were much less domesticated than those well-cultivated legumes such as soybean, the 5 studied species here still have a much stronger ability of resisting disease. Therefore, the identified R genes in these 5 species may be transported to major legume crops such as soybean to improve their ability to resist disease.

## Discussion

In this study, we generated chromosome-level genome assemblies and high-quality gene annotations for 5 underutilized legume crops. The assembly quality is near telomere-to-telomere level, with only a few gaps in each constructed chromosome. Smooth rattlebox and butterfly pea have much larger genome sizes than sword bean, scarlet runner bean, and winged bean due to a relatively long period of LTR-RT expansion in the evolutionary history of these 2 species. Phylogeny and divergence time for the studied plants were inferred with the single-copy gene families, and a whole-genome duplication event at the origin of the papilionoid clade (PWGD) around 55 MYA was detected for all the studied species. Moreover, we identified 5,328 unique genes in scarlet runner bean and 10,434 unique genes in common bean, which may be helpful in investigating the functional genes underling their phenotypic differences. The genomic resources in this study expand the lineage coverage in published Fabaceae species, which will promote evolutionary and comparative genomics studies in Fabaceae.

In comparison to the grains, most beans have a much higher protein content, which is closely related with nitrogen-fixing root nodule symbiosis. Based on sequence homology, we identified all the key genes involved in root nodulation in the 5 studied plants. The *NIN* gene was duplicated in the early Papilionoideae ancestor, and then gene loss happened on 1 branch in smooth rattlebox and butterfly pea. We also found that the *CHS* genes were obviously expanded through local tandem duplication in scarlet runner bean and winged bean. Our results provide more genomic evidence for the evolution of nitrogen-fixing RNS, which will promote the molecular breeding of more efficient RNS cultivars and benefit the utilization of global nitrogen fixing by legume plants.

Improvement of disease resistance in crops has great potential to increase productivity. Huge losses caused by pathogenic fungi, bacteria, nematodes, oomycetes, and viruses could be mitigated by breeding of disease-resistant cultivars. The 5 underutilized legume crops in this study are much less human-selected than other well-known legume crops such as soybean and thus may include more powerful R genes. In this study, we identified all the R genes in the 5 studied plants, which can be divided mainly into 2 classes, TNL and CNL. Notably, sword bean and winged bean have more CNL genes, but smooth rattlebox and butterfly pea have more TNL genes. In the future, these R genes can be transferred into major legume crops such as soybean to improve their disease resistance, which will reduce the application of chemical pesticides and promote global food security.

## Methods

### Plant materials and sequencing

Commercial seed of sword bean (LVBAO cultivar) was obtained from Nongzhizi Seeds. Commercial seed of scarlet runner bean (Climbing cultivar) was obtained from Jinminxinnong. Commercial seed of winged bean (DUOXI-ginseng cultivar) was obtained from Bosite. Commercial seed of smooth rattlebox (3-ellipse-leaf cultivar) was obtained from the forestry bureau permitted seed store company. The young seedling of butterfly pea (blue-flower cultivar) was obtained from Liuyi flowers and fruit seedlings company. The seeds were grown in a plant growth chamber, and young leaves from a single plant for each species were used to extract genomic DNA using the Hi-DNAsecure Plant Kit (Tiangen DP350). The genomic DNA was used to prepare a 20-kb insert sequencing library by the SMRTbell Express Template Prep Kit 2.0 (PacBio), and the library was sequenced on the Sequel II platform in HiFi mode (PacBio). The young leaves from the same plant were also used for Hi-C sequencing on the Illumina NovaSeq 6000 platform in PE150 mode. The roots, stems, leaves, and flowers of each species were sampled to extract total RNA using the RNeasy Plant Mini Kit (Qiagen). The extracted RNA was pooled together for full-length cDNA sequencing on the PacBio Sequel II (RRID:SCR_017990) with the Iso-Seq mode (PacBio).

### Genome assembly and annotation

The contigs for sword bean, scarlet runner bean, and winged bean were assembled from PacBio HiFi reads utilizing HIFIASM version 0.16.1 (RRID:SCR_021069) [44] with parameter “-l 0,” and the contigs for smooth rattlebox and butterfly pea were assembled from PacBio HiFi reads utilizing HIFIASM version 0.19.5 [44] with parameter “-l 0.” Contamination was removed by aligning all contigs to chloroplast and mitochondria sequences downloaded from the NCBI database, using MINIMAP2 version 2.20 (RRID:SCR_018550) [45] with an identity >0.95 and coverage >0.95. The remaining contigs were used to represent the nuclear contigs, and the completeness was evaluated using BUSCO version 5.1.2 (RRID:SCR_015008) [46] with ORTHODB version 10 (embryophyta lineage). Ultimately, the Hi-C reads were aligned to the nuclear contigs, and Hi-C contact matrices among the contig bins were generated utilizing HIC-PRO version 3.1.0 (RRID:SCR_017643) [47]. Utilizing the Hi-C linkage information between contig ends, nuclear contigs with sizes exceeding 1 Mb were assembled into scaffolds at the chromosome level utilizing ENDHIC version 1.0 (RRID:SCR_022110) [48].

TR elements were detected using Tandem Repeats Finder (TRF) version 4.09 [49]. Interspersed repeat elements (TEs) were identified through a 3-step process: (i) the prediction of structurally intact TEs, including LTR-RTs, DNA transposon, Helitron, and so on, was accomplished using EDTA version 1.9.9 [50]. Concurrently, an intact TE library was generated. (ii) Incomplete and homology TEs were detected against the abovementioned intact TE library, Repbase database version 26.05 (plant lineage), and the protein-coding TE database using REPEATMASKER version 4.1.2 (RRID:SCR_012954). (iii) A *de novo* TE library was generated from the masked genome with all the abovementioned identified TEs, using REPEATMODELER version 2.0.1 (RRID:SCR_015027), and then the TEs in the library were classified using TERL v1.0 (RRID:SCR_022064) [51]. The TEs that were assigned known TE types were then used by REPEATMASKER to identify species-specific TEs in the genome. Ultimately, merging the overlapping coordinates and removing any redundancy was used to produce a nonredundant TE annotation. All TEs larger than 80 bp in size for the scarlet runner bean and the winged bean, as well as those larger than 200 bp in size for the sword bean, the smooth rattlebox, and the butterfly pea, were soft-masked (uppercase to lowercase) on their genome sequences for gene prediction.

Transcript and homology hints were used to predict protein-coding gene models by AUGUSTUS version 3.4.0 (RRID:SCR_008417) [52]. The AUGUSTUS parameters of gene prediction were generated from the intermediate outcomes of the BUSCO assessment of genome assembly. Full-length transcripts generated by PacBio Iso-Seq were aligned to the genome using GMAP version 2019-12-01 (RRID:SCR_008992) [53] and AUGUSTUS filter script with parameters “–minId=95 –minCover=95.” To obtain homology hints, the proteome of 12 representative Papilionoideae species (*P. vulgaris* [32], *V. angularis* [23], *L. purpureus* [24], *G. max* [33], *P. montana* [26], *M. pruriens* [25], *P. sativum* [6], *M. truncatula* [34], *L. japonicus* [35], *A. evenia* [36], *A. hypogaea* [22], *S. japonicum* [37]) was aligned to the genome using EXONERATE version 2.4.0 (RRID:SCR_016088) [54]. The homology and transcript alignment results were transformed into hints files to support gene prediction of AUGUSTUS. To further filter the TE-contaminated genes, the genes whose coordinates overlapped more than 99% with the annotated TE were removed from the gene sets. BUSCO version 5.1.2 [46] was used to evaluate the completeness of the gene sets.

For the annotation of gene function, protein sequences were aligned to KEGG and NCBI-NR databases using the DIAMOND version 0.8.2 (RRID:SCR_009457) [55] with parameter “E-value 1E−5,” and the best hits were kept. INTERPROSCAN version 5.52–86 (RRID:SCR_005829) [56] with database searching of CDD-3.18, Coils-2.2.1, Gene3D-4.3.0, Hamap-2020_05, MobiDBLite-2.0, PANTHER-15.0, Pfam-33.1, PIRSF-3.10, PIRSR-2021_02, PRINTS-42.0, ProSitePatterns-2021_01, ProSiteProfiles-2021_01, SFLD-4, SMART-7.1, SUPERFAMILY-1.75, and TIGRFAM-15.0 was used to detect the protein domain and obtain related GO terms. The 8S, 18S, and 28S rRNAs were identified utilizing RNAMMER version 1.2 (RRID:SCR_017075) [57], and the tRNAs were identified utilizing TRNASCAN-SE version 2.0 (RRID:SCR_008637) [58].

### Phylogeny and polyploidization analysis

To construct the OGs, we used ORTHOFINDER version 2.5.2 (RRID:SCR_017118) [59] with parameters “-M msa -A mafft -T fasttree -1 -y,” with 12 Papilionoideae species (*P. vulgaris* [32], *V. angularis* [23], *L. purpureus* [24], *G. max* [33], *P. montana* [26], *M. pruriens* [25], *P. sativum* [6], *M. truncatula* [34], *L. japonicus* [35], *A. evenia* [36], *A. hypogaea* [22], *S. japonicum* [37]) and 1 outgroup species, *V. vinifera* [38].

From the orthogroups of ORTHOFINDER, the OG with *A. hypogaea* (recent WGD) [22] having 1 or 2 copies and other species having only 1 copy were selected, and then a gene of *A. hypogaea* from duplicated genes was randomly thrown. Subsequently, all single-copy genes of all species were used to employ multiple sequence alignment (MSA) utilizing MUSCLE version 3.8.31 (RRID:SCR_011812) [60], and these MSAs were combined to create a concatenated multiple sequence alignment (CMSA). Next, the CMSA was used to build a species tree utilizing RAXML version 1.0.3 (RRID:SCR_006086) [61] with parameters “–model GTR+G –tree pars –bs-trees 100 –outgroup Vitis_vinifera.” To estimate the divergence time, we used the RelTime branch method in MEGA11 (RRID:SCR_000667) [62] with 1 calibration time 8.0 to 19.5 million years ago between *P. vulgaris* and *V. angularis* and the other calibration time 47.7 to 56.0 million years ago between *G. max* and *A. hypogaea*. The 2 calibration times were obtained from TimeTree (RRID:SCR_021162). Subsequently, the expansion and contraction of gene families was inferred using CAFE version 5 (RRID:SCR_005983) [63] with the parameter “-k5.”

MCSCANX (RRID:SCR_022067) [64] was used to identify collinear gene blocks with more than 5 collinear genes. Synteny figures of the whole genome were plotted using the Java programs dual_synteny_plotter and dot_plotter from the MCSCANX package. According to the result of collinear genes, KAKS_CALCULATOR version 2.0 [65] with GMYN model was employed to calculate the synonymous substitution rate (Ks) value for syntenic gene pairs. Chromosome collinearity among species was drawn using JCVI (RRID:SCR_021641) with parameter “–cscore=.99.”

### Analysis of genes involved in nitrogen-fixing root nodulation

The protein sequences associated with nitrogen-fixing nodulation were obtained from NCBI and Phytozome, and they were aligned to the reference gene sets of the 5 studied species using the blastp algorithm in DIAMOND version 0.8.28 [55], with the parameters “–more-sensitive –evalue 0.00001.” The alignment results were further refined with a 50% identity and 60% coverage threshold. In this way, we identified the potential genes involved in nitrogen-fixing root nodulation.

To analyze the phylogenetic relationships, the protein sequences within each gene family of *NIN, RPG, CHS*, and *IFS* were aligned independently using the MUSCLE version 3.8.31 [60]. Subsequently, phylogenetic trees were constructed utilizing the FastTree version 2.1.11 (RRID:SCR_015501) [66]. *V. vinifera* was used as an outgroup. The phylogenetic trees for *NIN* and *RPG* genes were visualized using FigTree version 1.4.4 (RRID:SCR_008515), and those for *CHS* and *IFS* genes were displayed using iTol (RRID:SCR_018174).

### Analysis of R genes

The protein sequences of all genes from the 5 studied plants and *A. julibrissin* (NCBI: PRJNA1005079) were searched against the HMM profile of all domains using the hmmsearch program in HMMER version 3.1b2 [67] with the parameters “-E 1e-5 –domE 1e-5.” Genes with at least 1 of the identified domains, including TIR (PF01582), TIR_2 (PF13676), RPW8 (PF05659), NB-ARC (PF00931), LRR_1 (PF00560), LRR_2 (PF07723), LRR_3 (PF07725), LRR_4 (PF12799), LRR_5 (PF13306), LRR_6 (PF13516), LRR_8 (PF13855), and LRR_9 (PF14580), were chosen as the primary potential R genes. In addition, CC domains were further annotated using the Coils database from INTERPROSCAN version 5.52–86 [56]. The genes with TIR-NB-LRR, CC-NB-LRR, and RPW8-NB-LRR domain structures were classified as TNL, CNL, and RNL R genes, respectively. The alignment of protein sequences for CNL and TNL R genes was performed using MUSCLE version 3.8.31 [60]. Subsequently, a phylogenetic tree was constructed by FastTree version 2.1.11 [66], which was displayed using iTOL.

## Supplementary Material

giae063_GIGA-D-24-00031_Original_Submission

giae063_GIGA-D-24-00031_Revision_1

giae063_GIGA-D-24-00031_Revision_2

giae063_Response_to_Reviewer_Comments_Original_Submission

giae063_Response_to_Reviewer_Comments_Revision_1

giae063_Reviewer_1_Report_Original_Submission Shangang Jia -- 3/1/2024 Reviewed

giae063_Reviewer_1_Report_Revision_1 Shangang Jia -- 4/23/2024 Reviewed

giae063_Reviewer_2_Report_Original_Submission Paulo Izquierdo, Ph.D. -- 3/3/2024 Reviewed

giae063_Reviewer_2_Report_Revision_1 Paulo Izquierdo, Ph.D. -- 4/26/2024 Reviewed

giae063_Reviewer_3_Report_Original_Submission Vanika Garg -- 3/9/2024 Reviewed

giae063_Reviewer_3_Report_Revision_1 Vanika Garg -- 4/25/2024 Reviewed

giae063_Supplemental_File

## Data Availability

The genomic and transcriptomic sequencing reads generated in this study have been deposited in SRA of NCBI under accession numbers PRJNA1001638, PRJNA1002813, PRJNA1003673, PRJNA1014360, and PRJNA1016062 for *Canavalia gladiata, Phaseolus coccineus, Psophocarpus tetragonolobus, Crotalaria pallida*, and *Clitoria ternatea*, respectively. The genome assemblies and gene annotations have been deposited at GenBank of NCBI under accession numbers JAYMYQ000000000, JAYMYR000000000, JAYMYS000000000, JAYWIO000000000, and JAYKXN000000000. All additional supporting data for *Canavalia gladiata, Phaseolus coccineus, Psophocarpus tetragonolobus, Crotalaria pallida*, and *Clitoria ternatea* are available in the *GigaScience* repository, GigaDB [68–73].

## Additional Files

**Supplementary Fig. S1**. Distribution of *k*-mer (*k* = 17) frequency in sequencing reads of the 5 Papilionoideae plants: (a) *Canavalia gladiata*, (b) *Phaseolus coccineus*, (c) *Psophocarpus tetragonolobus*, (d) *Crotalaria pallida*, and (e) *Clitoria ternatea*. The used genomic sequencing data are from PacBio-HiFi technology. The *k*-mer frequency peak with an arrow is the main peak. The *k*-mer distribution indicates that the 5 Papilionoideae plants are homozygous.

**Supplementary Fig. S2**. Hi-C heatmap of chromosome assembly for 5 Papilionoideae plants. The color represents Log2(Links number), and the resolution (bin size) is 500 Kb. The links number represents the count of Hi-C links that fall within the 2 analyzed genomic bins.

**Supplementary Fig. S3**. Bandage view of EndHiC scaffold results and the position of telomere for 5 Papilionoideae plants: (a) *Canavalia gladiata*, (b) *Phaseolus coccineus*, (c) *Psophocarpus tetragonolobus*, (d) *Crotalaria pallida*, and (e) *Clitoria ternatea*. The chromosome ends, assembled with telomere-specific tandem repeats (unit: TTTAGGG), were marked with a red circle.

**Supplementary Fig. S4**. Chromosomal collinear comparison between Ma3 and DUOXI-ginseng before and after correction. The parameter for Minimap version 2.20 is set to “x asm5.”

**Supplementary Fig. S5**. Species tree of sequenced Papilionoideae species. The tree was built by RAxML using concatenated cds sequences extracted from 405 near-single-copy orthogroups, in which all species have 1 gene copy except for *Arachis hypogaea*, which possesses 2 gene copies. One of the 2 gene copies in *A. hypogaea* was selected randomly. *Vitis vinifera* (grape) were employed as outgroups in this context. The bar represents substitutions per nucleotide site.

**Supplementary Fig. S6**. Dot intraspecies synteny supplemental figures for *C. gladiata, P. coccineus, P. tetragonolobus, C. pallida*, and *C. ternatea*. The java program “dot_plotter” from the MCScanX package was used to draw colinear blocks with more than 10 syntenic gene pairs.

**Supplementary Fig. S7**. Analysis of gene family (orthogroup) expansion and contraction was conducted using CAFÉ. The numbers of expanded (“+”) and contracted (“−”) families on each branch are indicated on the phylogenetic timeline tree. The analysis utilized 31,643 OrthoFinder orthogroups (with a gene count of ≤200 and a species count of ≥2 and excluding families not present at the root) as input.

**Supplementary Fig. S8**. Root nodulation symbiosis. (A) Pathway of crucial genes involved in symbiotic nodulation and nitrogen fixation for Papilionoideae. The model figure is drawn using Figdraw. (B) The gene number of the symbiotic pathway identified in *C. gladiata, P. coccineus, P. tetragonolobus, C. pallida*, and *C. ternatea*. (C) Gene tree for *NIN*. Members of the gene family were obtained from OrthoFinder orthogroups, and the gene tree was constructed by FastTree. Bootstrap values are shown on each branch, and the 2 duplicated branches are highlighted. *V. vinifera* is utilized as the outgroup. (D) Gene tree for *CHS*, with similar style to *NIN*. The genes from the 5 sequenced species are shown in 5 different colors, and the tandem replicated genes of different species have been highlighted using distinct background colors.

**Supplementary Fig. S9**. Phylogenetic tree analysis of *RPG* genes. A phylogenetic tree for the *RPG* genes was constructed using FastTree with protein sequences. All these genes belong to an orthogroup, and bootstrap values are indicated on each branch. The diagram involves 6 species, including *Canavalia gladiata* (*Cgla*), *Phaseolus coccineus* (*Pcoc*), *Psophocarpus tetragonolobus* (*Ptet*), *Crotalaria pallida* (*Cpal*), *Clitoria ternatea* (*Cter*), and *Vitis vinifera* (*Vvin*). *V. vinifera* (*Vvin*) is utilized as the outgroup.

**Supplementary Fig. S10**. Phylogenetic tree analysis of *IFS* genes. A phylogenetic tree for the *IFS* genes was constructed using FastTree with protein sequences. All these genes belong to an orthogroup, and bootstrap values are indicated on each branch. The diagram involves 5 species, including *Canavalia gladiata* (*Cgla*), *Phaseolus coccineus* (*Pcoc*), *Psophocarpus tetragonolobus* (*Ptet*), *Crotalaria pallida* (*Cpal*), and *Clitoria ternatea* (*Cter*).

**Supplementary Fig. S11**. Resistance (R) genes. (A) Model figure for 3 types of R genes: TNL, CNL, and RNL. TNL consists of TIR, NB-ARC, and LRR domains; CNL consists of CC, NB-ARC, and LRR domains; and RNL consists of RPW8, NB-ARC, and LRR domains from N-terminal to C-terminal. (B) The number of CNL-type and TNL-type R genes in *C. gladiata, P. coccineus, P. tetragonolobus, C. pallida, C. ternatea*, and *A. julibrissin* species, respectively. (C) The phylogenetic tree of all identified R genes in the 5 studied species and 1 outgroup (*A. julibrissin*). The 2 major branches, TNL and CNL, are labeled with different background colors; a solid circle on the clade represents an OG; and the outgroup is marked with a gray graph. The R genes from various species are differentiated by various symbols.

**Supplementary Fig. S12**. The position of domains on the protein sequences of TNL-type, CNL-type, and RNL-type R genes. The diagram involves 6 species, including *Canavalia gladiata* (*Cgla*), *Phaseolus coccineus* (*Pcoc*), *Psophocarpus tetragonolobus* (*Ptet*), *Crotalaria pallida* (*Cpal*), *Clitoria ternatea* (*Cter*), and *Albizia julibrissin* (*Ajul*). The graphical display was generated using TBtools.

**Supplementary Table S1**. Statistics of genome sequencing statistics.

**Supplementary Table S2**. Statistics of Hi-C data mapping to contigs for *C. gladiata, P. coccineus*, and *P. tetragonolobus*.

**Supplementary Table S3**. Statistics of Hi-C data mapping to contigs for *C. pallida* and *C. ternatea*.

**Supplementary Table S4**. Statistics of reference organelle genomes used for filtering sequence from the organelle.

**Supplementary Table S5**. Statistics of genome assembly.

**Supplementary Table S6**. Statistics of tandem repeats annotation.

**Supplementary Table S7**. Statistics of full-length cDNA mapping.

**Supplementary Table S8**. Statistics of Exonerate homology predictions.

**Supplementary Table S9**. Statistics of predicted gene models by Augustus.

**Supplementary Table S10**. Statistics of gene function assignment.

**Supplementary Table S11**. Statistics of annotated noncoding RNA genes.

**Supplementary Table S12**. Statistics of TE annotations.

**Supplementary Table S13**. Statistics of TE content in various classes.

**Supplementary Table S14**. Statistics of TE content in various superfamilies.

**Supplementary Table S15**. Public genome data information.

**Supplementary Table S16**. Public gene data information.

**Supplementary Table S17**. Summary of duplicated genes.

**Supplementary Table S18**. Features between *Phaseolus coccineus* and *Phaseolus vulgaris*.

**Supplementary Table S19**. Reference gene protein sequence information of nitrogen-fixing root nodulation.

**Supplementary Table S20**. Genes involved in nitrogen-fixing root nodulation.

**Supplementary Table S21**. The Pfam domain annotation of *NIN* genes.

**Supplementary Table S22**. The Pfam domain annotation of *CHS* genes.

## Abbreviations

BUSCO: Benchmarking Universal Single-Copy Orthologs; CC: coiled-coil; CHS: chalcone synthase; CMSA: concatenated multiple sequence alignment; DMI3: does not make infections 3; ETS: effector-triggered susceptibility; GO: Gene Ontology; HMGR1: 3-hydroxy-3-methylglutaryl-CoA reductase 1; HR: hypersensitive response; IFS: isoflavone synthase; KEGG: Kyoto Encyclopedia of Genes and Genomes; Lb: leghemoglobin; LRR: leucine-rich repeat domain; LTR-RT: long terminal repeat retrotransposon; MSA: multiple sequence alignment; MYA: million years ago; NCBI: National Center for Biotechnology Information; NFN: nitrogen-fixing nodulation; NFR1: Nod factor receptor 1; NFR5: Nod factor receptor 5; NIN: nodule inception; NLP2: NIN-like protein 2; NSP1: nodulation signaling pathway 1; NSP2: nodulation signaling pathway 2; OG: orthologous group; PWGD: papilionoid-specific whole-genome duplication; QV: quality value; R: resistance; RNS: root-nodule symbiosis; RPG: rhizobium-directed polar growth; rRNA: ribosomal RNA; SYMRK: symbiosis receptor-like kinase; TE: transposable element; TIR: Toll/interleukin 1 receptor; TR: tandem repeats; tRNA: transfer RNA.
