# Supplementary material for: The genomes of 5 underutilized Papilionoideae crops provide insights into root nodulation and disease resistance
Source: Gigascience. 2024 Aug 27;13:giae063. doi: 10.1093/gigascience/giae063 (PMC11348429; doi:10.1093/gigascience/giae063)
Supplement: giae063_GIGA-D-24-00031_Original_Submission [file giae063_giga-d-24-00031_original_submission.pdf]

GigaScience

The genomes of five underutilized Papilionoideae crops provide insights into root nodulation and disease resistance

--Manuscript Draft--

**Manuscript Number:**	GIGA-D-24-00031
**Full Title:**	The genomes of five underutilized Papilionoideae crops provide insights into root nodulation and disease resistance
**Article Type:**	Research
**Funding Information:**	Shenzhen Key Laboratory Fund (ZDSYS20141118170111640) — Not applicable<br>Shenzhen Science and Technology Innovation Program (JCYJ20190814163805604, KQTD20180411143628272) — Not applicable
**Abstract:**	Background<br><br>The Papilionoideae subfamily contains a large amount of underutilized legume crops, which are important for food security and human sustainability. However, the lack of genomic resources has hindered the breeding and utilization of these crops.<br><br>Results<br><br>Here, we present chromosome-level reference genomes for five underutilized Papilionoideae crops: sword bean, scarlet runner bean, winged bean, smooth rattlebox and butterfly pea, with assembled genome sizes of 0.62, 0.59, 0.71, 1.22, 1.72 Gb, respectively. We found that long period of higher LTR activity is the major reason that enlarges the genome size of smooth rattlebox and butterfly pea, and there is no recent whole duplication (WGD) event in these 5 species except for the shared papilionoid-specific WGD event (PWGD, ~55 Mya). Then, we identified 6,710 and 669 unique genes between scarlet runner bean and common bean for each species, which may be responsible for their phenotypic differences and species-specific functions. Furthermore, we identified the key genes involved in root nodule symbiosis in all 5 species, and found that the NIN gene was duplicated in the early Papilionoideae ancestor, followed by the loss of one gene copy in smooth rattlebox and butterfly pea. At last, we identified the resistance (R) genes for plant defenses in these 5 species, and characterized their evolutionary history.<br><br>Conclusions<br><br>In summary, this study provides chromosome-scale reference genomes for three grain and vegetable beans (sword bean, scarlet runner bean, winged bean), along with genomes for a green manure crop (smooth rattlebox) and a food dyeing crop (butterfly pea). These genomes are crucial for studying phylogenetic history, unraveling nitrogen-fixing nodule symbiosis (RNS) evolution, and advancing plant defense research.
**Corresponding Author:**	Wei Fan<br>Chinese Academy of Agricultural Sciences<br>shenzhen, guangdong CHINA
**Corresponding Author Secondary Information:**	
**Corresponding Author's Institution:**	Chinese Academy of Agricultural Sciences
**Corresponding Author's Secondary Institution:**	
**First Author:**	Lihua Yuan
**First Author Secondary Information:**	

Powered by Editorial Manager® and ProduXion Manager® from Aries Systems Corporation

Order of Authors:	Lihua Yuan
	Lihong Lei
	Fan Jiang
	Anqi Wang
	Rong Chen
	Hengchao Wang
	Sihan Meng
	Wei Fan
Order of Authors Secondary Information:	
Additional Information:	
**Question**	**Response**
Are you submitting this manuscript to a special series or article collection?	No
**Experimental design and statistics**<br><br>Full details of the experimental design and statistical methods used should be given in the Methods section, as detailed in our Minimum Standards Reporting Checklist. Information essential to interpreting the data presented should be made available in the figure legends.<br><br>Have you included all the information requested in your manuscript?	Yes
**Resources**<br><br>A description of all resources used, including antibodies, cell lines, animals and software tools, with enough information to allow them to be uniquely identified, should be included in the Methods section. Authors are strongly encouraged to cite Research Resource Identifiers (RRIDs) for antibodies, model organisms and tools, where possible.<br><br>Have you included the information requested as detailed in our Minimum Standards Reporting Checklist?	Yes
**Availability of data and materials**	Yes

All datasets and code on which the conclusions of the paper rely must be either included in your submission or deposited in publicly available repositories (where available and ethically appropriate), referencing such data using a unique identifier in the references and in the "Availability of Data and Materials" section of your manuscript.<br><br>Have you have met the above requirement as detailed in our Minimum Standards Reporting Checklist?	

Click here to access/download;Manuscript;manuscript.docx

Title

The genomes of five underutilized Papilionoideae crops provide insights into root nodulation and disease resistance

Lihua Yuan[1,2,3,4], Lihong Lei[1,2,3,4], Fan Jiang[1,4], Anqi Wang[1], Rong Chen[1], Hengchao Wang[1], Sihan Meng[1], Wei Fan[1,*]

[1]Guangdong Laboratory for Lingnan Modern Agriculture (Shenzhen Branch), Genome Analysis Laboratory of the Ministry of Agriculture and Rural Affairs, Agricultural Genomics Institute at Shenzhen, Chinese Academy of Agricultural Sciences, Shenzhen, Guangdong, 518120, China

[2]State Key Laboratory of Crop Stress Adaptation and Improvement, School of Life Sciences, Henan University, Kaifeng 475004, China

[3]Shenzhen Research Institute of Henan University, Shenzhen 518000, China

[4]These authors contributed equally to this article.

*Correspondence author: Wei Fan (fanwei@caas.cn)

Abstract

Background: The Papilionoideae subfamily contains a large amount of underutilized legume crops, which are important for food security and human sustainability. However, the lack of genomic resources has hindered the breeding and utilization of these crops.

Results: Here, we present chromosome-level reference genomes for five underutilized Papilionoideae crops: sword bean, scarlet runner bean, winged bean, smooth rattlebox and butterfly pea, with assembled genome sizes of 0.62, 0.59, 0.71, 1.22, 1.72 Gb, respectively. We found that long period of higher LTR activity is the major reason that enlarges the genome size of smooth rattlebox and butterfly pea, and there is no recent whole duplication (WGD) event in these 5 species except for the shared papilionoid-specific WGD event (PWGD, ~55 Mya). Then, we identified 6,710 and 669 unique genes between scarlet runner bean and common bean for each species, which may be responsible for their phenotypic differences and species-specific functions. Furthermore, we identified the key genes involved in root nodule symbiosis in all 5 species, and found that the *NIN* gene was duplicated in the early Papilionoideae ancestor, followed by the loss of one gene copy in smooth rattlebox and butterfly pea. At last, we identified the resistance (R) genes for plant defenses in these 5 species, and characterized their evolutionary history.

Conclusions: In summary, this study provides chromosome-scale reference genomes for three grain and vegetable beans (sword bean, scarlet runner bean, winged bean), along with genomes for a green manure crop (smooth rattlebox) and a food dyeing crop (butterfly pea). These genomes are crucial for studying phylogenetic history, unraveling nitrogen-fixing nodule symbiosis (RNS) evolution, and advancing plant defense research.

Key words: Papilionoideae, underutilized legume, whole genome duplication, root nodule symbiosis, R genes

Introduction

Papilionoideae, the largest subfamily in Fabaceae (Legume) [1]whose name probably originated from its flower's resemblance to a butterfly (Latin: Papilio), has an extremely important position in agriculture and makes great contributions to the human diet and food security. Besides, the several well-known crops such as soybean [2, 3], peanut [2, 3], faba bean [4, 5], mung bean [4, 5], pea [6, 7], common bean [6, 7] and alfalfa [8], this subfamily also includes many other underutilized crops [9]. For example, sword bean (*Canavalia gladiata*), scarlet runner bean (*Phaseolus coccineus*) and winged bean (*Psophocarpus tetragonolobus*) are both grain and vegetable plants: the mature bean seeds are protein-rich grains, while the young bean pods are delicious vegetables. Smooth rattlebox (*Crotalaria pallida*) and butterfly pea (*Clitoria ternatea*) are often used as green manure and forage grass, due to their high protein content. The dried flowers of butterfly pea are also used as a natural food colorant (blue), which is popular in Southeast Aisa countries [10]. In addition, sword bean has also been used as a traditional medicine to improve poor appetite and alleviate vomiting in China for thousands of years [11], and scientists found that smooth rattlebox also has antitumor properties in recent years [12].

The Papilionoideae plants also play a unique ecological role in nitrogen fixation through symbiotic root nodules [13], which is indispensable for the global nitrogen cycle. The ability to nitrogen fixation from the atmosphere also helps agriculture production use fewer synthetic

fertilizers, thereby reducing the energy consumption and mitigating soil pollution [14]. As a model of special host-bacteria interaction, the formation of nitrogen-fixing root nodule has been intensively studied on two model species *Medicago truncatula* and *Lotus japonicus* [15, 16], both belong to subfamily Papilionoideae. The host plants excrete flavonoids into the rhizosphere, and induce the rhizobia to express the nodulation (nod) genes [13]. Then, the metabolite products of these nod genes (Nod factors) are sensed by the host plants to start nodulation, which requires the coordination of rhizobial infection at the root epidermis with cell division in the cortex [17]. Inside the nodule, rhizobia live in organelle-like structure known as symbiosome, and the host plants secrete leghemoglobin (Lb) to maintain a low-oxygen environment in order to facilitate the nitrogen-fixing reactions in symbiosomes [18]. Recent phylogenomics and phylotranscriptomics studies have shown a single origin of nitrogen-fixing root nodule symbiosis (RNS), and then multiple independent losses occurred in various lineages [19, 20].

For the purpose of nodulation studies and crop breeding, tens of agricultural important plants in Papilionoideae have been sequenced, including all the above well-known species, as well as adzuki bean [21], lablab bean [22], velvet bean [23], kudzu vine [24], and pagoda tree [25], et al.. However, the subfamily contains many other rare but valuable species, which still lack reference genomes, hindering the in-depth biological studies and exploitation of these species. Here, we present the chromosome-scale reference genomes for 3 grain and vegetable beans (sword bean, scarlet runner bean, winged bean), a green manure crop (smooth rattlebox), and a food dyeing crop (butterfly pea) to investigate the phylogenetic history, explore the evolution of RNS, and identify the resistance (R) genes involved in controlling crop diseases.

Results

Chromosome-scale assembly of 5 underutilized legumes

We generated 67 Gb, 70 Gb, 64 Gb, 69 Gb, and 167 Gb HiFi data for sword bean, scarlet runner bean, winged bean, smooth rattlebox, and butterfly pea, respectively (Supplementary Table S1). Analyzing the distribution of K-mer frequencies [26], we found that all the 5 sequencing materials are highly homozygous and the estimated genome size is 0.65 Gb, 0.59 Gb, 0.69 Gb, 1.33 Gb, and 1.76 Gb for each species (Supplementary Fig. S1). Then, the HiFi data were assembled into large contigs, which were further linked into 11, 11, 9, 8, 8 chromosome-scale scaffolds by Hi-C data (Supplementary Table S2-S4, Supplementary Fig. S2). Overall, most chromosomes include less than 5 contigs, suggesting a very high continuity of our assembly (Supplementary Fig. S3). Besides, the BUSCO (Benchmarking Universal Single-Copy Orthologs) complete ratio is over 99% and the QV value calculated by Merqury version 1.3 [27] is over 70 for all the 5 species, indicating the very high accuracy of our assembly (Table 1, Supplementary Table S5-S6).

In total, 51,158, 35,523, 40,081, 48,759 and 40,267 protein-coding gene models were predicted in the genome of sword bean, scarlet runner bean, winged bean, smooth rattlebox, and butterfly pea, respectively (Figure 1, Supplementary Table S7-S9). The coding regions cover 51 Mb (8.2%), 42 Mb (7.1%), 44 Mb (7.4%), 51 Mb (4.2%), and 42 Mb (2.4%) of the genome for each species. The BUSCO complete rates for the gene sets of these 5 species are comparable to those BUSCO complete rates for the genomes, suggesting a high completeness of our gene annotation (Table 1). For function annotation, 72.1%, 89.0%, 83.9%, 87.7% and 86.8% of genes in

the 5 species were annotated by at least one of the NCBI-NR, KEGG, InterPro or GO databases (Supplementary Table S10). In addition, we identified 970, 1,141, 1,283, 1,382 and 2,307 tRNA genes, and 1,535, 5,030, 3,020, 6,268 and 3,158 rRNA genes for the five species (Supplementary Table S11).

LTR activity influences the genome size

The highly continuous reference genomes enabled a comprehensive analysis of the transposable elements (TEs). In total, 55%, 63%, 64%, 82% and 86% of the genomes are composed of TEs for sword bean, scarlet runner bean, winged bean, smooth rattlebox, and butterfly pea, respectively (Figure 2A, Supplementary Table S12). Among all the TE types, LTR especially Gypsy-LRT is the most dominant TE type for all the 5 species. Notably, LTR activity is also the most contributing factor to the genome size (Figure 2B-2C, Supplementary Table S13-S14), which is consistent with previous reports for most plants [28]. The LTR expansion period in smooth rattlebox and butterfly pea are much wider than the other 3 species, which may partially explain their relatively larger genome sizes. Interestingly, there is a very recent sharp explosion of LTR in scarlet runner bean, though its LTR activity is much lower in the long history period. On the contrary, there is a high LTR expansion in the old history period, but the LTR activity gets lower and lower in the recent history period in winged bean (Figure 2D). Taken together, these results suggest that the genome size of legume species have been changing in the evolution history along with the LTR expansions and extractions.

No recent whole genome duplication was found in the five legumes

To study the evolution of Papilionoideae, the reference genes of sword bean, scarlet runner bean, winged bean, smooth rattlebox, butterfly pea, and 12 published Papilionoideae species, including *Phaseolus vulgaris* [7], *Vigna angularis* [21], *Lablab purpureus* [22], *Glycine max* [29], *Pueraria montana* [24], *Mucuna pruriens* [23], *Pisum sativum* [30], *Medicago truncatula* [31], *Lotus japonicus* [32], *Aeschynomene eveni* [33], *Arachis hypogaea* [34] and *Styphnolobium japonicum* [35] (Supplementary Table S15-S16), were clustered into 35,057 orthologous groups (orthogroups), with each orthogroup containing at least two genes. *Vitis vinifera* [36] was used as an outgroup. Then, the 405 single-copy orthogroups were used for phylogeny construction and divergence time estimation (Supplementary Fig. S4). The winged bean, scarlet runner bean, butterfly pea, sword bean and smooth rattlebox diverged from soybean (*Glycine max*) at 21.5 Mya, 23.2 Mya, 34.9 Mya, 36.4 Mya and 51.8 Mya, respectively (Figure 3A).

To investigate the whole genome duplication events, we calculated the Ks values of the paralogue pairs for each species. The distribution of Ks values showed a shared peak at around 0.6 for all the 5 species in this study as well as soybean (Figure 3B), consistent with previous reports that an ancient whole genome duplication event (PWGD) occurred at the origin of the papilionoid clade 55 Mya [37]. The large amounts of whole genome-wide syntenic fragments inside each species also confirms this inference (Supplementary Fig. S5-6, Supplementary Table S17). Unlike soybean which has a lineage-specific whole genome duplication event (G-LS) 13 Mya corresponding to Ks peak around 0.1 [2], all the 5 species in this study do not have any recent whole genome duplication events. Although the chromosome numbers have changed among the 5 species, many large syntenic blocks were still existent, but with multiple large-scale chromosome

inversion and translocation events (Figure 3C).

Unique genes identified between *P. coccineus* and *P. vulgaris*

We performed comparative genomic studies between the two *Phaseolus* plants scarlet runner bean (*P. coccineus*) and common bean (*P. vulgaris*) [7], which diverged from each other ~4.7 Mya (Figure 3A). Overall, all chromosomes from the two species have one-versus-one corresponding relationships, with only some intra-chromosome inversions (Figure 4A). The estimated genome sizes of the two species were both ~590 Mb. Our assembly size of scarlet runner bean with HiFi reads is 593 Mb almost equal to the estimated genome size, however, the assembly size of common bean with Roche/Illumina data is only 532 Mb, missing ~58 Mb (10%) sequences. Looking into the TEs, we found that scarlet runner bean has 52 Mb more LTR-TEs than common bean which largely explains the missing components in the assembly of common bean. Being close to scarlet runner bean, common bean is also very likely to have a recent explosion of LTR-TEs (Figure 2D), resulting in many highly similar copies of TE sequences which are too difficult to assemble using short reads data. In contrast, our assembly of scarlet runner bean with ultralong HiFi data can successfully overcome this problem.

Furthermore, the better assembly of scarlet runner bean enables an annotation of 35,523 genes, much higher than the 27,433 genes identified in common bean. Requiring an alignment E-value of 1e-5, 28,813 (81.1%) scarlet runner bean genes and 26,764 (97.6%) common bean genes were matched, leaving 6,710 (18.9%) unique genes in scarlet runner and 669 (2.4%) unique genes in common bean (Figure 4C, Supplementary Table S18), which may be responsible for the

phenotypic differences and species-specific functions between the two species. Due to the poor assembly, common bean should in fact have more unique genes, which may be approximate to the number of unique genes in scarlet runner bean.

Expansion of *NIN* and *CHS* genes

Legume is special in plants for its nitrogen-fixing root nodule symbiosis (RNS) [13], which requires a set of key genes (Figure 5A). The chalcone synthase (CHS) and isoflavone synthase (IFS) are responsible for the biosynthesis of isoflavone, which attracts Rhizobia. NFR1 and NFR5 receptors perceive the Nod factors secreted from Rhizobia, and interact with downstream SYMBIOSIS RECEPTOR-LIKE KINASE (SYMRK), who further activates 3-hydroxy-3-methylglutaryl-CoA reductase (HMGR1) that induce nuclear calcium oscillations. DMI3 (DOES NOT MAKE INFECTIONS 3) detects the calcium signal, and activates IPD3 (Protein CYCLOPS) and DELLA, which further induces downstream transcription factors NSP1 (Nodule Signaling Protein 1), NSP2 (Nodule Signaling Protein 2), NIN (NODULE INCEPTION), NLP2 (NIN-like protein 2), as well as RPG (Rhizobiumdirected polar growth). At last, the *Lb* gene was expressed to produce leghemoglobin. We identified all these key genes in sword bean, scarlet runner bean, winged bean, smooth rattlebox, and butterfly pea (Figure 5B, Supplementary Table S19-S20), providing a valuable gene resource for RNS studies.

In contrast to other symbiosis-relevant genes involved in infection, NIN and RPG are only known to have NFN symbiosis–specific functions, whereas the mutation of other genes may have more pleiotropic effects. The phylogenomics studies also found that both the *NIN* and *RPG* genes

exist in root nodulating legumes, but absence or became pseudogenes in non-nodulating legumes, indicating that *NIN* and *RPG* are the essential genes for root nodulation [19]. In study, we found that the *RPG* gene was single copy in each plant (Supplementary Fig. S7), but the *NIN* gene was duplicated in the early Papilionoideae ancestor, possibly as a result of the whole genome duplication event (PWGD) occurred 55 Mya. One gene copy was retained in all the 5 plants, but the other gene copy was missing in smooth rattlebox and butterfly pea (Figure 5C, Supplementary Table S21). Therefore, sword bean, scarlet runner bean and winged bean, each have two copies of the *NIN* genes, while smooth rattlebox and butterfly pea have only one copy of the *NIN* gene for each plant.

Isoflavones, a type of polyphenolic secondary metabolite from the phenylalanine pathway in plants with a C6-C3-C6 structure, are predominantly distributed in plants of the Papilionoideae subfamily and are majorly used as the signaling molecules between leguminous plants and rhizobia [38, 39]. In this study, we identified all the gene copies of *CHS* and *IFS* (Figure 5D, Supplementary Fig. S8, and Supplementary Table S22), which are the key genes responsible for isoflavone biosynthesis. Interestingly, scarlet runner bean and winged bean each have 11 copies of *CHS* genes, almost two times of that in the other 3 plants. From the phylogenetic analysis, we found that the expansion of *CHS* genes in scarlet runner bean and winged bean was species-specific. Most of the duplicated *CHS* genes are located in a small cluster region on the same chromosome, indicating that the gene expansion occurred through local tandem gene duplications (Figure 5D).

Evolutionary history of Resistance genes

Resistance (R) gene-mediated defense plays an important role in plant defenses against all pathogens. It recognizes the pathogen-derived proteins referred to as effectors, and induces a state in the host defined as effector-triggered susceptibility (ETS), which in turn leads to the local hypersensitive response (HR) cell death to restrict pathogen growth and propagation [40]. Most cloned R genes encode intracellular NLR receptors, which are typically composed of 3 domains: a central NB (NB-ARC) domain, bordered by a C-terminal leucine-rich repeat domain (LRR), and an N-terminal coiled-coil (CC) or Toll/ interleukin-1 receptor (TIR) or resistance to powdery mildew (RPW8) domain [34]. The RPW8 domain is rare in comparison to the two major CC and TIR domains. Based on the type of N-terminal domain, the R genes were traditionally divided into 3 classes: TNL (TIR-NB-LRR), CNL (CC-NB-LRR), and RNL (RPW8-NB-LRR) (Figure 6A).

In this study, we identified 7, 9, 5, 11, 12 TNL genes, and 13, 9, 7, 6, 5 CNL genes, for sword bean, scarlet runner bean, winged bean, smooth rattlebox, and butterfly pea, respectively (Figure 6B, Supplementary Fig. S9). In addition, only one R gene with the N-terminal RPW8 domain was identified in smooth rattlebox (Supplementary Fig. S9). Overall, scarlet runner bean has equal number of TNL and CNL genes, sword bean and winged bean have more CNL genes than TNL genes, while smooth rattlebox and butterfly pea have more TNL genes than CNL genes. Through phylogenetic analysis, all the TNL and CNL genes were separated into two major branches (Figure 6C), consistent with previous studies which shown that the three classes of NLR R-genes have evolved at the early origin of ancestral Angiosperm [41]. Using *Albizia julibrissin* as an outgroup, which belongs to the second largest subfamily Caesalpinioideae in Fabaceae (Legume), we

inferred 6 TNL orthologous groups (OGs) and 9 CNL OGs within family Fabaceae, with each OG derived from a single gene in the common Fabaceae ancestor (Figure 6C). The 6 TNL and 9 CNL ancestral genes in Fabaceae, were derived from multiple whole genome polyploidization events or gene duplications since the born of Angiosperm.

Discussion

In this study, we generated chromosome-level genome assemblies and high-quality gene annotations for 5 underutilized legume crops. The assembly quality is near telomere-to-telomere level, with only a few gaps in each constructed chromosome. Smooth rattlebox and butterfly pea have much larger genome sizes than sword bean, scarlet runner bean and winged bean, due to a relatively long period of LTR expansion in the evolutionary history of these two species. Phylogeny and divergence time for the studied plants were inferred with the single-copy gene families, and a whole genome duplication event at the origin of the papilionoid clade (PWGD) around 55 Mya was detected for all the studies species. By comparing the HiFi-assembled scarlet runner bean and the low-coverage assembly of common bean derived from Roche/Illuimna data, we found huge advantage of HiFi-assembly in overcoming the difficulties of recently burst repetitive sequences, and identified 6,710 unique genes in scarlet runner bean and only 669 unique gene in common bean. These genomic resources will promote evolutionary and comparative genomics studies in Fabaceae.

In comparison to the grains, most beans have much higher protein content, which is closely related with nitrogen-fixing root nodule symbiosis. Based on sequence homology, we identified all the key genes involved in root nodulation in the 5 studied plants. The *NIN* gene was duplicated in

the early Papilionoideae ancestor, and then gene loss happened on one branch in smooth rattlebox and butterfly pea. We also found that the *CHS* genes were obviously expanded through local tandem duplication in scarlet runner bean and winged bean. Our results provide more genomic evidence for the evolution of nitrogen-fixing root nodule symbiosis (RNS), which will promote the molecular breeding of more efficient RNS cultivar and benefit the utilization of global nitrogen-fixing by legume plants.

Improvement of disease resistance in crops has great potential to increase productivity. Huge losses caused by pathogenic fungi, bacteria, nematodes, oomycetes, and viruses could be mitigated by breeding of disease-resistant cultivars. The 5 underutilized legume crops in this study are much less human-selected than other well-known legume crops such as soybean, thus, they may include more powerful resistance (R) genes. In this study, we identified all the R genes in the 5 studied plants, which can be divided mainly into two classes TNL and CNL. Notably, sword bean and winged bean have more CNL genes, but smooth rattlebox and butterfly pea have more TNL genes. In future, these R genes can be transferred into major legume crops such as soybean to improve its disease resistance, which will reduce the application of chemical pesticide and promote the global food security.

Methods

Plant materials and sequencing

Commercial seed of sword bean (LVBAO cultivar) was obtained from NONGZHIZI SEEDS company (ZhuZhou, Hunan, China). Commercial seed of scarlet runner bean (Climbing cultivar)

was obtained from JINMINXINNONG company (Fuzhou, Fujian, China). Commercial seed of winged bean (DUOXI-ginseng cultivar) was obtained from BOSITE agricultural technology company (Shenyang, Liaoning, China). Commercial seed of smooth rattlebox (Three-ellipse-leaf cultivar) was obtained from the forestry-bureau permitted seed store company (Jiaxing, Zhejiang, China). The young seedling of butterfly pea (Blue-flower cultivar) was obtained from LIUYI flowers and fruit seedlings company.The seeds were grown in plant growth chamber, and young leaves from a single plant for each species were used to extract genomic DNA using the Hi-DNAsecure Plant Kit (TIANGEN DP350, China). The genomic DNA was used to prepare 20-kb inserts sequencing library by SMRTbell Express Template Prep Kit 2.0 (PacBio, USA), and sequenced on Sequel II with the HiFi mode (PacBio, USA). The young leaves from the same plant were also used for Hi-C sequencing on the Illumina NovaSeq 6000 in PE150 mode. The roots, stems, leaves, and flowers of each species were sampled to extract total RNA using the RNeasy Plant Mini Kit (QIAGEN, Germany). The extracted RNA was pooled together for full-length cDNA sequencing on PacBio Sequel II with Iso-Seq mode (PacBio, USA).

Genome assembly and annotation

The contigs for sword bean, scarlet runner bean, and winged bean were assembled from PacBio HiFi reads utilizing HIFIASM version 0.16.1 [42] with parameter “-l 0”, and the contigs for smooth rattlebox, and butterfly pea were assembled from PacBio HiFi reads utilizing HIFIASM version 0.19.5 [42] with parameter “-l 0”. Contaminations were removed by aligning all contigs to chloroplast and mitochondria sequences downloaded from the NCBI database, using MINIMAP2 version 2.20 [43] with an identity >0.95 and coverage >0.95. The remaining contigs were used to represent the nuclear contigs, and the completeness was evaluated using BUSCO

version 5.1.2 [44] with ORTHODB version 10 (embryophyta lineage). Ultimately, the Hi-C reads were aligned to the nuclear contigs and Hi-C contact matrices among the contig bins were generated utilizing HIC-PRO version 3.1.0 [45]. Utilizing the Hi-C linkage information between contig ends, nuclear contigs (with sizes exceeding 1 Mb) were assembled into scaffolds at the chromosome level utilizing ENDHIC version 1.0 [46].

Tandem repeat elements (TRs) were detected using Tandem Repeats Finder (TRF) version 4.09 [47]. Interspersed repeat elements (TEs) were identified through a three-step process: (1) The prediction of structurally intact transposon elements (TEs), including long-terminal-repeat retrotransposons (LTR-RTs), DNA transposon, Helitron, etc., was accomplished using EDTA version 1.9.9 [48]. Concurrently, an intact TE library was generated. (2) Incomplete and homology TEs were detected against the above-mentioned intact TE library, Repbase database version 26.05 (plant lineage), and the Protein-coding TE database using REPEATMASKER version 4.1.2 (http://www.repeatmasker.org). (3) A de novo TE library was generated from the masked genome with all the above-mentioned identified TEs, using REPEATMODELER version 2.0.1 (http://www.repeatmasker.org/RepeatModeler), then the TE sequences in the library were classified using TERL v1.0 [49]. Then, the classified TE sequences were used by REPEATMASKER to identify species-specific TEs in the genome. Ultimately, merging the overlapping coordinates and removing any redundancy was used to produce a non-redundant TE annotation. All TE elements larger than 80 bp in size of the scarlet runner bean and the winged bean, and those larger than 200 bp in size of the sword bean, the smooth rattlebox, and the butterfly pea were soft-masked (uppercase to lowercase) on their genome sequences for gene

prediction.

Transcript and homology hints files were used to predict protein-coding gene models by AUGUSTUS version 3.4.0 [50]. The AUGUSTUS parameters of gene prediction were generated from the intermediate outcomes of the BUSCO assessment of genome assembly. Full-length transcripts generated by PacBio Iso-Seq were aligned to the genome using GMAP version 2019-12-01 [51] and using Augustus filter script with parameters "--minId=95 --minCover=95". To obtain homology hints, the proteome of 12 representative Papilionoideae species (*Phaseolus vulgaris* [7], *Vigna angularis* [7], *Lablab purpureus* [7], *Glycine max* [7], *Pueraria montana* [24], *Mucuna pruriens* [23], *Pisum sativum* [30], *Medicago truncatula* [31], *Lotus japonicus* [32], *Aeschynomene evenia [33]*, *Arachis hypogaea [34]*, *Styphnolobium japonicum [35]*) were aligned to the genome using exonerate version 2.4.0 [52]. The homology and transcript alignment results were transformed into hints files to support gene prediction of AUGUSTUS. To further filter the TE-contaminated genes, the genes whose coordinates overlap more than 99% of the annotated TEs were removed from the gene sets. BUSCO version 5.1.2 [44] was used to evaluate the completeness of the gene sets.

For the annotation of gene function, protein sequences were aligned to KEGG and NCBI-NR databases using the DIAMON version 0.8.2 [53] with parameter "E-value 1E−5" to obtain the best hits. The INTERPROSCAN version 5.52-86 [54] with database searching of CDD-3.18, Coils-2.2.1, Gene3D-4.3.0, Hamap-2020_05, MobiDBLite-2.0, PANTHER-15.0, Pfam-33.1, PIRSF-3.10, PIRSR-2021_02, PRINTS-42.0, ProSitePatterns-2021_01, ProSiteProfiles-2021_01, SFLD-4, SMART-7.1, SUPERFAMILY-1.75 and TIGRFAM-15.0, was used to detect the protein domain

information and obtain related Gene Ontology (GO) terms. The 8S, 18S and 28S ribosomal RNA (rRNA) were identified utilizing RNAMMER v1.2 [55], and the transfer RNAs (tRNAs) were identified utilizing TRNASCAN-SE v2.0 [56].

Phylogeny and polyploidization Analysis

To construct the orthogroups, we used ORTHOFINDER version 2.5.2 [57] with parameters "-M msa -A mafft -T fasttree -1 -y", with 12 Papilionoideae species (*Phaseolus vulgaris* [7], *Vigna angularis* [21], *Lablab purpureus* [22], *Glycine max* [29], *Pueraria montana* [24], *Mucuna pruriens* [23], *Pisum sativum* [30], *Medicago truncatula* [31], *Lotus japonicus* [32], *Aeschynomene evenia [33]*, *Arachis hypogaea* [34], *Styphnolobium japonicum* [35]) and one outgroup species *Vitis vinifera* [36].

From the orthogroups of ORTHOFINDER, the OG with *Arachis hypogaea* (recent WGD) [34] having one or two copies and other species having only one copy were selected, then a gene of *Arachis hypogaea* from duplicate genes was randomly thrown. Subsequently, all single-copy genes of all species were used to employ multiple sequence alignment (MSA) utilizing MUSCLE version v3.8.31 [58], and these MSA were combined to create a concatenate multiple sequence alignment (CMSA). Next, the CMSA was used to build a species tree utilizing RAXML version 1.0.3 [59] with parameters "--model GTR+G --tree pars --bs-trees 100 --outgroup Vitis_vinifera". To estimate the divergence time, we used the RelTime branch method in MEGA11 [60] with one calibration time 8.0-19.5 million years ago between *Phaseolus vulgaris* and *Vigna angularis* and the other calibration time 47.7-56 million years ago between *Glycine max* and *Arachis hypogaea*. The two calibration times were obtained from TimeTree (www.timetree.org). Subsequently, the

expansion and contraction of gene families was identified using CAFE version 5 [61] with the parameter "-k5".

MCSCANX [62] was used to identify collinear gene blocks with more than five collinear genes. Synteny figures of the whole genome were plotted using the Java programs dual_synteny_plotter and dot_plotter from the MCSCANX package. According to the result of collinear genes, KaKs_CALCULATOR v2.0 [63] with GMYN model was employed to calculate the synonymous substitution rate (*Ks*) value for syntenic gene pairs. Chromosome collinearity among species was drawn using JCVI (https://github.com/tanghaibao/jcvi) with parameter --cscore=.99”.

Analysis of genes involved in nitrogen-fixing root nodulation

The protein sequences associated with nitrogen-fixing nodulation were obtained from NCBI and Phytozome, and were aligned to the five Papilionoideae species using the ‘blastp’ algorithm in DIAMOND version 0.8.28 [53], with the parameters “--more-sensitive --evalue 0.00001”. The Alignment results were further refined with a 50% identity and 60% coverage threshold. In this way, we identified the potential genes involved in nitrogen-fixing root nodulation.

To analyze the phylogenetic relationships, the protein sequences within each gene family of *NIN*, *RPG*, *CHS*, and *IFS* were aligned independently using the MUSCLE version 3.8.31 [58]. Subsequently, phylogenetic trees were constructed utilizing the FastTree version 2.1.11 [64]. *V. vinifera* was used as an outgroup. The phylogenetic trees for *NIN* and *RPG* genes were visualized using FigTree version1.4.4 (FigTree (ed.ac.uk)), while those for *CHS* and *IFS* genes were

displayed using iTol (https://itol.embl.de/).

Analysis of R genes

The protein sequences of all genes from the five studied plants and *Albizia julibrissin* (NCBI: PRJNA1005079) were searched against the HMM profile of all domains using the hmmsearch program in HMMER version 3.1b2 [65] with the parameters '-E 1e-5 --domE 1e-5'. The genes with at least one of the identified domains, including TIR (PF01582), TIR_2 (PF13676), RPW8 (PF05659), NB-ARC (PF00931), LRR_1 (PF00560), LRR_2 (PF07723), LRR_3 (PF07725), LRR_4 (PF12799), LRR_5 (PF13306), LRR_6 (PF13516), LRR_8 (PF13855), and LRR_9 (PF14580), were chosen as the primary potential R genes. In addition, Coiled-coil (CC) domains were furhter annotated using the Coils database from INTERPROSCAN version 5.52-86 [54]. The genes with TIR-NB-LRR, CC-NB-LRR and RPW8-NB-LRR domain structures were classified as TNL, CNL and RNL R genes, respectively. The alignment of protein sequences for CNL and TNL R genes was performed using MUSCLE version 3.8.31 [58]. Subsequently, the phylogenetic tree was constructed by FastTree version 2.1.11 [64], which was displayed using iTOL (https://itol.embl.de/).

Acknowledgements

We also thank Prof. Shifeng Cheng for giving helpful suggestions.

Data availability

The genomic and transcriptomic sequencing reads generated in this study have been deposited in SRA of NCBI under the accession PRJNA1001638, PRJNA1002813,

PRJNA1003673, PRJNA1014360, PRJNA1016062 for *Canavalia gladiata*, *Phaseolus coccineus*, *Psophocarpus tetragonolobus*, *Crotalaria pallida*, and *Clitoria ternatea*, respectively. The genome assemblies and gene annotations have been deposited at GenBank of NCBI under the accession JAYMYQ000000000, JAYMYR000000000, JAYMYS000000000, JAYWIO000000000, JAYKXN000000000 and also have been deposited at Figshare [10.6084/m9.figshare.24994343, 10.6084/m9.figshare.24995750, 10.6084/m9.figshare.24995753, 10.6084/m9.figshare.24995756, 10.6084/m9.figshare.24995759] for *Canavalia gladiata*, *Phaseolus coccineus*, *Psophocarpus tetragonolobus*, *Crotalaria pallida*, and *Clitoria ternatea*, respectively.

Funding

This work was supported by the Shenzhen Science and Technology Program (JCYJ20190814163805604, KQTD20180411143628272); Fund of Key Laboratory of Shenzhen (ZDSYS20141118170111640) and The Agricultural Science and Technology Innovation Program.

Author contributions

L.H.Y. and F.J. prepared the genomic and transcriptomic sequencing samples. L.H.Y., L.H.L., F.J., A.Q.W., R.C., H.C.W. and S.H.M. completed the bioinformatic analyses. L.H.Y. and L.H.L. made the tables, figures, and Supplemental information. W.F. supervised the project, and W.F., L.H.Y and L.H.L wrote the manuscript, and all authors revised and approved the final version of this manuscript.

Competing Interests

The authors declare no competing interest.

References

1. Zhao Y, Zhang R, Jiang KW, Qi J, Hu Y, Guo J, et al. Nuclear phylotranscriptomics and

phylogenomics support numerous polyploidization events and hypotheses for the evolution of rhizobial nitrogen-fixing symbiosis in Fabaceae. Mol Plant. 2021;14 5:748-73. doi:10.1016/j.molp.2021.02.006.
2. Schmutz J, Cannon SB, Schlueter J, Ma J, Mitros T, Nelson W, et al. Genome sequence of the palaeopolyploid soybean. Nature. 2010;463 7278:178-83. doi:10.1038/nature08670.
3. Zhuang W, Chen H, Yang M, Wang J, Pandey MK, Zhang C, et al. The genome of cultivated peanut provides insight into legume karyotypes, polyploid evolution and crop domestication. Nat Genet. 2019;51 5:865-76. doi:10.1038/s41588-019-0402-2.
4. Jayakodi M, Golicz AA, Kreplak J, Fechete LI, Angra D, Bednar P, et al. The giant diploid faba genome unlocks variation in a global protein crop. Nature. 2023;615 7953:652-9. doi:10.1038/s41586-023-05791-5.
5. Kang YJ, Kim SK, Kim MY, Lestari P, Kim KH, Ha BK, et al. Genome sequence of mungbean and insights into evolution within Vigna species. Nat Commun. 2014;5:5443. doi:10.1038/ncomms6443.
6. Kreplak J, Madoui MA, Capal P, Novak P, Labadie K, Aubert G, et al. A reference genome for pea provides insight into legume genome evolution. Nature Genetics. 2019;51 9:1411-+. doi:10.1038/s41588-019-0480-1.
7. Schmutz J, McClean PE, Mamidi S, Wu GA, Cannon SB, Grimwood J, et al. A reference genome for common bean and genome-wide analysis of dual domestications. Nat Genet. 2014;46 7:707-13. doi:10.1038/ng.3008.
8. Shen C, Du HL, Chen Z, Lu HW, Zhu FG, Chen H, et al. The Chromosome-Level Genome Sequence of the Autotetraploid Alfalfa and Resequencing of Core Germplasms Provide Genomic Resources for Alfalfa Research. Molecular Plant. 2020;13 9:1250-61. doi:10.1016/j.molp.2020.07.003.
9. Ayilara MS, Abberton M, Oyatomi OA, Odeyemi O and Babalola OO. Potentials of underutilized legumes in food security. Front Soil Sci. 2022;2 doi:10.3389/fsoil.2022.1020193.
10. Maneechot O, Hahor W, Thongprajukaew K, Nuntapong N and Bubaka S. A natural blue colorant from butterfly pea (*Clitoria ternatea*) petals for traditional rice cooking. J Food Sci Tech Mys. 2023;60 8:2255-64. doi:10.1007/s13197-023-05752-w.
11. Hu Y, Chen XJ, Hu M, Zhang DW, Yuan S, Li P, et al. Medicinal and edible plants in the treatment of dyslipidemia: advances and prospects. Chin Med-Uk. 2022;17 1 doi:10.1186/s13020-022-00666-9.
12. Gautam AK, Sharma D, Sharma J and Saini KC. Legume lectins: Potential use as a diagnostics and therapeutics against the cancer. Int J Biol Macromol. 2020;142:474-83. doi:10.1016/j.ijbiomac.2019.09.119.
13. Desbrosses GJ and Stougaard J. Root Nodulation: A Paradigm for How Plant-Microbe Symbiosis Influences Host Developmental Pathways. Cell Host Microbe. 2011;10 4:348-58. doi:10.1016/j.chom.2011.09.005.
14. Huisman R and Geurts R. A Roadmap toward Engineered Nitrogen-Fixing Nodule Symbiosis. Plant Commun. 2020;1 1:100019. doi:10.1016/j.xplc.2019.100019.
15. Young ND, Debelle F, Oldroyd GED, Geurts R, Cannon SB, Udvardi MK, et al. The Medicago genome provides insight into the evolution of rhizobial symbioses. Nature. 2011;480 7378:520-4. doi:10.1038/nature10625.

16. Sato S, Nakamura Y, Kaneko T, Asamizu E, Kato T, Nakao M, et al. Genome structure of the legume, Lotus japonicus. DNA Res. 2008;15 4:227-39. doi:10.1093/dnares/dsn008.
17. Yang J, Lan LY, Jin Y, Yu N, Wang D and Wang E. Mechanisms underlying legume-rhizobium symbioses. J Integr Plant Biol. 2022;64 2:244-67. doi:10.1111/jipb.13207.
18. Kundu S and Hargrove MS. Distal heme pocket regulation of ligand binding and stability in soybean leghemoglobin. Proteins. 2003;50 2:239-48. doi:10.1002/prot.10277.
19. Griesmann M, Chang Y, Liu X, Song Y, Haberer G, Crook MB, et al. Phylogenomics reveals multiple losses of nitrogen-fixing root nodule symbiosis. Science. 2018;361 6398 doi:10.1126/science.aat1743.
20. Libourel C, Keller J, Brichet L, Cazale AC, Carrere S, Vernie T, et al. Comparative phylotranscriptomics reveals ancestral and derived root nodule symbiosis programmes. Nat Plants. 2023;9 7:1067-80. doi:10.1038/s41477-023-01441-w.
21. Yang K, Tian ZX, Chen CH, Luo LH, Zhao B, Wang Z, et al. Genome sequencing of adzuki bean (Vigna angularis) provides insight into high starch and low fat accumulation and domestication. P Natl Acad Sci USA. 2015;112 43:13213-8. doi:10.1073/pnas.1420949112.
22. Njaci I, Waweru B, Kamal N, Muktar MS, Fisher D, Gundlach H, et al. Chromosome-level genome assembly and population genomic resource to accelerate orphan crop lablab breeding. Nat Commun. 2023;14 1:1915. doi:10.1038/s41467-023-37489-7.
23. Hao S, Ge Q, Shao Y, Tang B, Fan G, Qiu C, et al. Chromosomal-level genome of velvet bean (Mucuna pruriens) provides resources for L-DOPA synthetic research and development. DNA Res. 2022;29 5 doi:10.1093/dnares/dsac031.
24. Mo CJ, Wu ZD, Shang XH, Shi PL, Wei MH, Wang HY, et al. Chromosome-level and graphic genomes provide insights into metabolism of bioactive metabolites and cold-adaption of Pueraria lobata var. montana. DNA Research. 2022;29 5 doi:10.1093/dnares/dsac030.
25. Chen HF, Yao XZ, Cao BH, Zhang BH, Lu LT and Mao PL. A chromosome-level genome assembly of Styphnolobium japonicum combined with comparative genomic analyses offers insights on the evolution of flavonoid and lignin biosynthesis. Ind Crop Prod. 2022;187 doi:10.1016/j.indcrop.2022.115336.
26. Liu B, Shi Y, Yuan J, Hu X, Zhang H, Li N, et al. Estimation of genomic characteristics by analyzing k-mer frequency in de novo genome projects. 2013.
27. Rhie A, Walenz BP, Koren S and Phillippy AM. Merqury: reference-free quality, completeness, and phasing assessment for genome assemblies. Genome Biol. 2020;21 1:245. doi:10.1186/s13059-020-02134-9.
28. Galindo-González L, Mhiri C, Deyholos MK and Grandbastien MA. LTR-retrotransposons in plants: Engines of evolution. Gene. 2017;626:14-25. doi:10.1016/j.gene.2017.04.051.
29. Shen YT, Du HL, Liu YC, Ni LB, Wang Z, Liang CZ, et al. Update soybean Zhonghuang 13 genome to a golden reference. Sci China Life Sci. 2019;62 9:1257-60. doi:10.1007/s11427-019-9822-2.
30. Yang T, Liu R, Luo Y, Hu S, Wang D, Wang C, et al. Improved pea reference genome and pan-genome highlight genomic features and evolutionary characteristics. Nat Genet. 2022;54 10:1553-63. doi:10.1038/s41588-022-01172-2.
31. Pecrix Y, Staton SE, Sallet E, Lelandais-Brère C, Moreau S, Carrère S, et al. Whole-genome landscape of symbiotic genes. Nature Plants. 2018;4 12:1017-25. doi:10.1038/s41477-018-0286-7.

32. Kamal N, Mun T, Reid D, Lin JS, Akyol TY, Sandal N, et al. Insights into the evolution of symbiosis gene copy number and distribution from a chromosome-scale Gifu genome sequence. DNA Research. 2020;27 3 doi:10.1093/dnares/dsaa015.
33. Quilbe J, Lamy L, Brottier L, Leleux P, Fardoux J, Rivallan R, et al. Genetics of nodulation in Aeschynomene evenia uncovers mechanisms of the rhizobium-legume symbiosis. Nat Commun. 2021;12 1:829. doi:10.1038/s41467-021-21094-7.
34. Chen XP, Lu Q, Liu H, Zhang JA, Hong YB, Lan HF, et al. Sequencing of Cultivated Peanut，Yields Insights into Genome Evolution and Oil Improvement. Molecular Plant. 2019;12 7:920-34. doi:10.1016/j.molp.2019.03.005.
35. Lei W, Wang Z, Cao M, Zhu H, Wang M, Zou Y, et al. Chromosome-level genome assembly and characterization of Sophora Japonica. DNA Res. 2022;29 3 doi:10.1093/dnares/dsac009.
36. Jaillon O, Aury JM, Noel B, Policriti A, Clepet C, Casagrande A, et al. The grapevine genome sequence suggests ancestral hexaploidization in major angiosperm phyla. Nature. 2007;449 7161:463-7. doi:10.1038/nature06148.
37. Cannon SB, McKain MR, Harkess A, Nelson MN, Dash S, Deyholos MK, et al. Multiple Polyploidy Events in the Early Radiation of Nodulating and Nonnodulating Legumes. Mol Biol Evol. 2015;32 1:193-210. doi:10.1093/molbev/msu296.
38. Subramanian S, Stacey G and Yu O. Endogenous isoflavones are essential for the establishment of symbiosis between soybean and. Plant J. 2006;48 2:261-73. doi:10.1111/j.1365-313X.2006.02874.x.
39. Al-Maharik N. Isolation of naturally occurring novel isoflavonoids: an update. Nat Prod Rep. 2019;36 8:1156-95. doi:10.1039/c8np00069g.
40. Gururani MA, Venkatesh J, Upadhyaya CP, Nookaraju A, Pandey SK and Park SW. Plant disease resistance genes: Current status and future directions. Physiol Mol Plant P. 2012;78:51-65. doi:10.1016/j.pmpp.2012.01.002.
41. Shao ZQ, Xue JY, Wu P, Zhang YM, Wu Y, Hang YY, et al. Large-Scale Analyses of Angiosperm Nucleotide-Binding Site-Leucine-Rich Repeat Genes Reveal Three Anciently Diverged Classes with Distinct Evolutionary Patterns. Plant Physiol. 2016;170 4:2095-109. doi:10.1104/pp.15.01487.
42. Cheng H, Concepcion GT, Feng X, Zhang H and Li H. Haplotype-resolved de novo assembly using phased assembly graphs with hifiasm. Nat Methods. 2021;18 2:170-5. doi:10.1038/s41592-020-01056-5.
43. Li H. Minimap2: pairwise alignment for nucleotide sequences. Bioinformatics. 2018;34 18:3094-100. doi:10.1093/bioinformatics/bty191.
44. Simao FA, Waterhouse RM, Ioannidis P, Kriventseva EV and Zdobnov EM. BUSCO: assessing genome assembly and annotation completeness with single-copy orthologs. Bioinformatics. 2015;31 19:3210-2. doi:10.1093/bioinformatics/btv351.
45. Servant N, Varoquaux N, Lajoie BR, Viara E, Chen CJ, Vert JP, et al. HiC-Pro: an optimized and flexible pipeline for Hi-C data processing. Genome Biol. 2015;16:259. doi:10.1186/s13059-015-0831-x.
46. Wang S, Wang H, Jiang F, Wang A, Liu H, Zhao H, et al. EndHiC: assemble large contigs into chromosome-level scaffolds using the Hi-C links from contig ends. BMC Bioinformatics. 2022;23 1:528. doi:10.1186/s12859-022-05087-x.

47. Benson G. Tandem repeats finder: a program to analyze DNA sequences. Nucleic Acids Res. 1999;27 2:573-80. doi:10.1093/nar/27.2.573.
48. Ou S, Su W, Liao Y, Chougule K, Agda JRA, Hellinga AJ, et al. Author Correction: Benchmarking transposable element annotation methods for creation of a streamlined, comprehensive pipeline. Genome Biol. 2022;23 1:76. doi:10.1186/s13059-022-02645-7.
49. da Cruz MHP, Domingues DS, Saito PTM, Paschoal AR and Bugatti PH. TERL: classification of transposable elements by convolutional neural networks. Brief Bioinform. 2021;22 3 doi:10.1093/bib/bbaa185.
50. Stanke M, Diekhans M, Baertsch R and Haussler D. Using native and syntenically mapped cDNA alignments to improve de novo gene finding. Bioinformatics. 2008;24 5:637-44. doi:10.1093/bioinformatics/btn013.
51. Wu TD and Watanabe CK. GMAP: a genomic mapping and alignment program for mRNA and EST sequences. Bioinformatics. 2005;21 9:1859-75. doi:10.1093/bioinformatics/bti310.
52. Slater GS and Birney E. Automated generation of heuristics for biological sequence comparison. BMC Bioinformatics. 2005;6:31. doi:10.1186/1471-2105-6-31.
53. Buchfink B, Reuter K and Drost HG. Sensitive protein alignments at tree-of-life scale using DIAMOND. Nature Methods. 2021;18 4:366-+. doi:10.1038/s41592-021-01101-x.
54. Blum M, Chang HY, Chuguransky S, Grego T, Kandasaamy S, Mitchell A, et al. The InterPro protein families and domains database: 20 years on. Nucleic Acids Research. 2021;49 D1:D344-D54. doi:10.1093/nar/gkaa977.
55. Lagesen K, Hallin P, Rodland EA, Stærfeldt HH, Rognes T and Ussery DW. RNAmmer:: consistent and rapid annotation of ribosomal RNA genes. Nucleic Acids Research. 2007;35 9:3100-8. doi:10.1093/nar/gkm160.
56. Chan PP, Lin BY, Mak AJ and Lowe TM. tRNAscan-SE 2.0: improved detection and functional classification of transfer RNA genes. Nucleic Acids Res. 2021;49 16:9077-96. doi:10.1093/nar/gkab688.
57. Emms DM and Kelly S. OrthoFinder: phylogenetic orthology inference for comparative genomics. Genome Biol. 2019;20 1:238. doi:10.1186/s13059-019-1832-y.
58. Edgar RC. MUSCLE: multiple sequence alignment with high accuracy and high throughput. Nucleic Acids Research. 2004;32 5:1792-7. doi:10.1093/nar/gkh340.
59. Kozlov AM, Darriba D, Flouri T, Morel B and Stamatakis A. RAxML-NG: a fast, scalable and user-friendly tool for maximum likelihood phylogenetic inference. Bioinformatics. 2019;35 21:4453-5. doi:10.1093/bioinformatics/btz305.
60. Tamura K, Stecher G and Kumar S. MEGA11 Molecular Evolutionary Genetics Analysis Version 11. Mol Biol Evol. 2021;38 7:3022-7. doi:10.1093/molbev/msab120.
61. Mendes FK, Vanderpool D, Fulton B and Hahn MW. CAFE 5 models variation in evolutionary rates among gene families. Bioinformatics. 2020;36 22-23:5516-8. doi:10.1093/bioinformatics/btaa1022.
62. Wang YP, Tang HB, DeBarry JD, Tan X, Li JP, Wang XY, et al.: a toolkit for detection and evolutionary analysis of gene synteny and collinearity. Nucleic Acids Research. 2012;40 7 doi:10.1093/nar/gkr1293.
63. Wang D, Zhang Y, Zhang Z, Zhu J and Yu J. KaKs_Calculator 2.0: a toolkit incorporating gamma-series methods and sliding window strategies. Genomics Proteomics Bioinformatics. 2010;8 1:77-80. doi:10.1016/S1672-0229(10)60008-3.

64. Price MN, Dehal PS and Arkin AP. FastTree: Computing Large Minimum Evolution Trees with Profiles instead of a Distance Matrix. Mol Biol Evol. 2009;26 7:1641-50. doi:10.1093/molbev/msp077.
65. Mistry J, Finn RD, Eddy SR, Bateman A and Punta M. Challenges in homology search: HMMER3 and convergent evolution of coiled-coil regions. Nucleic Acids Research. 2013;41 12 doi:10.1093/nar/gkt263.

Table

Table 1. Statistics of genome assembly and annotation.

Genomic features	*Canavalia gladiata*	*Phaseolus coccineus*	*Psophocarpus tetragonolobus*	*Crotalaria pallida*	*Clitoria ternatea*
**Genome assembly**					
**Estimated genome size by K-mer (Mb)**	650	593	689	1,331	1,761
**Total assembly size (bp)**	619,186,046	592,734,161	712,813,888	1,217,645,575	1,724,627,994
**Contig N50 size (bp)**	39,462,069	39,559,522	13,237,817	100,840,643	126,428,166
**Scaffold N50 size (bp)**	55,284,388	52,871,251	79,694,302	142,152,887	168,933,288
**% of sequences anchored to chromosomes**	97.5%	95.7%	93.7%	98.2%	97.5%
**% of telomeres assembled**	72.7%	77.3%	55.6%	93.8%	75.0%
**busco complete rate of the genome**	99.4%	99.3%	99.2%	99.0%	99.0%
**QV**	70.0	74.3	69.5	69.6	72.2
**Genome annotation**					
**Length and % of tandem repeats (bp)**	105,656,450 (17.1%)	54,428,626 (9.2%)	134,417,242 (18.9%)	119,729,388 (9.8%)	130,389,725 (7.6%)
**Length and % of TE sequences (bp)**	341,308,218 (55%)	376,131,126 (63%)	456,822,987 (64%)	994,314,842 (82%)	148,366,6381 (86%)
**Number of tRNA genes**	970	1,141	1,283	1,382	2,307
**Number of rRNA (5S + 18S + 28S) genes**	1,535	5,030	3,020	6,268	3,158
**Number of protein-coding gene models**	51,158	35,523	40,081	48,759	40,267
**Total CDS size and % in genome (bp)**	50,888,808 (8.2%)	42,292,638 (7.1%)	43,752,003 (7.4%)	51,214,428 (4.2%)	41,669,007 (2.4%)
**BUSCO complete rate of the genes**	99.4%	99.6%	99.2%	98.1%	98.9%

Figure Legends

Figure 1. Circos plot of genomic annotations. **(A)** *Canavalia gladiata*, **(B)** P*haseolus coccineus*, **(C)** *Psophocarpus tetragonolobus*, **(D)** *Crotalaria pallida* and **(E)** *Clitoria ternatea*. The 5 circular

tracks from inner to outer refer to (a) GC percentage, (b) transposable element (TE) density, (c) tandem repeat (TR) density, and (d) gene density. These features were calculated by sliding 1-Mb windows. Pictures of species are placed inside the center of the circos plot.

Figure 2. TEs in the 5 sequenced species in this study. (A) Distribution of various types of transposable elements (TEs) in each species. **(B)** a scatter plot illustrating the correlation between the length of long terminal repeats (LTRs) and the genome size. **(C)** Distribution of various types of long terminal repeats (LTRs) in each species. **(D)** the insert time distribution of intact long terminal repeats (LTRs) for each species. The sequence of LTRs from intact LTR identified by EDTA version 1.9.9 were obtained, and the long terminal repeats of each LTR were aligned using MUSCLE version v3.8.31.The APE package in R and the K80 model (https://github.com/wangziwei08/LTR-insertion-time-estimation)was used to estimate the Pairwise distances from the LTR.

Figure 3. Evolution of Papilionoideae. **(A)** Phylogentic tree with divergence time estimated by the RelTime branch method in mega. Two calibration constraints were used: one was 8.0-19.5 million years ago between *Phaseolus vulgaris* and *Vigna angularis*, and the other was 47.7-56 million years ago between *Glycine max* and *Arachis hypogaea*. The five sequenced species in this study are marked with blue stars. The numbers on the side of the nodes represent divergence time values, and the whole-genome polyploidization events are indicated on the branches in red. **(B)** Homologous Ks distribution within species, paralogous gene pairs situated on collinear fragments containing over five syntenic gene pairs are employed for Ks calculation using the GMYN model in the KaKS_CALCULATOR. **(C)** Macro-synteny plots among the 5 studied species.

Figure 4. Genomic comparison between *Phaseolus coccineus* (scarlet runner bean**) and *Phaseolus vulgaris* (**common bean). **(A)** Macro-synteny blocks between *P. coccineus* and *P. vulgaris*. Collinear fragments containing over 20 syntenic gene pairs are utilized. Pc and Pv represent *P. coccineus* and *P. vulgaris* respectively. **(B)** Distribution of LTR TEs, DNA TEs, other TEs, tandem repeats (TR), and Non-repeat regions in *P. coccineus* and *P. vulgaris*. **(C)** Overlap of the reference gene sets between *P. coccineus* and *P. vulgaris*. The protein sequences are aligned using Diamond with the parameters "--sensitive --evalue 1e-5". Genes that remain unaligned are considered as species-specific genes.

Figure 5. Root nodulation symbiosis. **(A)** Pathway of crucial genes involved in symbiotic nodulation and nitrogen fixation for Papilionoideae. The model figure is drawn using Figdraw. **(B)** The gene number of symbiotic pathway identified in *C.gladiata*, *P. coccineus*, *P.tetragonolobus*, *C.pallida*, and *C.ternatea*. **(C)** Gene tree for *NIN*. Members of the gene family were obtained from orthoFinder orthogroups, and the gene tree was constructed by FastTree. Bootstrap values are shown on each branch, and the two duplicated branches were highlighted. *V. vinifera* is utilized as the outgroup. **(D)** Gene tree for *CHS*, with similar style to *NIN*. The genes from the five sequenced

species were shown in five different colors, and the tandem replicated genes of different species have been highlighted using distinct background colors.

Figure 6. Resistance (R) genes. (A) Model figure for three types of R genes TNL, CNL and RNL. TNL consists of TIR, NB-ARC, and LRR domains, CNL consists of CC, NB-ARC, and LRR domains, while RNL consists of RPW8, NB-ARC, and LRR domains from N-terminal to C-terminal. **(B)** The number of CNL-type and TNL-type R genes in *C.gladiata*, *P. coccineus*, *P.tetragonolobus*, *C.pallida*, *C.ternatea* and *A.julibrissin* species, respectively. **(C)** The phylogenetic tree of all identified R genes in the 5 studied species and one outgroup (*A.julibrissin*). The two major branches TNL and CNL are labeled with different background colors, and a solid circle on the clade represents an OG, and the outgroup is marked with a gray graph. The R genes from various species are differentiated by various symbols.

Figure 1

Click here to access/download;Figure;figure1.pdf

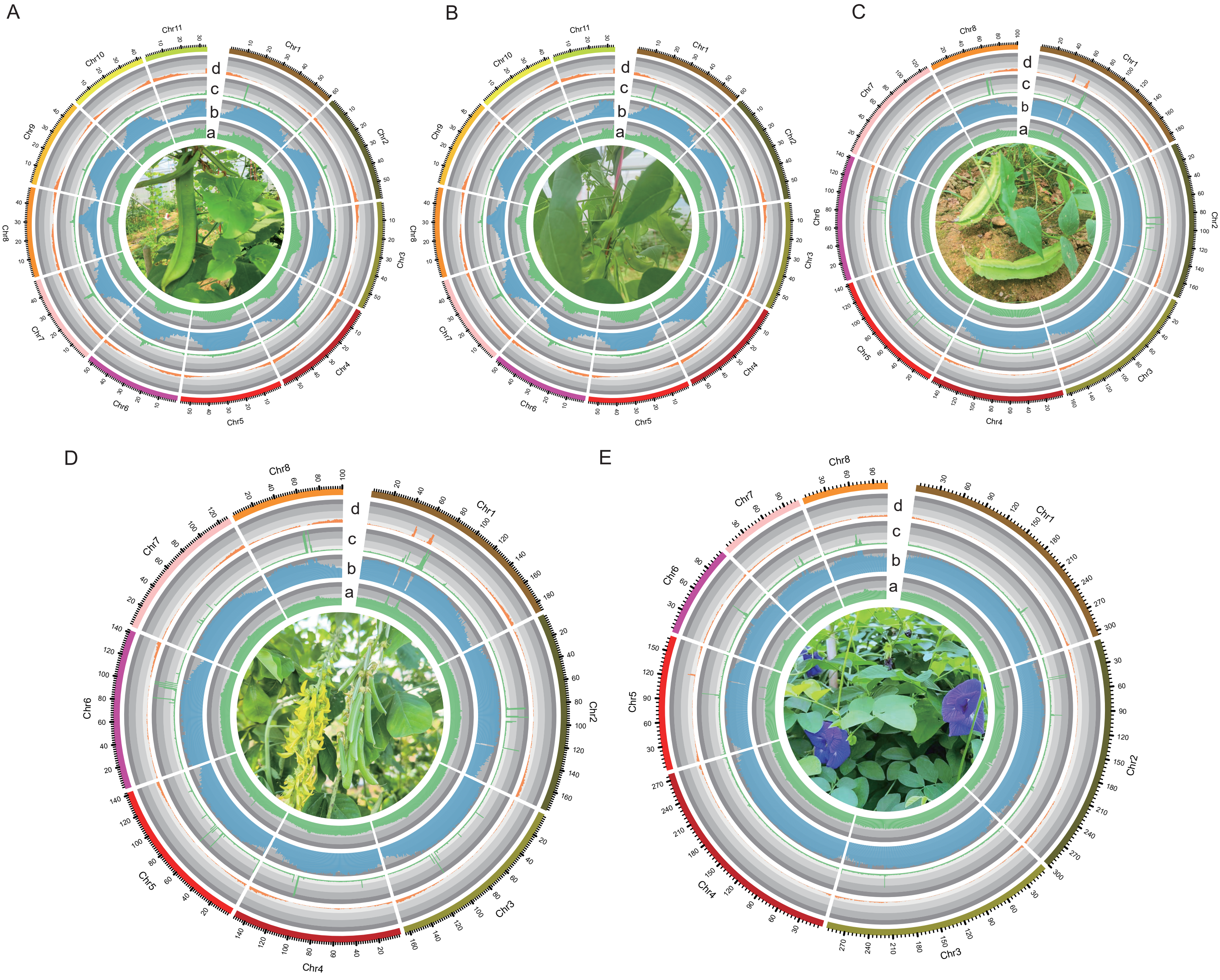

Figure 2

Click here to access/download;Figure;figure2.pdf

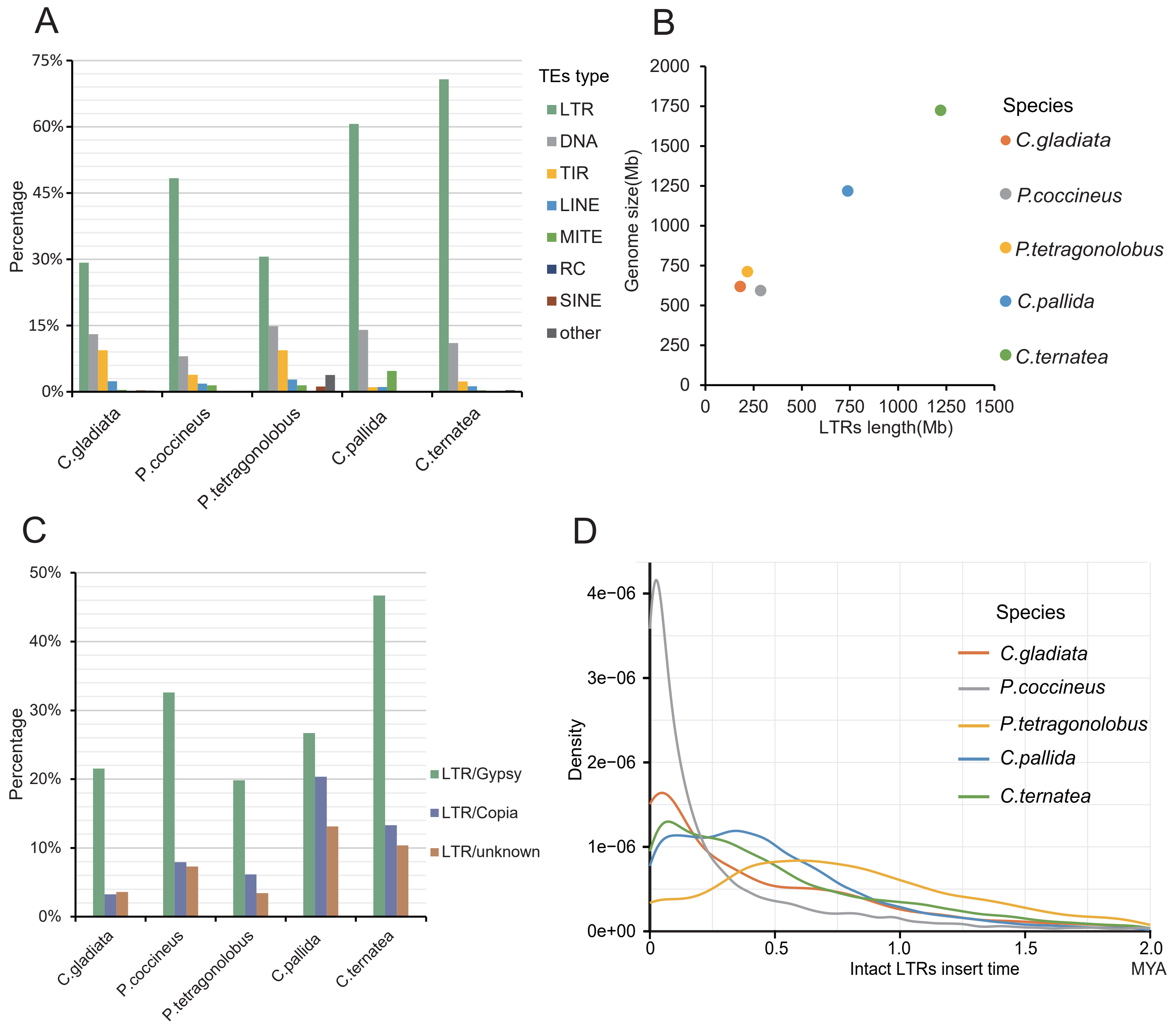

Figure 3

Click here to access/download;Figure;figure3.pdf

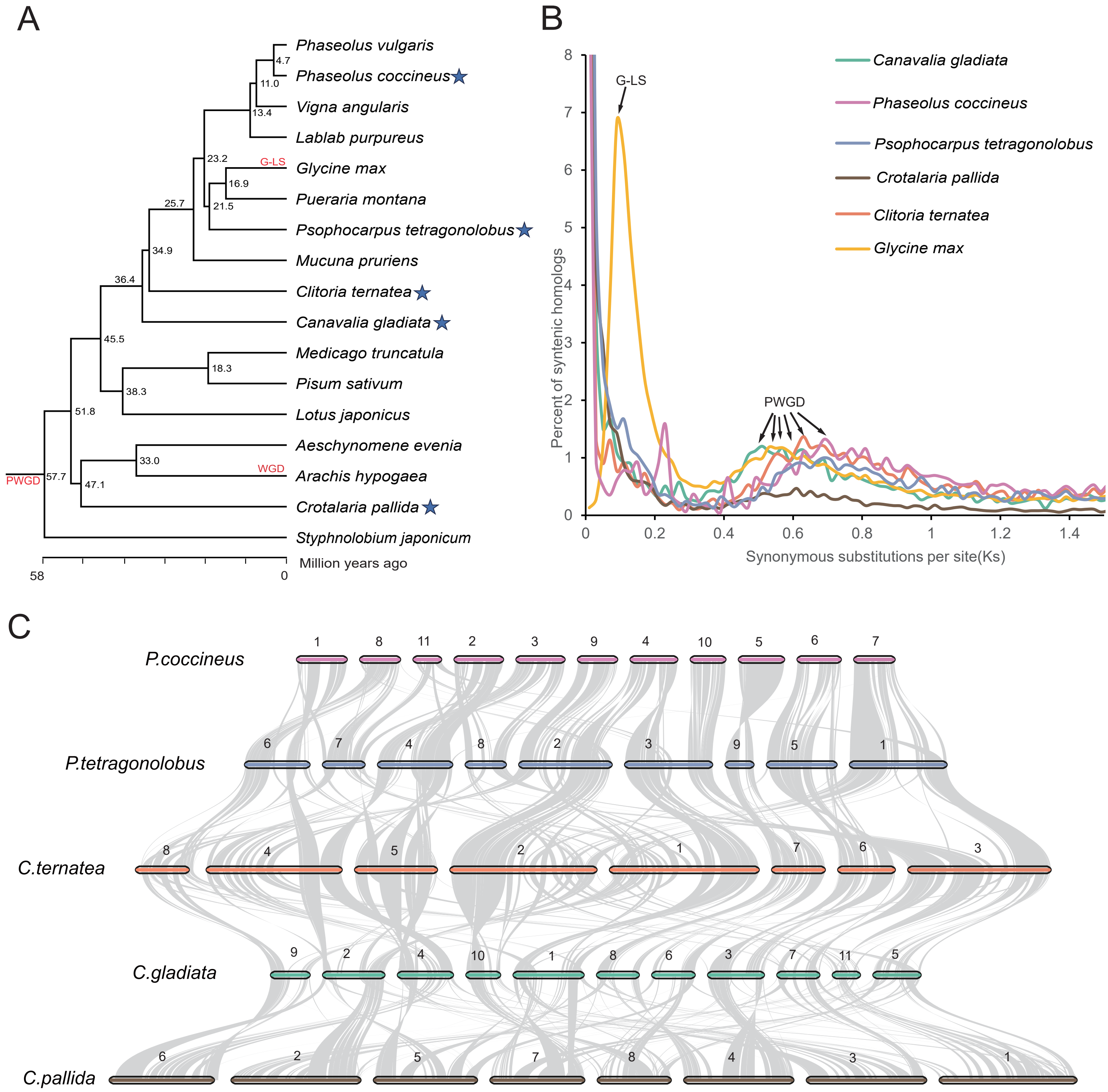

Figure 4

Click here to access/download;Figure;figure4.pdf

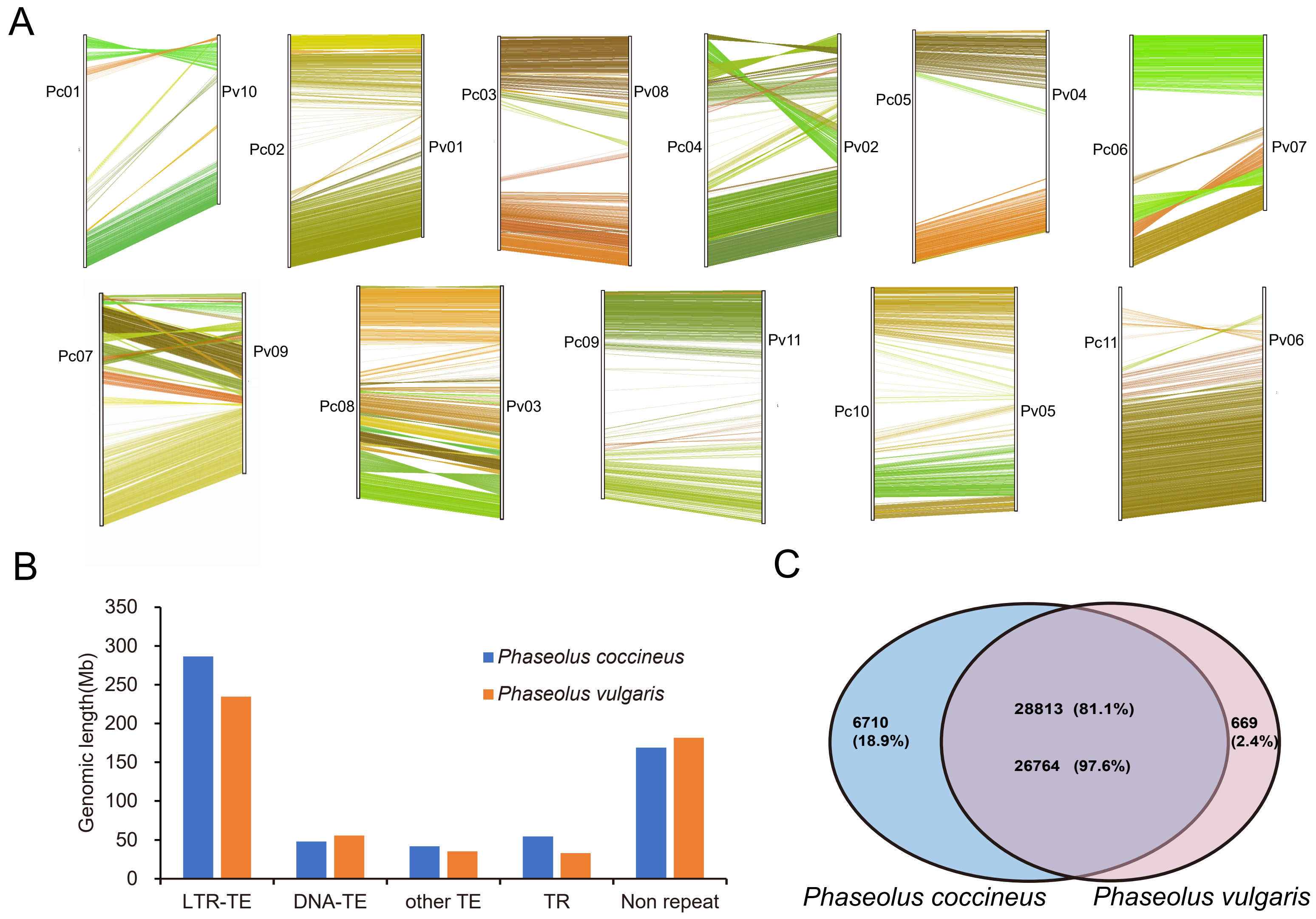

Figure 5

Click here to access/download;Figure;figure5.pdf

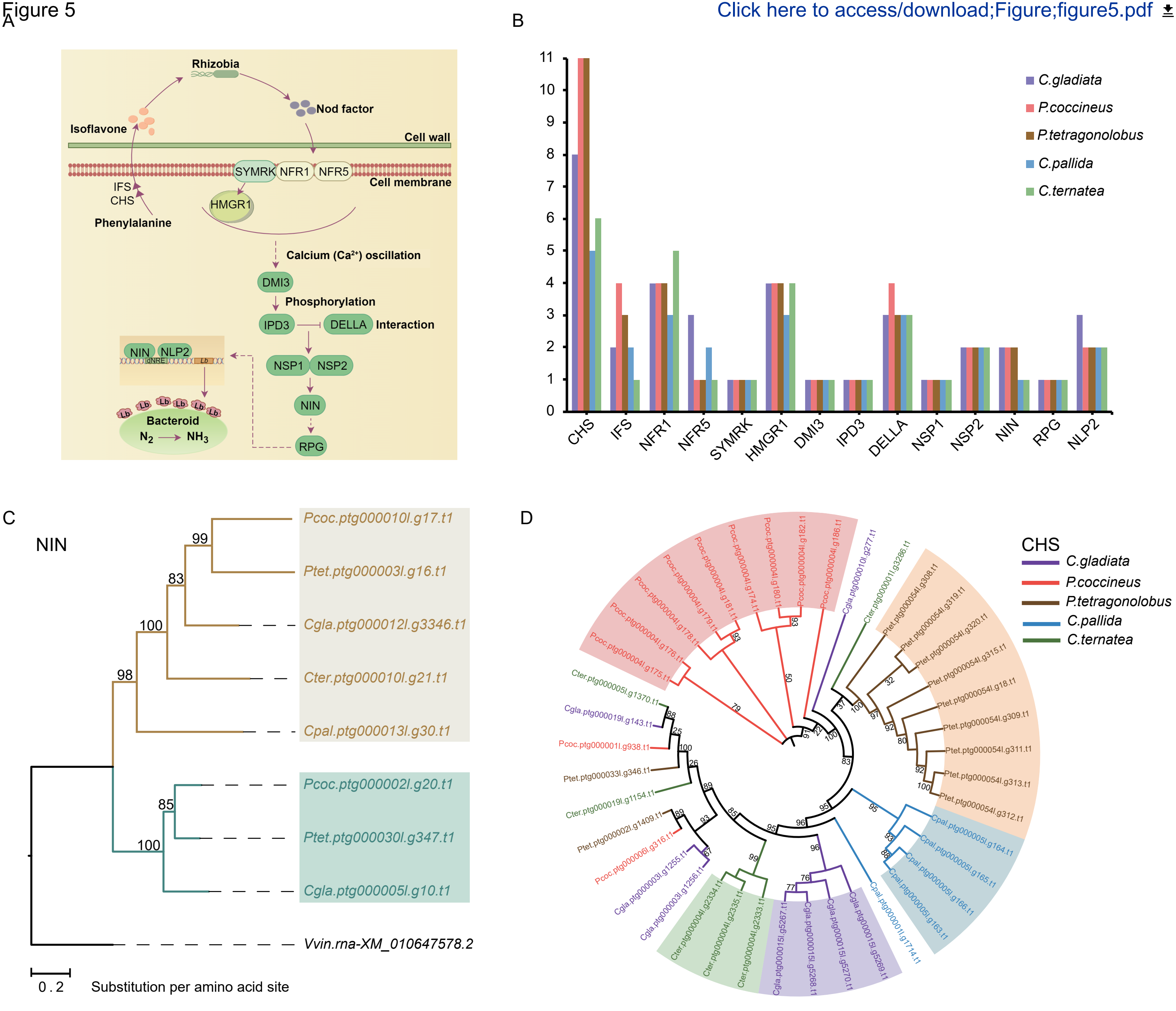

Figure 6

Click here to access/download;Figure;figure6.pdf

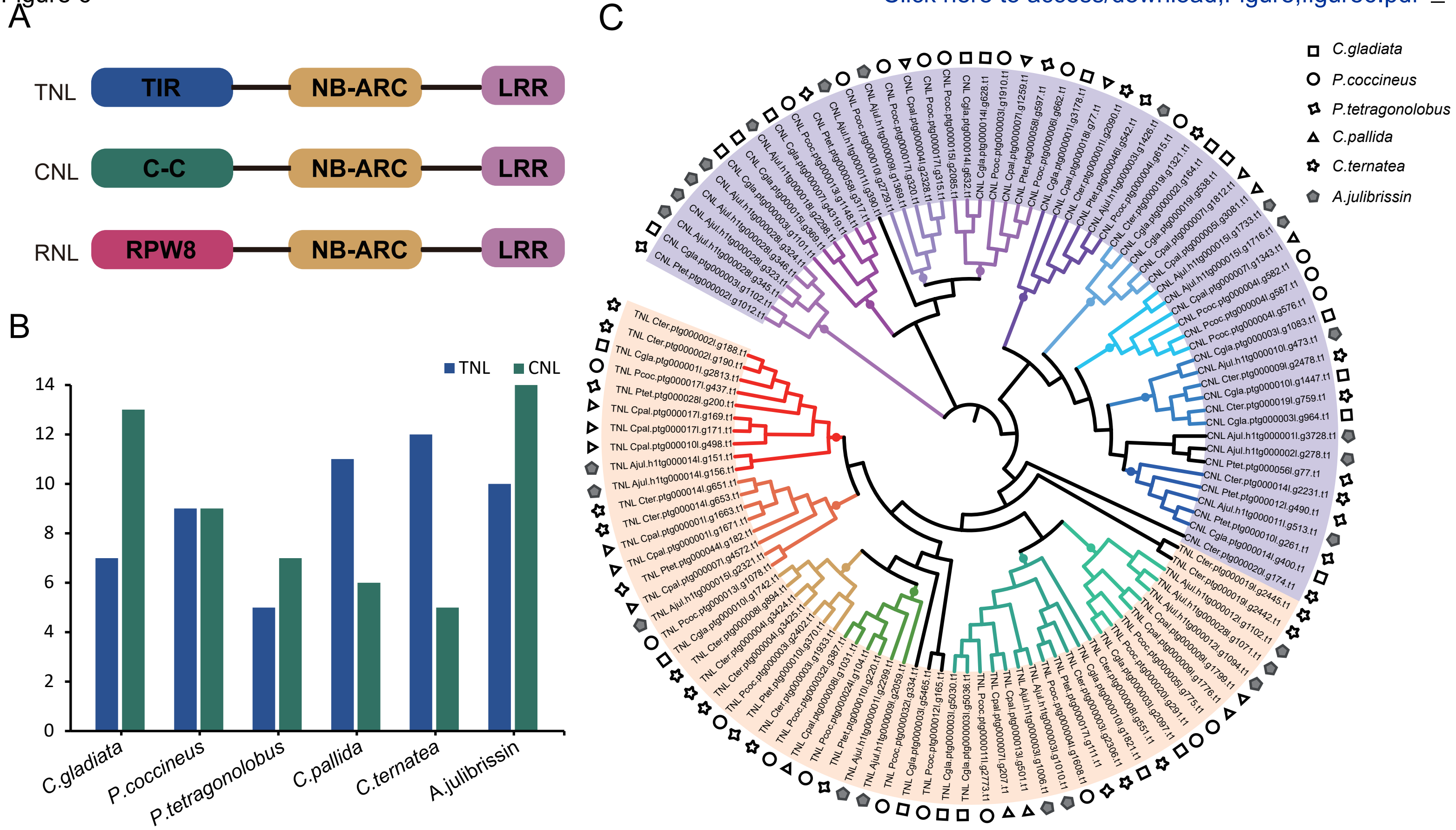

Click here to access/download
Supplementary Material
Supplementary_materials.docx
