# Supplementary material for: The genomes of 5 underutilized Papilionoideae crops provide insights into root nodulation and disease resistance
Source: Gigascience. 2024 Aug 27;13:giae063. doi: 10.1093/gigascience/giae063 (PMC11348429; doi:10.1093/gigascience/giae063)
Supplement: giae063_GIGA-D-24-00031_Revision_1 [file giae063_giga-d-24-00031_revision_1.pdf]

GigaScience

The genomes of five underutilized Papilionoideae crops provide insights into root nodulation and disease resistance

--Manuscript Draft--

**Manuscript Number:**	GIGA-D-24-00031R1
**Full Title:**	The genomes of five underutilized Papilionoideae crops provide insights into root nodulation and disease resistance
**Article Type:**	Data Note
**Funding Information:**	Shenzhen Science and Technology Innovation Program (JCYJ20190814163805604, KQTD20180411143628272) — Not applicable<br>Shenzhen Key Laboratory Fund (ZDSYS20141118170111640) — Not applicable
**Abstract:**	Background<br><br>The Papilionoideae subfamily contains a large amount of underutilized legume crops, which are important for food security and human sustainability. However, the lack of genomic resources has hindered the breeding and utilization of these crops.<br><br>Results<br><br>Here, we present chromosome-level reference genomes for five underutilized Papilionoideae crops: sword bean, scarlet runner bean, winged bean, smooth rattlebox and butterfly pea, with assembled genome sizes of 0.62, 0.59, 0.71, 1.22, 1.72 Gb, respectively. We found that long period of higher LTR activity is the major reason that enlarges the genome size of smooth rattlebox and butterfly pea, and there is no recent whole duplication (WGD) event in these 5 species except for the shared papilionoid-specific WGD event (PWGD, ~55 Mya). Then, we identified 6,710 and 669 unique genes between scarlet runner bean and common bean for each species, which may be responsible for their phenotypic differences and species-specific functions. Furthermore, we identified the key genes involved in root nodule symbiosis in all 5 species, and found that the NIN gene was duplicated in the early Papilionoideae ancestor, followed by the loss of one gene copy in smooth rattlebox and butterfly pea. At last, we identified the resistance (R) genes for plant defenses in these 5 species, and characterized their evolutionary history.<br><br>Conclusions<br><br>In summary, this study provides chromosome-scale reference genomes for three grain and vegetable beans (sword bean, scarlet runner bean, winged bean), along with genomes for a green manure crop (smooth rattlebox) and a food dyeing crop (butterfly pea). These genomes are crucial for studying phylogenetic history, unraveling nitrogen-fixing nodule symbiosis (RNS) evolution, and advancing plant defense research.
**Corresponding Author:**	Wei Fan<br>Chinese Academy of Agricultural Sciences<br>shenzhen, guangdong CHINA
**Corresponding Author Secondary Information:**	
**Corresponding Author's Institution:**	Chinese Academy of Agricultural Sciences
**Corresponding Author's Secondary Institution:**	
**First Author:**	Lihua Yuan
**First Author Secondary Information:**	

Powered by Editorial Manager® and ProduXion Manager® from Aries Systems Corporation

**Order of Authors:**	Lihua Yuan
	Lihong Lei
	Fan Jiang
	Anqi Wang
	Rong Chen
	Hengchao Wang
	Sihan Meng
	Wei Fan
**Order of Authors Secondary Information:**	
**Response to Reviewers:**	Reviewer reports:<br><br>Reviewer #1: The authors assembled five legume species' genomes, and conducted the comparisons. They searched the five genomes for genes involved in nitrogen-fixing root nodule symbiosis, phenylalanine pathway, and resistance. I got several suggestions for references.<br><br>1. Why was the study designed with the genome assembly of 5 legume crops? Why selected these five species? This study concluded on several gene identification, such as the copy loss of known NIN gene.<br>Reply: As an agriculture genomics institute, our prime goal is to provide the reference genomes for important species with agricultural value. The 5 legume crops we selected in this study are underutilized Papilionoideae crops. The high-quality reference genomes will not only benefit their breeding, but also benefit the whole researches in Papilionoideae. However, at current stage, we could not obtain more functional insights with only the genomic data. Future studies using multi-omics technologies and other technologies such as genome editing and RNA interference will generate meaningful biological results.<br><br>2. Abstract, full species names and genome ploid levels for the five species should be provided. "we identified 6,710 and 669 unique genes between scarlet runner bean and common bean for each species", I cannot understand this sentence. How were these two gene figures counted? Are these five species all diploid?<br><br>Reply: We have added full species names and genome ploid levels in the revised Abstract section: “Here, we present chromosome-level reference genomes for five underutilized diploid Papilionoideae crops: sword bean (Canavalia gladiata), scarlet runner bean (Phaseolus coccineus), winged bean (Psophocarpus tetragonolobus), smooth rattlebox (Crotalaria pallida) and butterfly pea (Clitoria ternatea)”. All these five species are diploid.<br><br>To make it clear, the sentence “"we identified 6,710 and 669 unique genes between scarlet runner bean and common bean for each species" was revised into “we identified 5,328 and 10,434 species-specific genes between scarlet runner bean and common bean for each species”. In addition, we have shown the method to determine the species-specific genes in the revised Figure 4C legend: “Overlap of the reference gene sets between P. coccineus and P. vulgaris. The protein sequences between the two species are aligned using Diamond with the parameters "--sensitive --evalue 1e-5". Genes in each species that remain unaligned are considered as species-specific genes.”.<br><br><br>3. Introduction, the latest legume genome should be cited.<br>Reply: In the revised Introduction part, we have added two latest published legume genome papers:<br>Ho, W.K., Tanzi, A.S., Sang, F. et al. A genomic toolkit for winged bean Psophocarpus tetragonolobus. Nat Commun 15, 1901 (2024). https://doi.org/10.1038/s41467-024-45048-x.<br>Sébastien Carrère, Baptiste Mayjonade, David Lalanne, Sylvain Gaillard, Jérôme Verdier, Nicolas W.G. Chen, First whole genome assembly and annotation of a

Powered by Editorial Manager® and ProduXion Manager® from Aries Systems Corporation

European common bean cultivar using PacBio HiFi and Iso-Seq data, Data in Brief, Volume 48, 2023, 109182, ISSN 2352-3409, https://doi.org/10.1016/j.dib.2023.109182.

4. "67 Gb, 70 Gb, 64 Gb, 69 Gb, and 167 Gb HiFi data", the data coverage should be provided to evalute the sequencing data size for genome assembly.
Reply: The sentence was revised into "67 Gb (103X), 70 Gb (119X), 64 Gb (93X), 69 Gb (52X), and 167 Gb (95X) HiFi data".

5. English should be polished for the whole manuscript body.
Reply: We have tried our best to polish the English in the whole manuscript.

6. "51,158, 35,523, 40,081, 48,759 and 40,267 protein-coding gene models" are from five genomes with 0.65 Gb, 0.59 Gb, 0.69 Gb, 1.33 Gb, and 1.76 Gb. Protein-coding gene number is expected to match the genome size. Why did 1.76 Gb genome contain only 40,267 genes? Is it reasonable and does it make sense? The authors should explain the rational of gene modules.
Reply: The largest genome in this study is butterfly pea (1.76 Gb), which also has the highest amount of TEs. The genome size is mostly determined by the activity of TEs. The gene number do not need to be in proportional to the genome size.

7. What are the final genome size in addition to the estimated ones? The authors did not mention them in the manuscript.
Reply: We have added the final assembled contig sizes in the revised Result part: "Then, the HiFi data were assembled into large contigs with total size 0.62 Mb, 0.59 Mb, 0.71 Gb, 1.22 Gb, and 1.72 Gb, which were further linked into 11, 11, 9, 8, 8 chromosome-scale scaffolds by Hi-C data, respectively".

8. "At last, the Lb gene was expressed to produce leghemoglobin." Is it based on the data in this study?
Reply: This sentence is based on previous studies. To make it clear, it was revised into "Then, the Lb gene was activated to produce leghemoglobin [39]". We added a reference paper for this sentence:
Wang et. al. CRISPR/Cas9 knockout of leghemoglobin genes in Lotus japonicus uncovers their synergistic roles in symbiotic nitrogen fixation. New Phytologist. 224:818–832 (2019).

9. Why do the authors claim the loss of NIN gene copies in smooth rattlebox and butterfly pea, rather than the gene duplication in scarlet runner bean and sword bean?
Reply: Our conclusion is based on the tree topology for NIN gene shown in Figure 5C. This tree suggests that the latest common ancestor of these 5 species have experienced a gene duplication event. Therefore, all the 5 species inherited two gene copies at first, then one copy lost in smooth rattlebox and butterfly pea.

10. Fig. 5 and Fig. 6, I did not observe any novel results. The comparisons among these five species cannot tell the unique gene copies, because the other legume species are not included.
Reply: Thanks for the reviewer's suggestion. We agree that Fig. 5 and Fig. 6 do not have significant scientific findings. Based on the reference genomes, we identified all the key genes involved in nitrogen-fixing root nodule symbiosis and resistance (R) gene-mediated defense for the 5 species in this study, providing a valuable resource for future functional studies. Then, we performed some simple and shallow evolutionary analyses for these genes. At current stage, we do not believe we can get solid conclusion by just adding more published legume species into the analyses, as we are lacking of accurate phenotype information. Finally, according to the editor's suggestion, we agree to change the article type of this manuscript into "Data Note".

Reviewer #2: The manuscript titled "The Genomes of Five Underutilized Papilionoideae Crops Provide Insights into Root Nodulation and Disease Resistance" describes the assembly of reference genomes for five Papilionoideae species. Although the genomic data generated in this study is relevant to advance our understanding of the genomic mechanisms that control adaptation in the five species used, I have some comments that could help to improve the manuscript before its

publication:

Page 2: Abstract
Change "whole duplication (WGD)" to "Whole Genome Duplication".
Reply: Corrected.

Change "nodule symbiosis (RNS)" to "root-nodule symbiosis".
Reply: Corrected.

Define "LTR".
Reply: “LTR” was replaced by “LTR-RTs (Long terminal repeat-retrotransposons)”.

Introduction:
Page 3: Replace subjective descriptions such as "the young bean pods are delicious vegetables" with more objective language.
Reply: this sentence was revised to “the young bean pods are used as vegetables”.

The introduction does not mention the importance of disease resistance genes. Which references have compared resistance genes in legumes, and how can this help in breeding for species of this family?
Reply: In the revised manuscript, the first paragraph of "Evolutionary History of Resistance Genes" was moved to the introduction.

How were the lines that were sequenced selected? Are they representative of a specific marker class or gene pool?
Reply: We selected the lines of highly homozygous cultivars that were widely grown in China.

Page 8: In the section "TE sequences which are too difficult," remove "too".
Reply: Corrected.

Figure 5A does not present results and should be moved to the introduction.
Reply: We agree with the reviewer that Figure 5A do not present results. However, we do not want to show any figure in the introduction. We thought that put Figure 5A and 5B-D together will help integrate information. To make it clear, we added a sentence in Figure 5A legend “The data source is from previous studies,”.

Figure 5B: Why are there differences across species? P. coccineous has double the number of CHS genes compared to C. pallida; what does this mean?
Reply: CHS is key gene for isoflavone biosynthesis. The gene number difference may influence the amount of isoflavone production in different species. However, we do not have accurate phenotype information, so we can’t make further conclusion. We added a discussing sentence in the revised manuscript “The difference in CHS gene number may influence the amount of isoflavone production in different species, which needs further investigations in future.”.

Page 9: Define "NFN".
Reply: Definition “NFN (Nitrogen-fixing Nodulation)” was provided in the revised manuscript.

Page 10: Replace "In study" with "In this study".
Reply: Corrected.

Page 11: The entire first paragraph of "Evolutionary History of Resistance Genes" should be moved to the introduction.
Reply: According to the reviewer’s suggestion, we moved this paragraph into introduction.

Page 12:
Clarify the coverage of the five reference genomes that were assembled. What constitutes "Low-coverage" for the common bean reference genome?
Confirm whether the latest reference genome was used (https://phytozome-next.jgi.doe.gov/info/Pvulgaris_v2_1), which was created using PacBio with a coverage of 83X. If the latest reference genome of the common bean was not used, the analysis

needs to be repeated.
Reply: Here the coverage means the ratio of assembled sequence in the whole genome. We have updated the analyses with the latest reference genome of the common bean, and largely revised the manuscript:

“We performed comparative genomic studies between the two Phaseolus plants scarlet runner bean (P. coccineus) and common bean (P. vulgaris) [7], which diverged from each other ~4.7 MYA (Figure 3A). Overall, all chromosomes from the two species have one-versus-one corresponding relationships, with only some intra-chromosome inversions (Figure 4A). The estimated genome sizes of the two species were both ~590 Mb. Our assembly size of scarlet runner bean is 593 Mb, almost equal to the estimated genome size. Meanwhile, the latest assembly size of common bean (NCBI: GCA_029448765.1) is 615 Mb, which is a little larger than the estimated genome size. Looking into the TEs, we found that 63.5% of the scarlet runner bean genome is composed of TEs, a little lower than that of the common bean genome (65%) (Figure 4B).

Furthermore, the 35,523 protein-coding genes of scarlet runner bean were compared to the 42,801 protein-coding genes of common bean, which was annotated by our pipeline in this study. Requiring an alignment E-value of 1e-5, 30,195 (85.0%) scarlet runner bean genes and 32,367 (75.6%) common bean genes were matched, leaving 5,328 (15.0%) unique genes in scarlet runner and 10,434 (24.4%) unique genes in common bean (Figure 4C, Supplementary Table S18), which may be responsible for the phenotypic differences and species-specific functions between the two species.
”

Modify "we found a huge advantage of HiFi-assembly" to "we found an advantage of HiFi-assembly".
Reply: In the revised manuscript, this sentence was deleted.

Discuss whether the difference in the number of unique genes between scarlet runner bean and common bean is due to sequencing advantages or actual genomic differences.
Reply: When updated the analyses with the latest assembly of common bean, we found that in the old analyses, much of the difference in the number of unique genes should be due to the sequencing advantages, but not actual genomic differences. So we removed our previous conclusion, and largely revised the manuscript.

Elaborate on how "These genomic resources will promote evolutionary and comparative genomics studies in Fabaceae".
Reply: This sentence was revised into “The genomic resources in this study expand the lineage coverage in published Fabaceae species, which will promote evolutionary and comparative genomics studies in Fabaceae.”.

On the identification of key genes in root nodulation for the five studied plants, is this not an expected finding since all are nitrogen-fixing legumes? Is there a difference in efficiency? If so, is there an enrichment of particular genes, more copies, or greater gene expression in the more efficient species?
Reply: Yes, all the sequenced 5 plants are nitrogen-fixing legumes. As we do not have accurate phenotype information on nitrogen-fixing efficiency, so we can’t make further conclusions. With the annotated genes for each species, we performed gene copies analyses, and found that sword bean, scarlet runner bean and winged bean have one more NIN gene copy than smooth rattlebox and butterfly pea. Moreover, scarlet runner bean and winged bean each have two times more copies of CHS genes than the other 3 plants. These results have been described in the Result part. As this study focus on genome level analyses, so gene expression analyses for functional genes were not performed.

Correct "was detected for all the studies species" to "was detected for all the studied species".
Reply: Corrected.

Page 13: Remove "obviously" from "We also found that the CHS genes were obviously

Powered by Editorial Manager® and ProduXion Manager® from Aries Systems Corporation

expanded through".
Reply: Corrected.

The information on R genes for the five studied plants belongs in the results section. It would be useful to compare the R genes across the five species to understand similarities and differenfces. How can this information be used to develop more resistant cultivars?
Reply: We have compared the gene number for each R gene class among the five species. However, we do not have accurate phenotype information on the disease resistance ability, so we can't make further inference between the gene copy number and disease resistance ability.
In the revised manuscript, we added a discussing sentence "Considering that the 5 species in this study were much less domesticated than those well-cultivated legumes such as soybean, the 5 studied species here still have much stronger ability of disease resistance. Therefore, the identified R genes in these 5 species may be transported to major legume crops such as soybean to improve their ability of disease resistance.".

Page 16: Correct the references for Papilionoideae species as they are incorrect in the sentence
Reply: Corrected.

Reviewer #3: The manuscript "The genomes of five underutilized Papilionoideae crops provide insights into root nodulation and disease resistance", authored by Yuan et al., presents the chromosome-length reference genomes of five crops: sword bean, scarlet runner bean, winged bean, smooth rattlebox, and butterfly pea. The manuscript is largely well-written and covers most of the analyses required for genome assembly and comparative study. However, there are a few areas that could be addressed to improve the manuscript's quality.

- Based on the Hi-C contact matrix for winged bean, it appears that the scaffolding for several chromosomes is not supported by Hi-C data. Additionally, according to Table 1, the genome assembly size for this species is higher than the estimated genome size. It would be advisable to check for contaminants in the assembly or correct the misjoins based on the Hi-C contact matrix.
Reply: For winged bean, the chromosome regions lacking Hi-C supports were repeat-rich centromeric regions. In fact, there are enough Hi-C data sequenced for these regions, but due to the requirement of unique mapping, most Hi-C data were filtered for these repeat-rich regions. Thus, they can't be shown in the Hi-C heatmap.

In Table 1, the estimated genome size by K-mer method is 689 Mb, while the assembled total contig size is 712 Mb, there is only ~23 Mb (3%) difference. Considering that the variations of the K-mer genome size estimation is about 5%, our genome size estimation generally consists with the genome assembly size . Please refer to the GCE paper:
"Estimation of genomic characteristics by analyzing k-mer frequency in de novo genome projects. https://doi.org/10.48550/arXiv.1308.2012".

According to the reviewer's suggestion, we checked the assembly again. We did not find significant contaminants in our assembly, and we could not find any obvious misjoins based on Hi-C data.

- It appears the genome assembly for the Ma3 cultivar of winged bean was recently published (https://www.nature.com/articles/s41467-024-45048-x). Comparing the with Ma3 could support assembly quality and identify differences between the cultivars.
Reply: The Ma3 cultivar assembly (586 Mb) used Oxford nanopore reads with 85%-90% single base accuracy, while our DUOXI-ginseng cultivar (712 Mb) used HiFi reads with 90-99.9% single base accuracy. In theory, HiFi data has great advantage in distinguishing repetitive sequences over Oxford nanopore reads, therefore, our assembly includes a more comprehensive assembly of the centromere sequences. To verify this thought, we downloaded the published Ma3 cultivar genome, and compared

with our DUOXI-ginseng cultivar genome. From the macrosynteny figure shown as Supplementary Fig. S4, we found that the two assembly were largely consistent in synteny, except for the middle centromere regions. The Ma3 cultivar assembly missed most of the centromere sequences, in total ~120 Mb, which explains why Ma3 cultivar (586 Mb) has a much smaller assembly size than DUOXI-ginseng cultivar (712 Mb).

However, the Ma3 cultivar assembly also has advantage over our assembly. For Chr06 in our assembly, the position of one chromosome segment seems incorrect due to the lack the Hi-C signal, we have corrected this mistake by referring to the Ma3 cultivar assembly, which has used a genetic map for scaffolding. Therefore, integrating all these genomic resources will generate a better assembly of winged bean.

We added the results of comparing analyses in the revised manuscript:
"In addition, we compared our DUOXI-ginseng cultivar assembly of winged bean (712 Mb) to the recently published Ma3 cultivar assembly (Figshare: https://doi.org/10.6084/m9.figshare.19196255) of winged bean (586 Mb), and found that our assembly has large advantage in resolving the repetitive centromere regions by using HiFi reads, while the Ma3 cultivar assembly has advantage in the chromosome-level scaffolding of contigs by using genetic map. The position of a chromosome segment in Chr06 of our assembly for winged bean was corrected according to the Ma3 cultivar assembly (Supplementary Fig. S4)."

- The genome used for the common bean seems to be outdated. More complete genomes are available, for example, Carrere et al., 2023 (https://www.sciencedirect.com/science/article/pii/S2352340923003013). The authors should utilize these newer genomes for a better comparison or provide reasoning for using the older reference genomes.
Reply: We have updated the analyses using the latest assembly of common bean. The updated results were shown in the revised manuscript:

"We performed comparative genomic studies between the two Phaseolus plants scarlet runner bean (P. coccineus) and common bean (P. vulgaris) [7], which diverged from each other ~4.7 MYA (Figure 3A). Overall, all chromosomes from the two species have one-versus-one corresponding relationships, with only some intra-chromosome inversions (Figure 4A). The estimated genome sizes of the two species were both ~590 Mb [39]. Our assembly size of scarlet runner bean is 593 Mb, almost equal to the estimated genome size. Meanwhile, the latest assembly size of common bean (NCBI: GCA_029448765.1) is 615 Mb, which is a little larger than the estimated genome size [7]. Looking into the TEs, we found that 63.5% of the scarlet runner bean genome is composed of TEs, a little lower than that of the common bean genome (65.0%) (Figure 4B, Supplementary Table S18).

Furthermore, the 35,523 protein-coding genes of scarlet runner bean were compared to the 42,801 protein-coding genes of common bean, which was annotated by our pipeline in this study. Requiring an alignment E-value of 1e-5, 30,195 (85.0%) scarlet runner bean genes and 32,367 (75.6%) common bean genes were matched, leaving 5,328 (15.0%) unique genes in scarlet runner and 10,434 (24.4%) unique genes in common bean (Figure 4C, Supplementary Table S18), which may be responsible for the phenotypic differences and species-specific functions between the two species."

- The expansion analysis is insufficient. The authors should conduct an unbiased contraction/expansion analysis using, for example, CAFE, and then identify significantly expanded/contracted gene families in the different species, rather than selectively citing gene families from the literature. This unbiased approach could help identify more gene families of interest that have not been reported before.
Reply: We have shown the CAFÉ results in Supplementary Fig. S6, which shows no genome-wide gene burst on the branches of five species in this study, indicating that there is no recent whole genome duplication like soybean.
To identify unbiased significantly expanded/contracted gene families in each species, the orthologous group results generated by OrthoFinder should be a good start. However, it is out of our interest to analyze more expanded or contracted gene families. According to the editor's suggestion, we decided to change the article type of this manuscript into "Data Note", which focuses on the primary analyses of the reference genomes.

Powered by Editorial Manager® and ProduXion Manager® from Aries Systems Corporation

	- Including the common names of all species in the figures and tables would be beneficial, as these are referred to in the text, making it easier for readers to match the text with the figures and tables.<br>Reply: We thought it is more rigorous to use latin (scientific) names in the figures and tables. We have shown both the common names and latin (scientific) names for the first time appearance in the maintext, and then to be simple, we used the common names in the maintext, as the 5 species in this study are all people familiar trees.<br><br>- In the Introduction, the citation numbers for the genomes of the different crops are incorrect.<br>Reply: Corrected.<br><br>- On page 5, in the second paragraph, including the exact numbers in the text is unnecessary as they are already in Table 1. Mentioning just the range or average numbers could be more effective.<br><br>Reply: The sentence “The coding regions cover 51 Mb (8.2%), 42 Mb (7.1%), 44 Mb (7.4%), 51 Mb (4.2%), and 42 Mb (2.4%) of the genome for each species.” was revised into “with the coding regions covering 2.4-8.2% of the genome for each species”.<br>The sentence “For function annotation, 72.1%, 89.0%, 83.9%, 87.7% and 86.8% of genes in the 5 species were annotated” was revised into “For function annotation, 72.1-89.0% of genes in the 5 species were annotated”.<br><br>- On page 7, the full form of PWGD should be provided.<br>Reply: The full form “PWGD, papilionoid-specific WGD” was provided in the revised manuscript.<br><br>- Several typographical and grammatical errors need to be corrected to enhance the manuscript's readability.<br>Reply: We have carefully checked and corrected the typographical and grammatical errors.<br><br>Editor comments:<br><br>The reviewers also mentioned (in their confidential comments to editor) that the story is not that attractive for the presence and minor copy variations of all the related genes, and the manuscript is more suitable for the GigaScience Data Note, rather than the regular research article. Therefore, we suggest you change the article type from "Research" to "Data Note".<br>Reply: We agree to change the article type into “Data Note”.
**Additional Information:**	
**Question**	**Response**
Are you submitting this manuscript to a special series or article collection?	No
**Experimental design and statistics**<br><br>Full details of the experimental design and statistical methods used should be given in the Methods section, as detailed in our Minimum Standards Reporting Checklist. Information essential to interpreting the data presented should be made available in the figure legends.	Yes

Have you included all the information requested in your manuscript?	
**Resources**<br><br>A description of all resources used, including antibodies, cell lines, animals and software tools, with enough information to allow them to be uniquely identified, should be included in the Methods section. Authors are strongly encouraged to cite Research Resource Identifiers (RRIDs) for antibodies, model organisms and tools, where possible.<br><br>Have you included the information requested as detailed in our Minimum Standards Reporting Checklist?	Yes
**Availability of data and materials**<br><br>All datasets and code on which the conclusions of the paper rely must be either included in your submission or deposited in publicly available repositories (where available and ethically appropriate), referencing such data using a unique identifier in the references and in the "Availability of Data and Materials" section of your manuscript.<br><br>Have you have met the above requirement as detailed in our Minimum Standards Reporting Checklist?	Yes

Manuscript
Click here to access/download;Manuscript;manuscript_R1.docx

Title

The genomes of five underutilized Papilionoideae crops provide insights into root nodulation and disease resistance

Lihua Yuan[1,2,3,4], Lihong Lei[1,2,3,4], Fan Jiang[1,4], Anqi Wang[1], Rong Chen[1], Hengchao Wang[1], Sihan Meng[1], Wei Fan[1,*]

[1]Guangdong Laboratory for Lingnan Modern Agriculture (Shenzhen Branch), Genome Analysis Laboratory of the Ministry of Agriculture and Rural Affairs, Agricultural Genomics Institute at Shenzhen, Chinese Academy of Agricultural Sciences, Shenzhen, Guangdong, 518120, China

[2]State Key Laboratory of Crop Stress Adaptation and Improvement, School of Life Sciences, Henan University, Kaifeng 475004, China

[3]Shenzhen Research Institute of Henan University, Shenzhen 518000, China

[4]These authors contributed equally to this article.

*Correspondence author: Wei Fan (fanwei@caas.cn)

Abstract

Background: The Papilionoideae subfamily contains a large amount of underutilized legume crops, which are important for food security and human sustainability. However, the lack of genomic resources has hindered the breeding and utilization of these crops.

Results: Here, we present chromosome-level reference genomes for five underutilized diploid Papilionoideae crops: sword bean (*Canavalia gladiata*), scarlet runner bean (*Phaseolus coccineus*), winged bean (*Psophocarpus tetragonolobus*), smooth rattlebox (*Crotalaria pallida*) and butterfly pea (*Clitoria ternatea*), with assembled genome sizes of 0.62, 0.59, 0.71, 1.22, 1.72 Gb, respectively. We found that long period of higher LTR-RTs (Long terminal repeat-retrotransposons) activity is the major reason that enlarges the genome size of smooth rattlebox and butterfly pea.~~,~~ Additionally,~~and~~ there have been ~~is~~ no recent W~~w~~hole Genome D~~d~~uplication (WGD) events in these 5 species except for the shared papilionoid-specific WGD event (PWGD, ~55 ~~Mya~~MYA). Then, we identified 5,328 and 10,434~~6,710 and 669~~ species-specific ~~unique~~ genes between scarlet runner bean and common bean, ~~for each species~~reapectively, which may be responsible for their phenotypic and functional differences~~ and species-specific functions~~. Furthermore, we identified the key genes involved in root~~-~~nodule symbiosis (RNS) in all 5 species, and found that the *NIN* gene was duplicated in the early Papilionoideae ancestor, followed by the loss of one gene copy in smooth rattlebox and butterfly pea lineages. At last, we identified the resistance (R) genes for plant defenses in these 5 species, and characterized their evolutionary history.

Conclusions: In summary, this study provides chromosome-scale reference genomes for three grain and vegetable beans (sword bean, scarlet runner bean, winged bean), along with genomes for a green manure crop (smooth rattlebox) and a food dyeing crop (butterfly pea). These genomes are

crucial for studying phylogenetic history, unraveling nitrogen-fixing root-nodule symbiosis (RNS) evolution, and advancing plant defense research.

Key-words: Papilionoideae, underutilized legume, whole genome duplication, root nodule symbiosis, R genes

Introduction

Papilionoideae, the largest subfamily in Fabaceae (Legume) [1] whose name probably originated from its flower's resemblance to a butterfly (Latin: Papilio), has an extremely important position in agriculture and makes great contributions to the human diet and food security. In addition to~~Besides,~~ the several well-known crops such as soybean [2], peanut [3], faba bean [4], mung bean [5], pea [6], common bean [7]and alfalfa [8], this subfamily also includes many other underutilized crops [9]. For example, sword bean (*Canavalia gladiata*), scarlet runner bean (*Phaseolus coccineus*) and winged bean (*Psophocarpus tetragonolobus*) are both grain and vegetable plants: the mature bean seeds are protein-rich grains, while the young bean pods are ~~delicious~~ used as vegetables. Smooth rattlebox (*Crotalaria pallida*) and butterfly pea (*Clitoria ternatea*) are often used as green manure and forage grass, due to their high protein content. The dried flowers of butterfly pea are also used as a natural food colorant (blue), which is popular in Southeast Aisa countries [10]. In addition, sword bean has also been used as a traditional medicine to improve poor appetite and alleviate vomiting in China for thousands of years [11], and scientists found that smooth rattlebox also has antitumor properties in recent years [12].

The Papilionoideae plants also play a unique ecological role in nitrogen fixation through

symbiotic root nodules [13], which is indispensable for the global nitrogen cycle. The ability to nitrogen fixation from the atmosphere also helps agriculture production use fewer synthetic fertilizers, thereby reducing ~~the -~~ energy consumption and mitigating soil pollution [14]. As a model of special host-bacteria interaction, the formation of nitrogen-fixing root nodules has been intensively studied on two model species *Medicago truncatula* and *Lotus japonicus* [15, 16], both belonging to the subfamily Papilionoideae. The host plants excrete flavonoids into the rhizosphere, and induce the rhizobia to express the nodulation (nod) genes [13]. Then, the metabolite products of these nod genes (Nod factors) are sensed by the host plants to start nodulation, which requires the coordination of rhizobial infection at the root epidermis with cell division in the cortex [17]. Inside the nodule, rhizobia live in an organelle-like structure known as symbiosome, and the host plants secrete leghemoglobin (Lb) to maintain a low-oxygen environment in order to facilitate the nitrogen-fixing reactions in symbiosomes [18]. Recent phylogenomics and phylotranscriptomics studies have shown a single origin of nitrogen-fixing root-~~-~~nodule symbiosis (RNS), and then multiple independent losses occurred in various lineages [19, 20].

Resistance (R) gene-mediated defense plays an important role in plant defenses against all pathogens. It recognizes the pathogen-derived proteins referred to as effectors, and induces a state in the host defined as effector-triggered susceptibility (ETS), which in turn leads to the local hypersensitive response (HR) cell death to restrict pathogen growth and propagation [42]. Most cloned R genes encode intracellular NLR receptors, which are typically composed of 3 domains: a central NB (NB-ARC) domain, bordered by a C-terminal leucine-rich repeat domain (LRR), and an N-terminal coiled-coil (CC) or Toll/ interleukin-1 receptor (TIR) or resistance to powdery mildew (RPW8) domain [35]. The RPW8 domain is rare in comparison to the two major CC and TIR domains.

For the purpose of nodulation studies and crop breeding, tens of agriculturally important plants in the subfamily Papilionoideae have been sequenced, including all the above well-known species, as well as adzuki bean [21], lablab bean [22], velvet bean [23], kudzu vine [24], pagoda tree [25], and winged bean (Ma3 cultivar) [26] et al.. However, the subfamily contains many other rare but valuable species, which still lack reference genomes, hindering the in-depth biological studies and exploitation of these species. Here, we present the chromosome-scale reference genomes for 3 grain and vegetable beans (sword bean, scarlet runner bean, winged bean), a green manure crop (smooth rattlebox), and a food dyeing crop (butterfly pea) to investigate the phylogenetic history, explore the evolution of RNS, and identify the resistance (R) genes involved in controlling crop diseases.

Results

Chromosome-scale assembly of 5 underutilized legumes

We generated 67 Gb (103X), 70 Gb (119X), 64 Gb (93X), 69 Gb (52X), and 167 Gb (95X) HiFi data for sword bean, scarlet runner bean, winged bean, smooth rattlebox, and butterfly pea, respectively (Supplementary Table S1). Analyzing the distribution of K-mer frequencies [27], we found that all ~~the~~ 5 sequencing materials are highly homozygous and the estimated genome size is 0.65 Gb, 0.59 Gb, 0.69 Gb, 1.33 Gb, and 1.76 Gb for each species (Supplementary Fig. S1). Then, the HiFi data were assembled into large contigs with total size 0.62 Mb, 0.59 Mb, 0.71 Gb, 1.22 Gb, and 1.72 Gb, which were further linked into 11, 11, 9, 8, 8 chromosome-scale scaffolds by Hi-C data, respectively (Supplementary Table S2-S4, Supplementary Fig. S2). Overall, most chromosomes include less than 5 contigs, suggesting a very high continuity of our assembly

(Supplementary Fig. S3). Besides, the BUSCO (Benchmarking Universal Single-Copy Orthologs) complete ratio is over 99% and the QV value calculated by Merqury version 1.3 [28] is over 70 for all the 5 species, indicating the very high accuracy of our assembly (Table 1, Supplementary Table S5-S6).

In addition, we compared our DUOXI-ginseng cultivar assembly of winged bean (712 Mb) to the recently published Ma3 cultivar assembly (Figshare: https://doi.org/10.6084/m9.figshare.19196255) of winged bean (586 Mb), and found that our assembly has large advantage in resolving the repetitive centromere regions by using HiFi reads, while the Ma3 cultivar assembly has advantage in the chromosome-level scaffolding of contigs by using genetic map. The position of a chromosome segment in Chr06 of our assembly for winged bean was corrected according to the Ma3 cultivar assembly (Supplementary Fig. S4).

In total, 51,158, 35,523, 40,081, 48,759 and 40,267 protein-coding gene models were predicted in the genome of sword bean, scarlet runner bean, winged bean, smooth rattlebox, and butterfly pea, respectively (Figure 1, Supplementary Table S7-S9) , with~~.~~ t~~T~~he coding regions covering 2.4-8.2% of the genome for each species~~ 51 Mb (8.2%), 42 Mb (7.1%), 44 Mb (7.4%), 51 Mb (4.2%), and 42 Mb (2.4%) of the genome for each species~~. The BUSCO complete rates for the gene sets of these 5 species are comparable to those of ~~BUSCO complete rates for~~ the genomes, suggesting a high completeness of our gene annotation (Table 1). For function annotation, 72.1~~1%, ~~ 89.0%~~,~~ 83.9%~~, 87.7% and 86.8%~~ of genes in the 5 species were annotated by at least one of the NCBI-NR, KEGG, InterPro or GO databases (Supplementary Table S10). In addition, we identified 970, 1,141, 1,283, 1,382 and 2,307 tRNA genes, and 1,535, 5,030, 3,020, 6,268 and 3,158 rRNA genes for the five species (Supplementary Table S11).

LTR-RTs activity influences the genome size

The highly continuous reference genomes enabled a comprehensive analysis of the transposable elements (TEs). In total, 55%, 63%, 64%, 82% and 86% of the genomes are composed of TEs for sword bean, scarlet runner bean, winged bean, smooth rattlebox, and butterfly pea, respectively (Figure 2A, Supplementary Table S12). Among all the TE types, LTR-RTs especially Gypsy-LRT is the most dominant TE type for all the 5 species. Notably, LTR-RTs activity is also the most contributing factor to the genome size (Figure 2B-2C, Supplementary Table S13-S14), which is consistent with previous reports for most plants [29]. The LTR-RTs expansion period in smooth rattlebox and butterfly pea are much wider than the other 3 species, which may partially explain their relatively larger genome sizes. Interestingly, there is a very recent sharp explosion of LTR-RTs in scarlet runner bean, though its LTR-RTs activity is much lower in the long history period. On the contrary, there is a high LTR-RTs expansion in the old history period, but the LTR-RTs activity gets lower and lower in the recent history period in winged bean (Figure 2D). Taken together, these results suggest that the genome size of legume species have been changing in the evolution history along with the LTR-RTs expansions and extractions.

No recent whole genome duplication was found in the five legumes

To study the evolution of Papilionoideae, the reference genes of sword bean, scarlet runner bean, winged bean, smooth rattlebox, butterfly pea, and 12 published Papilionoideae species, including *Phaseolus vulgaris* [30], *Vigna angularis* [21], *Lablab purpureus* [22], *Glycine max* [31],

Pueraria montana [24], *Mucuna pruriens* [23], *Pisum sativum* [6], *Medicago truncatula* [32], *Lotus japonicus* [33], *Aeschynomene eveni* [34], *Arachis hypogaea* [35] and *Styphnolobium japonicum* [36] (Supplementary Table S15-S16), were clustered into 35,057 orthologous groups (orthogroups), with each orthogroup containing at least two genes. *Vitis vinifera* [37] was used as an outgroup. Then, the 405 single-copy orthogroups were used for phylogeny construction and divergence time estimation (Supplementary Fig. S5~~4~~). The winged bean, scarlet runner bean, butterfly pea, sword bean and smooth rattlebox diverged from soybean (*Glycine max*) at 21.5 ~~Mya~~MYA, 23.2 ~~Mya~~MYA, 34.9 ~~Mya~~MYA, 36.4 MYA~~Mya~~ and 51.8 ~~Mya~~MYA, respectively (Figure 3A).

To investigate the whole genome duplication events, we calculated the Ks values of the paralogue pairs for each species. The distribution of Ks values showed a shared peak at around 0.6 for all the 5 species in this study as well as soybean (Figure 3B), consistent with previous reports that an ancient whole genome duplication event (PWGD, papilionoid-specific WGD) occurred at the origin of the papilionoid clade 55 ~~Mya~~ MYA [38]. The large amounts of whole genome-wide syntenic fragments inside each species also confirms this inference (Supplementary Fig. S6~~5-6~~, Supplementary Table S17). Unlike soybean which has a lineage-specific whole genome duplication event (G-LS) 13 MYA~~ya~~ corresponding to Ks peak around 0.1 [2], all the 5 species in this study do not have any recent Ks peaks, indicating that they do not have recent whole genome duplication events. The gene expansion and contraction analyses along the phylogeny tree also showed no recent genome-wide gene burst on the branches for all the 5 species in this study (Supplementary Fig. S7) Although the chromosome numbers have changed among the 5 species,

many large syntenic blocks ~~were~~ are still present~~existent~~, ~~but~~ with multiple large-scale chromosome inversion and translocation events (Figure 3C).

Unique genes identified between *P. coccineus* and *P. vulgaris*

We performed comparative genomic studies between the two *Phaseolus* plants scarlet runner bean (*P. coccineus*) and common bean (*P. vulgaris*) [7], which diverged from each other ~4.7 ~~Mya~~ MYA (Figure 3A). Overall, all chromosomes from the two species have one-versus-one corresponding relationships, with only some intra-chromosome inversions (Figure 4A). The estimated genome sizes of the two species were both ~590 Mb. Our assembly size of scarlet runner bean is 593 Mb, almost equal to the estimated genome size. Meanwhile, the latest assembly size of common bean (NCBI: GCA_029448765.1) is 615 Mb, which is a little larger than the estimated genome size. Looking into the TEs, we found that 63.5% of the scarlet runner bean genome is composed of TEs, a little lower than that of the common bean genome (65%) (Figure 4B, Supplementary Table S18).~~Our assembly size of scarlet runner bean with HiFi reads is 593 Mb almost equal to the estimated genome size, however, the assembly size of common bean with Roche/Illumina data is only 532 Mb, missing ~58 Mb (10%) sequences. Looking into the TEs, we found that scarlet runner bean has 52 Mb more LTR-TEs than common bean which largely explains the missing components in the assembly of common bean. Being close to scarlet runner bean, common bean is also very likely to have a recent explosion of LTR-TEs (Figure 2D), resulting in many highly similar copies of TE sequences which are too difficult to assemble using short reads data. In contrast, our assembly of scarlet runner bean with ultralong HiFi data can successfully overcome this problem.~~

Furthermore, the 35,523 protein-coding genes of scarlet runner bean were compared to the 42,801 protein-coding genes of common bean, which was annotated by our pipeline in this study.the better assembly of scarlet runner bean enables an annotation of 35,523 genes, much higher than the 27,433 genes identified in common bean. Requiring an alignment E-value of 1e-5, 28,813 30,195 (85.01.1%) scarlet runner bean genes and 32,36726,764 (75.697.6%) common bean genes were matched, leaving 5,3286,710 (18.915.0%) unique genes in scarlet runner and 10,434669 (2.424.4%) unique genes in common bean (Figure 4C, Supplementary Table S18), which may be responsible for the phenotypic differences and species-specific functions between the two species. Due to the poor assembly, common bean should in fact have more unique genes, which may be approximate to the number of unique genes in scarlet runner bean.

Expansion of *NIN* and *CHS* genes

Legume is special in plants for its nitrogen-fixing root--nodule symbiosis (RNS) [13], which requires a set of key genes (Figure 5A). The chalcone synthase (CHS) and isoflavone synthase (IFS) are responsible for the biosynthesis of isoflavone, which attracts Rhizobia. NFR1 and NFR5 receptors perceive the Nod factors secreted from Rhizobia, and interact with downstream SYMBIOSIS RECEPTOR-LIKE KINASE (SYMRK), who further activates 3-hydroxy-3-methylglutaryl-CoA reductase (HMGR1) that induce nuclear calcium oscillations. DMI3 (DOES NOT MAKE INFECTIONS 3) detects the calcium signal, and activates IPD3 (Protein CYCLOPS) and DELLA, which further induces downstream transcription factors NSP1 (Nodule Signaling Protein 1), NSP2 (Nodule Signaling Protein 2), NIN (NODULE INCEPTION), NLP2 (NIN-like

protein 2), as well as RPG (Rhizobiumdirected polar growth). ~~At last~~Then, the *Lb* gene was ~~expressed~~ activated to produce leghemoglobin [39]. We identified all these key genes in sword bean, scarlet runner bean, winged bean, smooth rattlebox, and butterfly pea (Figure 5B, Supplementary Table S19-S20), providing a valuable gene resource for RNS studies.

In contrast to other symbiosis-relevant genes involved in infection, NIN and RPG are only known to have NFN (Nitrogen-fixing Nodulation) symbiosis~~-~~specific functions, whereas the mutation of other genes may have more pleiotropic effects. The phylogenomics studies also found that both the *NIN* and *RPG* genes exist in root nodulating legumes, but ~~absence~~are absent or have bec~~a~~ome pseudogenes in non-nodulating legumes, indicating that *NIN* and *RPG* are the essential genes for root nodulation [19]. In this study, we found that the *RPG* gene was single copy in each plant (Supplementary Fig. S8~~7~~), but the *NIN* gene was duplicated in the early Papilionoideae ancestor, possibly as a result of the whole genome duplication event (PWGD) occurred 55 ~~Mya~~MYA. One gene copy was retained in all ~~the~~ 5 plants, but the other gene copy was missing in smooth rattlebox and butterfly pea (Figure 5C, Supplementary Table S21). Therefore, sword bean, scarlet runner bean and winged bean, each have two copies of the *NIN* genes, while smooth rattlebox and butterfly pea have only one copy of the *NIN* gene for each plant.

Isoflavones, a type of polyphenolic secondary metabolite from the phenylalanine pathway in plants with a C6-C3-C6 structure, are predominantly distributed in plants of the Papilionoideae subfamily and are majorly used as the signaling molecules between leguminous plants and rhizobia [40, 41]. In this study, we identified all the gene copies of *CHS* and *IFS* (Figure 5D,

Supplementary Fig. S98, and Supplementary Table S22), which are the key genes responsible for isoflavone biosynthesis. Interestingly, scarlet runner bean and winged bean each have 11 copies of *CHS* genes, almost two times of that in the other 3 plants. From the phylogenetic analysis, we found that the expansion of *CHS* genes in scarlet runner bean and winged bean was species-specific. Most of the duplicated *CHS* genes are located in a small cluster region on the same chromosome, indicating that the gene expansion occurred through local tandem gene duplications (Figure 5D). The difference in *CHS* gene number may influence the amount of isoflavone production in different species, which needs further investigations in future.

Evolutionary history of Resistance genes

Resistance (R) gene-mediated defense plays an important role in plant defenses against all pathogens. It recognizes the pathogen-derived proteins referred to as effectors, and induces a state in the host defined as effector-triggered susceptibility (ETS), which in turn leads to the local hypersensitive response (HR) cell death to restrict pathogen growth and propagation [42]. Most cloned R genes encode intracellular NLR receptors, which are typically composed of 3 domains: a central NB (NB-ARC) domain, bordered by a C-terminal leucine-rich repeat domain (LRR), and an N-terminal coiled-coil (CC) or Toll/ interleukin-1 receptor (TIR) or resistance to powdery mildew (RPW8) domain [35]. The RPW8 domain is rare in comparison to the two major CC and TIR domains. Based on the type of N-terminal domain, the R genes were traditionally divided into 3 classes: TNL (TIR-NB-LRR), CNL (CC-NB-LRR), and RNL (RPW8-NB-LRR) (Figure 6A)..

In this study, we identified 7, 9, 5, 11, 12 TNL genes, and 13, 9, 7, 6, 5 CNL genes, for sword bean,

Formatted: Indent: First line: 0"

scarlet runner bean, winged bean, smooth rattlebox, and butterfly pea, respectively (Figure 6B, Supplementary Fig. S9). In addition, only one R gene with the N-terminal RPW8 domain was identified in smooth rattlebox (Supplementary Fig. S109). Overall, scarlet runner bean has equal number of TNL and CNL genes, sword bean and winged bean have more CNL genes than TNL genes, while smooth rattlebox and butterfly pea have more TNL genes than CNL genes. Through phylogenetic analysis, all the TNL and CNL genes were separated into two major branches (Figure 6C), consistent with previous studies which shown that the three classes of NLR R-genes have evolved at the early origin of ancestral Angiosperm [43]. Using *Albizia julibrissin* as an outgroup, which belongs to the second largest subfamily Caesalpinioideae in Fabaceae (Legume), we inferred 6 TNL orthologous groups (OGs) and 9 CNL OGs within the family Fabaceae, with each OG derived from a single gene in the common Fabaceae ancestor (Figure 6C). The 6 TNL and 9 CNL ancestral genes in Fabaceae, were derived from multiple whole genome polyploidization events or gene duplications since the born of Angiosperm. Considering that the 5 species in this study were much less domesticated than those well-cultivated legumes such as soybean, the 5 studied species here still have a much stronger ability of disease resistance. Therefore, the identified R genes in these 5 species may be transported to major legume crops such as soybean to improve their ability of disease resistance.

Discussion

In this study, we generated chromosome-level genome assemblies and high-quality gene annotations for 5 underutilized legume crops. The assembly quality is near telomere-to-telomere level, with only a few gaps in each constructed chromosome. Smooth rattlebox and butterfly pea have much larger genome sizes than sword bean, scarlet runner bean and winged bean, due to a

relatively long period of LTR expansion in the evolutionary history of these two species. Phylogeny and divergence time for the studied plants were inferred with the single-copy gene families, and a whole genome duplication event at the origin of the papilionoid clade (PWGD) around 55 Mya MYA was detected for all the studieds species. Moreover, we By comparing the HiFi-assembled scarlet runner bean and the low-coverage assembly of common bean derived from Roche/Illuimna data, we found huge advantage of HiFi-assembly in overcoming the difficulties of recently burst repetitive sequences, and identified 6,7105,328 unique genes in scarlet runner bean and only 10,434669 unique gene in common bean, which may be helpful in investigating the functional genes underling their phenotypic differences.. These genomic resources in this study expand the lineage coverage in published Fabaceae species, which will promote evolutionary and comparative genomics studies in Fabaceae.

In comparison to the grains, most beans have much higher protein content, which is closely related with nitrogen-fixing root nodule symbiosis. Based on sequence homology, we identified all the key genes involved in root nodulation in the 5 studied plants. The *NIN* gene was duplicated in the early Papilionoideae ancestor, and then gene loss happened on one branch in smooth rattlebox and butterfly pea. We also found that the *CHS* genes were obviously expanded through local tandem duplication in scarlet runner bean and winged bean. Our results provide more genomic evidence for the evolution of nitrogen-fixing root nodule symbiosis (RNS), which will promote the molecular breeding of more efficient RNS cultivar and benefit the utilization of global nitrogen-fixing by legume plants.

Improvement of disease resistance in crops has great potential to increase productivity. Huge

losses caused by pathogenic fungi, bacteria, nematodes, oomycetes, and viruses could be mitigated by breeding of disease-resistant cultivars. The 5 underutilized legume crops in this study are much less human-selected than other well-known legume crops such as soybean, thus, they may include more powerful resistance (R) genes. In this study, we identified all the R genes in the 5 studied plants, which can be divided mainly into two classes TNL and CNL. Notably, sword bean and winged bean have more CNL genes, but smooth rattlebox and butterfly pea have more TNL genes. In future, these R genes can be transferred into major legume crops such as soybean to improve its disease resistance, which will reduce the application of chemical pesticide and promote the global food security.

Methods

Plant materials and sequencing

Commercial seed of sword bean (LVBAO cultivar) was obtained from NONGZHIZI SEEDS company (ZhuZhou, Hunan, China). Commercial seed of scarlet runner bean (Climbing cultivar) was obtained from JINMINXINNONG company (Fuzhou, Fujian, China). Commercial seed of winged bean (DUOXI-ginseng cultivar) was obtained from BOSITE agricultural technology company (Shenyang, Liaoning, China). Commercial seed of smooth rattlebox (Three-ellipse-leaf cultivar) was obtained from the forestry-bureau permitted seed store company (Jiaxing, Zhejiang, China). The young seedling of butterfly pea (Blue-flower cultivar) was obtained from LIUYI flowers and fruit seedlings company. The seeds were grown in plant growth chamber, and young leaves from a single plant for each species were used to extract genomic DNA using the Hi-DNAsecure Plant Kit (TIANGEN DP350, China). The genomic DNA was used to prepare 20-kb

inserts sequencing library by SMRTbell Express Template Prep Kit 2.0 (PacBio, USA), and sequenced on Sequel II with the HiFi mode (PacBio, USA). The young leaves from the same plant were also used for Hi-C sequencing on the Illumina NovaSeq 6000 in PE150 mode. The roots, stems, leaves, and flowers of each species were sampled to extract total RNA using the RNeasy Plant Mini Kit (QIAGEN, Germany). The extracted RNA was pooled together for full-length cDNA sequencing on PacBio Sequel II with Iso-Seq mode (PacBio, USA).

Genome assembly and annotation

The contigs for sword bean, scarlet runner bean, and winged bean were assembled from PacBio HiFi reads utilizing HIFIASM version 0.16.1 [44] with parameter “-l 0”, and the contigs for smooth rattlebox, and butterfly pea were assembled from PacBio HiFi reads utilizing HIFIASM version 0.19.5 [44] with parameter “-l 0”. Contaminations were removed by aligning all contigs to chloroplast and mitochondria sequences downloaded from the NCBI database, using MINIMAP2 version 2.20 [45] with an identity >0.95 and coverage >0.95. The remaining contigs were used to represent the nuclear contigs, and the completeness was evaluated using BUSCO version 5.1.2 [46] with ORTHODB version 10 (embryophyta lineage). Ultimately, the Hi-C reads were aligned to the nuclear contigs and Hi-C contact matrices among the contig bins were generated utilizing HIC-PRO version 3.1.0 [47]. Utilizing the Hi-C linkage information between contig ends, nuclear contigs (with sizes exceeding 1 Mb) were assembled into scaffolds at the chromosome level utilizing ENDHIC version 1.0 [48].

Tandem repeat elements (TRs) were detected using Tandem Repeats Finder (TRF) version 4.09 [49]. Interspersed repeat elements (TEs) were identified through a three-step process: (1) The

prediction of structurally intact transposon elements (TEs), including long-terminal-repeat retrotransposons (LTR-RTs), DNA transposon, Helitron, etc., was accomplished using EDTA version 1.9.9 [50]. Concurrently, an intact TE library was generated. (2) Incomplete and homology TEs were detected against the above-mentioned intact TE library, Repbase database version 26.05 (plant lineage), and the Protein-coding TE database using REPEATMASKER version 4.1.2 (http://www.repeatmasker.org). (3) A de novo TE library was generated from the masked genome with all the above-mentioned identified TEs, using REPEATMODELER version 2.0.1 (http://www.repeatmasker.org/RepeatModeler), then the TE sequences in the library were classified using TERL v1.0 [51]. Then, the classified TE sequences were used by REPEATMASKER to identify species-specific TEs in the genome. Ultimately, merging the overlapping coordinates and removing any redundancy was used to produce a non-redundant TE annotation. All TE elements larger than 80 bp in size of the scarlet runner bean and the winged bean, and those larger than 200 bp in size of the sword bean, the smooth rattlebox, and the butterfly pea were soft-masked (uppercase to lowercase) on their genome sequences for gene prediction.

Transcript and homology hints files were used to predict protein-coding gene models by AUGUSTUS version 3.4.0 [52]. The AUGUSTUS parameters of gene prediction were generated from the intermediate outcomes of the BUSCO assessment of genome assembly. Full-length transcripts generated by PacBio Iso-Seq were aligned to the genome using GMAP version 2019-12-01 [53] and using Augustus filter script with parameters "--minId=95 --minCover=95". To obtain homology hints, the ~~proteome~~proteome of 12 representative Papilionoideae species

(*Phaseolus vulgaris* [30], *Vigna angularis* [21], *Lablab purpureus* [22], *Glycine max* [31], *Pueraria montana* [24], *Mucuna pruriens* [23], *Pisum sativum* [6], *Medicago truncatula* [32], *Lotus japonicus* [33], *Aeschynomene evenia* [34], *Arachis hypogaea* [35], *Styphnolobium japonicum* [36]) were aligned to the genome using exonerate version 2.4.0 [54]. The homology and transcript alignment results were transformed into hints files to support gene prediction of AUGUSTUS. To further filter the TE-contaminated genes, the genes whose coordinates overlap more than 99% of the annotated TEs were removed from the gene sets. BUSCO version 5.1.2 [46] was used to evaluate the completeness of the gene sets.

Formatted: Font: Not Italic

Formatted: Font: Not Italic

Formatted: Font: Not Italic

Formatted: Font: Not Italic

For the annotation of gene function, protein sequences were aligned to KEGG and NCBI-NR databases using the DIAMON version 0.8.2 [55] with parameter “E-value 1E−5” to obtain the best hits. The INTERPROSCAN version 5.52-86 [56] with database searching of CDD-3.18, Coils-2.2.1, Gene3D-4.3.0, Hamap-2020_05, MobiDBLite-2.0, PANTHER-15.0, Pfam-33.1, PIRSF-3.10, PIRSR-2021_02, PRINTS-42.0, ProSitePatterns-2021_01, ProSiteProfiles-2021_01, SFLD-4, SMART-7.1, SUPERFAMILY-1.75 and TIGRFAM-15.0, was used to detect the protein domain information and obtain related Gene Ontology (GO) terms. The 8S, 18S and 28S ribosomal RNA (rRNA) were identified utilizing RNAMMER v1.2 [57], and the transfer RNAs (tRNAs) were identified utilizing TRNASCAN-SE v2.0 [58].

Phylogeny and polyploidization Analysis

To construct the orthogroups, we used ORTHOFINDER version 2.5.2 [59] with parameters “-M msa -A mafft -T fasttree -1 -y”, with 12 Papilionoideae species (*Phaseolus vulgaris* [30], *Vigna angularis* [21], *Lablab purpureus* [22], *Glycine max* [31], *Pueraria montana* [24], *Mucuna*

pruriens [23], *Pisum sativum* [6], *Medicago truncatula* [32], *Lotus japonicus* [33], *Aeschynomene evenia* [34], *Arachis hypogaea* [35], *Styphnolobium japonicum* [36]) and one outgroup species *Vitis vinifera* [37].

From the orthogroups of ORTHOFINDER, the OG with *Arachis hypogaea* (recent WGD) [35] having one or two copies and other species having only one copy were selected, then a gene of *Arachis hypogaea* from duplicate genes was randomly thrown. Subsequently, all single-copy genes of all species were used to employ multiple sequence alignment (MSA) utilizing MUSCLE version v3.8.31 [60], and these MSA were combined to create a concatenate multiple sequence alignment (CMSA). Next, the CMSA was used to build a species tree utilizing RAXML version 1.0.3 [61] with parameters "--model GTR+G --tree pars --bs-trees 100 --outgroup Vitis_vinifera". To estimate the divergence time, we used the RelTime branch method in MEGA11 [62] with one calibration time 8.0-19.5 million years ago between *Phaseolus vulgaris* and *Vigna angularis* and the other calibration time 47.7-56 million years ago between *Glycine max* and *Arachis hypogaea*. The two calibration times were obtained from TimeTree (www.timetree.org). Subsequently, the expansion and contraction of gene families was identified using CAFE version 5 [63] with the parameter "-k5".

MCSCANX [64] was used to identify collinear gene blocks with more than five collinear genes. Synteny figures of the whole genome were plotted using the Java programs dual_synteny_plotter and dot_plotter from the MCSCANX package. According to the result of collinear genes, KaKs_CALCULATOR v2.0 [65] with GMYN model was employed to calculate the synonymous substitution rate (*Ks*) value for syntenic gene pairs. Chromosome collinearity

among species was drawn using JCVI (https://github.com/tanghaibao/jcvi) with parameter --cscore=.99”.

Analysis of genes involved in nitrogen-fixing root nodulation

The protein sequences associated with nitrogen-fixing nodulation were obtained from NCBI and Phytozome, and were aligned to the five Papilionoideae species using the ‘blastp’ algorithm in DIAMOND version 0.8.28 [55], with the parameters “--more-sensitive --evalue 0.00001”. The Alignment results were further refined with a 50% identity and 60% coverage threshold. In this way, we identified the potential genes involved in nitrogen-fixing root-nodulation.

To analyze the phylogenetic relationships, the protein sequences within each gene family of *NIN*, *RPG*, *CHS*, and *IFS* were aligned independently using the MUSCLE version 3.8.31 [60]. Subsequently, phylogenetic trees were constructed utilizing the FastTree version 2.1.11 [66]. *V. vinifera* was used as an outgroup. The phylogenetic trees for *NIN* and *RPG* genes were visualized using FigTree version1.4.4 (FigTree (ed.ac.uk)), while those for *CHS* and *IFS* genes were displayed using iTol (https://itol.embl.de/).

Analysis of R genes

The protein sequences of all genes from the five studied plants and *Albizia julibrissin* (NCBI: PRJNA1005079) were searched against the HMM profile of all domains using the hmmsearch program in HMMER version 3.1b2 [67] with the parameters ‘-E 1e-5 --domE 1e-5’. The genes with at least one of the identified domains, including TIR (PF01582), TIR_2 (PF13676), RPW8 (PF05659), NB-ARC (PF00931), LRR_1 (PF00560), LRR_2 (PF07723), LRR_3 (PF07725),

LRR_4 (PF12799), LRR_5 (PF13306), LRR_6 (PF13516), LRR_8 (PF13855), and LRR_9 (PF14580), were chosen as the primary potential R genes. In addition, Coiled-coil (CC) domains were furthter annotated using the Coils database from INTERPROSCAN version 5.52-86 [56]. The genes with TIR-NB-LRR, CC-NB-LRR and RPW8-NB-LRR domain structures were classified as TNL, CNL and RNL R genes, respectively. The alignment of protein sequences for CNL and TNL R genes was performed using MUSCLE version 3.8.31 [60]. Subsequently, the phylogenetic tree was constructed by FastTree version 2.1.11 [66], which was displayed using iTOL (https://itol.embl.de/).

Acknowledgements

We also thank Prof. Shifeng Cheng for giving helpful suggestions.

Data availability

The genomic and transcriptomic sequencing reads generated in this study have been deposited in SRA of NCBI under the accession PRJNA1001638, PRJNA1002813, PRJNA1003673, PRJNA1014360, PRJNA1016062 for *Canavalia gladiata*, *Phaseolus coccineus*, *Psophocarpus tetragonolobus*, *Crotalaria pallida*, and *Clitoria ternatea*, respectively. The genome assemblies and gene annotations have been deposited at GenBank of NCBI under the accession JAYMYQ000000000, JAYMYR000000000, JAYMYS000000000, JAYWIO000000000, JAYKXN000000000 and also have been deposited at Figshare [10.6084/m9.figshare.24994343, 10.6084/m9.figshare.24995750, 10.6084/m9.figshare.24995753, 10.6084/m9.figshare.24995756, 10.6084/m9.figshare.24995759] for *Canavalia gladiata*, *Phaseolus coccineus*, *Psophocarpus*

tetragonolobus, *Crotalaria pallida*, and *Clitoria ternatea*, respectively.

Funding

This work was supported by the Shenzhen Science and Technology Program (JCYJ20190814163805604, KQTD20180411143628272); Fund of Key Laboratory of Shenzhen (ZDSYS20141118170111640) and The Agricultural Science and Technology Innovation Program.

Author contributions

L.H.Y. and F.J. prepared the genomic and transcriptomic sequencing samples. L.H.Y., L.H.L., F.J., A.Q.W., R.C., H.C.W. and S.H.M. completed the bioinformatic analyses. L.H.Y. and L.H.L. made the tables, figures, and Supplemental information. W.F. supervised the project, and W.F., L.H.Y and L.H.L wrote the manuscript, and all authors revised and approved the final version of this manuscript.

Competing Interests

The authors declare no competing interest.

References

1. Zhao Y, Zhang R, Jiang KW, Qi J, Hu Y, Guo J, et al. Nuclear phylotranscriptomics and phylogenomics support numerous polyploidization events and hypotheses for the evolution of rhizobial nitrogen-fixing symbiosis in Fabaceae. Mol Plant. 2021; 14(5): 748-73. doi:10.1016/j.molp.2021.02.006.
2. Schmutz J, Cannon SB, Schlueter J, Ma J, Mitros T, Nelson W, et al. Genome sequence of the palaeopolyploid soybean. Nature. 2010; 463(7278): 178-83. doi:10.1038/nature08670.
3. Zhuang W, Chen H, Yang M, Wang J, Pandey MK, Zhang C, et al. The genome of cultivated peanut provides insight into legume karyotypes, polyploid evolution and crop domestication. Nat Genet. 2019; 51(5): 865-76. doi:10.1038/s41588-019-0402-2.
4. Jayakodi M, Golicz AA, Kreplak J, Fechete LI, Angra D, Bednar P, et al. The giant diploid faba genome unlocks variation in a global protein crop. Nature. 2023; 615(7953): 652-9. doi:10.1038/s41586-023-05791-5.
5. Kang YJ, Kim SK, Kim MY, Lestari P, Kim KH, Ha BK, et al. Genome sequence of mungbean and insights into evolution within Vigna species. Nat Commun. 2014; 5: 5443. doi:10.1038/ncomms6443.
6. Yang T, Liu R, Luo Y, Hu S, Wang D, Wang C, et al. Improved pea reference genome and pan-genome highlight genomic features and evolutionary characteristics. Nat Genet. 2022; 54(10): 1553-63. doi:10.1038/s41588-022-01172-2.
7. Carrère S, Mayjonade B, Lalanne D, Gaillard S, Verdier J and Chen NWG. First whole genome assembly and annotation of a European common bean cultivar using PacBio HiFi and Iso-Seq data. Data Brief. 2023; 48(109182). doi:10.1016/j.dib.2023.109182.
8. Shen C, Du HL, Chen Z, Lu HW, Zhu FG, Chen H, et al. The Chromosome-Level Genome Sequence of the Autotetraploid Alfalfa and Resequencing of Core Germplasms Provide Genomic Resources for Alfalfa Research. Molecular Plant. 2020; 13(9): 1250-61. doi:10.1016/j.molp.2020.07.003.
9. Ayilara MS, Abberton M, Oyatomi OA, Odeyemi O and Babalola OO. Potentials of underutilized legumes in food security. Front Soil Sci. 2022; 2. doi:10.3389/fsoil.2022.1020193.
10. Maneechot O, Hahor W, Thongprajukaew K, Nuntapong N and Bubaka S. A natural blue colorant from butterfly pea (*Clitoria ternatea*) petals for traditional rice cooking. J Food Sci Tech Mys. 2023; 60(8): 2255-64. doi:10.1007/s13197-023-05752-w.
11. Hu Y, Chen XJ, Hu M, Zhang DW, Yuan S, Li P, et al. Medicinal and edible plants in the treatment of dyslipidemia: advances and prospects. Chin Med-Uk. 2022; 17(1). doi:10.1186/s13020-022-00666-9.
12. Gautam AK, Sharma D, Sharma J and Saini KC. Legume lectins: Potential use as a diagnostics and therapeutics against the cancer. Int J Biol Macromol. 2020; 142: 474-83. doi:10.1016/j.ijbiomac.2019.09.119.
13. Desbrosses GJ and Stougaard J. Root Nodulation: A Paradigm for How Plant-Microbe Symbiosis Influences Host Developmental Pathways. Cell Host Microbe. 2011; 10(4): 348-58. doi:10.1016/j.chom.2011.09.005.
14. Huisman R and Geurts R. A Roadmap toward Engineered Nitrogen-Fixing Nodule Symbiosis. Plant Commun. 2020; 1(1): 100019. doi:10.1016/j.xplc.2019.100019.
15. Young ND, Debelle F, Oldroyd GED, Geurts R, Cannon SB, Udvardi MK, et al. The

Medicago genome provides insight into the evolution of rhizobial symbioses. Nature. 2011; 480(7378): 520-4. doi:10.1038/nature10625.

16. Sato S, Nakamura Y, Kaneko T, Asamizu E, Kato T, Nakao M, et al. Genome structure of the legume, Lotus japonicus. DNA Res. 2008; 15(4): 227-39. doi:10.1093/dnares/dsn008.
17. Yang J, Lan LY, Jin Y, Yu N, Wang D and Wang E. Mechanisms underlying legume-rhizobium symbioses. J Integr Plant Biol. 2022; 64(2): 244-67. doi:10.1111/jipb.13207.
18. Kundu S and Hargrove MS. Distal heme pocket regulation of ligand binding and stability in soybean leghemoglobin. Proteins. 2003; 50(2): 239-48. doi:10.1002/prot.10277.
19. Griesmann M, Chang Y, Liu X, Song Y, Haberer G, Crook MB, et al. Phylogenomics reveals multiple losses of nitrogen-fixing root nodule symbiosis. Science. 2018; 361(6398). doi:10.1126/science.aat1743.
20. Libourel C, Keller J, Brichet L, Cazale AC, Carrere S, Vernie T, et al. Comparative phylotranscriptomics reveals ancestral and derived root nodule symbiosis programmes. Nat Plants. 2023; 9(7): 1067-80. doi:10.1038/s41477-023-01441-w.
21. Yang K, Tian ZX, Chen CH, Luo LH, Zhao B, Wang Z, et al. Genome sequencing of adzuki bean (Vigna angularis) provides insight into high starch and low fat accumulation and domestication. P Natl Acad Sci USA. 2015; 112(43): 13213-8. doi:10.1073/pnas.1420949112.
22. Njaci I, Waweru B, Kamal N, Muktar MS, Fisher D, Gundlach H, et al. Chromosome-level genome assembly and population genomic resource to accelerate orphan crop lablab breeding. Nat Commun. 2023; 14(1): 1915. doi:10.1038/s41467-023-37489-7.
23. Hao S, Ge Q, Shao Y, Tang B, Fan G, Qiu C, et al. Chromosomal-level genome of velvet bean (Mucuna pruriens) provides resources for L-DOPA synthetic research and development. DNA Res. 2022; 29(5). doi:10.1093/dnares/dsac031.
24. Mo CJ, Wu ZD, Shang XH, Shi PL, Wei MH, Wang HY, et al. Chromosome-level and graphic genomes provide insights into metabolism of bioactive metabolites and cold-adaption of Pueraria lobata var. montana. DNA Research. 2022; 29(5). doi:10.1093/dnares/dsac030.
25. Chen HF, Yao XZ, Cao BH, Zhang BH, Lu LT and Mao PL. A chromosome-level genome assembly of Styphnolobium japonicum combined with comparative genomic analyses offers insights on the evolution of flavonoid and lignin biosynthesis. Ind Crop Prod. 2022; 187. doi:10.1016/j.indcrop.2022.115336.
26. Ho WK, Tanzi AS, Sang F, Tsoutsoura N, Shah N, Moore C, et al. A genomic toolkit for winged bean Psophocarpus tetragonolobus. Nat Commun. 2024; 15(1): 1901. doi:10.1038/s41467-024-45048-x.
27. Liu B, Shi Y, Yuan J, Hu X, Zhang H, Li N, et al. Estimation of genomic characteristics by analyzing k-mer frequency in de novo genome projects. 2013.
28. Rhie A, Walenz BP, Koren S and Phillippy AM. Merqury: reference-free quality, completeness, and phasing assessment for genome assemblies. Genome Biol. 2020; 21(1): 245. doi:10.1186/s13059-020-02134-9.
29. Galindo-González L, Mhiri C, Deyholos MK and Grandbastien MA. LTR-retrotransposons in plants: Engines of evolution. Gene. 2017; 626: 14-25. doi:10.1016/j.gene.2017.04.051.
30. Schmutz J, McClean PE, Mamidi S, Wu GA, Cannon SB, Grimwood J, et al. A reference genome for common bean and genome-wide analysis of dual domestications. Nat Genet. 2014; 46(7): 707-13. doi:10.1038/ng.3008.
31. Shen YT, Du HL, Liu YC, Ni LB, Wang Z, Liang CZ, et al. Update soybean Zhonghuang 13

genome to a golden reference. Sci China Life Sci. 2019; 62(9): 1257-60. doi:10.1007/s11427-019-9822-2.
32. Pecrix Y, Staton SE, Sallet E, Lelandais-Brère C, Moreau S, Carrère S, et al. Whole-genome landscape of symbiotic genes. Nature Plants. 2018; 4(12): 1017-25. doi:10.1038/s41477-018-0286-7.
33. Kamal N, Mun T, Reid D, Lin JS, Akyol TY, Sandal N, et al. Insights into the evolution of symbiosis gene copy number and distribution from a chromosome-scale Gifu genome sequence. DNA Research. 2020; 27(3). doi:10.1093/dnares/dsaa015.
34. Quilbe J, Lamy L, Brottier L, Leleux P, Fardoux J, Rivallan R, et al. Genetics of nodulation in Aeschynomene evenia uncovers mechanisms of the rhizobium-legume symbiosis. Nat Commun. 2021; 12(1): 829. doi:10.1038/s41467-021-21094-7.
35. Chen XP, Lu Q, Liu H, Zhang JA, Hong YB, Lan HF, et al. Sequencing of Cultivated Peanut，Yields Insights into Genome Evolution and Oil Improvement. Molecular Plant. 2019; 12(7): 920-34. doi:10.1016/j.molp.2019.03.005.
36. Lei W, Wang Z, Cao M, Zhu H, Wang M, Zou Y, et al. Chromosome-level genome assembly and characterization of Sophora Japonica. DNA Res. 2022; 29(3). doi:10.1093/dnares/dsac009.
37. Jaillon O, Aury JM, Noel B, Policriti A, Clepet C, Casagrande A, et al. The grapevine genome sequence suggests ancestral hexaploidization in major angiosperm phyla. Nature. 2007; 449(7161): 463-7. doi:10.1038/nature06148.
38. Cannon SB, McKain MR, Harkess A, Nelson MN, Dash S, Deyholos MK, et al. Multiple Polyploidy Events in the Early Radiation of Nodulating and Nonnodulating Legumes. Mol Biol Evol. 2015; 32(1): 193-210. doi:10.1093/molbev/msu296.
39. Wang LL, Rubio MC, Xin X, Zhang BL, Fan QL, Wang Q, et al. CRISPR/Cas9 knockout of leghemoglobin genes in uncovers their synergistic roles in symbiotic nitrogen fixation. New Phytol. 2019; 224(2): 818-32. doi:10.1111/nph.16077.
40. Subramanian S, Stacey G and Yu O. Endogenous isoflavones are essential for the establishment of symbiosis between soybean and. Plant J. 2006; 48(2): 261-73. doi:10.1111/j.1365-313X.2006.02874.x.
41. Al-Maharik N. Isolation of naturally occurring novel isoflavonoids: an update. Nat Prod Rep. 2019; 36(8): 1156-95. doi:10.1039/c8np00069g.
42. Gururani MA, Venkatesh J, Upadhyaya CP, Nookaraju A, Pandey SK and Park SW. Plant disease resistance genes: Current status and future directions. Physiol Mol Plant P. 2012; 78: 51-65. doi:10.1016/j.pmpp.2012.01.002.
43. Shao ZQ, Xue JY, Wu P, Zhang YM, Wu Y, Hang YY, et al. Large-Scale Analyses of Angiosperm Nucleotide-Binding Site-Leucine-Rich Repeat Genes Reveal Three Anciently Diverged Classes with Distinct Evolutionary Patterns. Plant Physiol. 2016; 170(4): 2095-109. doi:10.1104/pp.15.01487.
44. Cheng H, Concepcion GT, Feng X, Zhang H and Li H. Haplotype-resolved de novo assembly using phased assembly graphs with hifiasm. Nat Methods. 2021; 18(2): 170-5. doi:10.1038/s41592-020-01056-5.
45. Li H. Minimap2: pairwise alignment for nucleotide sequences. Bioinformatics. 2018; 34(18): 3094-100. doi:10.1093/bioinformatics/bty191.
46. Simao FA, Waterhouse RM, Ioannidis P, Kriventseva EV and Zdobnov EM. BUSCO: assessing genome assembly and annotation completeness with single-copy orthologs.

Bioinformatics. 2015; 31(19): 3210-2. doi:10.1093/bioinformatics/btv351.

47. Servant N, Varoquaux N, Lajoie BR, Viara E, Chen CJ, Vert JP, et al. HiC-Pro: an optimized and flexible pipeline for Hi-C data processing. Genome Biol. 2015; 16: 259. doi:10.1186/s13059-015-0831-x.

48. Wang S, Wang H, Jiang F, Wang A, Liu H, Zhao H, et al. EndHiC: assemble large contigs into chromosome-level scaffolds using the Hi-C links from contig ends. BMC Bioinformatics. 2022; 23(1): 528. doi:10.1186/s12859-022-05087-x.

49. Benson G. Tandem repeats finder: a program to analyze DNA sequences. Nucleic Acids Res. 1999; 27(2): 573-80. doi:10.1093/nar/27.2.573.

50. Ou S, Su W, Liao Y, Chougule K, Agda JRA, Hellinga AJ, et al. Author Correction: Benchmarking transposable element annotation methods for creation of a streamlined, comprehensive pipeline. Genome Biol. 2022; 23(1): 76. doi:10.1186/s13059-022-02645-7.

51. da Cruz MHP, Domingues DS, Saito PTM, Paschoal AR and Bugatti PH. TERL: classification of transposable elements by convolutional neural networks. Brief Bioinform. 2021; 22(3). doi:10.1093/bib/bbaa185.

52. Stanke M, Diekhans M, Baertsch R and Haussler D. Using native and syntenically mapped cDNA alignments to improve de novo gene finding. Bioinformatics. 2008; 24(5): 637-44. doi:10.1093/bioinformatics/btn013.

53. Wu TD and Watanabe CK. GMAP: a genomic mapping and alignment program for mRNA and EST sequences. Bioinformatics. 2005; 21(9): 1859-75. doi:10.1093/bioinformatics/bti310.

54. Slater GS and Birney E. Automated generation of heuristics for biological sequence comparison. BMC Bioinformatics. 2005; 6: 31. doi:10.1186/1471-2105-6-31.

55. Buchfink B, Reuter K and Drost HG. Sensitive protein alignments at tree-of-life scale using DIAMOND. Nature Methods. 2021; 18(4): 366-8. doi:10.1038/s41592-021-01101-x.

56. Blum M, Chang HY, Chuguransky S, Grego T, Kandasaamy S, Mitchell A, et al. The InterPro protein families and domains database: 20 years on. Nucleic Acids Research. 2021; 49(D1): D344-D54. doi:10.1093/nar/gkaa977.

57. Lagesen K, Hallin P, Rodland EA, Stærfeldt HH, Rognes T and Ussery DW. RNAmmer:: consistent and rapid annotation of ribosomal RNA genes. Nucleic Acids Research. 2007; 35(9): 3100-8. doi:10.1093/nar/gkm160.

58. Chan PP, Lin BY, Mak AJ and Lowe TM. tRNAscan-SE 2.0: improved detection and functional classification of transfer RNA genes. Nucleic Acids Res. 2021; 49(16): 9077-96. doi:10.1093/nar/gkab688.

59. Emms DM and Kelly S. OrthoFinder: phylogenetic orthology inference for comparative genomics. Genome Biol. 2019; 20(1): 238. doi:10.1186/s13059-019-1832-y.

60. Edgar RC. MUSCLE: multiple sequence alignment with high accuracy and high throughput. Nucleic Acids Research. 2004; 32(5): 1792-7. doi:10.1093/nar/gkh340.

61. Kozlov AM, Darriba D, Flouri T, Morel B and Stamatakis A. RAxML-NG: a fast, scalable and user-friendly tool for maximum likelihood phylogenetic inference. Bioinformatics. 2019; 35(21): 4453-5. doi:10.1093/bioinformatics/btz305.

62. Tamura K, Stecher G and Kumar S. MEGA11 Molecular Evolutionary Genetics Analysis Version 11. Mol Biol Evol. 2021; 38(7): 3022-7. doi:10.1093/molbev/msab120.

63. Mendes FK, Vanderpool D, Fulton B and Hahn MW. CAFE 5 models variation in evolutionary rates among gene families. Bioinformatics. 2020; 36(22-23): 5516-8.

doi:10.1093/bioinformatics/btaa1022.

64. Wang YP, Tang HB, DeBarry JD, Tan X, Li JP, Wang XY, et al.: a toolkit for detection and evolutionary analysis of gene synteny and collinearity. Nucleic Acids Research. 2012; 40(7). doi:10.1093/nar/gkr1293.

65. Wang D, Zhang Y, Zhang Z, Zhu J and Yu J. KaKs_Calculator 2.0: a toolkit incorporating gamma-series methods and sliding window strategies. Genomics Proteomics Bioinformatics. 2010; 8(1): 77-80. doi:10.1016/S1672-0229(10)60008-3.

66. Price MN, Dehal PS and Arkin AP. FastTree: Computing Large Minimum Evolution Trees with Profiles instead of a Distance Matrix. Mol Biol Evol. 2009; 26(7): 1641-50. doi:10.1093/molbev/msp077.

67. Mistry J, Finn RD, Eddy SR, Bateman A and Punta M. Challenges in homology search: HMMER3 and convergent evolution of coiled-coil regions. Nucleic Acids Research. 2013; 41(12). doi:10.1093/nar/gkt263.

Table

Table 1. Statistics of genome assembly and annotation.

Genomic features	*Canavalia gladiata*	*Phaseolus coccineus*	*Psophocarpus tetragonolobus*	*Crotalaria pallida*	*Clitoria ternatea*
**Genome assembly**					
**Estimated genome size by K-mer (Mb)**	650	593	689	1,331	1,761
**Total assembly size (bp)**	619,186,046	592,734,161	712,813,888	1,217,645,575	1,724,627,994
**Contig N50 size (bp)**	39,462,069	39,559,522	13,237,817	100,840,643	126,428,166
**Scaffold N50 size (bp)**	55,284,388	52,871,251	79,694,302	142,152,887	168,933,288
**% of sequences anchored to chromosomes**	97.5%	95.7%	93.7%	98.2%	97.5%
**% of telomeres assembled**	72.7%	77.3%	55.6%	93.8%	75.0%
**busco complete rate of the genome**	99.4%	99.3%	99.2%	99.0%	99.0%
**QV**	70.0	74.3	69.5	69.6	72.2
**Genome annotation**					
**Length and % of tandem repeats (bp)**	105,656,450 (17.1%)	54,428,626 (9.2%)	134,417,242 (18.9%)	119,729,388 (9.8%)	130,389,725 (7.6%)
**Length and % of TE sequences (bp)**	341,308,218 (55%)	376,131,126 (63%)	456,822,987 (64%)	994,314,842 (82%)	148,366,6381 (86%)
**Number of tRNA genes**	970	1,141	1,283	1,382	2,307
**Number of rRNA (5S + 18S + 28S) genes**	1,535	5,030	3,020	6,268	3,158
**Number of protein-coding gene models**	51,158	35,523	40,081	48,759	40,267
**Total CDS size and % in genome (bp)**	50,888,808 (8.2%)	42,292,638 (7.1%)	43,752,003 (7.4%)	51,214,428 (4.2%)	41,669,007 (2.4%)
**BUSCO complete rate of the genes**	99.4%	99.6%	99.2%	98.1%	98.9%

Figure Legends

Figure 1. Circos plot of genomic annotations. **(A)** *Canavalia gladiata*, **(B)** P*haseolus coccineus*, **(C)** *Psophocarpus tetragonolobus*, **(D)** *Crotalaria pallida* and **(E)** *Clitoria ternatea*. The 5 circular tracks from inner to outer refer to (a) GC percentage, (b) transposable element (TE) density, (c) tandem repeat (TR) density, and (d) gene density. These features were calculated by sliding 1-Mb windows. Pictures of species are placed inside the center of the circos plot.

Figure 2. TEs in the 5 sequenced species in this study. (A) Distribution of various types of transposable elements (TEs) in each species. **(B)** a scatter plot illustrating the correlation between the length of ~~long terminal repeats (LTRs)~~LTR-RTs and the genome size. **(C)** Distribution of

various types of ~~long terminal repeats (LTRs)~~LTR-RTs in each species. **(D)** the insert time distribution of intact long terminal repeats (LTRs) for each species. The sequence of LTRs from intact LTR-RTs identified by EDTA version 1.9.9 were obtained, and the long terminal repeats of each LTR-RTs were aligned using MUSCLE version v3.8.31.The APE package in R and the K80 model (https://github.com/wangziwei08/LTR-insertion-time-estimation)was used to estimate the Pairwise distances from the LTR.

Figure 3. Evolution of Papilionoideae. **(A)** Phylogentic tree with divergence time estimated by the RelTime branch method in mega. Two calibration constraints were used: one was 8.0-19.5 million years ago between *Phaseolus vulgaris* and *Vigna angularis*, and the other was 47.7-56 million years ago between *Glycine max* and *Arachis hypogaea*. The five sequenced species in this study are marked with blue stars. The numbers on the side of the nodes represent divergence time values, and the whole-genome polyploidization events are indicated on the branches in red. **(B)** Homologous Ks distribution within species, paralogous gene pairs situated on collinear fragments containing over five syntenic gene pairs are employed for Ks calculation using the GMYN model in the KaKS_CALCULATOR. **(C)** Macro-synteny plots among the 5 studied species.

Figure 4. Genomic comparison between *Phaseolus coccineus* (scarlet runner bean) **and *Phaseolus vulgaris* (**common bean). **(A)** Macro-synteny blocks between *P. coccineus* and *P. vulgaris*. Collinear fragments containing over 20 syntenic gene pairs are utilized. Pc and Pv represent *P. coccineus* and *P. vulgaris* respectively. **(B)** Distribution of LTR TEs, DNA TEs, other TEs, tandem repeats (TR), and Non-repeat regions in *P. coccineus* and *P. vulgaris*. **(C)** Overlap of the reference gene sets between *P. coccineus* and *P. vulgaris*. The protein sequences are aligned using Diamond with the parameters "--sensitive --evalue 1e-5". Genes that remain unaligned are considered as species-specific genes.

Figure 5. Root nodulation symbiosis. **(A)** Pathway of crucial genes involved in symbiotic nodulation and nitrogen fixation for Papilionoideae. The model figure is drawn using Figdraw. **(B)** The gene number of symbiotic pathway identified in *C.gladiata*, *P. coccineus*, *P.tetragonolobus*, *C.pallida*, and *C.ternatea*. The data source is from previous studies. **(C)** Gene tree for *NIN*. Members of the gene family were obtained from orthoFinder orthogroups, and the gene tree was constructed by FastTree. Bootstrap values are shown on each branch, and the two duplicated branches were highlighted. *V. vinifera* is utilized as the outgroup. **(D)** Gene tree for *CHS*, with similar style to *NIN*. The genes from the five sequenced species were shown in five different colors, and the tandem replicated genes of different species have been highlighted using distinct background colors.

Figure 6. Resistance (R) genes. (A) Model figure for three types of R genes TNL, CNL and RNL. TNL consists of TIR, NB-ARC, and LRR domains, CNL consists of CC, NB-ARC, and LRR domains, while RNL consists of RPW8, NB-ARC, and LRR domains from N-terminal to C-

terminal. **(B)** The number of CNL-type and TNL-type R genes in *C.gladiata*, *P. coccineus*, *P.tetragonolobus*, *C.pallida*, *C.ternatea* and *A.julibrissin* species, respectively. **(C)** The phylogenetic tree of all identified R genes in the 5 studied species and one outgroup (*A.julibrissin*). The two major branches TNL and CNL are labeled with different background colors, and a solid circle on the clade represents an OG, and the outgroup is marked with a gray graph. The R genes from various species are differentiated by various symbols.

Figure1

Click here to access/download;Figure;figure1.pdf

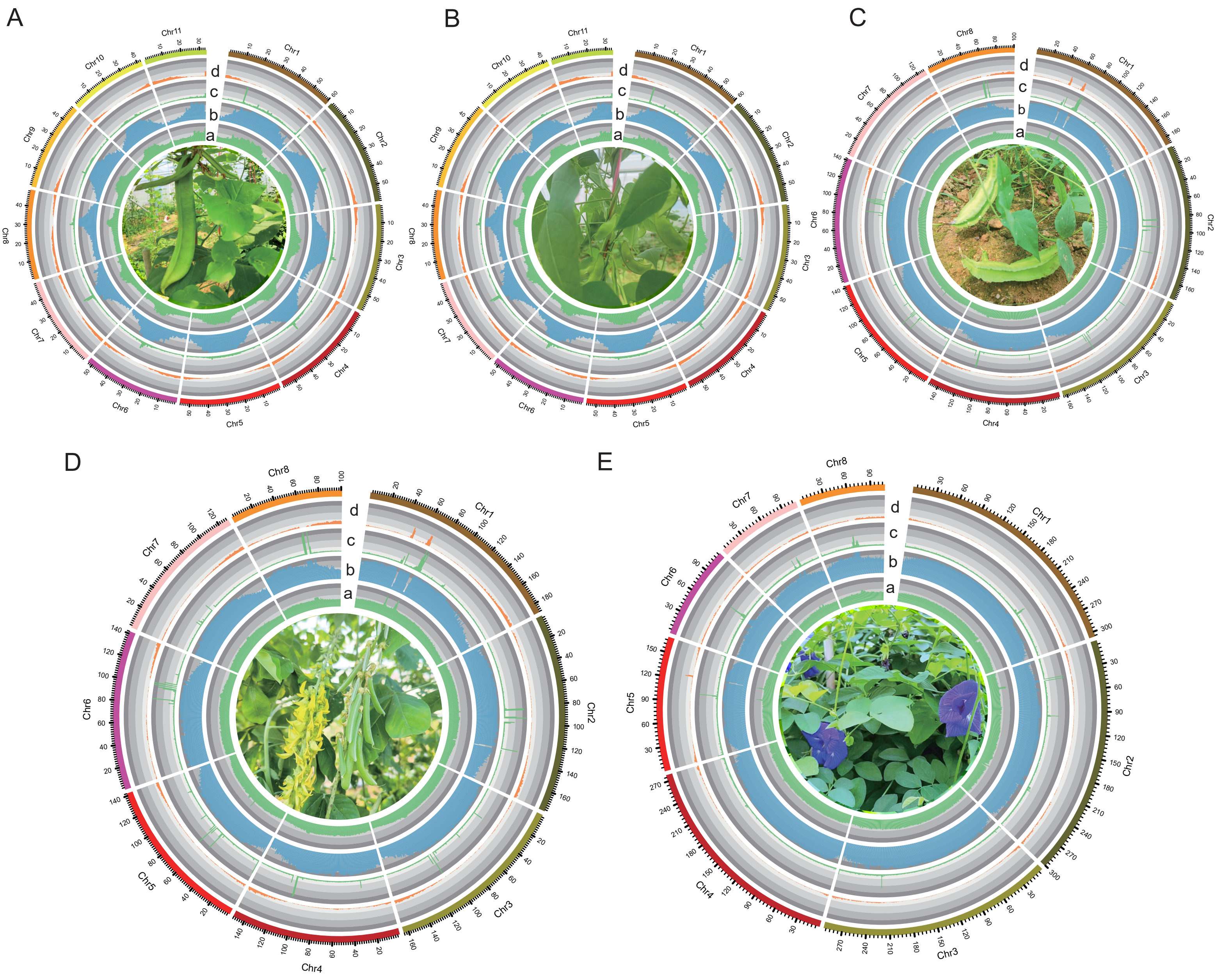

Figure 2

Click here to access/download;Figure;figure2.pdf

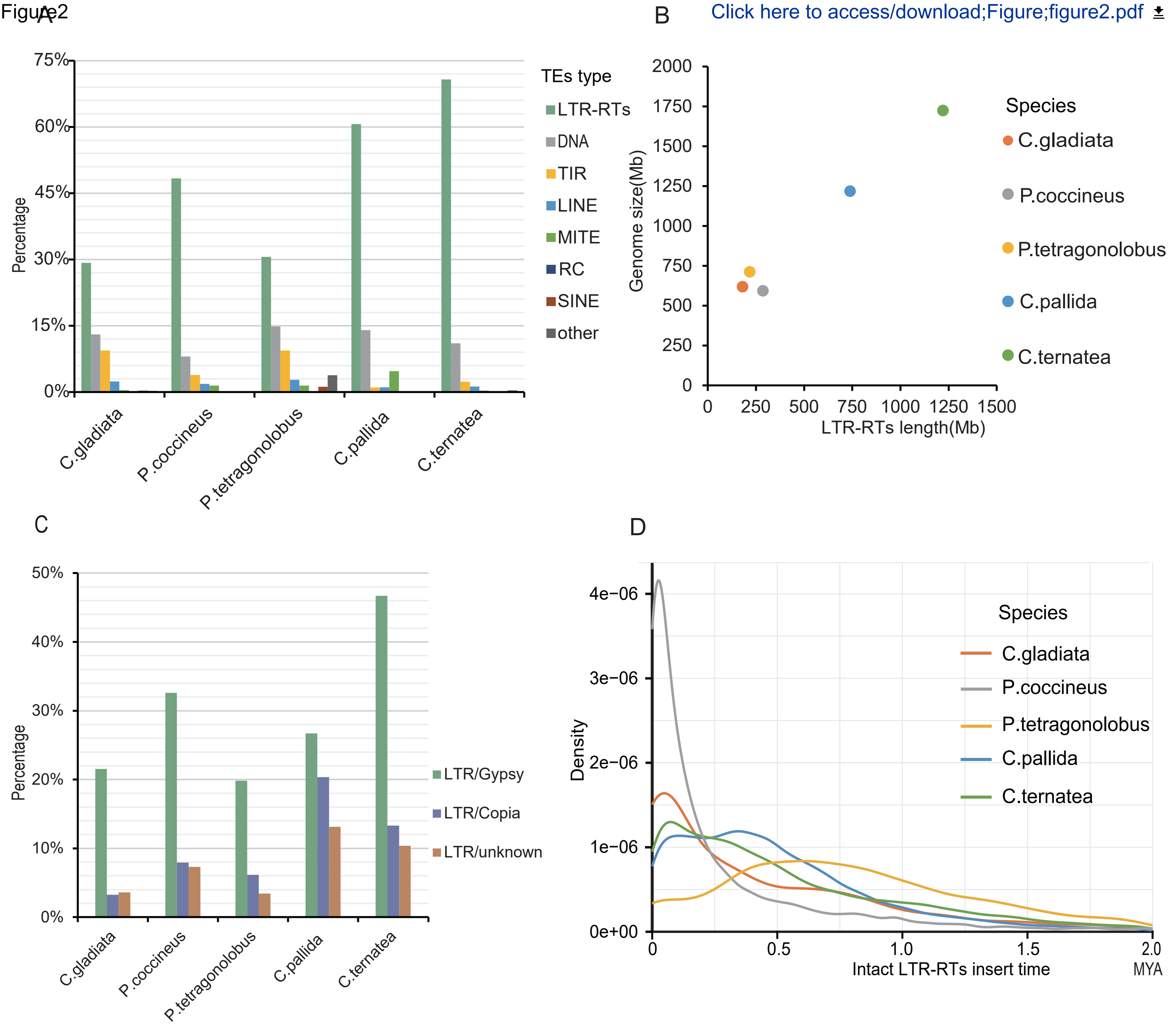

Figure3

Click here to access/download;Figure;figure3.pdf

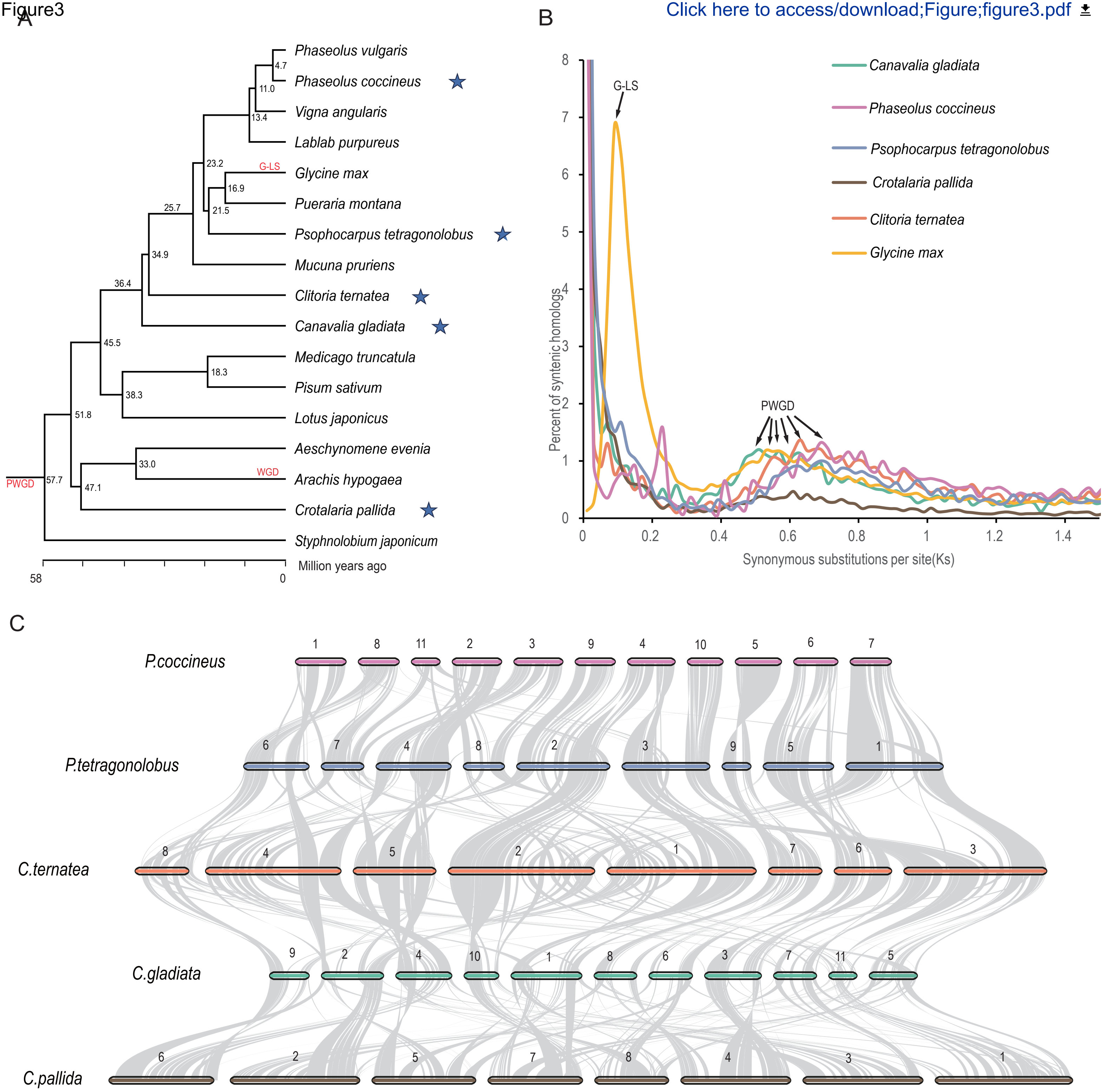

Figure4

Click here to access/download;Figure;figure4.pdf

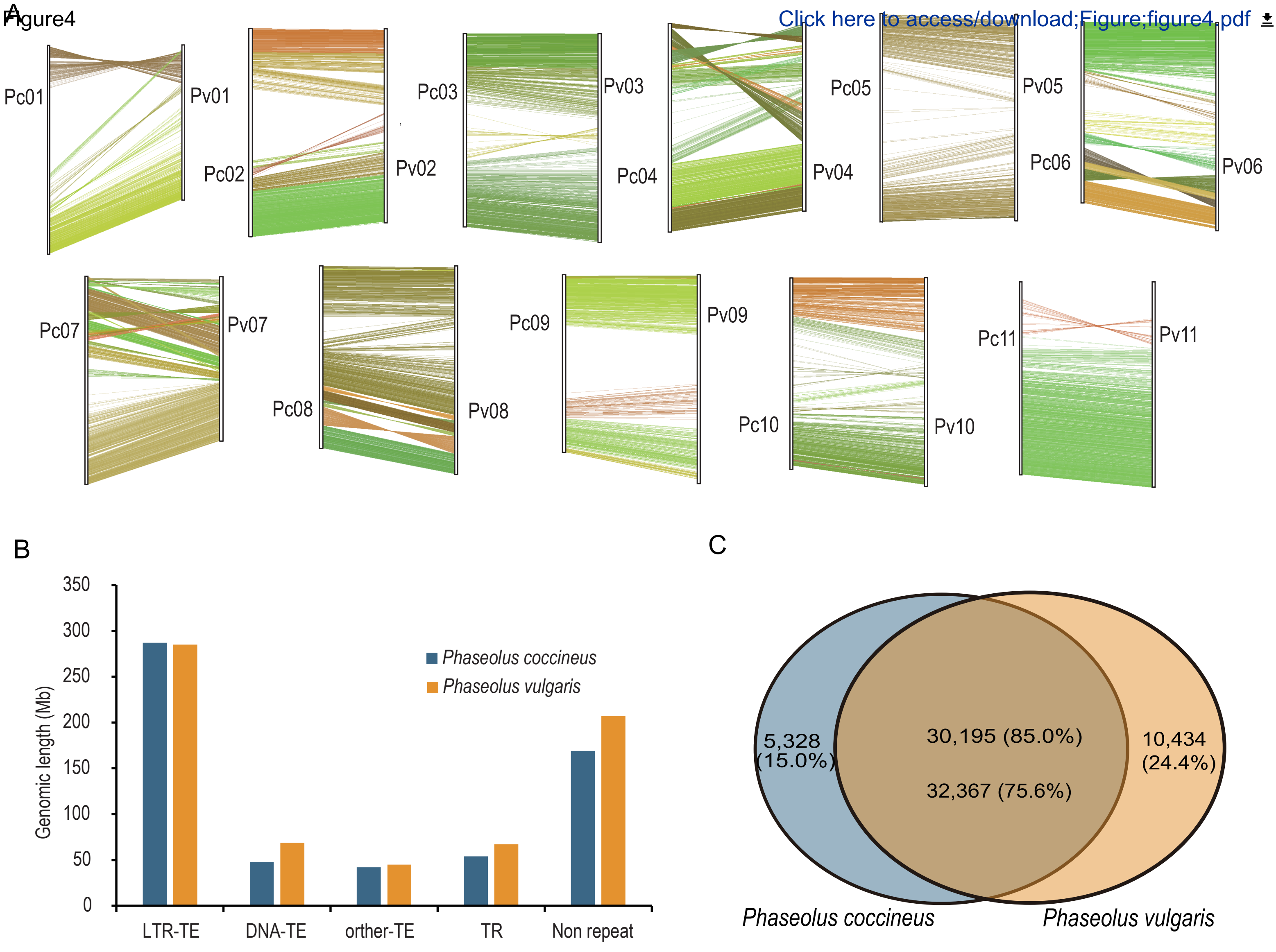

Figure5

Click here to access/download;Figure;figure5.pdf

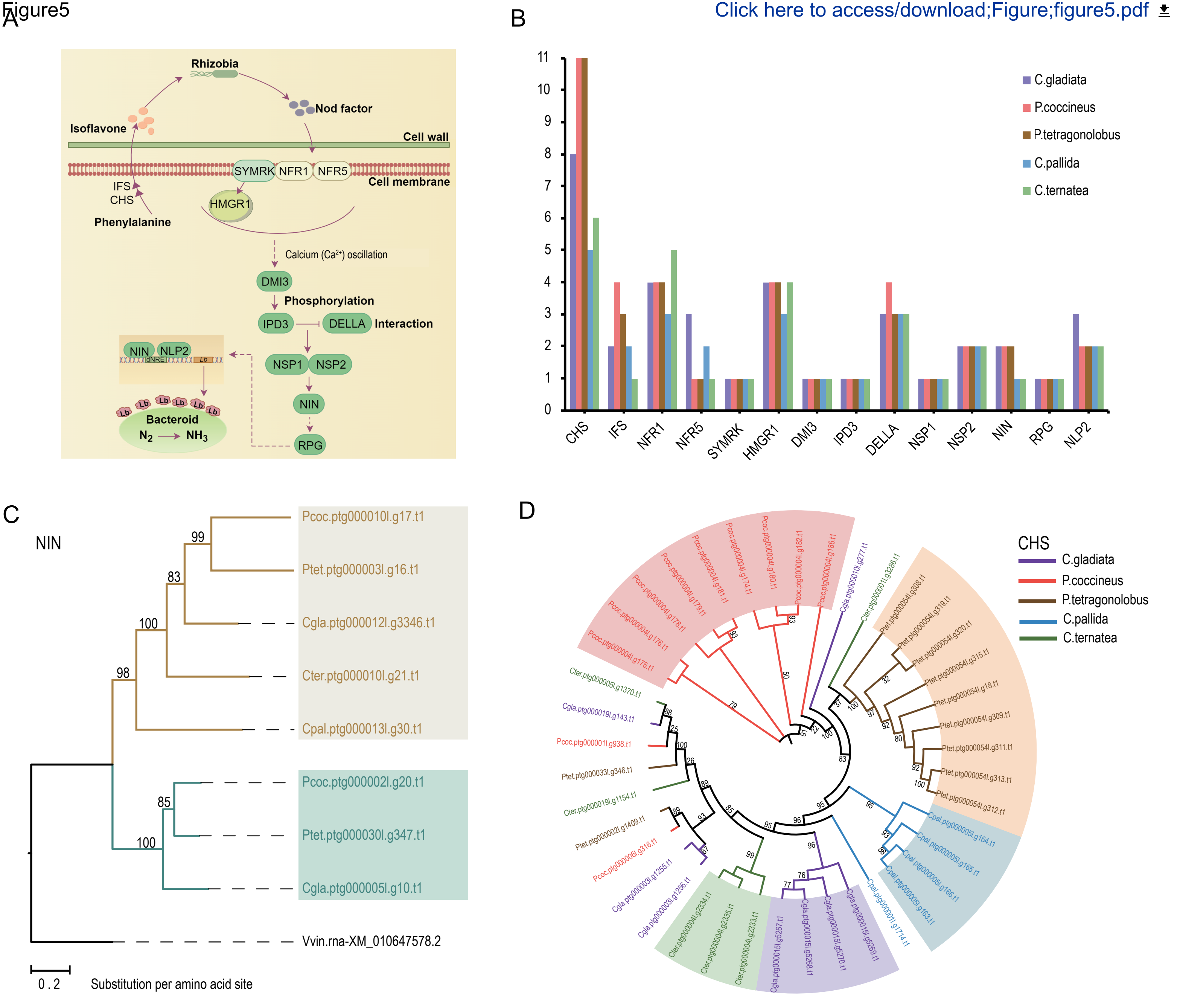

Figure6

Click here to access/download;Figure;figure6.pdf

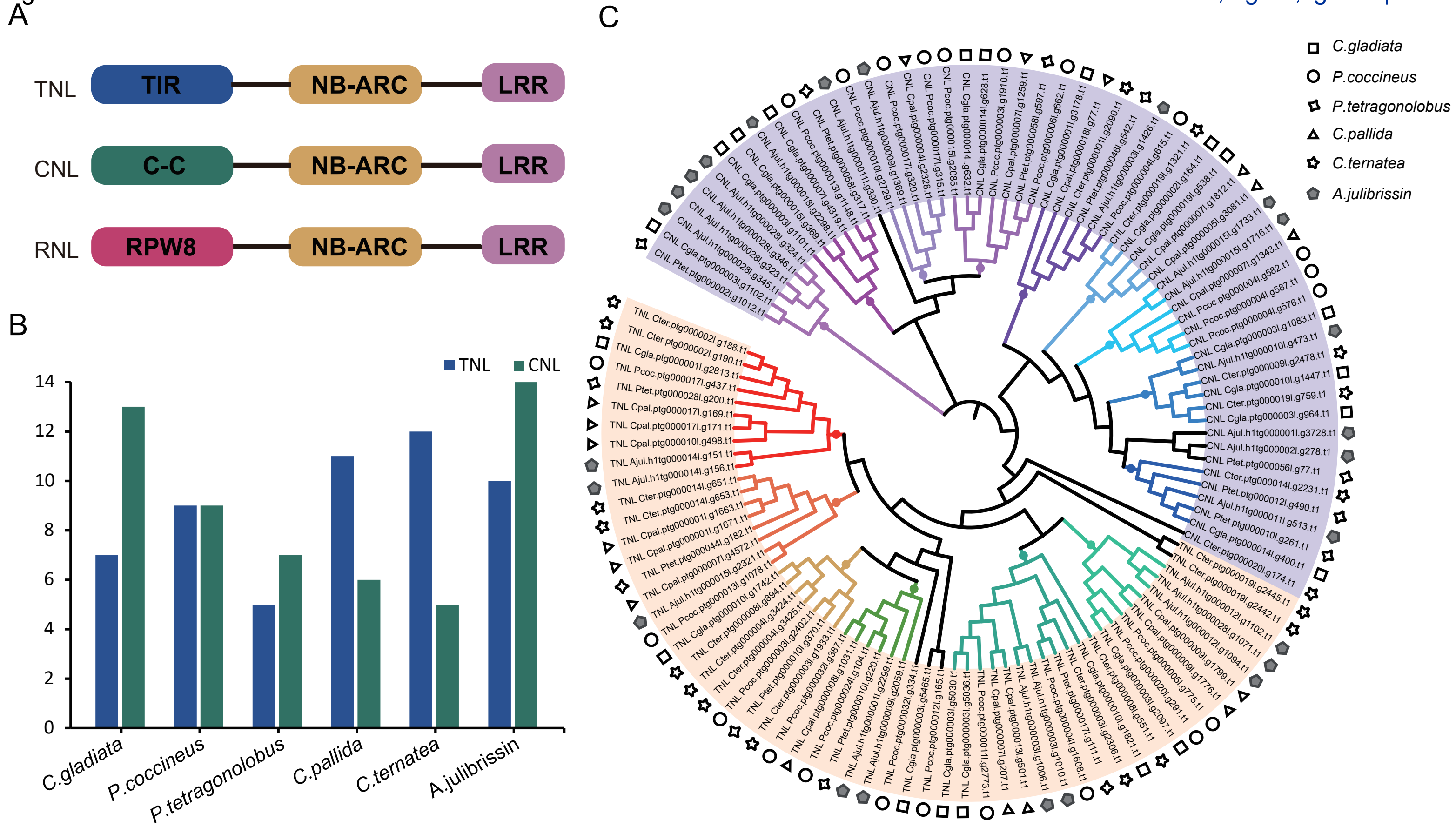

Click here to access/download
Supplementary Material
Supplementary_materials.docx
