# Supplementary material for: The genomes of 5 underutilized Papilionoideae crops provide insights into root nodulation and disease resistance
Source: Gigascience. 2024 Aug 27;13:giae063. doi: 10.1093/gigascience/giae063 (PMC11348429; doi:10.1093/gigascience/giae063)
Supplement: giae063_Supplemental_File [file giae063_supplemental_file.docx]

# Supplementary Information for

# The genomes of five underutilized Papilionoideae crops provide insights into root nodulation and disease resistance

Lihua Yuan^1,2,3,4^, Lihong Lei^1,2,3,4^, Fan Jiang^1,4^, Anqi Wang^1^, Rong Chen^1^, Hengchao Wang^1^, Sihan Meng^1^, Wei Fan^1,^*

^1^Guangdong Laboratory for Lingnan Modern Agriculture (Shenzhen Branch), Genome Analysis Laboratory of the Ministry of Agriculture and Rural Affairs, Agricultural Genomics Institute at Shenzhen, Chinese Academy of Agricultural Sciences, Shenzhen, Guangdong, 518120, China

^2^State Key Laboratory of Crop Stress Adaptation and Improvement, School of Life Sciences, Henan University, Kaifeng 475004, China

^3^Shenzhen Research Institute of Henan University, Shenzhen 518000, China

^4^These authors contributed equally to this article.

*Correspondence author: Wei Fan ([fanwei@caas.cn](mailto:fanwei@caas.cn))

**Supplementary Figures**


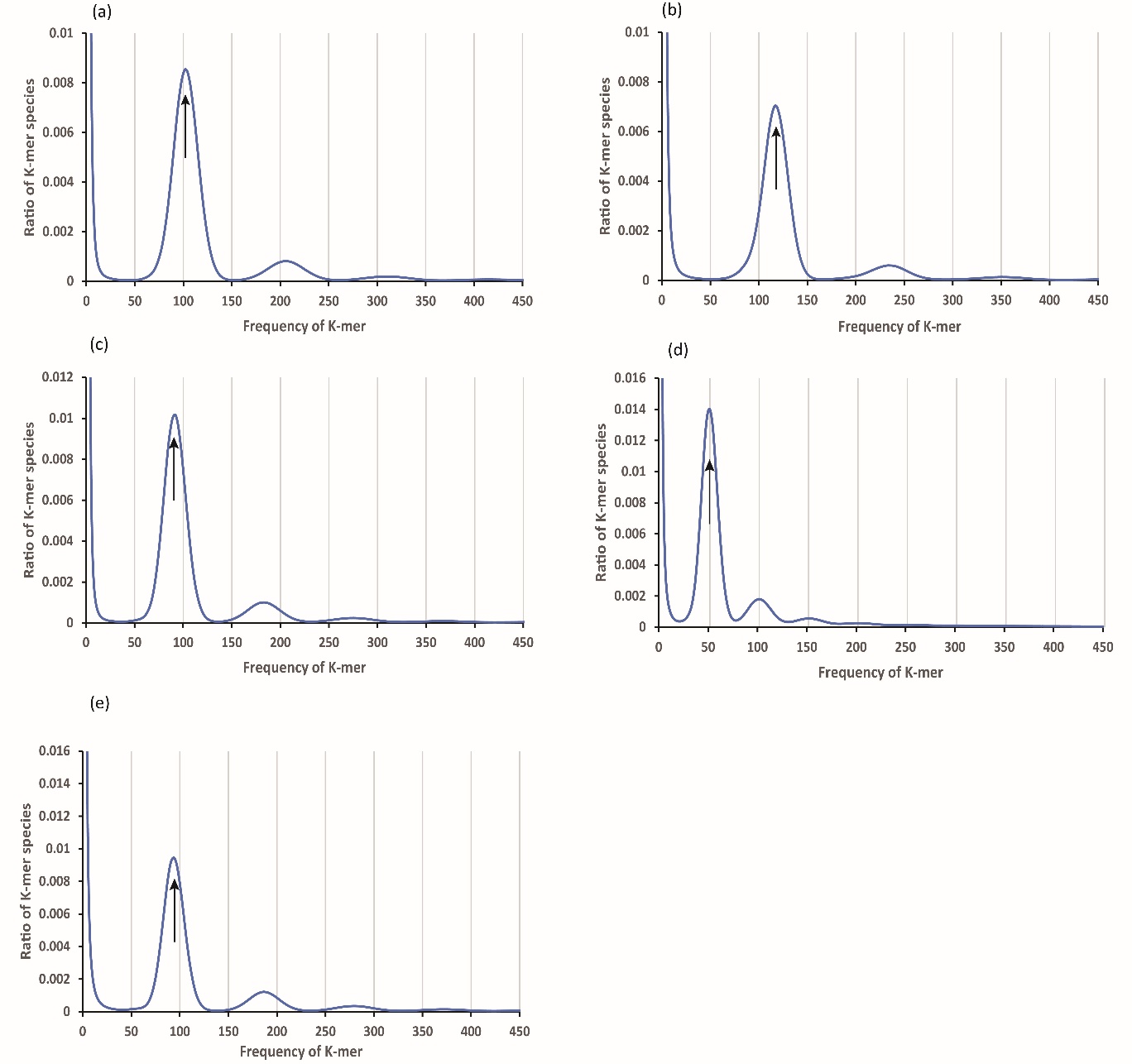
**Figure S1. Distribution of K-mer (K = 17) frequency in sequencing reads of the five Papilionoideae plants.** (a) *Canavalia gladiata*, (b) *Phaseolus coccineus*, (c) *Psophocarpus tetragonolobus*, (d) *Crotalaria pallida*, (e) *Clitoria ternatea.* The used genomic sequencing data are from PacBio-HiFi technology. The K-mer frequency peak with an arrow is the main peak. The K-mer distribution indicates that the five Papilionoideae plants are homozygous.


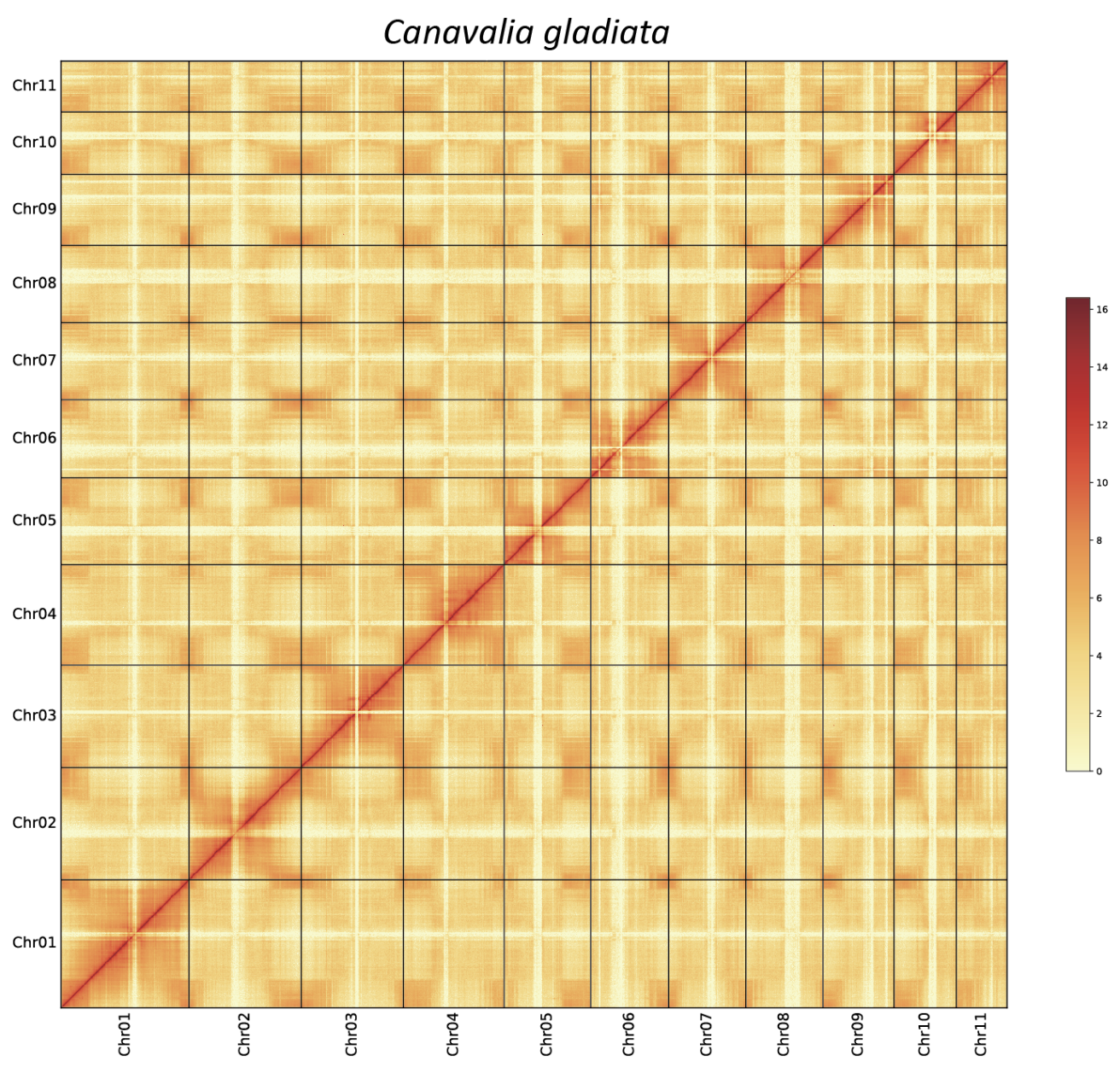


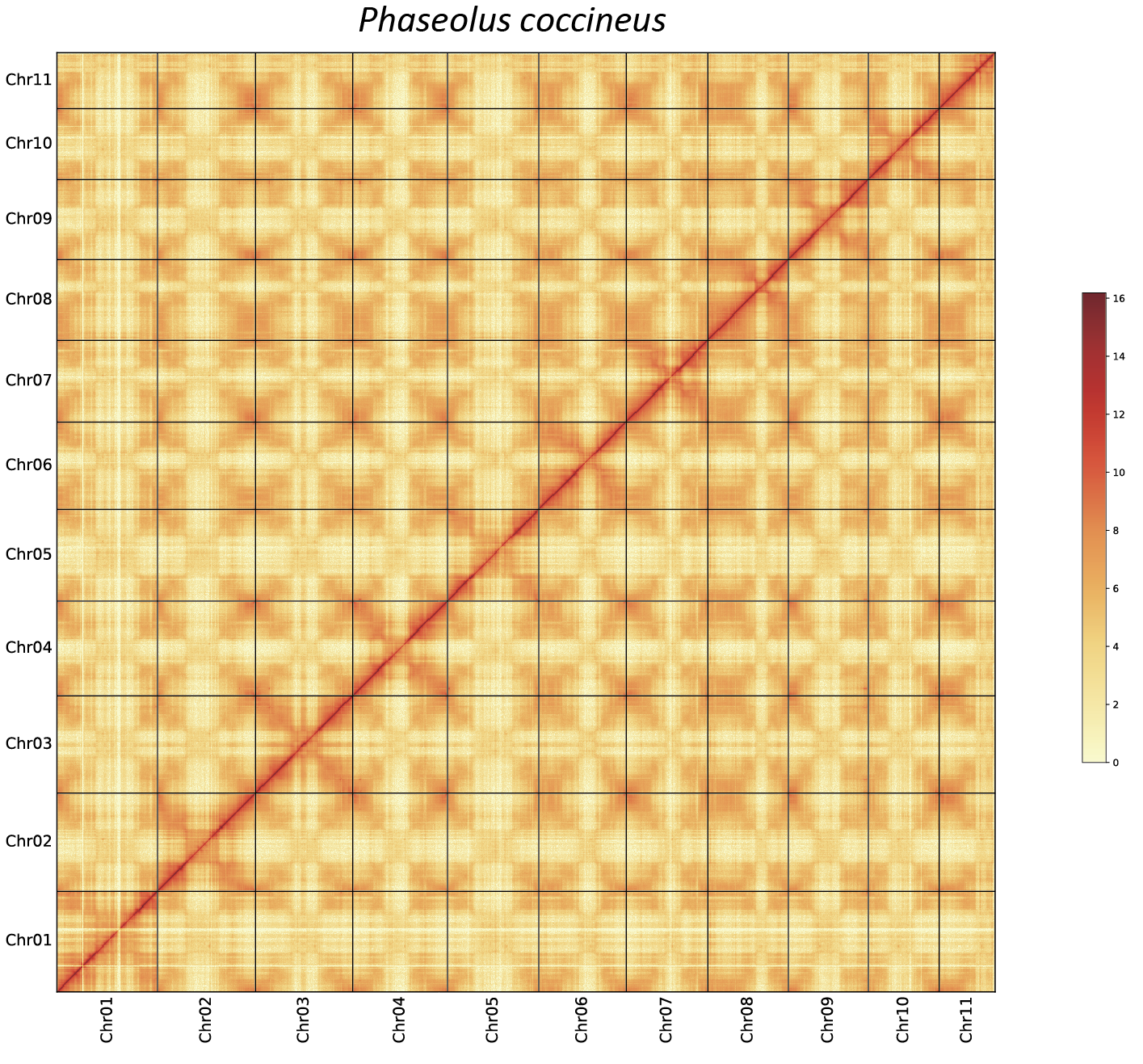


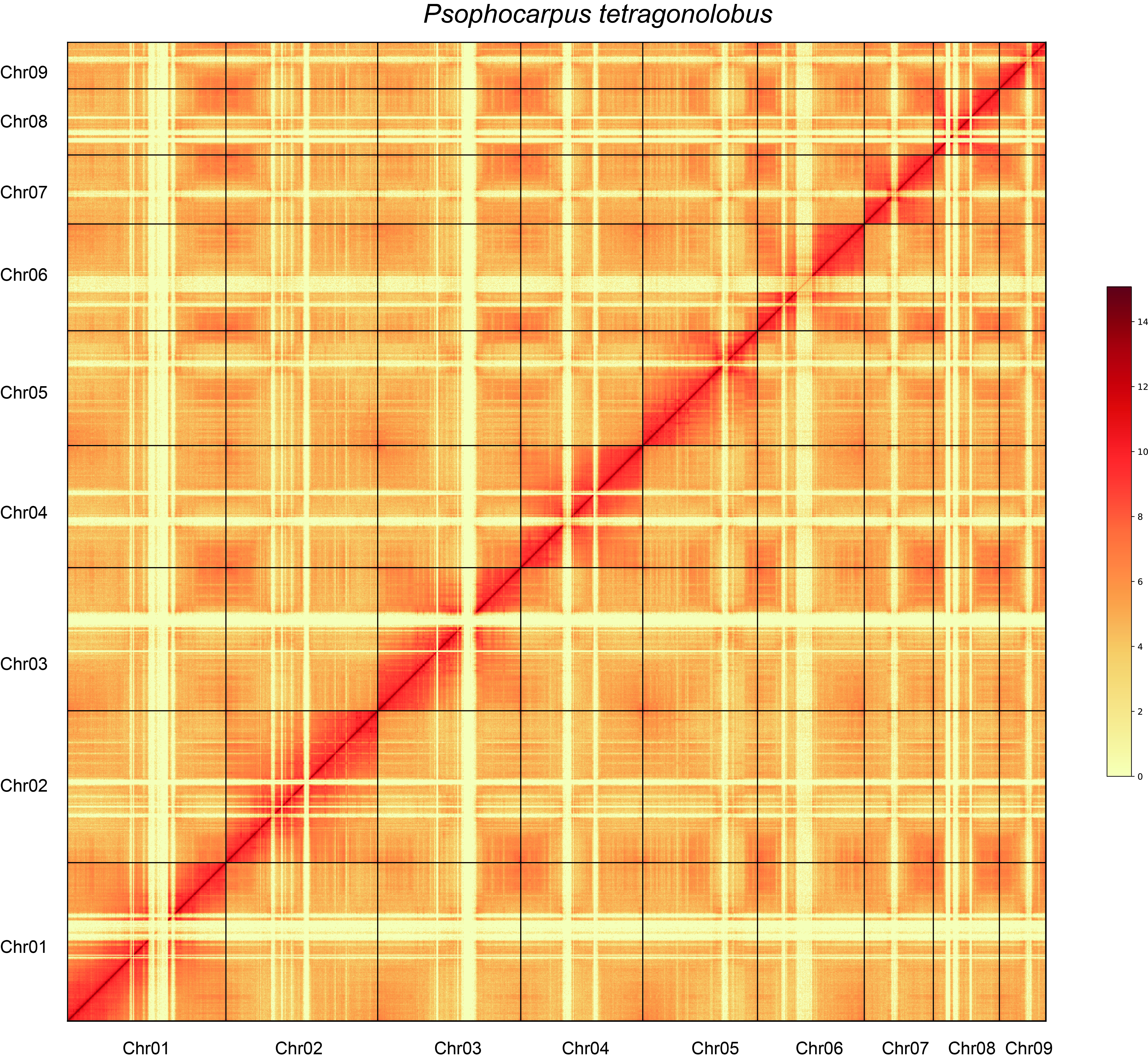


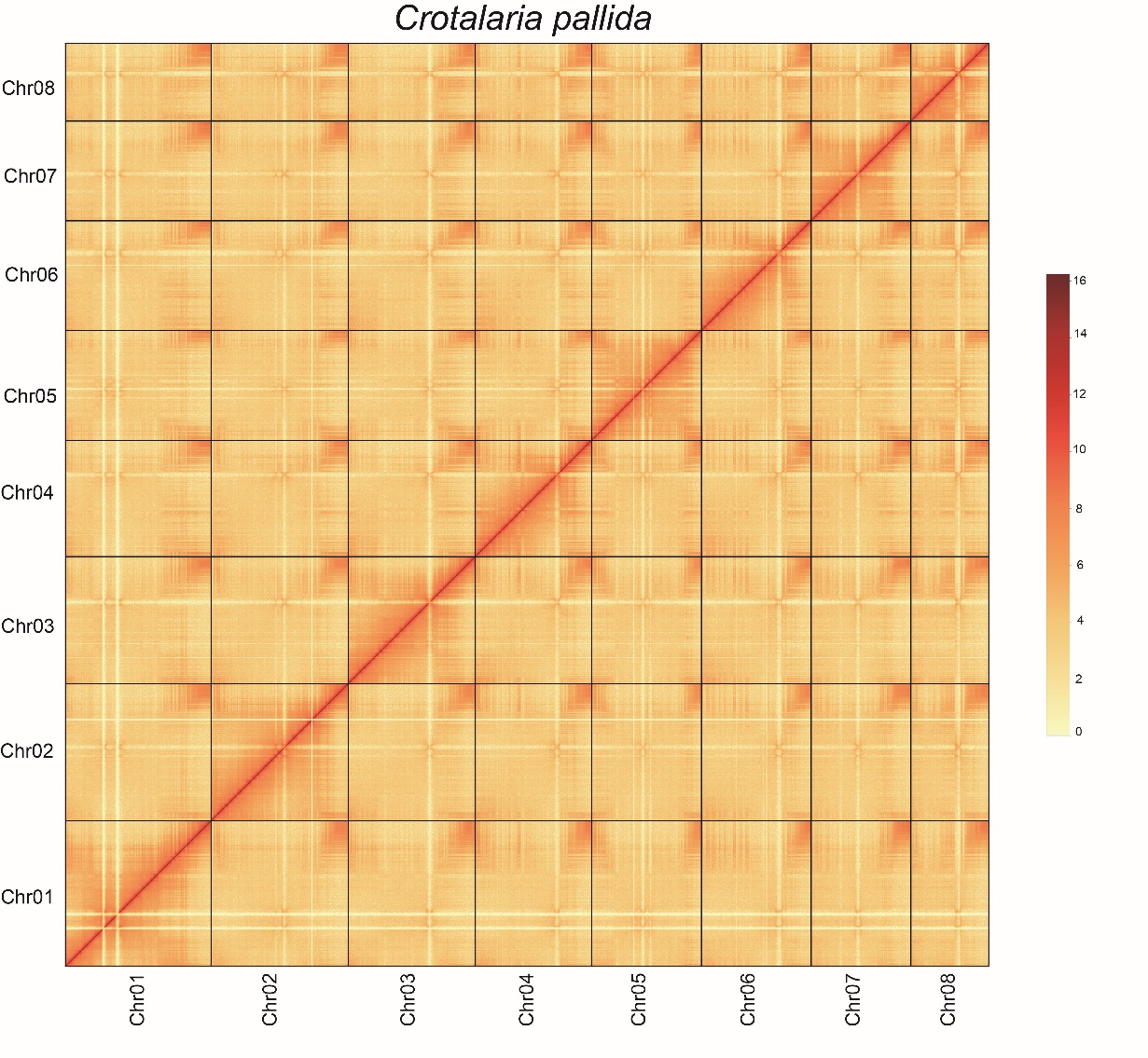


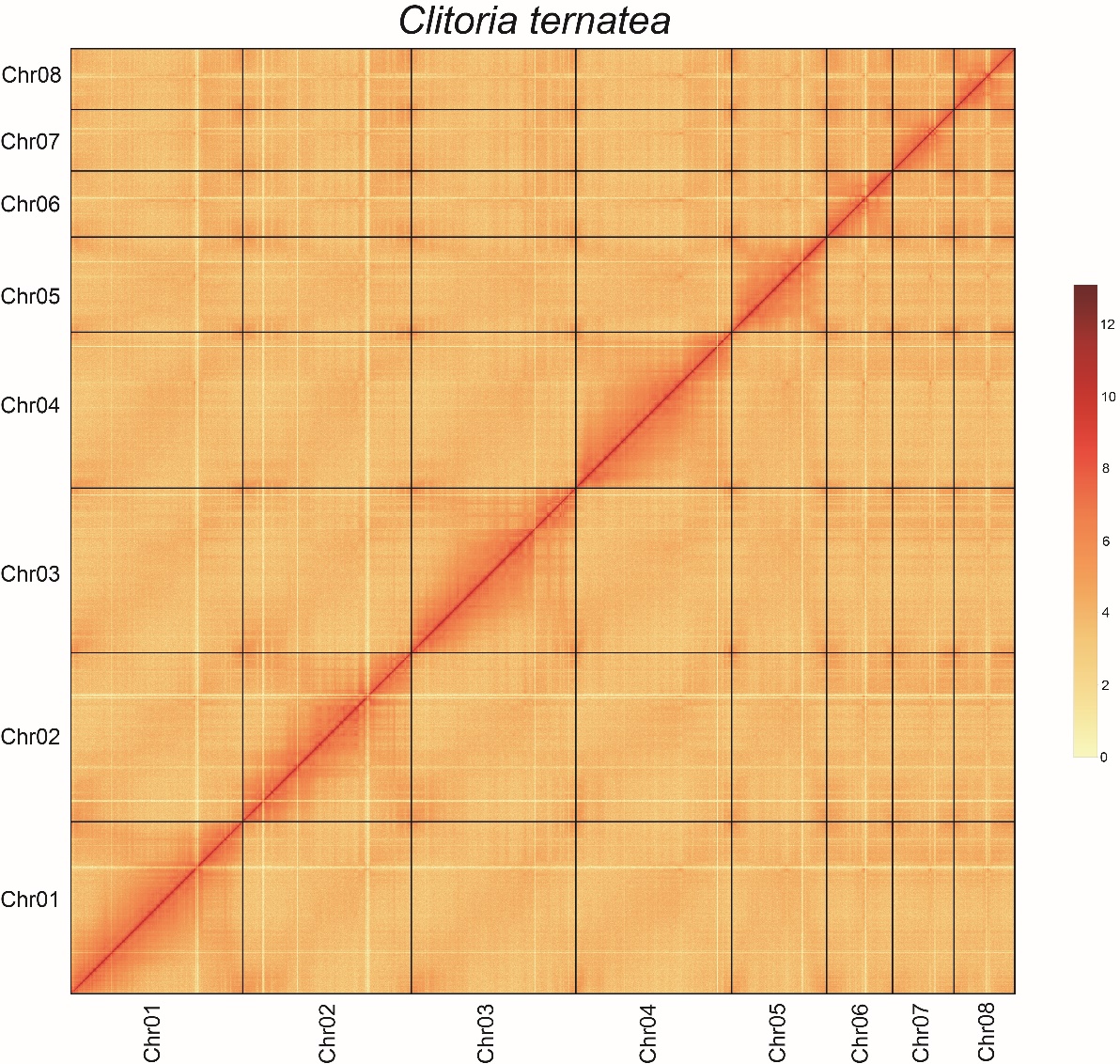
**Figure S2. Hi-C heatmap of chromosome assembly for five Papilionoideae plants.** The color represents Log2(Links number), and the resolution (bin size) is 500-Kb. The links number represents the count of Hi-C links that fall within the two analyzed genomic bins.

­


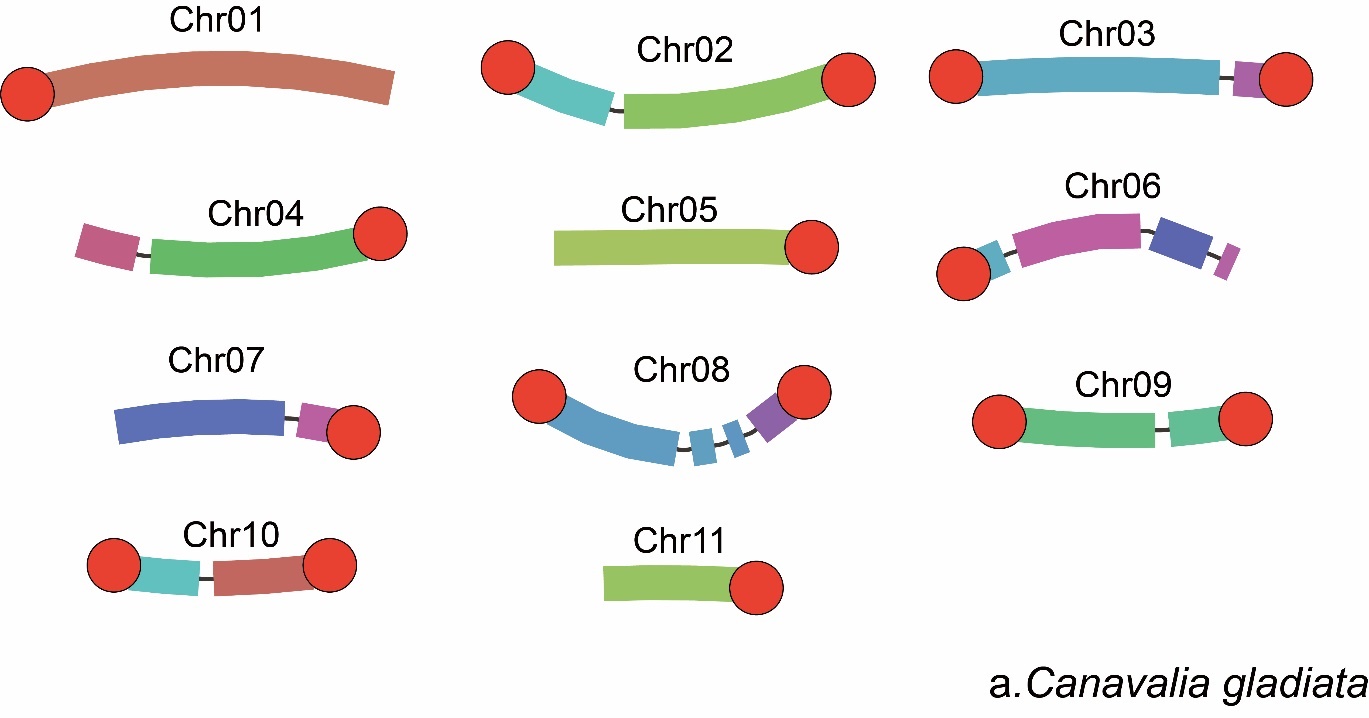


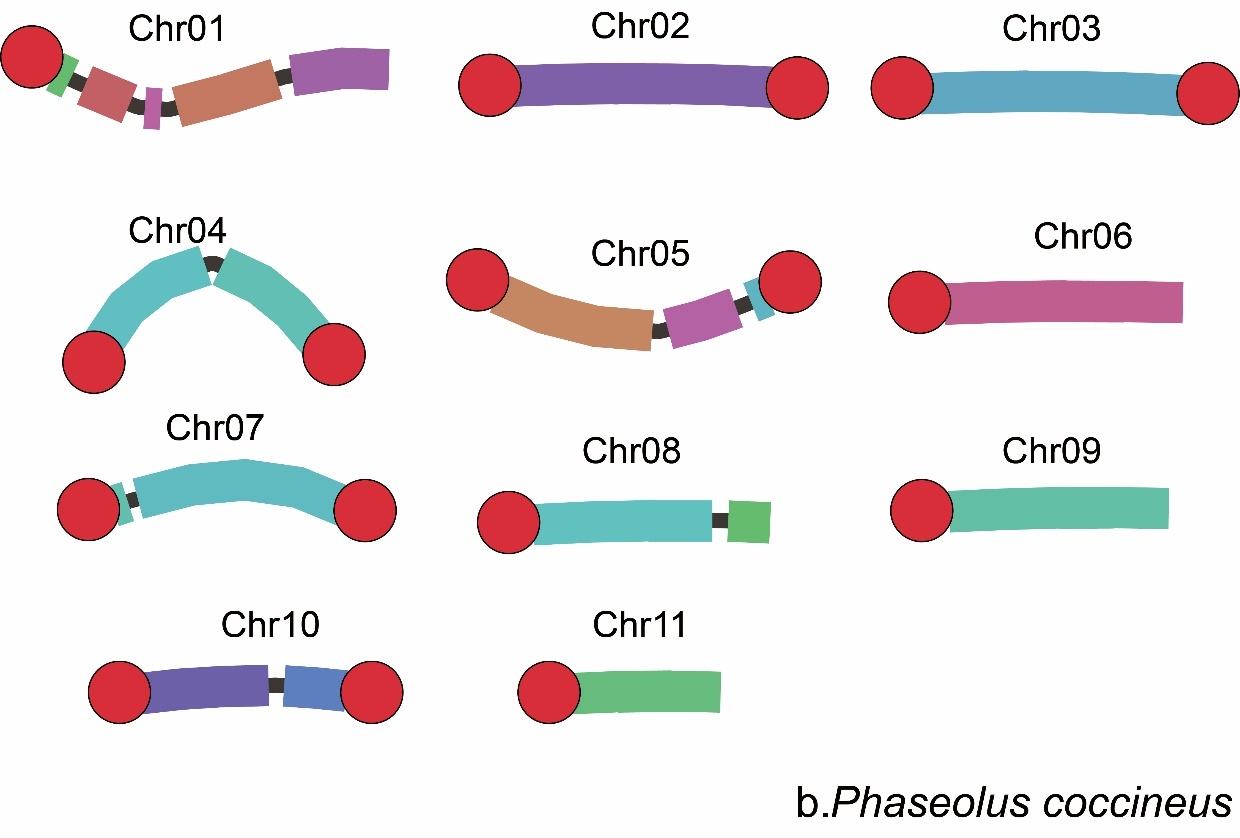


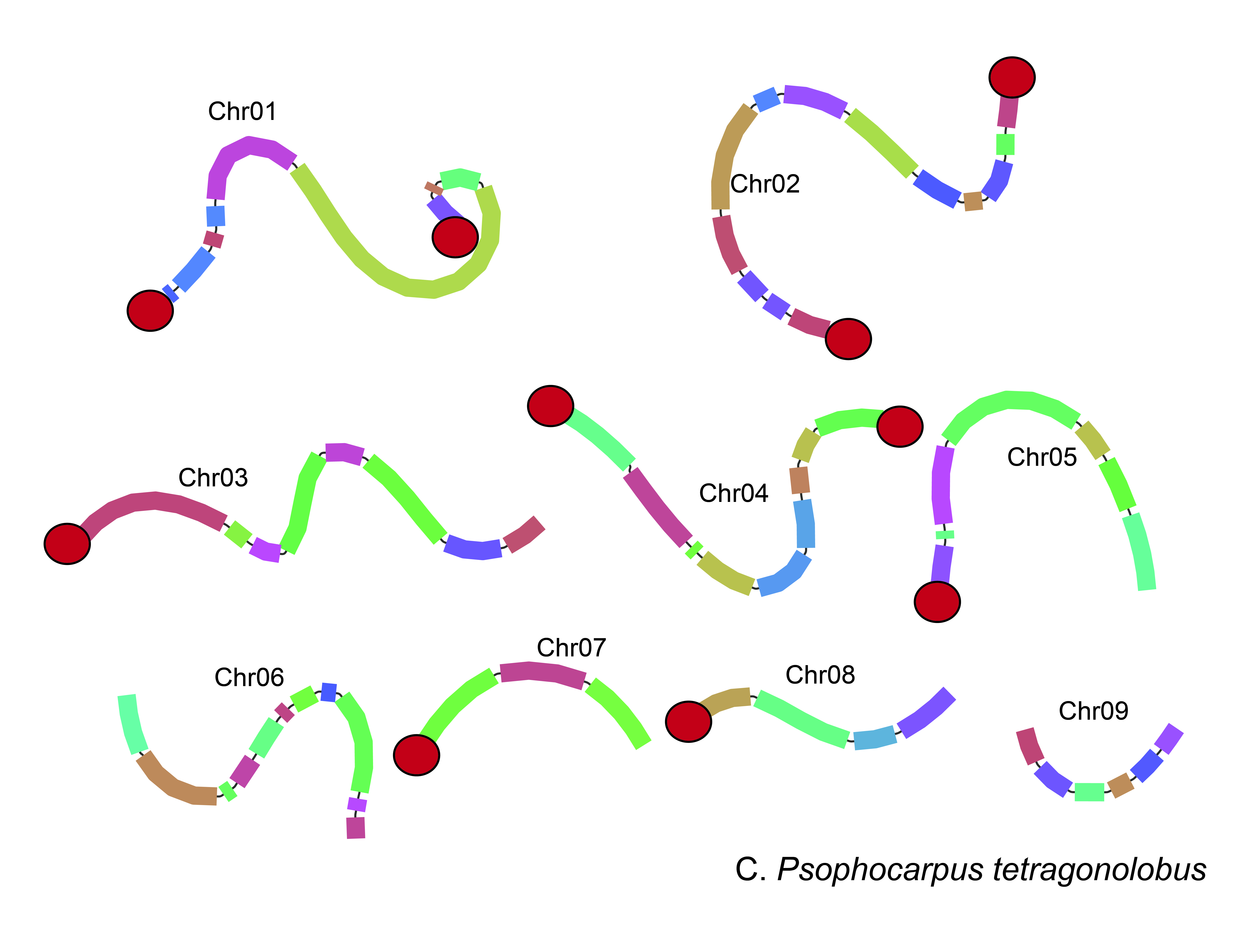


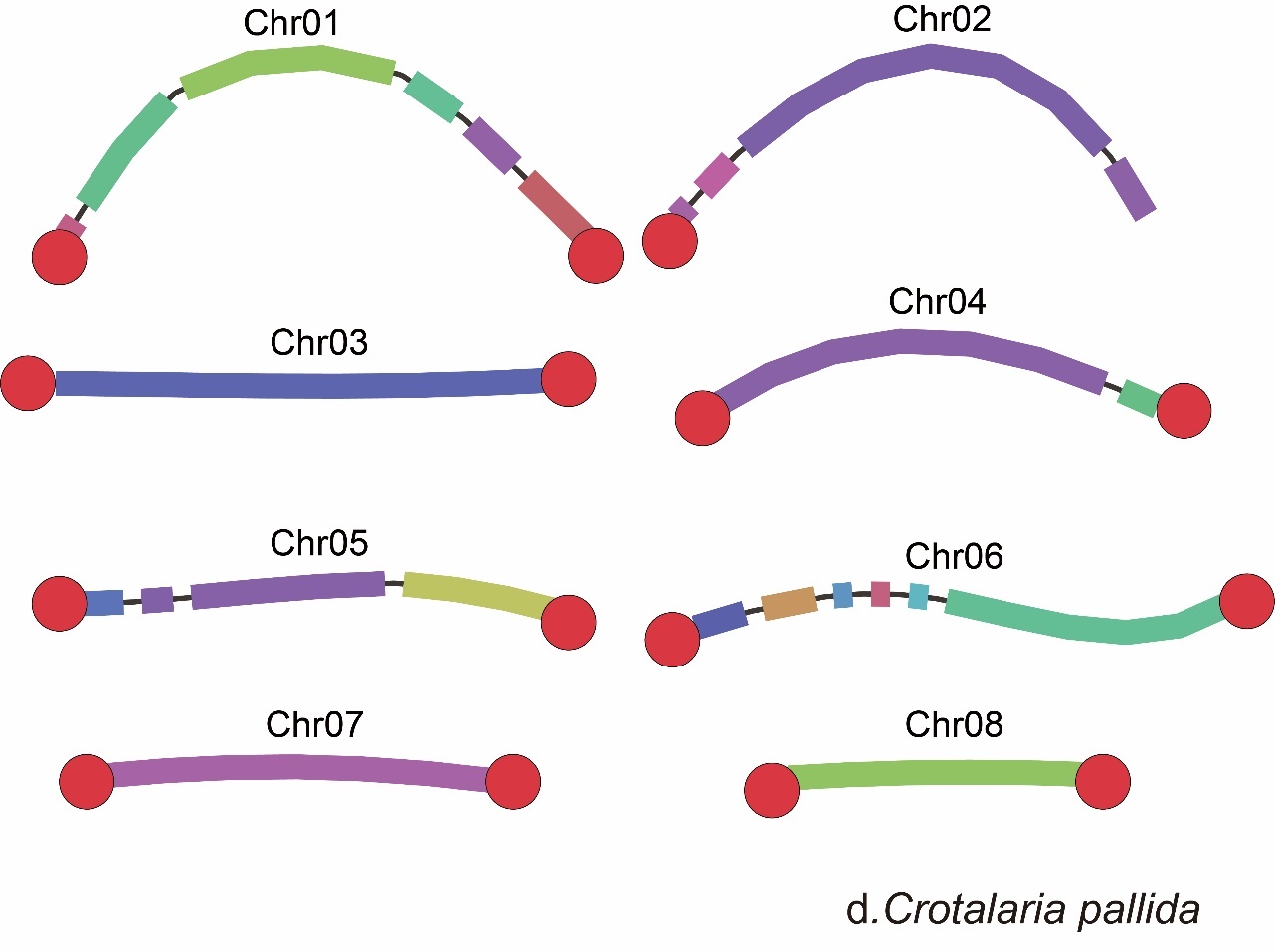


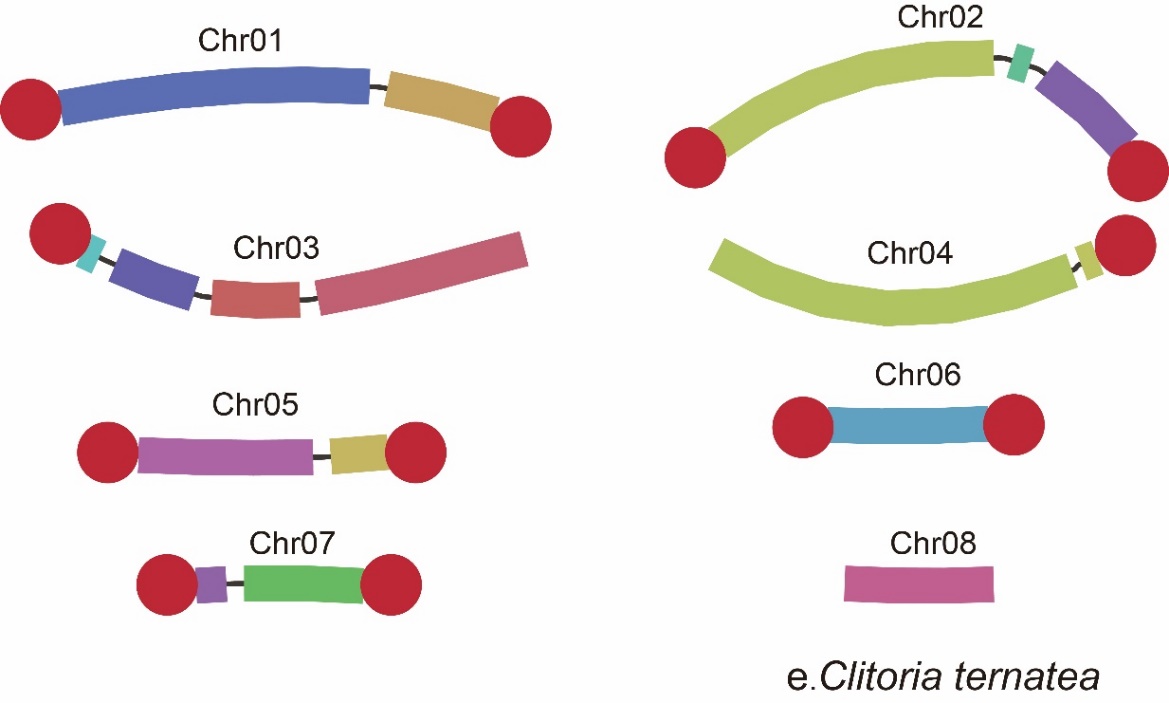


**Figure S****3**. **Bandage view of EndHiC scaffold results and the position of telomere for five Papilionoideae plants.** (a) *Canavalia gladiata*, (b) *Phaseolus coccineus*, (c) *Psophocarpus tetragonolobus*, (d) *Crotalaria pallida*, (e) *Clitoria ternatea.* The chromosome ends, assembled with telomere-specific tandem repeats (unit: TTTAGGG), were marked with a red circle.


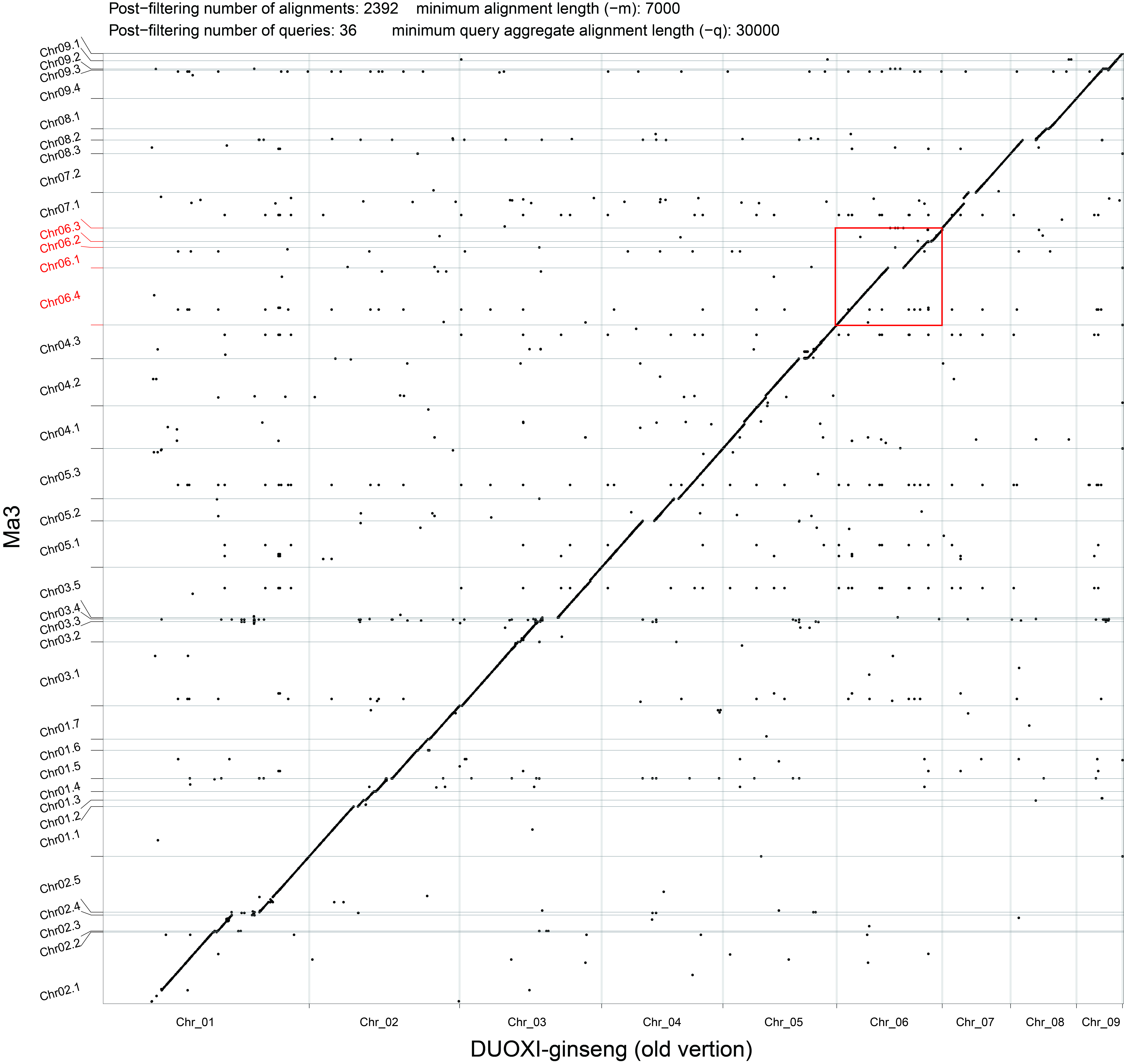


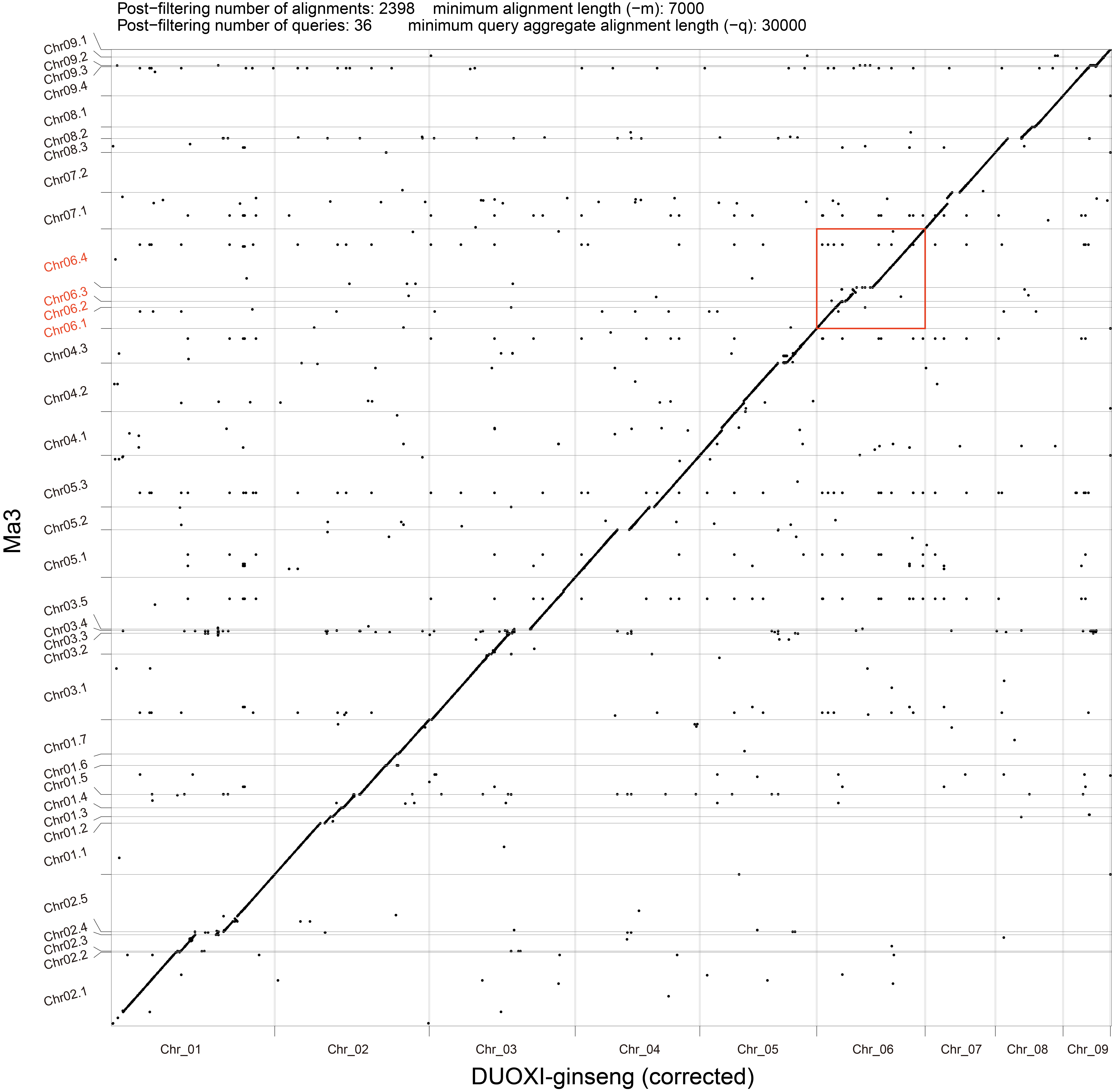


**Figure S4. Chromosomal collinear comparison between Ma3 and DUOXI-ginseng before and after correction.** The parameter for Minimap version 2.20 is set to “x asm5".


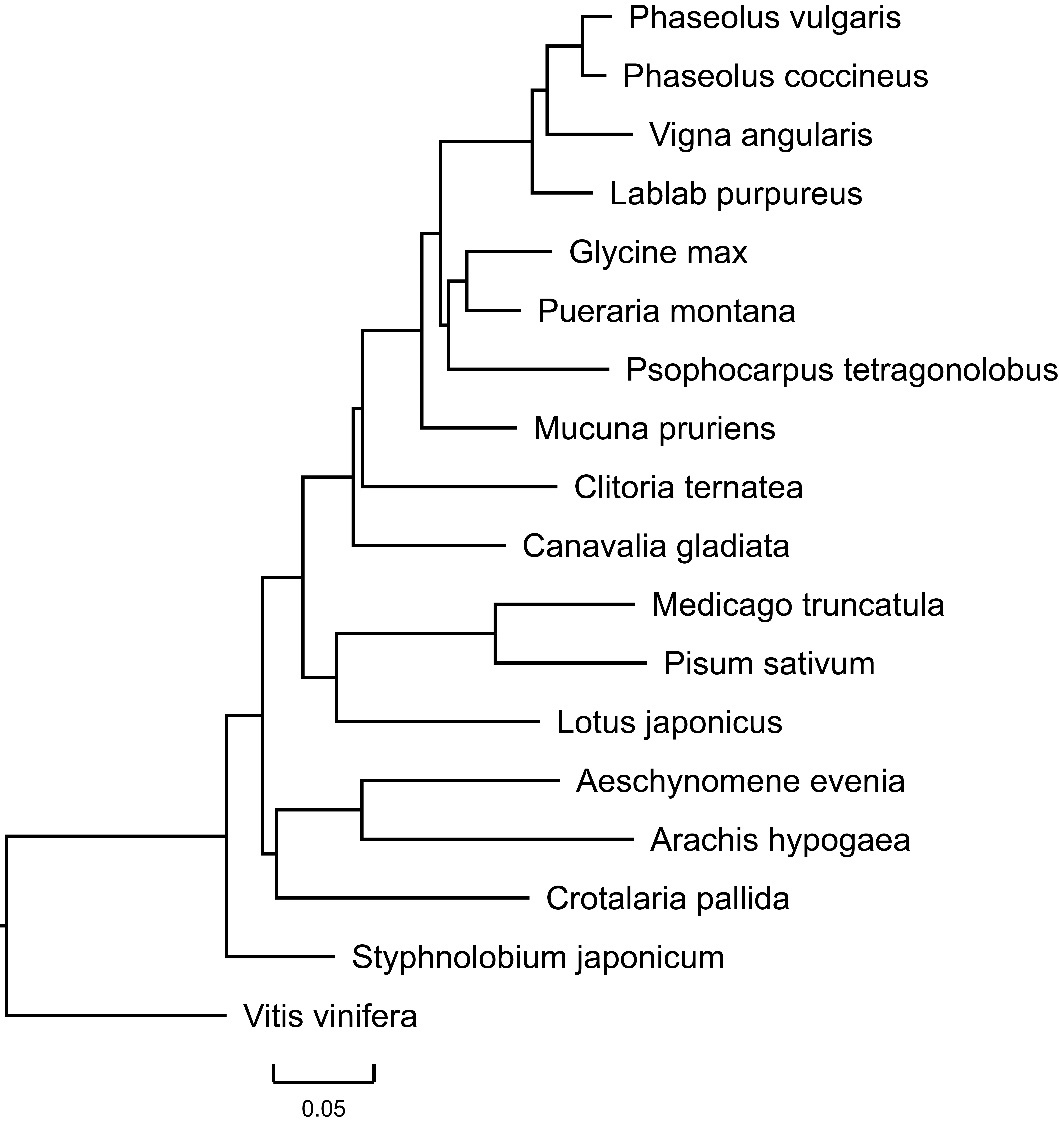


**Figure S5**. **Species tree of sequenced Papilionoideae species**. The tree was built by RAxML using concatenated cds sequences extracted from 405 near-single-copy orthogroups, in which all species have one gene copy except for Arachis hypogaea possesses two gene copies. One of the two gene copies in Arachis hypogaea was selected randomly. *Vitis vinifera* (grape) were employed as outgroups in this context. The bar represents substitutions per nucleotide site.


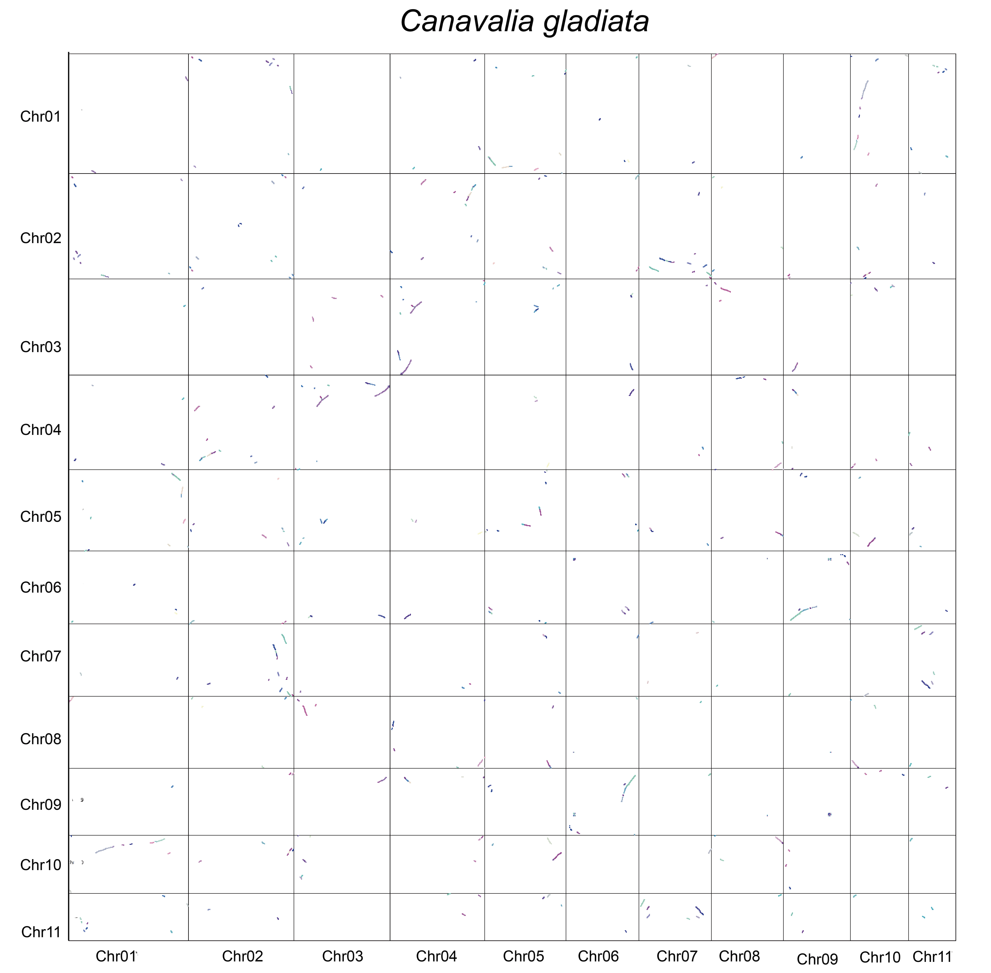


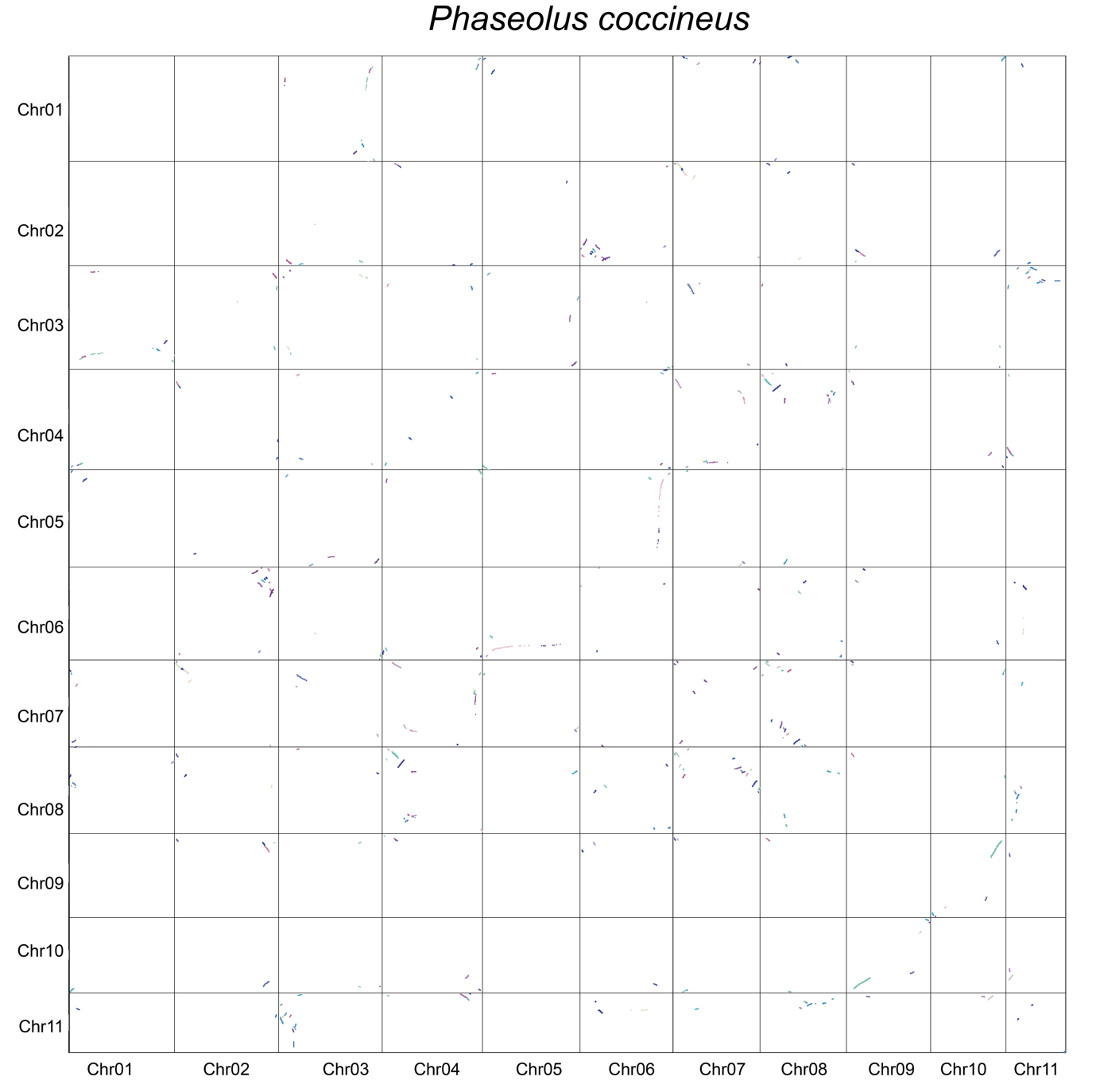


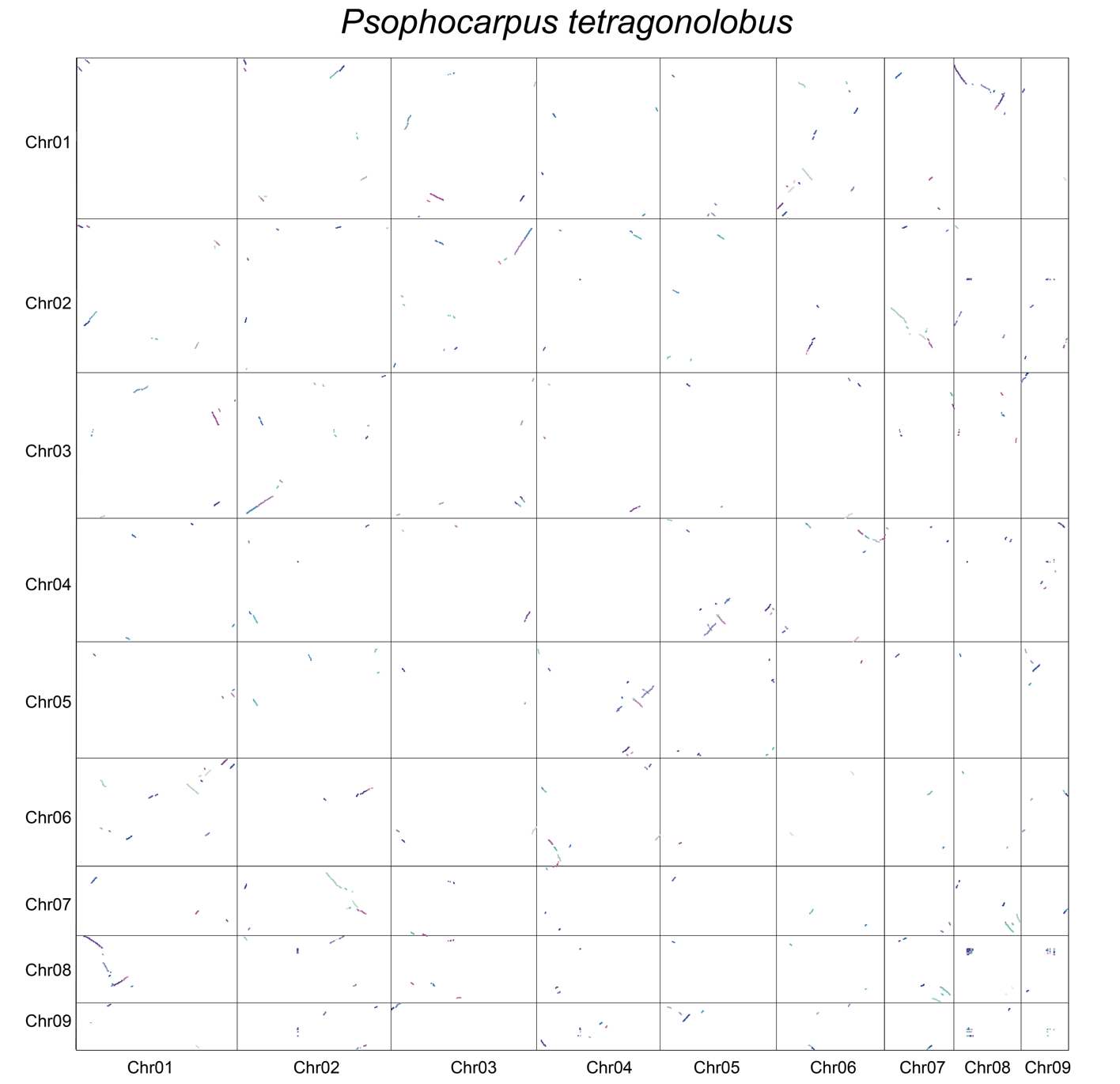


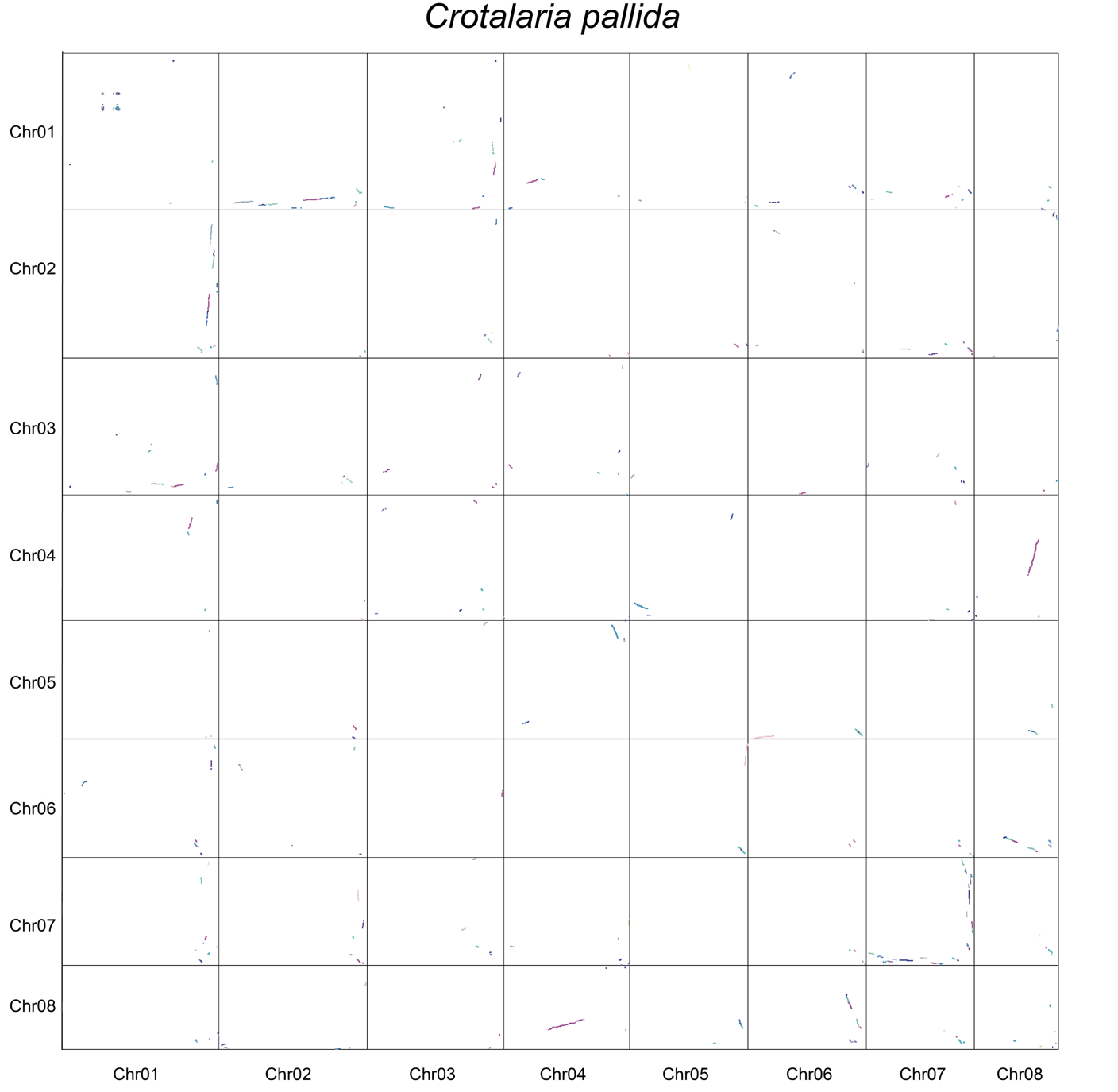


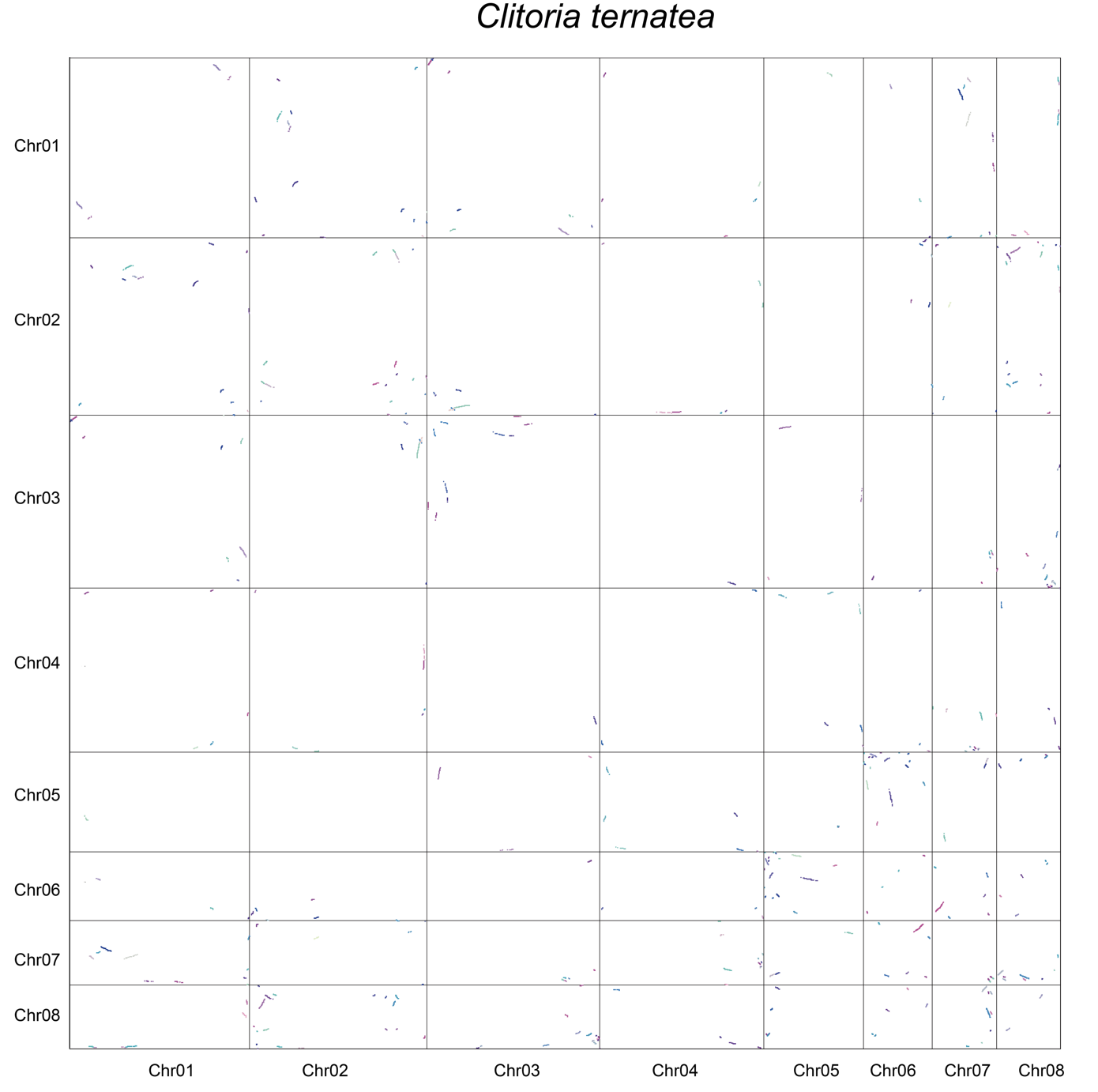


**Figure S6**. **Dot intra-species synteny Supplemental Figures for *C.gladiata*, *P.coccineus*, *P.tetragonolobus*, *C. pallida*, *C. ternatea***. The java program “dot_plotter” from the MCScanX package was used to draw colinear blocks with more than 10 syntenic gene pairs.


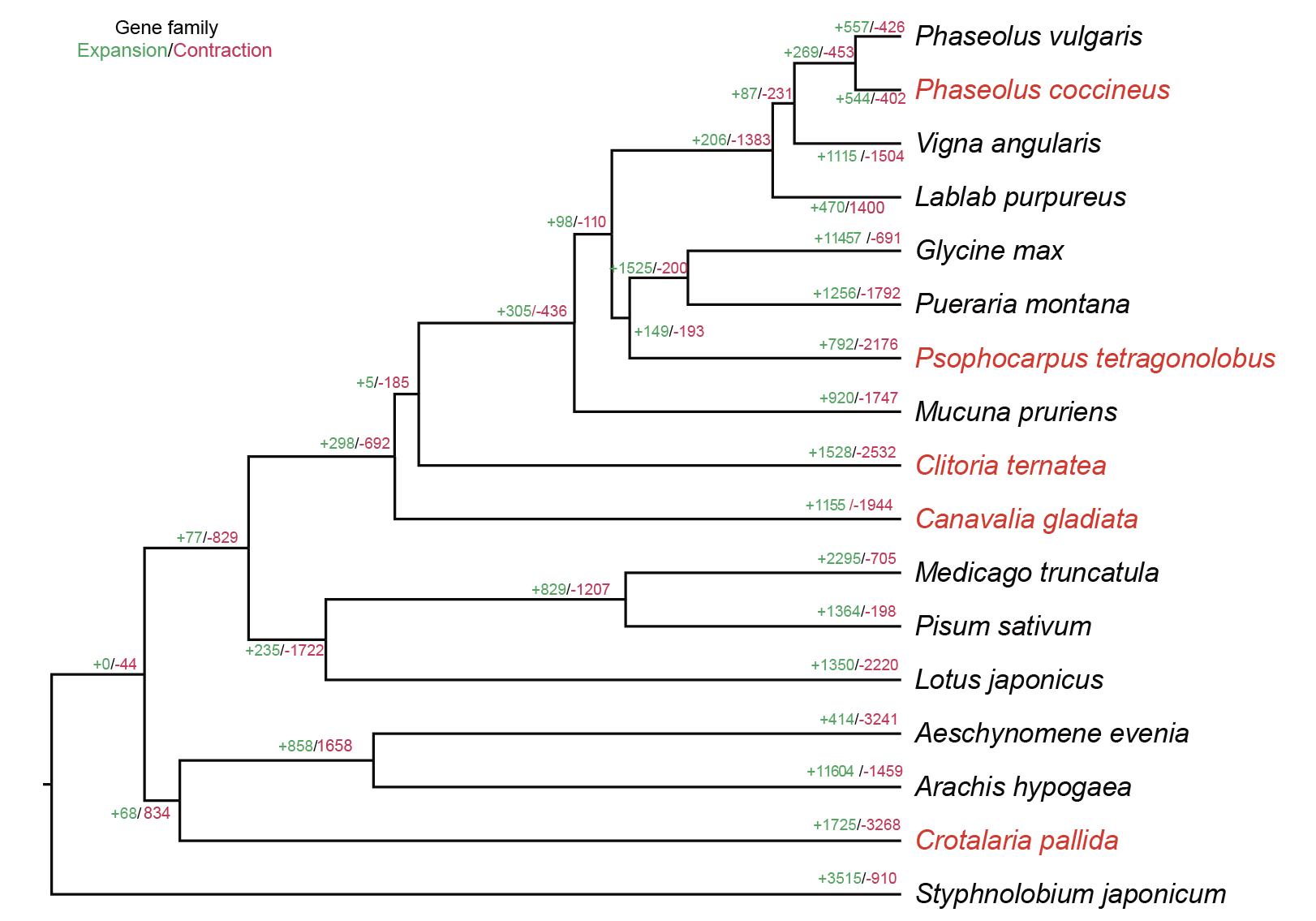
**Figure S7. Analysis of gene family (orthogroup) expansion and contraction was conducted using CAFÉ**. The numbers of expanded (“+”) and contracted (“-”) families on each branch are indicated on the phylogenetic timeline tree. The analysis utilized 31,643 OrthoFinder orthogroups (with a gene count of <= 200 and a species count of >= 2 and excluding families not present at the root) as input.

**
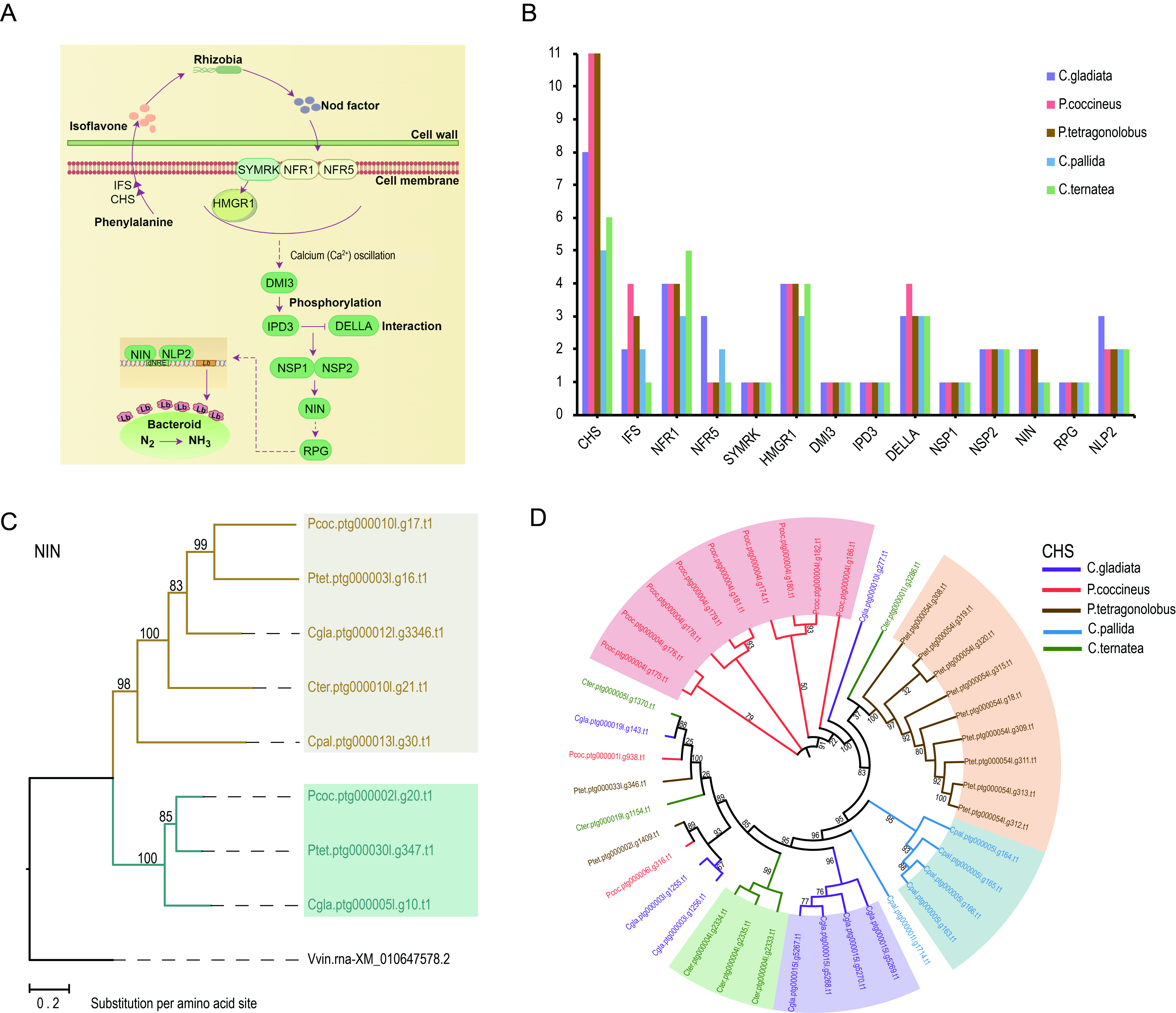
**

**Figure S8. Root nodulation symbiosis**. **(A)** Pathway of crucial genes involved in symbiotic nodulation and nitrogen fixation for Papilionoideae. The model figure is drawn using Figdraw. **(B)** The gene number of symbiotic pathway identified in *C.gladiata*, *P. coccineus*, *P.tetragonolobus*, *C.pallida*, and *C.ternatea*. **(C)** Gene tree for *NIN*. Members of the gene family were obtained from orthoFinder orthogroups, and the gene tree was constructed by FastTree. Bootstrap values are shown on each branch, and the two duplicated branches were highlighted. *V. vinifera* is utilized as the outgroup. **(D)** Gene tree for *CHS*, with similar style to *NIN*. The genes from the five sequenced species were shown in five different colors, and the tandem replicated genes of different species have been highlighted using distinct background colors.


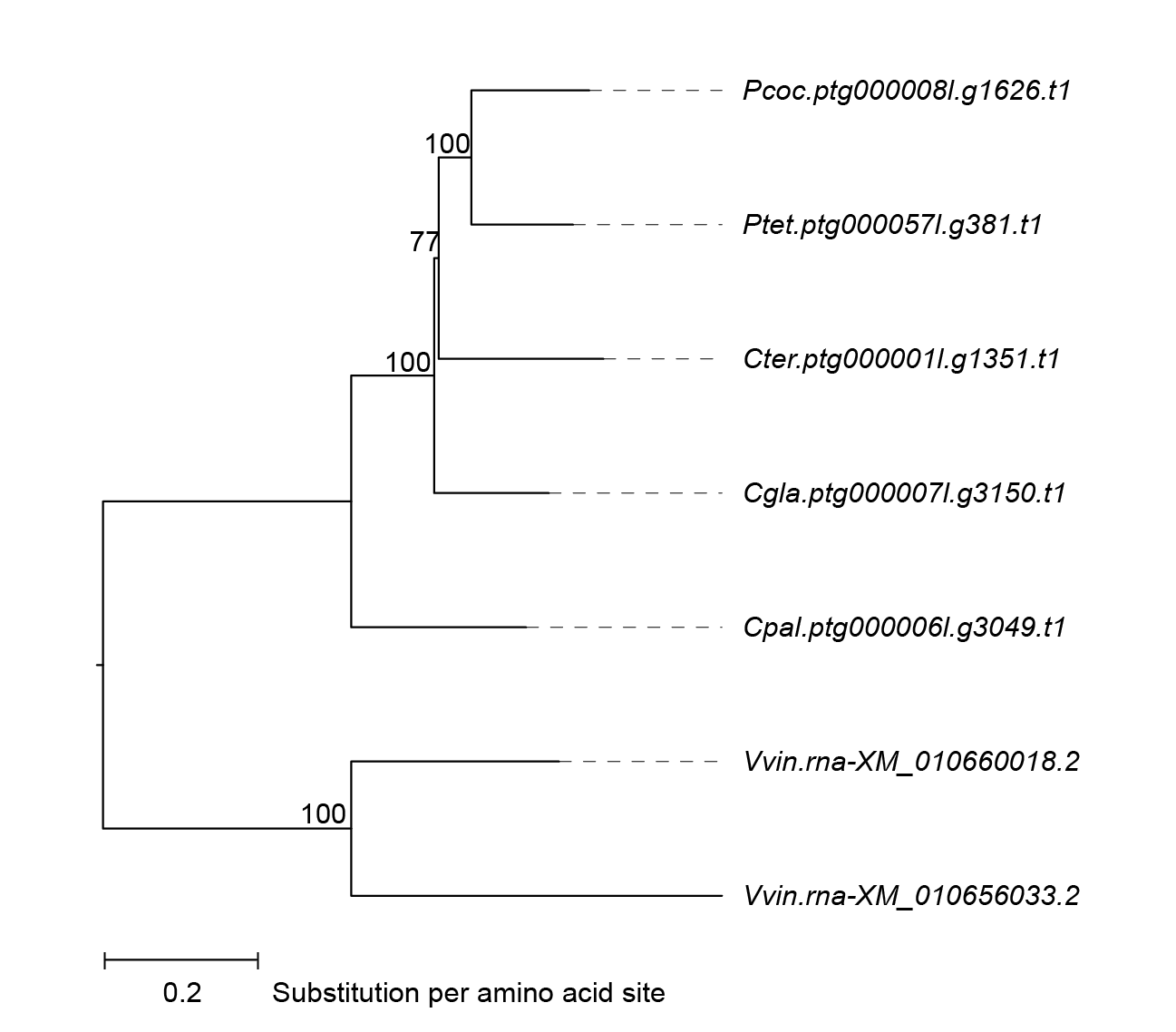
**Figure S9. Phylogenetic tree analysis of *RPG* genes.** A phylogenetic tree for the *RPG* genes was constructed using FastTree with protein sequences. All these genes belong to an orthogroup, and bootstrap values are indicated on each branch. The diagram involves six species, including *Canavalia gladiata* (*Cgla*), *Phaseolus coccineus* (*Pcoc*), *Psophocarpus tetragonolobus* (*Ptet*), *Crotalaria pallida* (*Cpal*), *Clitoria ternatea* (*Cter*) and *Vitis vinifera* (*Vvin*). *Vitis vinifera* (*Vvin*) is utilized as the outgroup.

**
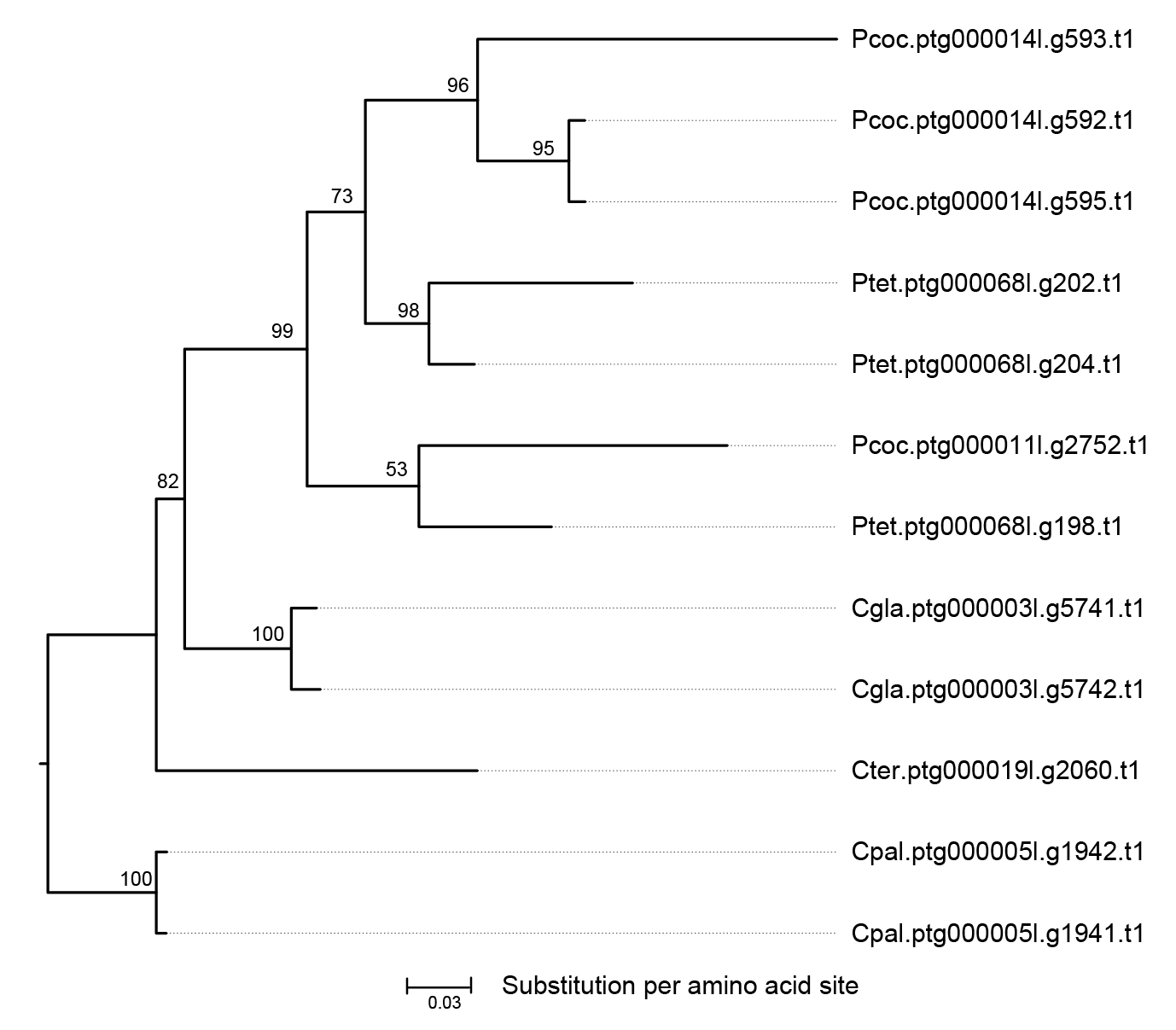
**

**Figure S10. Phylogenetic tree analysis of *IFS* genes.** A phylogenetic tree for the *IFS* genes was constructed using FastTree with protein sequences. All these genes belong to an orthogroup, and bootstrap values are indicated on each branch. The diagram involves five species, including *Canavalia gladiata* (*Cgla*), *Phaseolus coccineus* (*Pcoc*), *Psophocarpus tetragonolobus* (*Ptet*), *Crotalaria pallida* (*Cpal*), and *Clitoria ternatea* (*Cter*).


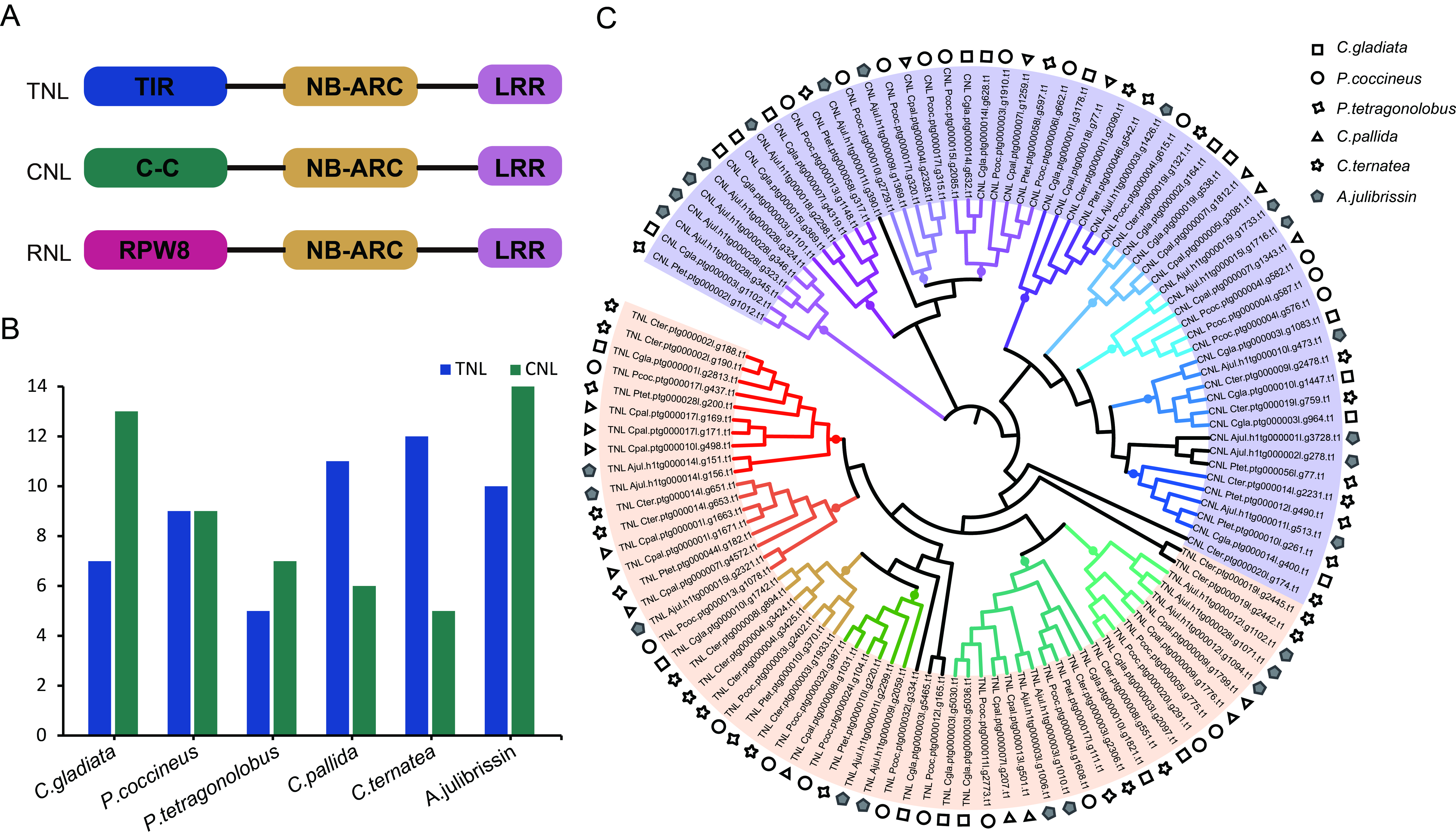


**Figure S11. Resistance (R) genes. (A)** Model figure for three types of R genes TNL, CNL and RNL. TNL consists of TIR, NB-ARC, and LRR domains, CNL consists of CC, NB-ARC, and LRR domains, while RNL consists of RPW8, NB-ARC, and LRR domains from N-terminal to C-terminal. **(B)** The number of CNL-type and TNL-type R genes in *C.gladiata*, *P. coccineus*, *P.tetragonolobus*, *C.pallida*, *C.ternatea* and *A.julibrissin* species, respectively. **(C)** The phylogenetic tree of all identified R genes in the 5 studied species and one outgroup (*A.julibrissin*). The two major branches TNL and CNL are labeled with different background colors, and a solid circle on the clade represents an OG, and the outgroup is marked with a gray graph. The R genes from various species are differentiated by various symbols.


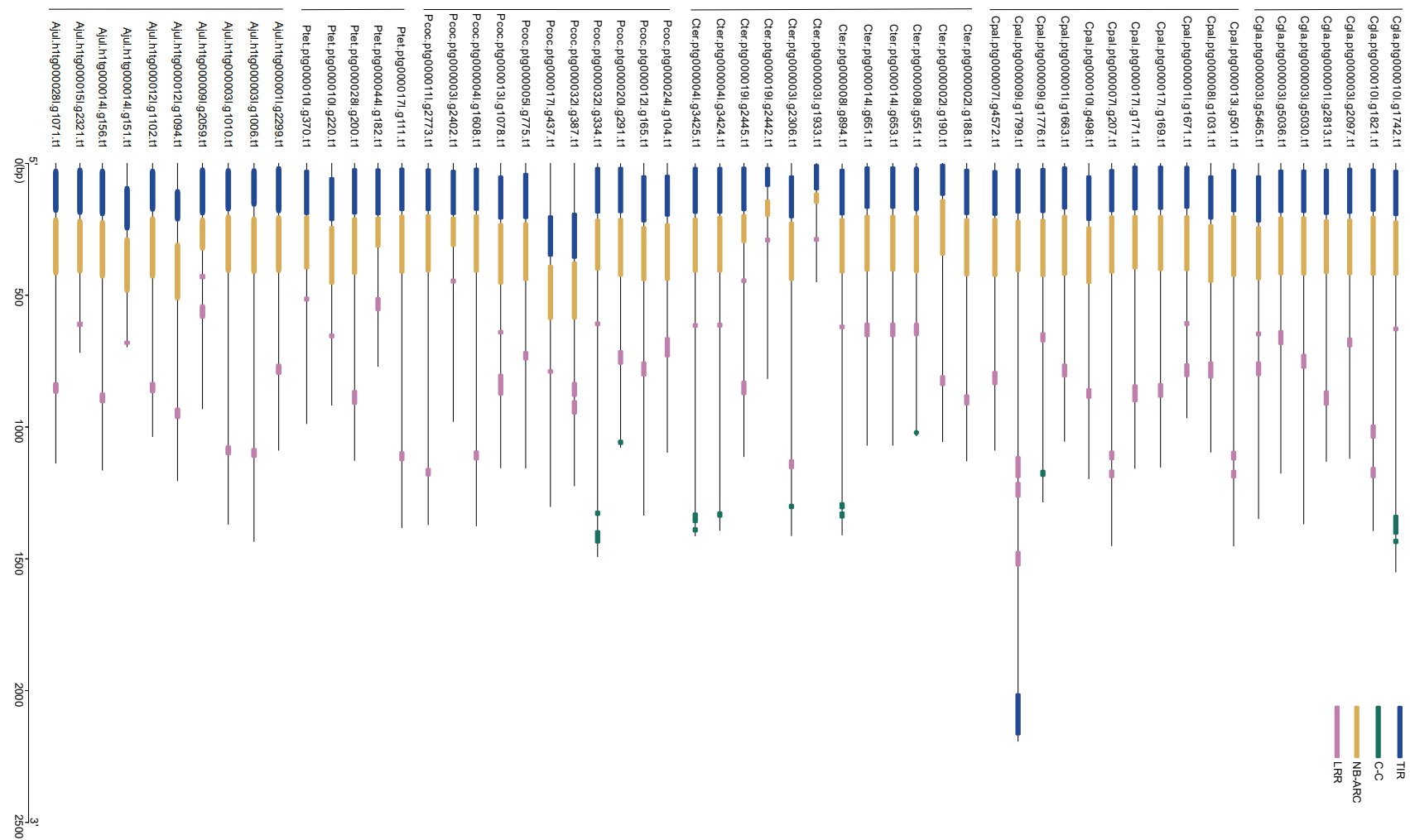


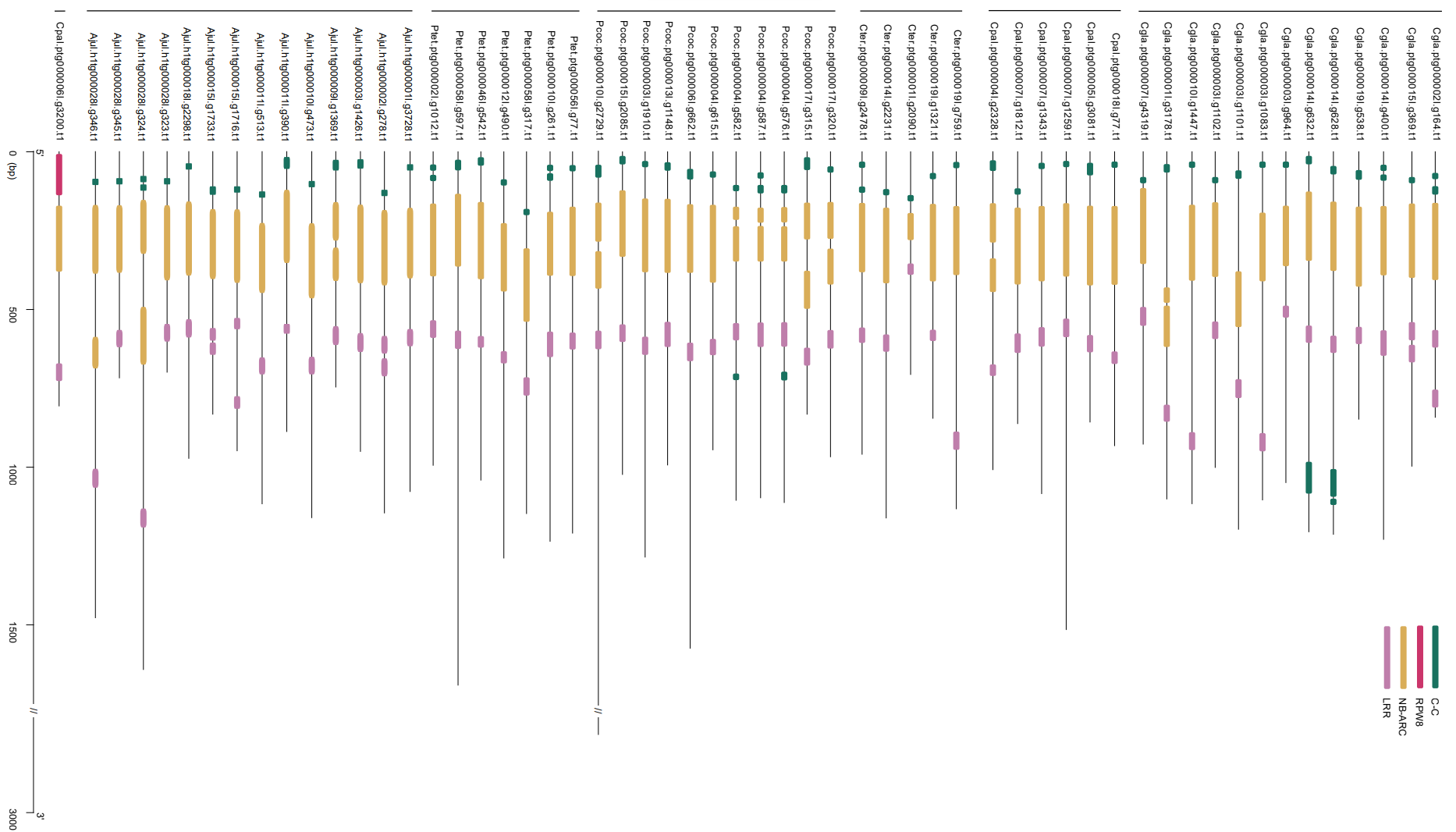


**Figure S12. The position of domains on the protein sequences of TNL-type, CNL-type and RNL-type R genes.** The diagram involves six species, including *Canavalia gladiata* (*Cgla*), *Phaseolus coccineus* (*Pcoc*), *Psophocarpus tetragonolobus* (*Ptet*), *Crotalaria pallida* (*Cpal*), *Clitoria ternatea* (*Cter*), and *Albizia julibrissin* (*Ajul*). The graphical display was generated using TBtools.

**Supplementary Tables**

**Table S1. Statistics of genome sequencing statistics.**

| Species | Genomic Hifi (CCS) | | Genomic HiC data | |
| --- | --- | --- | --- | --- |
|  | **reads** | **bases** | **reads** | **bases** |
| *Canavalia gladiata* | 5,254,356 | 67,153,848,829 | 544,673,832 | 81,053,025,114 |
| *Phaseolus coccineus* | 4,647,388 | 70,377,332,195 | 580,395,882 | 86,465,961,806 |
| *Psophocarpus tetragonolobus* | 4,919,291 | 63,520,379,127 | 525,419,080 | 78,102,069,218 |
| *Crotalaria pallida* | 3,559,622 | 68,697,592,772 | 836,931,218 | 125,315,752,681 |
| *Clitoria ternatea* | 10,758,736 | 167,098,573,949 | 775,610,084 | 115,837,330,827 |

**Table S2. Statistics of Hi-C data mapping to contigs for *C.gladiata*, *P.coccineus*, *P. tetragonolobus.***

| HiC pro results | *Canavalia gladiata* | | *Phaseolus coccineus* | | *Psophocarpus tetragonolobus* | |
| --- | --- | --- | --- | --- | --- | --- |
|  | **Reads number** | **Reads %** | **Reads number** | **Reads %** | **Reads number** | **Reads %** |
| Total pairs processed | 272,336,916 | 100.00% | 290,197,941 | 100.00% | 262,709,540 | 100.00% |
| Unmapped pairs | 9,098,332 | 3.34% | 7,455,639 | 2.57% | 3,527,288 | 1.34% |
| Low qual pairs | 107,954,672 | 39.64% | 149,817,489 | 51.63% | 109,659,386 | 41.74% |
| Pairs with singleton | 33,020,613 | 12.13% | 23,242,237 | 8.01% | 17,714,653 | 6.74% |
| Unique paired alignments | 122,263,299 | 44.89% | 109,682,576 | 37.80% | 131,808,213 | 50.17% |
| Valid interaction pairs | 97,740,294 | 35.89% | 88,828,009 | 30.61% | 109,721,108 | 41.77% |
| Dangling end pairs | 16,644,149 | 6.11% | 15,022,162 | 5.18% | 16,567,700 | 6.31% |
| Religation pairs | 745,414 | 0.27% | 661,362 | 0.23% | 277,559 | 0.11% |
| Self Cycle pairs | 961,908 | 0.35% | 1,225,058 | 0.42% | 953,743 | 0.36% |
| Filtered pairs | 6,138,593 | 2.25% | 3,916,741 | 1.35% | 4,255,551 | 1.62% |
| Dumped pairs | 32,941 | 0.01% | 29,244 | 0.01% | 32,552 | 0.01% |
| valid interaction rmdup | 51,559,442 | 18.93% | 58,354,680 | 20.11% | 57,990,563 | 22.07% |

Note: The statistical data are extracted from Hi-C pro result files: *.mpairstat, *.mRSstat, and * allValidPairs.mergestat. The term “valid interaction rmdup” denotes non-redundant and valid Hi-C read pairs, which were employed by EndHiC for the scaffolding process.

**Table S3. Statistics of Hi-C data mapping to contigs for *C.pallida*, *C.ternatea.***

| HiC pro results | *Crotalaria pallida* | | *Clitoria ternatea* | |
| --- | --- | --- | --- | --- |
|  | **Reads number** | **Reads %** | **Reads number** | **Reads %** |
| Total pairs processed | 418,465,609 | 100.00% | 387,805,042 | 100.00% |
| Unmapped pairs | 1,087,095 | 0.26% | 3,221,763 | 0.83% |
| Low qual pairs | 247,300,894 | 59.10% | 188,839,578 | 48.69% |
| Pairs with singleton | 10,787,880 | 2.58% | 36,855,118 | 9.50% |
| Unique paired alignments | 159,289,740 | 38.07% | 158,888,583 | 40.97% |
| Valid interaction pairs | 150,325,401 | 35.92% | 153,435,879 | 39.57% |
| Dangling end pairs | 6,604,619 | 1.58% | 2,577,822 | 0.66% |
| Religation pairs | 1,458,955 | 0.35% | 283,368 | 0.07% |
| Self Cycle pairs | 90,310 | 0.02% | 122,164 | 0.03% |
| Filtered pairs | 808,853 | 0.19% | 2,468,872 | 0.64% |
| Dumped_pairs | 1,602 | 0.00% | 478 | 0.00% |
| Valid_interaction_rmdup | 147,331,145 | 35.21% | 123,100,374 | 31.74% |

Note: The statistical data are extracted from Hi-C pro result files: *.mpairstat, *.mRSstat, and * allValidPairs.mergestat. The term “valid interaction rmdup” denotes non-redundant and valid Hi-C read pairs, which EndHiC used for the scaffolding process.

**Table S4. Statistics of reference organelle genomes used for filtering sequence from the organelle.**

| Organelle genomes | Length (bp) | NCBI accession |
| --- | --- | --- |
| *Canavalia gladiata* chloroplast | 157,923 | MT922037.1 |
| *Phaseolus vulgaris* mitochondrion | 395,782 | CM055725.1 |
| *Phaseolus vulgaris* chloroplast | 150,284 | EU196765.1 |
| *Psophocarpus tetragonolobus* chloroplast | 151,571 | NC_057457.1 |
| *Crotalaria pallida* chloroplast | 152,658 | NC_053562.1 |
| *Clitoria ternatea* chloroplast | 151,673 | NC_047365.1 |

**Table S5. Statistics of genome assembly.**

| Species | Total contig size （bp) | Contig N50 size （bp) | Contig N90 size （bp) | Total scaffold size （bp) | Scaffold N50 size （bp) | Scaffold N90 size （bp) | % anchored to chromosomes | BUSCO results with database embryophyta_odb10 |
| --- | --- | --- | --- | --- | --- | --- | --- | --- |
|  |  |  |  |  |  |  |  |  |
| *Canavalia gladiata* | 619,186,046 | 39,462,069 | 13,049,192 | 619,198,046 | 55,284,388 | 39,687,968 | 97.49% | C:99.4%[S:96.5%,D:2.9%] |
| *Phaseolus coccineus* | 592,734,161 | 39,559,522 | 13,867,545 | 592,744,161 | 52,871,251 | 33,944,536 | 95.65% | C:99.3%[S:96.6%,D:2.7%] |
| *Psophocarpus tetragonolobus* | 712,813,888 | 13,237,817 | 3,743,186 | 712,874,888 | 83,509,198 | 31,692,552 | 93.79% | C:99.2%[S:96.5%,D:2.7%] |
| *Crotalaria pallida* | 1,217,645,575 | 100,840,643 | 18,636,630 | 1,217,662,575 | 150,534,199 | 100,840,643 | 98.20% | C:99.0%[S:93.9%,D:5.1%] |
| *Clitoria ternatea* | 1,724,627,994 | 159,058,970 | 60,804,403 | 1,724,636,994 | 293,041,693 | 109,104,625 | 97.47% | C:99.0%[S:94.6%,D:4.4%] |

Note: The term “% anchored to chromosomes” represents the percentage of contig sequences assembled into scaffolds at the chromosome level. In BUSCO results, “C” represents a complete gene, “S” represents a single-copy gene, and “D” represents a duplicated gene. Both “S” and “D” correspond to complete genes.

**Table S6. Statistics of tandem repeats annotation.**

| Species | Total contig size (bp) | Total TR size (bp) | TR rate % | TR N50 size (bp) |
| --- | --- | --- | --- | --- |
| *Canavalia gladiata* | 619,186,046 | 105,656,450 | 17.06% | 1,911 |
| *Phaseolus coccineus* | 592,734,161 | 54,428,626 | 9.18% | 361 |
| *Psophocarpus tetragonolobus* | 712,813,888 | 134,417,242 | 18.86% | 38,568 |
| *Crotalaria pallida* | 1,217,645,575 | 119,729,388 | 9.83% | 9,431 |
| *Clitoria ternatea* | 1,724,627,994 | 130,389,725 | 7.56% | 339 |

Note: TR represents “tandem repeat”.

**Table S7. Statistics of full-length cDNA mapping.**

| Species | CCS reads number | Isoseq3 non-redundant isoforms | Ratio in total | Gmap  +FilterPSL (best,minId=95,  minCover=95) | Ratio in total |
| --- | --- | --- | --- | --- | --- |
|  |  |  |  |  |  |
| *Canavalia gladiata* | 649,210 | 64,142 | 9.88% | 62,790 | 9.67% |
| *Phaseolus coccineus* | 456,451 | 45,395 | 9.95% | 44,779 | 9.81% |
| *Psophocarpus tetragonolobus* | 667,611 | 58,222 | 8.72% | 56,776 | 8.50% |
| *Crotalaria pallida* | 874,562 | 87,294 | 9.98% | 86,320 | 9.87% |
| *Clitoria ternatea* | 691,787 | 55,735 | 8.06% | 55,103 | 7.97% |

Note: Isoseq3 is a tool package developed by PacificBiosciences. FilterPSL, a script from the Augustus package, was employed with the parameters “--best --minId=95 --minCover=95” here.

**Table S8. Statistics of Exonerate homology predictions.**

| using species | *Canavalia gladiata* | *Phaseolus coccineus* | *Psophocarpus tetragonolobus* | *Crotalaria pallida* | *Clitoria ternatea* |
| --- | --- | --- | --- | --- | --- |
| *Aeschynomene evenia* | 16,521 | 15,548 | 15,352 | 16,007 | 15,984 |
| *Arachis hypogaea* | 33,523 | 31,480 | 31,167 | 32,710 | 32,610 |
| *Glycine max* | 39,966 | 41,163 | 40,811 | 34,822 | 38,313 |
| *Lablab purpureus* | 21,567 | 23,431 | 21,654 | 18,949 | 20,712 |
| *Lotus japonicus* | 20,286 | 19,335 | 18,957 | 19,475 | 19,868 |
| *Medicago truncatula* | 25,086 | 23,465 | 23,225 | 23,370 | 24,066 |
| *Mucuna pruriens* | 22,875 | 22,908 | 22,513 | 19,810 | 21,719 |
| *Phaseolus vulgaris* | 21,869 | 26,119 | 22,112 | 18,931 | 20,749 |
| *Pisum sativum* | 21,344 | 20,229 | 20,166 | 20,581 | 21,044 |
| *Pueraria montana* | 24,666 | 25,166 | 24,903 | 21,285 | 23,258 |
| *Styphnolobium japonicum* | 23,734 | 22,068 | 21,895 | 22,627 | 22,743 |
| *Vigna angularis* | 20,655 | 23,351 | 20,850 | 17,897 | 19,801 |

Note: The protein sequences of *Aeschynomene evenia, Arachis hypogaea, Glycine max, Lablab purpureus, Lotus japonicus, Medicago truncatula, Mucuna pruriens, Phaseolus vulgaris, Pisum sativum, Pueraria montana, Styphnolobium japonicum, Vigna angularis* were mapped to the reference genomes of *Canavalia gladiate, Phaseolus coccineus, Psophocarpus tetragonolobus, Crotalaria pallida, Clitoria ternatea* by Exonerate. Each query gene retained a maximum of two best target predictions, with the alignment score of the second-best prediction required to be greater than 90% of that obtained from the best prediction. In Augustus gene model prediction, these homology gene regions were transformed into hints in gff format and utilized as hints data.

**Table S9. Statistics of predicted gene models by Augustus.**

| species | Gene number | Average CDS Length | Average Exon Number | Genes with hints | Genes with hints % | BUSCO results with database embryophyta_odb10 |
| --- | --- | --- | --- | --- | --- | --- |
| *Canavalia gladiata* | 51,158 | 994 | 4.72 | 27,758 | 54.26% | C:99.4%[S:96.5%,D:2.9%] |
| *Phaseolus coccineus* | 35,523 | 1,190 | 5.19 | 26,440 | 74.43% | C:99.6%[S:96.6%,D:3.0%] |
| *Psophocarpus tetragonolobus* | 40,081 | 1,091 | 4.95 | 25,991 | 64.85% | C:99.2%[S:96.6%,D:2.6%] |
| *Crotalaria pallida* | 48,759 | 1,050 | 4.84 | 28,056 | 57.54% | C:98.1%[S:93.0%,D:5.1%] |
| *Clitoria ternatea* | 40,267 | 1,034 | 4.77 | 25,767 | 63.99% | C:98.9%[S:94.7%,D:4.2%] |

Note: In BUSCO results, “C” represents a complete gene, “S” represents a single-copy gene, and “D” represents a duplicated gene. Both “S” and “D” correspond to complete genes.

**Table S10. Statistics of gene function assignment.**

| species | gene number | NR hit % | KEGG hit % | InterPro hits % | InterPro term % | GO terms % | All function % |
| --- | --- | --- | --- | --- | --- | --- | --- |
| *Canavalia gladiata* | 51,158 | 63.62% | 46.58% | 69.48% | 53.49% | 39.26% | 72.12% |
| *Phaseolus coccineus* | 35,523 | 83.30% | 61.81% | 85.66% | 70.14% | 52.84% | 89.01% |
| *Psophocarpus tetragonolobus* | 40,081 | 73.36% | 53.88% | 80.95% | 61.22% | 45.99% | 83.88% |
| *Crotalaria pallida* | 48,759 | 70.21% | 51.29% | 85.43% | 57.57% | 42.53% | 87.73% |
| *Clitoria ternatea* | 40,267 | 73.95% | 54.39% | 83.95% | 61.46% | 46.17% | 86.76% |

Note: All function refers to the comprehensive set of function annotations, inclusive of at least one of the NR, KEGG, InterPro, and GO annotations.

**Table S11. Statistics of annotated non-coding RNA genes.**

| Species | tRNA genes | 5S_rRNA genes | 18S_rRNA genes | 28S_rRNA genes | All rRNA genes |
| --- | --- | --- | --- | --- | --- |
| *Canavalia gladiata* | 970 | 23 | 792 | 720 | 1,535 |
| *Phaseolus coccineus* | 1,141 | 2,972 | 1,032 | 1,026 | 5,030 |
| *Psophocarpus tetragonolobus* | 1,283 | 22 | 1,517 | 1,481 | 3,020 |
| *Crotalaria pallida* | 1,382 | 2,419 | 1,921 | 1,928 | 6,268 |
| *Clitoria ternatea* | 2,307 | 80 | 1,537 | 1,541 | 3,158 |

Note: All rRNA genes consist of the collective number of 5S, 18S, and 28S rRNA genes.

**Table S12. Statistics of TE annotations.**

| Species | Intact TE | Intact  TE % | Non-intact TE | Non-intact TE % | Total TE | Total TE % |
| --- | --- | --- | --- | --- | --- | --- |
| *Canavalia gladiata* | 16,092,529 | 2.60% | 325,215,689 | 52.52% | 341,308,218 | 55.12% |
| *Phaseolus coccineus* | 62,833,080 | 10.60% | 313,298,046 | 52.86% | 376,131,126 | 63.46% |
| *Psophocarpus tetragonolobus* | 18,401,976 | 2.58% | 438,421,011 | 61.51% | 456,822,987 | 64.09% |
| *Crotalaria pallida* | 175,471,468 | 14.41% | 818,843,374 | 67.25% | 994,314,842 | 81.66% |
| *Clitoria ternatea* | 248,041,221 | 14.38% | 1,235,625,160 | 71.65% | 1,483,666,381 | 86.03% |

Note: TE stands for “transposable element.” Intact TEs were predicted by EDTA, while non-intact TEs were identified through annotation by RepeatMasker.

**Table S13. Statistics of TE content in various classes.**

| TE class | *Canavalia gladiata* | | *Phaseolus coccineus* | | *Psophocarpus tetragonolobus* | | *Crotalaria pallida* | | *Clitoria ternatea* | |
| --- | --- | --- | --- | --- | --- | --- | --- | --- | --- | --- |
|  | **Length (bp)** | **Percent** | **Length (bp)** | **Percent** | **Length (bp)** | **Percent** | **Length (bp)** | **Percent** | **Length (bp)** | **Percent** |
| LTR | 180,846,828 | 29.21% | 286,668,150 | 48.36% | 218,093,565 | 30.60% | 738,471,806 | 60.65% | 1,220,549,182 | 70.77% |
| DNA | 80,715,977 | 13.04% | 47,793,118 | 8.06% | 105,578,526 | 14.81% | 170,435,349 | 14.00% | 189,781,139 | 11.00% |
| TIR | 58,171,489 | 9.39% | 22,961,517 | 3.87% | 66,833,829 | 9.38% | 12,718,949 | 1.04% | 40,704,472 | 2.36% |
| LINE | 14,849,466 | 2.40% | 10,979,496 | 1.85% | 19,893,664 | 2.79% | 12,878,117 | 1.06% | 21,867,861 | 1.27% |
| MITE | 2,701,111 | 0.44% | 5,442,876 | 1.45% | 10,311,782 | 1.45% | 57,637,836 | 4.73% | 5,330,481 | 0.31% |
| RC | 467,493 | 0.08% | 896,271 | 0.15% | 559,742 | 0.08% | 702,095 | 0.06% | 879,084 | 0.05% |
| SINE | 1,865,781 | 0.30% | 836,223 | 0.14% | 8,608,358 | 1.21% | 1,109,373 | 0.09% | 3,055,672 | 0.18% |
| other | 1,687,400 | 0.27% | 551,679 | 0.09% | 26,943,521 | 3.78% | 346,617 | 0.03% | 6,828,971 | 0.40% |
| Total | 341,305,545 | 55.12% | 376,129,330 | 63.46% | 456,822,987 | 64.09% | 994,300,142 | 81.66% | 1,483,666,381 | 86.03% |

**Table S14. Statistics of TE content in various superfamily.**

| Species | LTR/Gypsy | | LTR/Copia | | LTR/unknown | |
| --- | --- | --- | --- | --- | --- | --- |
|  | **Length(bp)** | **Percent** | **Length(bp)** | **Percent** | **Length(bp)** | **Percent** |
| *Canavalia gladiata* | 133,458,668 | 21.55% | 20,125,133 | 3.25% | 22,316,875 | 3.60% |
| *Phaseolus coccineus* | 193,226,843 | 32.60% | 47,021,236 | 7.93% | 43,237,009 | 7.29% |
| *Psophocarpus tetragonolobus* | 141,257,264 | 19.82% | 43,980,834 | 6.17% | 24,535,043 | 3.44% |
| *Crotalaria pallida* | 325,036,285 | 26.69% | 247,820,444 | 20.35% | 159,989,760 | 13.14% |
| *Clitoria ternatea* | 805,467,836 | 46.70% | 229,355,338 | 13.30% | 178,980,864 | 10.38% |

**Table S15. Public genome data information.**

| Species Name | Ploidy | SeqTech | Assembled size | Contig N50 | Assembly level/Scaffold N50 | Data Address | Data version |
| --- | --- | --- | --- | --- | --- | --- | --- |
| Aeschynomene evenia | 2n=2x=20 | Pacbio + genetic map | 376 Mb | - | Chromosome level, 0.985 Mb | Phytozome | Aeschynomene_evenia_v1.0 |
| Arachis hypogaea | 2n=4x=40 | Pacbio + Illumina + bionano | 2.55 Gb | 47.91 Kb | Chromosome level, 31.82 Mb | NCBI | ASM417044v1 |
| Glycine max | 2n=40 | Pacbio + bionano + hic | 1,01 Gb | 22.6 Mb | Chromosome level, 52.0 Mb | NGDC | Gmax_ZH13_2.0 |
| Lablab purpureus | 2n=22 | nanopore + illumina + hic | 426.2 Mb | 11.0 Mb | Chromosome level, 38.1 Mb | NCBI | ILRI_Lpur |
| Lotus japonicus | 2n=12 | Pacbio + hic + genetic map | 500 Mb | 808 Kb | Chromosome level, 95.6 Mb | NCBI | LjGifu_v1.2 |
| Medicago truncatula | 2n=16 | Pacbio + bionano | 430 Mb | - | Chromosome level, 56.2 Mb | NCBI | MtrunA17r5.0 |
| Mucuna pruriens var. utilis | 2n=22 | BGI-seq + hic | 500.49 Mb | 92.5 Kb | Chromosome level, 48.4 Mb | CNSA | CNA0046511 |
| Phaseolus vulgaris | 2n=2x=22 | Pacbio | 537 Mb | 1.9 Mb | Chromosome level, 50 Mb | phytozome | Pvulgaris_442_v2.1 |
| Pisum sativum | 2n=2x=14 | Pacbio + bionano + hic | 3.80 Gb | 8.98 Mb | Chromosome level, 77.1Mb | NCBI | Psat_ZW6_1.0_ |
| Pueraria_lobata_var_montana | 2*n* = 2*x* = 22 | Pacbio + hic | 979 Mb | 1.61Mb | Chromosome level, 80Mb | NCBI | ASM2517757v1 |
| Styphnolobium japonicum [Sophora japonica] | 2n=28 | nanopore + illumina + hic | 511.49 Mb | 17.3 Mb | Chromosome level, 31.32MB | figshare | Sjaponica |
| Vigna angularis | 2n=22 | Illumina | 467 Mb | 38.4 Kb | Chromosome level, 1.3 Mb | NCBI | Vigan1.1 |
| Vitis vinifera (grape) | 2n=38 | Sanger+genetic map | 490Mb | 65.9 Kb | Chromosome level, 22.36Mb | NCBI | 12X |

**Table S16. Public gene data information.**

| Species | Gene number reported | Gene number used | Average exon number | Total length of exon (bp) | Average CDS length (bp) | BUSCO completeness estimation(embryophyta_odb10) |
| --- | --- | --- | --- | --- | --- | --- |
| *Aeschynomene evenia* | 32,667 | 32,667 | 5.04 | 39,616,937 | 1,212 | C:96.4%[S:92.6%D:3.8%] |
| *Arachis hypogaea* | 83,087 | 83,061 | 4.95 | 94,806,187 | 1,141 | C:96.7%[S:19.3%D:77.4%] |
| *Glycine max* | 55,443 | 55,443 | 4.88 | 62,003,377 | 1,118 | C:96.3%[S:48.2%D:48.1%] |
| *Lablab purpureus* | 24,972 | 24,972 | 5.44 | 32,181,304 | 1,288 | C:97.4%[S:95.2%D:2.2%] |
| *Lotus japonicus* | 30,243 | 32,752 | 4.88 | 43,002,523 | 1,312 | C:99.3%[S:95.0%D:4.3%] |
| *Medicago truncatula* | 44,623 | 44,450 | 3.92 | 45,297,900 | 1,019 | C:97.3%[S:93.0%D:4.3%] |
| *Mucuna pruriens* | 28,010 | 28,010 | 5.19 | 34,729,143 | 1,239 | C:98.4%[S:95.5%D:2.9%] |
| *Phaseolus vulgaris* | 27,433 | 27,433 | 5.24 | 34,532,154 | 1,258 | C:98.0%[S:95.2%D:2.8%] |
| *Pisum sativum* | 47,526 | 47,515 | 4.59 | 52,902,081 | 1,113 | C:98.6%[S:94.9%D:3.7%] |
| *Pueraria montana* | 38,812 | 38,812 | 4.59 | 40,085,380 | 1,032 | C:93.9%[S:86.9%D:7.0%] |
| *Styphnolobium japonicum* | 31,000 | 31,000 | 5.65 | 40,627,512 | 1,310 | C:94.7%[S:84.8%D:9.9%] |
| *Vigna angularis* | 34,183 | 34,172 | 4.48 | 38,074,920 | 1,114 | C:84.3%[S:81.5%D:2.8%] |
| *Vitis vinifera* (grape) | 30,434 | 25,676 | 5.25 | 34,648,907 | 1,349 | C:97.7%[S:96.1%,D:1.6%] |

**Table S17. Summary of duplicated genes.**

| Species |  | Singleton | Dispersed | Proximal | Tandem | WGD / Segmental | Total |
| --- | --- | --- | --- | --- | --- | --- | --- |
| *Canavalia gladiata* | number | 12,083 | 17,207 | 2,743 | 4,139 | 14,986 | 51,158 |
|  | percent | 23.6% | 33.6% | 5.4% | 8.1% | 29.3% | 100.0% |
| *Phaseolus coccineus* | number | 6,416 | 13,375 | 1,928 | 3,443 | 10,361 | 35,523 |
|  | percent | 18.1% | 37.7% | 5.4% | 9.7% | 29.2% | 100.0% |
| *Psophocarpus tetragonolobus* | number | 8,357 | 15,160 | 2,091 | 3,445 | 11,028 | 40,081 |
|  | percent | 20.9% | 37.8% | 5.2% | 8.6% | 27.5% | 100.0% |
| *Crotalaria pallida* | number | 6,358 | 21,218 | 2,804 | 3,812 | 14,567 | 48,759 |
|  | percent | 13.0% | 43.5% | 5.8% | 7.8% | 29.9% | 100.0% |
| *Clitoria ternatea* | number | 6,424 | 18,363 | 1,814 | 2,846 | 10,820 | 40,267 |
|  | percent | 16.0% | 45.6% | 4.5% | 7.1% | 26.9% | 100.0% |

Note: These statistics were derived from the output of the duplicate_gene_classifier, which is part of the MCScanX package. “Singleton” represents non-duplicated single copy genes; “Dispersed” represents gene duplications distributed randomly across the genome; “Proximal” represents gene duplications located near each other, “Tandem” represents exact tandem duplicated genes; “WGD/Segmental” represents gene duplications situated within long colinear fragments.

**Table S18. Features between Phaseolus coccineus and Phaseolus vulgaris.**

|  | Phaseolus coccineus | Phaseolus vulgaris |
| --- | --- | --- |
| assembly size | 592,734,161 | 615,158,789 |
| gene number | 35,523 | 42,801 |
| LTR-TE | 286,668,150 | 285,271,855 |
| DNA-TE | 47,793,118 | 69,306,760 |
| orther-TE | 41,669,747 | 45,158,874 |
| all TE | 376,131,126 | 399,737,489 |
| all TE% | 63.5% | 65.0% |
| TR | 54,428,626 | 67,180,099 |
| TR % | 9.2% | 10.9% |
| Non repeat | 168,793,995 | 207,053,918 |

Note: The repeat annotation and gene prediction of *Phaseolus vulgaris* using the pipeline we used in *Phaseolus coccineus*, the repeat is the sum of TR and TE, and the non-repeats refer to the regions of the genome that are not made up of repeated sequences.

**Table S19. Reference gene protein sequence information of nitrogen-fixing root nodulation.**

| Gene | Accession | protein length | Database |
| --- | --- | --- | --- |
| *CHS* | AAA32771.1  NP_001358310.1  NP_001358311.1  NP_001358312.1  NP_001304378.2  NP_001337038.1  NP_001347353.1  AAA02824.1  NP_001276296.1  NP_001358304.1  NP_001347352.1  NP_001347288.1  AAA33951.1  NP_001340309.1  NP_001304585.2  NP_001358306.1  Q9ZRV7  NP_001267879.1  P49440 | 395  388  388  391  390  388  388  389  388  388  388  388  388  389  389  388  389  393  389 | NCBI |
| *IFS* | AFJ80647.1  SBU44857.1  AFJ80646.1  AKI30023.1  ACA81463.1  ACA81506.1  ALL55646.1  AAF34522.1  AAF34523.1  AAF34521.1  ACF04277.1  Q7FZC0  A0A0S3R1L0  Q9M6C5 | 521  521  521  521  521  521  521  500  500  500  522  521  521  521 | NCBI |
| *NFR1* | CAE02590.1  CAE02589.1  CAE02592.1  CAE02591.1  CAZ66916.1 | 623  621  623  621  610 | NCBI |
| *NFR5* | AER51029.1  AER51028.1  AER51027.1  CAE02598.1  CAZ66917.1  WCK83815.1  ADJ19106.1 | 595  595  595  595  595  595  598 | NCBI |
| *SYMRK* | AAM67418.1  AAY22390.1  AAM76685.1 | 923  923  925 | NCBI |
| *HMGR1* | ABY20972.1 | 550 | NCBI |
| *DMI3* | AET75798.1  AET75786.1  RDX70282.1  NP_001363154.1 | 523  523  526  523 | NCBI |
| *IPD3* | A7TUE1.1 | 513 | NCBI |
| *DELLA* | NP_001411950.1  XP_050893651.1  XP_039684656.1 | 594  532  533 | NCBI |
| *NSP1* | LJ_A1DQP9.1 | 542 | NCBI |
| *NSP2* | LJ_Q2PEG7.1 | 499 | NCBI |
| *NIN* | UWK31661.1  CAD37948.1  CAD37947.1  CAD37949.1  CAD37946.1  CAB61338.1  CAB61243.1 | 934  921  924  924  922  878  878 | NCBI |
|  | Glyma.06G000400  Glyma.04G000600  Glyma.14G001600 Glyma.02G311000 | 581  785  583  710 | Phytozome |
| *RPG* | WBW64332.1  ABI51615.1 | 1176  1255 | NCBI |
| *NLP2* | CAE30325.1  XP_003518588.1 | 972  710 | NCBI |

**Table S20. Genes involved in nitrogen-fixing root nodulation.**

| Gene | *Canavalia gladiata* | *Phaseolus coccineus* | *Psophocarpus tetragonolobus* | *Crotalaria pallida* | *Clitoria ternatea* |
| --- | --- | --- | --- | --- | --- |
| *CHS* | Cgla.ptg000003l.g1255.t1  Cgla.ptg000003l.g1256.t1  Cgla.ptg000010l.g277.t1  Cgla.ptg000019l.g143.t1  Cgla.ptg000015l.g5267.t1  Cgla.ptg000015l.g5268.t1  Cgla.ptg000015l.g5269.t1  Cgla.ptg000015l.g5270.t1 | Pcoc.ptg000004l.g174.t1  Pcoc.ptg000004l.g175.t1  Pcoc.ptg000004l.g176.t1  Pcoc.ptg000004l.g178.t1  Pcoc.ptg000004l.g179.t1  Pcoc.ptg000004l.g180.t1  Pcoc.ptg000004l.g181.t1  Pcoc.ptg000004l.g182.t1  Pcoc.ptg000004l.g186.t1  Pcoc.ptg000001l.g938.t1  Pcoc.ptg000006l.g316.t1 | Ptet.ptg000002l.g1409.t1  Ptet.ptg000033l.g346.t1  Ptet.ptg000054l.g18.t1  Ptet.ptg000054l.g308.t1  Ptet.ptg000054l.g309.t1  Ptet.ptg000054l.g311.t1  Ptet.ptg000054l.g312.t1  Ptet.ptg000054l.g313.t1  Ptet.ptg000054l.g315.t1  Ptet.ptg000054l.g319.t1  Ptet.ptg000054l.g320.t1 | Cpal.ptg000001l.g1714.t1  Cpal.ptg000005l.g163.t1  Cpal.ptg000005l.g164.t1  Cpal.ptg000005l.g165.t1  Cpal.ptg000005l.g166.t1 | Cter.ptg000001l.g3286.t1  Cter.ptg000004l.g2333.t1  Cter.ptg000004l.g2334.t1  Cter.ptg000004l.g2335.t1  Cter.ptg000005l.g1370.t1  Cter.ptg000019l.g1154.t1 |
| *IFS* | Cgla.ptg000003l.g5741.t1  Cgla.ptg000003l.g5742.t1 | Pcoc.ptg000011l.g2752.t1  Pcoc.ptg000014l.g592.t1  Pcoc.ptg000014l.g595.t1  Pcoc.ptg000014l.g593.t1 | Ptet.ptg000068l.g198.t1  Ptet.ptg000068l.g202.t1  Ptet.ptg000068l.g204.t1 | Cpal.ptg000005l.g1942.t1  Cpal.ptg000005l.g1941.t1 | Cter.ptg000019l.g2060.t1 |
| *NFR1* | Cgla.ptg000003l.g1806.t1  Cgla.ptg000012l.g2798.t1  Cgla.ptg000012l.g2799.t1  Cgla.ptg000014l.g2399.t1 | Pcoc.ptg000010l.g897.t1  Pcoc.ptg000010l.g898.t1  Pcoc.ptg000015l.g2567.t1  Pcoc.ptg000015l.g857.t1 | Ptet.ptg000003l.g433.t1  Ptet.ptg000003l.g434.t1  Ptet.ptg000012l.g709.t1  Ptet.ptg000071l.g556.t1 | Cpal.ptg000004l.g4457.t1  Cpal.ptg000004l.g5084.t1  Cpal.ptg000007l.g3345.t1 | Cter.ptg000004l.g3453.t1  Cter.ptg000010l.g666.t1  Cter.ptg000010l.g667.t1  Cter.ptg000010l.g669.t1  Cter.ptg000012l.g1132.t1 |
| *NFR5* | Cgla.ptg000003l.g1940.t1  Cgla.ptg000010l.g1033.t1  Cgla.ptg000010l.g1574.t1 | Pcoc.ptg000004l.g1067.t1 | Ptet.ptg000041l.g505.t1 | Cpal.ptg000005l.g4632.t1  Cpal.ptg000005l.g618.t1 | Cter.ptg000003l.g2914.t1 |
| *SYMRK* | Cgla.ptg000010l.g1595.t1 | Pcoc.ptg000004l.g491.t1 | Ptet.ptg000017l.g371.t1 | Cpal.ptg000005l.g1353.t1 | Cter.ptg000003l.g2170.t1 |
| *HMGR1* | Cgla.ptg000003l.g2419.t1  Cgla.ptg000010l.g1343.t1  Cgla.ptg000012l.g2828.t1  Cgla.ptg000014l.g2531.t1 | Pcoc.ptg000004l.g763.t1  Pcoc.ptg000010l.g918.t1  Pcoc.ptg000011l.g315.t1  Pcoc.ptg000015l.g2408.t1 | Ptet.ptg000003l.g411.t1  Ptet.ptg000022l.g135.t1  Ptet.ptg000041l.g849.t1  Ptet.ptg000053l.g17.t1  Ptet.ptg000080l.g9.t1 | Cpal.ptg000004l.g4488.t1  Cpal.ptg000005l.g1076.t1  Cpal.ptg000005l.g4838.t1 | Cter.ptg000003l.g2546.t1  Cter.ptg000004l.g3339.t1  Cter.ptg000010l.g629.t1  Cter.ptg000014l.g853.t1 |
| *DMI3* | Cgla.ptg000002l.g166.t1 | Pcoc.ptg000003l.g2288.t1 | Ptet.ptg000056l.g16.t1 | Cpal.ptg000017l.g617.t1 | Cter.ptg000004l.g1730.t1 |
| *IPD3* | Cgla.ptg000010l.g1438.t1 | Pcoc.ptg000004l.g668.t1 | Ptet.ptg000041l.g968.t1 | Cpal.ptg000005l.g1184.t1 | Cter.ptg000003l.g2412.t1 |
| *DELLA* | Cgla.ptg000003l.g1466.t1  Cgla.ptg000014l.g1267.t1  Cgla.ptg000015l.g857.t1  Cpal.ptg000004l.g4150.t1 | Pcoc.ptg000001l.g2942.t1  Pcoc.ptg000008l.g1655.t1  Pcoc.ptg000010l.g594.t1  Pcoc.ptg000011l.g76.t1 | Ptet.ptg000002l.g1241.t1  Ptet.ptg000022l.g116.t1  Ptet.ptg000046l.g434.t1 | Cpal.ptg000014l.g70.t1  Cpal.ptg000019l.g277.t1 | Cter.ptg000016l.g1468.t1  Cter.ptg000019l.g1388.t1  Cter.ptg000036l.g85.t1 |
| *NSP1* | Cgla.ptg000001l.g3174.t1 | Pcoc.ptg000032l.g418.t1 | Ptet.ptg000047l.g121.t1 | Cpal.ptg000011l.g904.t1 | Cter.ptg000002l.g2881.t1 |
| *NSP2* | Cgla.ptg000005l.g1446.t1  Cgla.ptg000012l.g1683.t1 | Pcoc.ptg000002l.g1246.t1  Pcoc.ptg000010l.g1433.t1 | Ptet.ptg000006l.g533.t1  Ptet.ptg000023l.g1140.t1 | Cpal.ptg000003l.g437.t1  Cpal.ptg000004l.g3529.t1 | Cter.ptg000005l.g853.t1  Cter.ptg000010l.g1824.t1 |
| *NIN* | Cgla.ptg000005l.g10.t1  Cgla.ptg000012l.g3346.t1 | Pcoc.ptg000002l.g20.t1  Pcoc.ptg000010l.g17.t1 | Ptet.ptg000003l.g16.t1  Ptet.ptg000030l.g347.t1 | Cpal.ptg000013l.g30.t1 | Cter.ptg000010l.g21.t1 |
| *RPG* | Cgla.ptg000007l.g3150.t1 | Pcoc.ptg000008l.g1626.t1 | Ptet.ptg000057l.g381.t1 | Cpal.ptg000006l.g3049.t1 | Cter.ptg000001l.g1351.t1 |
| *NLP2* | Cgla.ptg000006l.g189.t1  Cgla.ptg000006l.g196.t1  Cgla.ptg000009l.g2686.t1 | Pcoc.ptg000003l.g617.t1  Pcoc.ptg000013l.g346.t1 | Ptet.ptg000050l.g270.t1  Ptet.ptg000097l.g54.t1 | Cpal.ptg000001l.g675.t1  Cpal.ptg000015l.g980.t1 | Cter.ptg000009l.g209.t1  Cter.ptg000014l.g2731.t1 |

**Table S21. The Pfam domain annotation of *NIN* genes.**

| *NIN* gene | Protein length | Pfam ID | Domain description | Start | End | E-value |
| --- | --- | --- | --- | --- | --- | --- |
| Cgla.ptg000005l.g10.t1 | 802 | PF02042 | RWP-RK domain | 513 | 561 | 6.80E-25 |
| Cgla.ptg000005l.g10.t1 | 802 | PF00564 | PB1 domain | 712 | 793 | 1.40E-15 |
| Cgla.ptg000012l.g3346.t1 | 753 | PF02042 | RWP-RK domain | 497 | 544 | 3.90E-24 |
| Cgla.ptg000012l.g3346.t1 | 753 | PF00564 | PB1 domain | 656 | 737 | 1.20E-14 |
| Cter.ptg000010l.g21.t1 | 686 | PF02042 | RWP-RK domain | 453 | 500 | 3.40E-24 |
| Cter.ptg000010l.g21.t1 | 686 | PF00564 | PB1 domain | 594 | 676 | 3.50E-12 |
| Cpal.ptg000013l.g30.t1 | 925 | PF02042 | RWP-RK domain | 603 | 650 | 5.00E-24 |
| Cpal.ptg000013l.g30.t1 | 925 | PF00564 | PB1 domain | 808 | 889 | 2.90E-15 |
| Pcoc.ptg000002l.g20.t1 | 822 | PF02042 | RWP-RK domain | 516 | 563 | 4.30E-24 |
| Pcoc.ptg000002l.g20.t1 | 822 | PF00564 | PB1 domain | 724 | 805 | 1.20E-16 |
| Pcoc.ptg000010l.g17.t1 | 741 | PF02042 | RWP-RK domain | 509 | 556 | 3.80E-24 |
| Pcoc.ptg000010l.g17.t1 | 741 | PF00564 | PB1 domain | 655 | 736 | 4.10E-13 |
| Ptet.ptg000003l.g16.t1 | 699 | PF02042 | RWP-RK domain | 487 | 535 | 1.80E-24 |
| Ptet.ptg000003l.g16.t1 | 699 | PF00564 | PB1 domain | 605 | 689 | 4.30E-15 |
| Ptet.ptg000030l.g347.t1 | 913 | PF02042 | RWP-RK domain | 643 | 690 | 4.90E-24 |
| Ptet.ptg000030l.g347.t1 | 913 | PF00564 | PB1 domain | 815 | 897 | 9.50E-16 |

Note: The information about Pfam domain was obtained from InterProScan results. Each *NIN* has an RWP-RK domain and a PB1 domain.

**Table S22. The Pfam domain annotation of *CHS* genes.**

| CHS gene | Protein length | Pfam ID | Domain description | Start | End | E-value |
| --- | --- | --- | --- | --- | --- | --- |
| Cgla.ptg000003l.g1256.t1 | 388 | PF00195 | Chalcone and stilbene synthases, N-terminal domain | 5 | 228 | 8.20E-126 |
| Cgla.ptg000003l.g1256.t1 | 388 | PF02797 | Chalcone and stilbene synthases, C-terminal domain | 237 | 387 | 1.20E-71 |
| Cgla.ptg000003l.g1255.t1 | 384 | PF02797 | Chalcone and stilbene synthases, C-terminal domain | 233 | 383 | 1.80E-69 |
| Cgla.ptg000003l.g1255.t1 | 384 | PF00195 | Chalcone and stilbene synthases, N-terminal domain | 47 | 224 | 1.60E-90 |
| Cgla.ptg000010l.g277.t1 | 389 | PF00195 | Chalcone and stilbene synthases, N-terminal domain | 6 | 228 | 1.90E-124 |
| Cgla.ptg000010l.g277.t1 | 389 | PF02797 | Chalcone and stilbene synthases, C-terminal domain | 238 | 387 | 1.10E-68 |
| Cgla.ptg000015l.g5267.t1 | 389 | PF00195 | Chalcone and stilbene synthases, N-terminal domain | 5 | 228 | 1.10E-122 |
| Cgla.ptg000015l.g5267.t1 | 389 | PF02797 | Chalcone and stilbene synthases, C-terminal domain | 238 | 387 | 5.70E-67 |
| Cgla.ptg000015l.g5268.t1 | 389 | PF00195 | Chalcone and stilbene synthases, N-terminal domain | 5 | 228 | 1.50E-124 |
| Cgla.ptg000015l.g5268.t1 | 389 | PF02797 | Chalcone and stilbene synthases, C-terminal domain | 238 | 387 | 6.80E-70 |
| Cgla.ptg000015l.g5270.t1 | 389 | PF00195 | Chalcone and stilbene synthases, N-terminal domain | 5 | 228 | 3.60E-125 |
| Cgla.ptg000015l.g5270.t1 | 389 | PF02797 | Chalcone and stilbene synthases, C-terminal domain | 238 | 387 | 1.10E-69 |
| Cgla.ptg000015l.g5269.t1 | 389 | PF02797 | Chalcone and stilbene synthases, C-terminal domain | 238 | 387 | 6.50E-70 |
| Cgla.ptg000015l.g5269.t1 | 389 | PF00195 | Chalcone and stilbene synthases, N-terminal domain | 5 | 228 | 6.30E-125 |
| Cgla.ptg000019l.g143.t1 | 391 | PF00195 | Chalcone and stilbene synthases, N-terminal domain | 5 | 228 | 2.30E-124 |
| Cgla.ptg000019l.g143.t1 | 391 | PF02797 | Chalcone and stilbene synthases, C-terminal domain | 238 | 388 | 3.40E-71 |
| Pcoc.ptg000001l.g938.t1 | 468 | PF02797 | Chalcone and stilbene synthases, C-terminal domain | 315 | 465 | 5.60E-72 |
| Pcoc.ptg000001l.g938.t1 | 468 | PF00195 | Chalcone and stilbene synthases, N-terminal domain | 82 | 305 | 1.60E-124 |
| Pcoc.ptg000004l.g186.t1 | 414 | PF00195 | Chalcone and stilbene synthases, N-terminal domain | 30 | 253 | 3.50E-125 |
| Pcoc.ptg000004l.g186.t1 | 414 | PF02797 | Chalcone and stilbene synthases, C-terminal domain | 263 | 412 | 6.10E-69 |
| Pcoc.ptg000004l.g180.t1 | 442 | PF00195 | Chalcone and stilbene synthases, N-terminal domain | 58 | 281 | 4.20E-125 |
| Pcoc.ptg000004l.g180.t1 | 442 | PF02797 | Chalcone and stilbene synthases, C-terminal domain | 291 | 440 | 7.00E-69 |
| Pcoc.ptg000004l.g179.t1 | 389 | PF02797 | Chalcone and stilbene synthases, C-terminal domain | 238 | 387 | 5.40E-69 |
| Pcoc.ptg000004l.g179.t1 | 389 | PF00195 | Chalcone and stilbene synthases, N-terminal domain | 5 | 228 | 1.40E-124 |
| Pcoc.ptg000004l.g175.t1 | 389 | PF02797 | Chalcone and stilbene synthases, C-terminal domain | 238 | 387 | 5.40E-69 |
| Pcoc.ptg000004l.g175.t1 | 389 | PF00195 | Chalcone and stilbene synthases, N-terminal domain | 5 | 228 | 1.40E-124 |
| Pcoc.ptg000004l.g181.t1 | 458 | PF00195 | Chalcone and stilbene synthases, N-terminal domain | 74 | 297 | 2.20E-124 |
| Pcoc.ptg000004l.g181.t1 | 458 | PF02797 | Chalcone and stilbene synthases, C-terminal domain | 307 | 456 | 7.60E-69 |
| Pcoc.ptg000004l.g178.t1 | 389 | PF02797 | Chalcone and stilbene synthases, C-terminal domain | 238 | 387 | 5.40E-69 |
| Pcoc.ptg000004l.g178.t1 | 389 | PF00195 | Chalcone and stilbene synthases, N-terminal domain | 5 | 228 | 1.40E-124 |
| Pcoc.ptg000004l.g174.t1 | 389 | PF00195 | Chalcone and stilbene synthases, N-terminal domain | 5 | 228 | 8.10E-125 |
| Pcoc.ptg000004l.g174.t1 | 389 | PF02797 | Chalcone and stilbene synthases, C-terminal domain | 238 | 387 | 5.40E-69 |
| Pcoc.ptg000004l.g182.t1 | 389 | PF02797 | Chalcone and stilbene synthases, C-terminal domain | 238 | 387 | 1.50E-68 |
| Pcoc.ptg000004l.g182.t1 | 389 | PF00195 | Chalcone and stilbene synthases, N-terminal domain | 5 | 228 | 3.00E-125 |
| Pcoc.ptg000004l.g176.t1 | 389 | PF02797 | Chalcone and stilbene synthases, C-terminal domain | 238 | 387 | 5.40E-69 |
| Pcoc.ptg000004l.g176.t1 | 389 | PF00195 | Chalcone and stilbene synthases, N-terminal domain | 5 | 228 | 1.40E-124 |
| Pcoc.ptg000006l.g316.t1 | 388 | PF00195 | Chalcone and stilbene synthases, N-terminal domain | 5 | 228 | 3.10E-122 |
| Pcoc.ptg000006l.g316.t1 | 388 | PF02797 | Chalcone and stilbene synthases, C-terminal domain | 237 | 387 | 3.30E-71 |
| Ptet.ptg000002l.g1409.t1 | 421 | PF00195 | Chalcone and stilbene synthases,N-terminal domain | 54 | 108 | 1.30E-18 |
| Ptet.ptg000002l.g1409.t1 | 421 | PF00195 | Chalcone and stilbene synthases,N-terminal domain | 133 | 261 | 3.10E-71 |
| Ptet.ptg000002l.g1409.t1 | 421 | PF02797 | Chalcone and stilbene synthases,C-terminal domain | 270 | 420 | 2.80E-70 |
| Ptet.ptg000033l.g346.t1 | 430 | PF00195 | Chalcone and stilbene synthases,N-terminal domain | 45 | 268 | 6.40E-123 |
| Ptet.ptg000033l.g346.t1 | 430 | PF02797 | Chalcone and stilbene synthases,C-terminal domain | 278 | 427 | 8.80E-72 |
| Ptet.ptg000054l.g309.t1 | 414 | PF00195 | Chalcone and stilbene synthases,N-terminal domain | 31 | 253 | 5.10E-123 |
| Ptet.ptg000054l.g309.t1 | 414 | PF02797 | Chalcone and stilbene synthases,C-terminal domain | 263 | 413 | 4.20E-68 |
| Ptet.ptg000054l.g315.t1 | 414 | PF00195 | Chalcone and stilbene synthases,N-terminal domain | 31 | 253 | 5.10E-123 |
| Ptet.ptg000054l.g315.t1 | 414 | PF02797 | Chalcone and stilbene synthases,C-terminal domain | 263 | 413 | 3.90E-68 |
| Ptet.ptg000054l.g313.t1 | 389 | PF02797 | Chalcone and stilbene synthases,C-terminal domain | 238 | 388 | 5.70E-68 |
| Ptet.ptg000054l.g313.t1 | 389 | PF00195 | Chalcone and stilbene synthases,N-terminal domain | 6 | 228 | 4.30E-123 |
| Ptet.ptg000054l.g312.t1 | 389 | PF00195 | Chalcone and stilbene synthases,N-terminal domain | 6 | 228 | 4.30E-123 |
| Ptet.ptg000054l.g312.t1 | 389 | PF02797 | Chalcone and stilbene synthases,C-terminal domain | 238 | 388 | 1.10E-67 |
| Ptet.ptg000054l.g308.t1 | 426 | PF02797 | Chalcone and stilbene synthases,C-terminal domain | 275 | 425 | 4.10E-68 |
| Ptet.ptg000054l.g308.t1 | 426 | PF00195 | Chalcone and stilbene synthases,N-terminal domain | 43 | 265 | 6.00E-123 |
| Ptet.ptg000054l.g320.t1 | 439 | PF00195 | Chalcone and stilbene synthases,N-terminal domain | 56 | 278 | 6.00E-123 |
| Ptet.ptg000054l.g320.t1 | 439 | PF02797 | Chalcone and stilbene synthases,C-terminal domain | 288 | 438 | 4.40E-68 |
| Ptet.ptg000054l.g311.t1 | 414 | PF02797 | Chalcone and stilbene synthases,C-terminal domain | 263 | 413 | 6.60E-68 |
| Ptet.ptg000054l.g311.t1 | 414 | PF00195 | Chalcone and stilbene synthases,N-terminal domain | 31 | 253 | 5.10E-123 |
| Ptet.ptg000054l.g18.t1 | 389 | PF02797 | Chalcone and stilbene synthases,C-terminal domain | 238 | 388 | 1.80E-67 |
| Ptet.ptg000054l.g18.t1 | 389 | PF00195 | Chalcone and stilbene synthases,N-terminal domain | 6 | 228 | 4.30E-123 |
| Ptet.ptg000054l.g319.t1 | 389 | PF00195 | Chalcone and stilbene synthases,N-terminal domain | 6 | 228 | 4.30E-123 |
| Ptet.ptg000054l.g319.t1 | 389 | PF02797 | Chalcone and stilbene synthases,C-terminal domain | 238 | 388 | 3.40E-68 |
| Cpal.ptg000001l.g1714.t1 | 411 | PF00195 | Chalcone and stilbene synthases, N-terminal domain | 27 | 250 | 1.00E-122 |
| Cpal.ptg000001l.g1714.t1 | 411 | PF02797 | Chalcone and stilbene synthases, C-terminal domain | 260 | 409 | 5.60E-72 |
| Cpal.ptg000005l.g165.t1 | 389 | PF00195 | Chalcone and stilbene synthases, N-terminal domain | 5 | 228 | 8.80E-122 |
| Cpal.ptg000005l.g165.t1 | 389 | PF02797 | Chalcone and stilbene synthases, C-terminal domain | 238 | 387 | 1.80E-68 |
| Cpal.ptg000005l.g166.t1 | 389 | PF02797 | Chalcone and stilbene synthases, C-terminal domain | 238 | 387 | 6.90E-68 |
| Cpal.ptg000005l.g166.t1 | 389 | PF00195 | Chalcone and stilbene synthases, N-terminal domain | 5 | 228 | 8.80E-122 |
| Cpal.ptg000005l.g164.t1 | 389 | PF00195 | Chalcone and stilbene synthases, N-terminal domain | 5 | 228 | 8.80E-122 |
| Cpal.ptg000005l.g164.t1 | 389 | PF02797 | Chalcone and stilbene synthases, C-terminal domain | 238 | 387 | 2.50E-68 |
| Cpal.ptg000005l.g163.t1 | 389 | PF02797 | Chalcone and stilbene synthases, C-terminal domain | 238 | 387 | 6.80E-68 |
| Cpal.ptg000005l.g163.t1 | 389 | PF00195 | Chalcone and stilbene synthases, N-terminal domain | 5 | 228 | 8.80E-122 |
| Cter.ptg000001l.g3286.t1 | 389 | PF00195 | Chalcone and stilbene synthases, N-terminal domain | 6 | 228 | 8.30E-125 |
| Cter.ptg000001l.g3286.t1 | 389 | PF02797 | Chalcone and stilbene synthases, C-terminal domain | 238 | 387 | 1.60E-68 |
| Cter.ptg000004l.g2333.t1 | 428 | PF00195 | Chalcone and stilbene synthases, N-terminal domain | 44 | 267 | 9.30E-125 |
| Cter.ptg000004l.g2333.t1 | 428 | PF02797 | Chalcone and stilbene synthases, C-terminal domain | 277 | 426 | 4.80E-70 |
| Cter.ptg000004l.g2335.t1 | 389 | PF02797 | Chalcone and stilbene synthases, C-terminal domain | 238 | 387 | 3.90E-70 |
| Cter.ptg000004l.g2335.t1 | 389 | PF00195 | Chalcone and stilbene synthases, N-terminal domain | 5 | 228 | 7.20E-125 |
| Cter.ptg000004l.g2334.t1 | 389 | PF02797 | Chalcone and stilbene synthases, C-terminal domain | 238 | 387 | 3.90E-70 |
| Cter.ptg000004l.g2334.t1 | 389 | PF00195 | Chalcone and stilbene synthases, N-terminal domain | 5 | 228 | 7.20E-125 |
| Cter.ptg000005l.g1370.t1 | 391 | PF02797 | Chalcone and stilbene synthases, C-terminal domain | 238 | 388 | 3.00E-71 |
| Cter.ptg000005l.g1370.t1 | 391 | PF00195 | Chalcone and stilbene synthases, N-terminal domain | 5 | 228 | 1.10E-118 |
| Cter.ptg000019l.g1154.t1 | 389 | PF00195 | Chalcone and stilbene synthases, N-terminal domain | 5 | 228 | 1.60E-123 |
| Cter.ptg000019l.g1154.t1 | 389 | PF02797 | Chalcone and stilbene synthases, C-terminal domain | 238 | 387 | 2.00E-71 |

Note: The Pfam domain information was obtained from InterProScan results. Each *CHS* gene has a Chalcone and stilbene synthases, N-terminal domain and a Chalcone and stilbene synthases, C-terminal domain.
