# Supplementary material for: The genomes of 5 underutilized Papilionoideae crops provide insights into root nodulation and disease resistance
Source: Gigascience. 2024 Aug 27;13:giae063. doi: 10.1093/gigascience/giae063 (PMC11348429; doi:10.1093/gigascience/giae063)
Supplement: giae063_Response_to_Reviewer_Comments_Original_Submission [file giae063_response_to_reviewer_comments_original_submission.pdf]

Author's Response To Reviewer Comments

Reviewer reports:

Reviewer #1: The authors assembled five legume species' genomes, and conducted the comparisons. They searched the five genomes for genes involved in nitrogen-fixing root nodule symbiosis, phenylalanine pathway, and resistance. I got several suggestions for references.

1. Why was the study designed with the genome assembly of 5 legume crops? Why selected these five species? This study concluded on several gene identification, such as the copy loss of known NIN gene.
Reply: As an agriculture genomics institute, our prime goal is to provide the reference genomes for important species with agricultural value. The 5 legume crops we selected in this study are underutilized Papilionoideae crops. The high-quality reference genomes will not only benefit their breeding, but also benefit the whole researches in Papilionoideae. However, at current stage, we could not obtain more functional insights with only the genomic data. Future studies using multi-omics technologies and other technologies such as genome editing and RNA interference will generate meaningful biological results.

2. Abstract, full species names and genome ploid levels for the five species should be provided. "we identified 6,710 and 669 unique genes between scarlet runner bean and common bean for each species", I cannot understand this sentence. How were these two gene figures counted? Are these five species all diploid?

Reply: We have added full species names and genome ploid levels in the revised Abstract section: “Here, we present chromosome-level reference genomes for five underutilized diploid Papilionoideae crops: sword bean (Canavalia gladiata), scarlet runner bean (Phaseolus coccineus), winged bean (Psophocarpus tetragonolobus), smooth rattlebox (Crotalaria pallida) and butterfly pea (Clitoria ternatea)”. All these five species are diploid.

To make it clear, the sentence “"we identified 6,710 and 669 unique genes between scarlet runner bean and common bean for each species" was revised into “we identified 5,328 and 10,434 species-specific genes between scarlet runner bean and common bean for each species”. In addition, we have shown the method to determine the species-specific genes in the revised Figure 4C legend: “Overlap of the reference gene sets between P. coccineus and P. vulgaris. The protein sequences between the two species are aligned using Diamond with the parameters "--sensitive --evalue 1e-5". Genes in each species that remain unaligned are considered as species-specific genes.”.

3. Introduction, the latest legume genome should be cited.
Reply: In the revised Introduction part, we have added two latest published legume genome papers:
Ho, W.K., Tanzi, A.S., Sang, F. et al. A genomic toolkit for winged bean Psophocarpus tetragonolobus. Nat Commun 15, 1901 (2024). https://doi.org/10.1038/s41467-024-45048-x.
Sébastien Carrère, Baptiste Mayjonade, David Lalanne, Sylvain Gaillard, Jérôme Verdier, Nicolas W.G. Chen, First whole genome assembly and annotation of a European common bean cultivar using PacBio HiFi and Iso-Seq data, Data in Brief, Volume 48, 2023, 109182, ISSN 2352-3409, https://doi.org/10.1016/j.dib.2023.109182.

4. "67 Gb, 70 Gb, 64 Gb, 69 Gb, and 167 Gb HiFi data", the data coverage should be provided to evalute the sequencing data size for genome assembly.
Reply: The sentence was revised into “67 Gb (103X), 70 Gb (119X), 64 Gb (93X), 69 Gb (52X), and 167 Gb (95X) HiFi data”.

5. English should be polished for the whole manuscript body.
Reply: We have tried our best to polish the English in the whole manuscript.

6. "51,158, 35,523, 40,081, 48,759 and 40,267 protein-coding gene models" are from five genomes with 0.65 Gb, 0.59 Gb, 0.69 Gb, 1.33 Gb, and 1.76 Gb. Protein-coding gene number is expected to match the genome size. Why did 1.76 Gb genome contain only 40,267 genes? Is it reasonable and does it make sense? The authors should explain the rational of gene modules.
Reply: The largest genome in this study is butterfly pea (1.76 Gb), which also has the highest amount of TEs. The genome size is mostly determined by the activity of TEs. The gene number do not need to be in proportional to the genome size.

7. What are the final genome size in addition to the estimated ones? The authors did not mention them in the manuscript.
Reply: We have added the final assembled contig sizes in the revised Result part:
"Then, the HiFi data were assembled into large contigs with total size 0.62 Mb, 0.59 Mb, 0.71 Gb, 1.22 Gb, and 1.72 Gb, which were further linked into 11, 11, 9, 8, 8 chromosome-scale scaffolds by Hi-C data, respectively".

8. "At last, the Lb gene was expressed to produce leghemoglobin." Is it based on the data in this study?
Reply: This sentence is based on previous studies. To make it clear, it was revised into "Then, the Lb gene was activated to produce leghemoglobin [39]". We added a reference paper for this sentence:
Wang et. al. CRISPR/Cas9 knockout of leghemoglobin genes in Lotus japonicus uncovers their synergistic roles in symbiotic nitrogen fixation. New Phytologist. 224:818–832 (2019).

9. Why do the authors claim the loss of NIN gene copies in smooth rattlebox and butterfly pea, rather than the gene duplication in scarlet runner bean and sword bean?
Reply: Our conclusion is based on the tree topology for NIN gene shown in Figure 5C. This tree suggests that the latest common ancestor of these 5 species have experienced a gene duplication event. Therefore, all the 5 species inherited two gene copies at first, then one copy lost in smooth rattlebox and butterfly pea.

10. Fig. 5 and Fig. 6, I did not observe any novel results. The comparisons among these five species cannot tell the unique gene copies, because the other legume species are not included.
Reply: Thanks for the reviewer's suggestion. We agree that Fig. 5 and Fig. 6 do not have significant scientific findings. Based on the reference genomes, we identified all the key genes involved in nitrogen-fixing root nodule symbiosis and resistance (R) gene-mediated defense for the 5 species in this study, providing a valuable resource for future functional studies. Then, we performed some simple and shallow evolutionary analyses for these genes. At current stage, we do not believe we can get solid conclusion by just adding more published legume species into the analyses, as we are lacking of accurate phenotype information. Finally, according to the editor's suggestion, we agree to change the article type of this manuscript into "Data Note".

Reviewer #2: The manuscript titled "The Genomes of Five Underutilized Papilionoideae Crops Provide Insights into Root Nodulation and Disease Resistance" describes the assembly of reference genomes for five Papilionoideae species. Although the genomic data generated in this study is relevant to advance our understanding of the genomic mechanisms that control adaptation in the five species used, I have some comments that could help to improve the manuscript before its publication:

Page 2: Abstract
Change "whole duplication (WGD)" to "Whole Genome Duplication".
Reply: Corrected.

Change "nodule symbiosis (RNS)" to "root-nodule symbiosis".
Reply: Corrected.

Define "LTR".
Reply: "LTR" was replaced by "LTR-RTs (Long terminal repeat-retrotransposons)".

Introduction:

Page 3: Replace subjective descriptions such as "the young bean pods are delicious vegetables" with more objective language.
Reply: this sentence was revised to "the young bean pods are used as vegetables".

The introduction does not mention the importance of disease resistance genes. Which references have compared resistance genes in legumes, and how can this help in breeding for species of this family?
Reply: In the revised manuscript, the first paragraph of "Evolutionary History of Resistance Genes" was moved to the introduction.

How were the lines that were sequenced selected? Are they representative of a specific marker class or gene pool?
Reply: We selected the lines of highly homozygous cultivars that were widely grown in China.

Page 8: In the section "TE sequences which are too difficult," remove "too".
Reply: Corrected.

Figure 5A does not present results and should be moved to the introduction.
Reply: We agree with the reviewer that Figure 5A do not present results. However, we do not want to show any figure in the introduction. We thought that put Figure 5A and 5B-D together will help integrate information. To make it clear, we added a sentence in Figure 5A legend "The data source is from previous studies,".

Figure 5B: Why are there differences across species? P. coccineous has double the number of CHS genes compared to C. pallida; what does this mean?
Reply: CHS is key gene for isoflavone biosynthesis. The gene number difference may influence the amount of isoflavone production in different species. However, we do not have accurate phenotype information, so we can't make further conclusion. We added a discussing sentence in the revised manuscript "The difference in CHS gene number may influence the amount of isoflavone production in different species, which needs further investigations in future.".

Page 9: Define "NFN".
Reply: Definition "NFN (Nitrogen-fixing Nodulation)" was provided in the revised manuscript.

Page 10: Replace "In study" with "In this study".
Reply: Corrected.

Page 11: The entire first paragraph of "Evolutionary History of Resistance Genes" should be moved to the introduction.
Reply: According to the reviewer's suggestion, we moved this paragraph into introduction.

Page 12:
Clarify the coverage of the five reference genomes that were assembled. What constitutes "Low-coverage" for the common bean reference genome?
Confirm whether the latest reference genome was used (https://phytozome-next.jgi.doe.gov/info/Pvulgaris_v2_1), which was created using PacBio with a coverage of 83X. If the latest reference genome of the common bean was not used, the analysis needs to be repeated.
Reply: Here the coverage means the ratio of assembled sequence in the whole genome. We have updated the analyses with the latest reference genome of the common bean, and largely revised the manuscript:

"We performed comparative genomic studies between the two Phaseolus plants scarlet runner bean (P. coccineus) and common bean (P. vulgaris) [7], which diverged from each other ~4.7 MYA (Figure 3A). Overall, all chromosomes from the two species have one-versus-one corresponding relationships, with only some intra-chromosome inversions (Figure 4A). The estimated genome sizes of the two species were both ~590 Mb. Our assembly size of scarlet runner bean is 593 Mb, almost equal to the estimated genome size. Meanwhile, the latest assembly size of common bean (NCBI: GCA_029448765.1) is 615 Mb, which is a little larger than the estimated genome size. Looking into the TEs, we found that 63.5% of the scarlet runner bean genome is composed of TEs, a little lower than that of the common bean genome (65%)

(Figure 4B).

Furthermore, the 35,523 protein-coding genes of scarlet runner bean were compared to the 42,801 protein-coding genes of common bean, which was annotated by our pipeline in this study. Requiring an alignment E-value of 1e-5, 30,195 (85.0%) scarlet runner bean genes and 32,367 (75.6%) common bean genes were matched, leaving 5,328 (15.0%) unique genes in scarlet runner and 10,434 (24.4%) unique genes in common bean (Figure 4C, Supplementary Table S18), which may be responsible for the phenotypic differences and species-specific functions between the two species.
”

Modify "we found a huge advantage of HiFi-assembly" to "we found an advantage of HiFi-assembly".
Reply: In the revised manuscript, this sentence was deleted.

Discuss whether the difference in the number of unique genes between scarlet runner bean and common bean is due to sequencing advantages or actual genomic differences.
Reply: When updated the analyses with the latest assembly of common bean, we found that in the old analyses, much of the difference in the number of unique genes should be due to the sequencing advantages, but not actual genomic differences. So we removed our previous conclusion, and largely revised the manuscript.

Elaborate on how "These genomic resources will promote evolutionary and comparative genomics studies in Fabaceae".
Reply: This sentence was revised into “The genomic resources in this study expand the lineage coverage in published Fabaceae species, which will promote evolutionary and comparative genomics studies in Fabaceae.”.

On the identification of key genes in root nodulation for the five studied plants, is this not an expected finding since all are nitrogen-fixing legumes? Is there a difference in efficiency? If so, is there an enrichment of particular genes, more copies, or greater gene expression in the more efficient species?
Reply: Yes, all the sequenced 5 plants are nitrogen-fixing legumes. As we do not have accurate phenotype information on nitrogen-fixing efficiency, so we can’t make further conclusions. With the annotated genes for each species, we performed gene copies analyses, and found that sword bean, scarlet runner bean and winged bean have one more NIN gene copy than smooth rattlebox and butterfly pea. Moreover, scarlet runner bean and winged bean each have two times more copies of CHS genes than the other 3 plants. These results have been described in the Result part. As this study focus on genome level analyses, so gene expression analyses for functional genes were not performed.

Correct "was detected for all the studies species" to "was detected for all the studied species".
Reply: Corrected.

Page 13: Remove "obviously" from "We also found that the CHS genes were obviously expanded through".
Reply: Corrected.

The information on R genes for the five studied plants belongs in the results section. It would be useful to compare the R genes across the five species to understand similarities and differenfces. How can this information be used to develop more resistant cultivars?
Reply: We have compared the gene number for each R gene class among the five species. However, we do not have accurate phenotype information on the disease resistance ability, so we can’t make further inference between the gene copy number and disease resistance ability.
In the revised manuscript, we added a discussing sentence “Considering that the 5 species in this study were much less domesticated than those well-cultivated legumes such as soybean, the 5 studied species here still have much stronger ability of disease resistance. Therefore, the identified R genes in these 5 species may be transported to major legume crops such as soybean to improve their ability of disease resistance.”.

Page 16: Correct the references for Papilionoideae species as they are incorrect in the sentence
Reply: Corrected.

Reviewer #3: The manuscript "The genomes of five underutilized Papilionoideae crops provide insights into root nodulation and disease resistance", authored by Yuan et al., presents the chromosome-length reference genomes of five crops: sword bean, scarlet runner bean, winged bean, smooth rattlebox, and butterfly pea. The manuscript is largely well-written and covers most of the analyses required for genome assembly and comparative study. However, there are a few areas that could be addressed to improve the manuscript's quality.

- Based on the Hi-C contact matrix for winged bean, it appears that the scaffolding for several chromosomes is not supported by Hi-C data. Additionally, according to Table 1, the genome assembly size for this species is higher than the estimated genome size. It would be advisable to check for contaminants in the assembly or correct the misjoins based on the Hi-C contact matrix.
Reply: For winged bean, the chromosome regions lacking Hi-C supports were repeat-rich centromeric regions. In fact, there are enough Hi-C data sequenced for these regions, but due to the requirement of unique mapping, most Hi-C data were filtered for these repeat-rich regions. Thus, they can't be shown in the Hi-C heatmap.

In Table 1, the estimated genome size by K-mer method is 689 Mb, while the assembled total contig size is 712 Mb, there is only ~23 Mb (3%) difference. Considering that the variations of the K-mer genome size estimation is about 5%, our genome size estimation generally consists with the genome assembly size .
Please refer to the GCE paper:
"Estimation of genomic characteristics by analyzing k-mer frequency in de novo genome projects. https://doi.org/10.48550/arXiv.1308.2012".

According to the reviewer's suggestion, we checked the assembly again. We did not find significant contaminants in our assembly, and we could not find any obvious misjoins based on Hi-C data.

- It appears the genome assembly for the Ma3 cultivar of winged bean was recently published (https://www.nature.com/articles/s41467-024-45048-x). Comparing the with Ma3 could support assembly quality and identify differences between the cultivars.
Reply: The Ma3 cultivar assembly (586 Mb) used Oxford nanopore reads with 85%-90% single base accuracy, while our DUOXI-ginseng cultivar (712 Mb) used HiFi reads with 90-99.9% single base accuracy. In theory, HiFi data has great advantage in distinguishing repetitive sequences over Oxford nanopore reads, therefore, our assembly includes a more comprehensive assembly of the centromere sequences. To verify this thought, we downloaded the published Ma3 cultivar genome, and compared with our DUOXI-ginseng cultivar genome. From the macrosynteny figure shown as Supplementary Fig. S4, we found that the two assembly were largely consistent in synteny, except for the middle centromere regions. The Ma3 cultivar assembly missed most of the centromere sequences, in total ~120 Mb, which explains why Ma3 cultivar (586 Mb) has a much smaller assembly size than DUOXI-ginseng cultivar (712 Mb).

However, the Ma3 cultivar assembly also has advantage over our assembly. For Chr06 in our assembly, the position of one chromosome segment seems incorrect due to the lack the Hi-C signal, we have corrected this mistake by referring to the Ma3 cultivar assembly, which has used a genetic map for scaffolding. Therefore, integrating all these genomic resources will generate a better assembly of winged bean.

We added the results of comparing analyses in the revised manuscript:
"In addition, we compared our DUOXI-ginseng cultivar assembly of winged bean (712 Mb) to the recently published Ma3 cultivar assembly (Figshare: https://doi.org/10.6084/m9.figshare.19196255) of winged bean (586 Mb), and found that our assembly has large advantage in resolving the repetitive centromere regions by using HiFi reads, while the Ma3 cultivar assembly has advantage in the chromosome-level scaffolding of contigs by using genetic map. The position of a chromosome segment in Chr06 of our assembly for winged bean was corrected according to the Ma3 cultivar assembly (Supplementary Fig. S4)."

- The genome used for the common bean seems to be outdated. More complete genomes are available, for example, Carrere et al., 2023 (https://www.sciencedirect.com/science/article/pii/S2352340923003013). The authors should utilize these newer genomes for a better comparison or provide reasoning for using the older reference genomes.
Reply: We have updated the analyses using the latest assembly of common bean. The updated results were shown in the revised manuscript:

"We performed comparative genomic studies between the two Phaseolus plants scarlet runner bean (P. coccineus) and common bean (P. vulgaris) [7], which diverged from each other ~4.7 MYA (Figure 3A). Overall, all chromosomes from the two species have one-versus-one corresponding relationships, with only some intra-chromosome inversions (Figure 4A). The estimated genome sizes of the two species were both ~590 Mb [39]. Our assembly size of scarlet runner bean is 593 Mb, almost equal to the estimated genome size. Meanwhile, the latest assembly size of common bean (NCBI: GCA_029448765.1) is 615 Mb, which is a little larger than the estimated genome size [7]. Looking into the TEs, we found that 63.5% of the scarlet runner bean genome is composed of TEs, a little lower than that of the common bean genome (65.0%) (Figure 4B, Supplementary Table S18).
Furthermore, the 35,523 protein-coding genes of scarlet runner bean were compared to the 42,801 protein-coding genes of common bean, which was annotated by our pipeline in this study. Requiring an alignment E-value of 1e-5, 30,195 (85.0%) scarlet runner bean genes and 32,367 (75.6%) common bean genes were matched, leaving 5,328 (15.0%) unique genes in scarlet runner and 10,434 (24.4%) unique genes in common bean (Figure 4C, Supplementary Table S18), which may be responsible for the phenotypic differences and species-specific functions between the two species."

- The expansion analysis is insufficient. The authors should conduct an unbiased contraction/expansion analysis using, for example, CAFE, and then identify significantly expanded/contracted gene families in the different species, rather than selectively citing gene families from the literature. This unbiased approach could help identify more gene families of interest that have not been reported before.
Reply: We have shown the CAFÉ results in Supplementary Fig. S6, which shows no genome-wide gene burst on the branches of five species in this study, indicating that there is no recent whole genome duplication like soybean.
To identify unbiased significantly expanded/contracted gene families in each species, the orthologous group results generated by OrthoFinder should be a good start. However, it is out of our interest to analyze more expanded or contracted gene families. According to the editor's suggestion, we decided to change the article type of this manuscript into "Data Note", which focuses on the primary analyses of the reference genomes.

- Including the common names of all species in the figures and tables would be beneficial, as these are referred to in the text, making it easier for readers to match the text with the figures and tables.
Reply: We thought it is more rigorous to use latin (scientific) names in the figures and tables. We have shown both the common names and latin (scientific) names for the first time appearance in the maintext, and then to be simple, we used the common names in the maintext, as the 5 species in this study are all people familiar trees.

- In the Introduction, the citation numbers for the genomes of the different crops are incorrect.
Reply: Corrected.

- On page 5, in the second paragraph, including the exact numbers in the text is unnecessary as they are already in Table 1. Mentioning just the range or average numbers could be more effective.

Reply: The sentence "The coding regions cover 51 Mb (8.2%), 42 Mb (7.1%), 44 Mb (7.4%), 51 Mb (4.2%), and 42 Mb (2.4%) of the genome for each species." was revised into "with the coding regions covering 2.4-8.2% of the genome for each species".
The sentence "For function annotation, 72.1%, 89.0%, 83.9%, 87.7% and 86.8% of genes in the 5 species were annotated" was revised into "For function annotation, 72.1-89.0% of genes in the 5 species were annotated".

- On page 7, the full form of PWGD should be provided.
Reply: The full form “PWGD, papilionoid-specific WGD” was provided in the revised manuscript.

- Several typographical and grammatical errors need to be corrected to enhance the manuscript's readability.
Reply: We have carefully checked and corrected the typographical and grammatical errors.

Editor comments:

The reviewers also mentioned (in their confidential comments to editor) that the story is not that attractive for the presence and minor copy variations of all the related genes, and the manuscript is more suitable for the GigaScience Data Note, rather than the regular research article. Therefore, we suggest you change the article type from "Research" to "Data Note".
Reply: We agree to change the article type into “Data Note”.
