# Supplementary material for: The genomes of 5 underutilized Papilionoideae crops provide insights into root nodulation and disease resistance
Source: Gigascience. 2024 Aug 27;13:giae063. doi: 10.1093/gigascience/giae063 (PMC11348429; doi:10.1093/gigascience/giae063)
Supplement: giae063_Response_to_Reviewer_Comments_Revision_1 [file giae063_response_to_reviewer_comments_revision_1.pdf]

Author's Response To Reviewer Comments

Reviewer reports:
Reviewer #1: I agree with the acceptance of this manuscript as Data Note, as it is improved in the revision.
1. Figure 5 and Figure 6 might be considered to be removed to Supplemental Materials, to comply with the potential requirement as Data Note.
Reply: 1. Figure 5 and Figure 6 were changed to Supplementary Figure S8 and Figure S11, respectively.
2. English polish service is strongly suggested for this manuscript before formal publication. For example, abstract, reapectively to respectively.
"we identified 5,328 and 10,434 species-specific unique genes between scarlet runner bean and common bean, for each species reapectively. " I don't understand this sentence with the current state. Do the authors mean 5,328 and 10,434 species-specific unique genes for scarlet runner bean and common bean respectively? I am concerned of more language problems.
Reply: We have corrected the mis-typo "reapectively" into "respectively", therefore, we believe this sentence is more easily understood now. In addition, the readers can get more details from the Result and Method parts of this manuscript. In the revised manuscript, we have largely improved the English language.

Reviewer #2: Please check the following comments:

Line 33: reapectively -> respectively
Reply: Corrected.

Line 105: we found that all the 5 sequencing materials -> we found that all sequenced materials
Reply: Corrected.

Line 112: QV value -> quality value (QV)
Reply: Corrected.

Line 113: indicating the very high accuracy -> indicating the high accuracy
Line 294: gene -> genes
Reply: Corrected.

Reviewer #3: The authors have addressed all my concerns. Also I note that the manuscript type has been changed to Data Note which is more apt for this manuscript. I still see few typing mistakes which needs to be corrected before the online publication of this manuscript.

Minor correction:
- Line 107: The assembly sizes mentioned are incorrect. It appears to be a typing mistake where "Mb" is mentioned instead of "Gb".
Reply: Corrected.
