# Supplementary material for: The genomes of 5 underutilized Papilionoideae crops provide insights into root nodulation and disease resistance
Source: Gigascience. 2024 Aug 27;13:giae063. doi: 10.1093/gigascience/giae063 (PMC11348429; doi:10.1093/gigascience/giae063)
Supplement: giae063_Reviewer_1_Report_Original_Submission — Shangang Jia -- 3/1/2024 Reviewed [file giae063_reviewer_1_report_original_submission.pdf]

Reviewer Report

Title: The genomes of five underutilized Papilionoideae crops provide insights into root nodulation and disease resistance

Version: Original Submission **Date:** 3/1/2024

Reviewer name: Shangang Jia

Reviewer Comments to Author:

The authors assembled five legume species' genomes, and conducted the comparisons. They searched the five genomes for genes involved in nitrogen-fixing root nodule symbiosis, phenylalanine pathway, and resistance. I got several suggestions for references.1. Why was the study designed with the genome assembly of 5 legume crops? Why selected these five species? This study concluded on several gene identification, such as the copy loss of known NIN gene.2. Abstract, full species names and genome ploid levels for the five species should be provided. "we identified 6,710 and 669 unique genes between scarlet runner bean and common bean for each species", I cannot understand this sentence. How were these two gene figures counted? Are these five species all diploid?3. Introduction, the latest legume genome should be cited.4. "67 Gb, 70 Gb, 64 Gb, 69 Gb, and 167 Gb HiFi data", the data coverage should be provided to evalute the sequencing data size for genome assembly.5. English should be polished for the whole manuscript body.6. "51,158, 35,523, 40,081, 48,759 and 40,267 protein-coding gene models" are from five genomes with 0.65 Gb, 0.59 Gb, 0.69 Gb, 1.33 Gb, and 1.76 Gb. Protein-coding gene number is expected to match the genome size. Why did 1.76 Gb genome contain only 40,267 genes? Is it reasonable and does it make sense? The authors should explain the rational of gene modules.7. What are the final genome size in addition to the estimated ones? The authors did not mention them in the manuscript.8. "At last, the Lb gene was expressed to produce leghemoglobin." Is it based on the data in this study?9. Why do the authors claim the loss of NIN gene copies in smooth rattlebox and butterfly pea, rather than the gene duplication in scarlet runner bean and sword bean?10. Fig. 5 and Fig. 6, I did not observe any novel results. The comparisons among these five species cannot tell the unique gene copies, because the other legume species are not included.

Level of Interest

Please indicate how interesting you found the manuscript: Choose an item.

Quality of Written English

Please indicate the quality of language in the manuscript: Choose an item.

Declaration of Competing Interests

Please complete a declaration of competing interests, considering the following questions:

- Have you in the past five years received reimbursements, fees, funding, or salary from an organisation that may in any way gain or lose financially from the publication of this manuscript, either now or in the future?
- Do you hold any stocks or shares in an organisation that may in any way gain or lose financially from the publication of this manuscript, either now or in the future?
- Do you hold or are you currently applying for any patents relating to the content of the manuscript?
- Have you received reimbursements, fees, funding, or salary from an organization that holds or has applied for patents relating to the content of the manuscript?
- Do you have any other financial competing interests?
- Do you have any non-financial competing interests in relation to this paper?

If you can answer no to all of the above, write 'I declare that I have no competing interests' below. If your reply is yes to any, please give details below.

I declare that I have no competing interests

I agree to the open peer review policy of the journal. I understand that my name will be included on my report to the authors and, if the manuscript is accepted for publication, my named report including any attachments I upload will be posted on the website along with the authors' responses. I agree for my report to be made available under an Open Access Creative Commons CC-BY license (http://creativecommons.org/licenses/by/4.0/). I understand that any comments which I do not wish to be included in my named report can be included as confidential comments to the editors, which will not be published.

Choose an item.

To further support our reviewers, we have joined with Publons, where you can gain additional credit to further highlight your hard work (see: https://publons.com/journal/530/gigascience). On publication of this paper, your review will be automatically added to Publons, you can then choose whether or not to claim your Publons credit. I understand this statement.

Yes Choose an item.
