# Supplementary material for: The genomes of 5 underutilized Papilionoideae crops provide insights into root nodulation and disease resistance
Source: Gigascience. 2024 Aug 27;13:giae063. doi: 10.1093/gigascience/giae063 (PMC11348429; doi:10.1093/gigascience/giae063)
Supplement: giae063_Reviewer_2_Report_Original_Submission — Paulo Izquierdo, Ph.D. -- 3/3/2024 Reviewed [file giae063_reviewer_2_report_original_submission.pdf]

**Reviewer Report**

**Title: The genomes of five underutilized Papilionoideae crops provide insights into root nodulation and disease resistance**

**Version: Original Submission** **Date:** 3/3/2024

**Reviewer name: Paulo Izquierdo, Ph.D.**

**Reviewer Comments to Author:**

The manuscript titled "The Genomes of Five Underutilized Papilionoideae Crops Provide Insights into Root Nodulation and Disease Resistance" describes the assembly of reference genomes for five Papilionoideae species. Although the genomic data generated in this study is relevant to advance our understanding of the genomic mechanisms that control adaptation in the five species used, I have some comments that could help to improve the manuscript before its publication:Page 2: AbstractChange "whole duplication (WGD)" to "Whole Genome Duplication".Change "nodule symbiosis (RNS)" to "root-nodule symbiosis".Define "LTR".Introduction:Page 3: Replace subjective descriptions such as "the young bean pods are delicious vegetables" with more objective language.The introduction does not mention the importance of disease resistance genes. Which references have compared resistance genes in legumes, and how can this help in breeding for species of this family?How were the lines that were sequenced selected? Are they representative of a specific marker class or gene pool?Page 8: In the section "TE sequences which are too difficult," remove "too".Figure 5A does not present results and should be moved to the introduction.Figure 5B: Why are there differences across species? P. coccineous has double the number of CHS genes compared to C. pallida; what does this mean?Page 9: Define "NFN".Page 10: Replace "In study" with "In this study".Page 11: The entire first paragraph of "Evolutionary History of Resistance Genes" should be moved to the introduction.Page 12:Clarify the coverage of the five reference genomes that were assembled.What constitutes "Low-coverage" for the common bean reference genome?Confirm whether the latest reference genome was used (https://phytozome-next.jgi.doe.gov/info/Pvulgaris_v2_1), which was created using PacBio with a coverage of 83X.If the latest reference genome of the common bean was not used, the analysis needs to be repeated.Modify "we found a huge advantage of HiFi-assembly" to "we found an advantage of HiFi-assembly".Discuss whether the difference in the number of unique genes between scarlet runner bean and common bean is due to sequencing advantages or actual genomic differences.Elaborate on how "These genomic resources will promote evolutionary and comparative genomics studies in Fabaceae".On the identification of key genes in root nodulation for the five studied plants, is this not an expected finding since all are nitrogen-fixing legumes? Is there a difference in efficiency? If so, is there an enrichment of particular genes, more copies, or greater gene expression in the more efficient species?Correct "was detected for all the studies species" to "was detected for all the studied species".Page 13: Remove "obviously" from "We also found that the CHS genes were obviously expanded through".The information on R genes for the five studied plants belongs in the results section. It would be useful to compare the R genes across the five species to understand similarities and

differences. How can this information be used to develop more resistant cultivars?Page 16: Correct the references for Papilionoideae species as they are incorrect in the sentence

**Level of Interest**

Please indicate how interesting you found the manuscript: Choose an item.

**Quality of Written English**

Please indicate the quality of language in the manuscript: Choose an item.

**Declaration of Competing Interests**

Please complete a declaration of competing interests, considering the following questions:

- Have you in the past five years received reimbursements, fees, funding, or salary from an organisation that may in any way gain or lose financially from the publication of this manuscript, either now or in the future?
- Do you hold any stocks or shares in an organisation that may in any way gain or lose financially from the publication of this manuscript, either now or in the future?
- Do you hold or are you currently applying for any patents relating to the content of the manuscript?
- Have you received reimbursements, fees, funding, or salary from an organization that holds or has applied for patents relating to the content of the manuscript?
- Do you have any other financial competing interests?
- Do you have any non-financial competing interests in relation to this paper?

If you can answer no to all of the above, write 'I declare that I have no competing interests' below. If your reply is yes to any, please give details below.

I declare that I have no competing interests

I agree to the open peer review policy of the journal. I understand that my name will be included on my report to the authors and, if the manuscript is accepted for publication, my named report including any attachments I upload will be posted on the website along with the authors' responses. I agree for my report to be made available under an Open Access Creative Commons CC-BY license (http://creativecommons.org/licenses/by/4.0/). I understand that any comments which I do not wish to be included in my named report can be included as confidential comments to the editors, which will not be published.

Choose an item.

To further support our reviewers, we have joined with Publons, where you can gain additional credit to further highlight your hard work (see: https://publons.com/journal/530/gigascience). On publication of this paper, your review will be automatically added to Publons, you can then choose whether or not to claim your Publons credit. I understand this statement.

Yes Choose an item.
