# Supplementary material for: The genomes of 5 underutilized Papilionoideae crops provide insights into root nodulation and disease resistance
Source: Gigascience. 2024 Aug 27;13:giae063. doi: 10.1093/gigascience/giae063 (PMC11348429; doi:10.1093/gigascience/giae063)
Supplement: giae063_Reviewer_3_Report_Original_Submission — Vanika Garg -- 3/9/2024 Reviewed [file giae063_reviewer_3_report_original_submission.pdf]

Reviewer Report

Title: The genomes of five underutilized Papilionoideae crops provide insights into root nodulation and disease resistance

Version: Original Submission **Date:** 3/9/2024

Reviewer name: Vanika Garg

Reviewer Comments to Author:

The manuscript "The genomes of five underutilized Papilionoideae crops provide insights into root nodulation and disease resistance", authored by Yuan et al., presents the chromosome-length reference genomes of five crops: sword bean, scarlet runner bean, winged bean, smooth rattlebox, and butterfly pea. The manuscript is largely well-written and covers most of the analyses required for genome assembly and comparative study. However, there are a few areas that could be addressed to improve the manuscript's quality.- Based on the Hi-C contact matrix for winged bean, it appears that the scaffolding for several chromosomes is not supported by Hi-C data. Additionally, according to Table 1, the genome assembly size for this species is higher than the estimated genome size. It would be advisable to check for contaminants in the assembly or correct the misjoins based on the Hi-C contact matrix.- It appears the genome assembly for the Ma3 cultivar of winged bean was recently published (https://www.nature.com/articles/s41467-024-45048-x). Comparing the DUOXI-ginseng with Ma3 could support assembly quality and identify differences between the cultivars.- The genome used for the common bean seems to be outdated. More complete genomes are available, for example, Carrere et al., 2023 (https://www.sciencedirect.com/science/article/pii/S2352340923003013). The authors should utilize these newer genomes for a better comparison or provide reasoning for using the older reference genomes.- The expansion analysis is insufficient. The authors should conduct an unbiased contraction/expansion analysis using, for example, CAFE, and then identify significantly expanded/contracted gene families in the different species, rather than selectively citing gene families from the literature. This unbiased approach could help identify more gene families of interest that have not been reported before.- Including the common names of all species in the figures and tables would be beneficial, as these are referred to in the text, making it easier for readers to match the text with the figures and tables.- In the Introduction, the citation numbers for the genomes of the different crops are incorrect.- On page 5, in the second paragraph, including the exact numbers in the text is unnecessary as they are already in Table 1. Mentioning just the range or average numbers could be more effective.- On page 7, the full form of PWGD should be provided.- Several typographical and grammatical errors need to be corrected to enhance the manuscript's readability.

Level of Interest

Please indicate how interesting you found the manuscript: Choose an item.

Quality of Written English

Please indicate the quality of language in the manuscript: Choose an item.

Declaration of Competing Interests

Please complete a declaration of competing interests, considering the following questions:

- Have you in the past five years received reimbursements, fees, funding, or salary from an organisation that may in any way gain or lose financially from the publication of this manuscript, either now or in the future?
- Do you hold any stocks or shares in an organisation that may in any way gain or lose financially from the publication of this manuscript, either now or in the future?
- Do you hold or are you currently applying for any patents relating to the content of the manuscript?
- Have you received reimbursements, fees, funding, or salary from an organization that holds or has applied for patents relating to the content of the manuscript?
- Do you have any other financial competing interests?
- Do you have any non-financial competing interests in relation to this paper?

If you can answer no to all of the above, write 'I declare that I have no competing interests' below. If your reply is yes to any, please give details below.

I declare that I have no competing interests

I agree to the open peer review policy of the journal. I understand that my name will be included on my report to the authors and, if the manuscript is accepted for publication, my named report including any attachments I upload will be posted on the website along with the authors' responses. I agree for my report to be made available under an Open Access Creative Commons CC-BY license (http://creativecommons.org/licenses/by/4.0/). I understand that any comments which I do not wish to be included in my named report can be included as confidential comments to the editors, which will not be published.

Choose an item.

To further support our reviewers, we have joined with Publons, where you can gain additional credit to further highlight your hard work (see: https://publons.com/journal/530/gigascience). On publication of this paper, your review will be automatically added to Publons, you can then choose whether or not to claim your Publons credit. I understand this statement.

Yes Choose an item.
